# The influence of acceptor nucleophilicity on the glycosylation reaction mechanism†
†Electronic supplementary information (ESI) available. See DOI: 10.1039/c6sc04638j



**DOI:** 10.1039/c6sc04638j

**Published:** 2016-11-09

**Authors:** S. van der Vorm, T. Hansen, H. S. Overkleeft, G. A. van der Marel, J. D. C. Codée

**Affiliations:** a Leiden Institute of Chemistry , Leiden University , Einsteinweg 55 , 2333 CC Leiden , The Netherlands . Email: jcodee@chem.leidenuniv.nl

## Abstract

A set of model nucleophiles of gradually changing nucleophilicity is used to probe the glycosylation reaction mechanism. Glycosylations of ethanol-based acceptors, bearing varying amounts of fluorine atoms, report on the dependency of the stereochemistry in condensation reactions on the nucleophilicity of the acceptor. Three different glycosylation systems were scrutinized, that differ in the reaction mechanism, that – putatively – prevails during the coupling reaction. It is revealed that the stereoselectivity in glycosylations of benzylidene protected glucose donors are very susceptible to acceptor nucleophilicity whereas condensations of benzylidene mannose and mannuronic acid donors represent more robust glycosylation systems in terms of diastereoselectivity. The change in stereoselectivity with decreasing acceptor nucleophilicity is related to a change in reaction mechanism shifting from the S_N_2 side to the S_N_1 side of the reactivity spectrum. Carbohydrate acceptors are examined and the reactivity–selectivity profile of these nucleophiles mirrored those of the model acceptors studied. The set of model ethanol acceptors thus provides a simple and effective “toolbox” to investigate glycosylation reaction mechanisms and report on the robustness of glycosylation protocols.

## Abstract 2

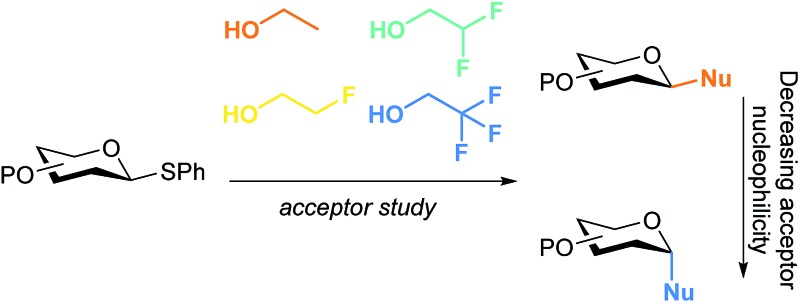
The acceptor dependence on the glycosylation stereoselectivity is revealed by a systematic study employing model acceptors of gradually changing nucleophilicity.

## Introduction

**Fig. 1:**
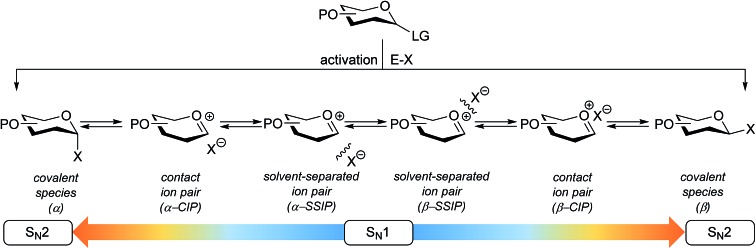
The reaction mechanism manifold operational during glycosylation reactions.

The connection of two carbohydrate building blocks to construct a glycosidic linkage in a glycosylation reaction is one of the most important and one of the most difficult steps in the assembly of an oligosaccharide.^1–3^ The stereoselective formation of 1,2-*cis* glycosidic linkages remains a major synthetic challenge and often requires careful tuning of reaction conditions for a profitable outcome.^4^ The variation in stereochemical outcome of a chemical glycosylation reaction originates from the different mechanistic pathways that can be followed for the union of an activated donor glycoside and an acceptor. Fig. 1 depicts the current understanding of the continuum of mechanisms operational during a glycosylation reaction. The activation of a donor glycoside leads to an array of reactive intermediates, formed from the donor glycoside and the activator derived counterion. α- and β-configured covalent reactive intermediates can be formed and these are in equilibrium with less stable and more reactive oxocarbenium ion based species. These can be either closely associated with the counterion providing close (or contact) ion pairs (CIPs), or further separated from their counterion in solvent separated ion pairs (SSIPs). These reactive intermediates can be attacked by an incoming nucleophile following a reaction mechanism with both S_N_1 and S_N_2 features. The covalent species are displaced in a reaction mechanism having an associative S_N_2-character, while the oxocarbenium ion-like intermediates are engaged in an S_N_1-like reaction. The exact position(s) on the continuum where a given glycosylation reaction takes place, and hence the stereoselectivity of the process, depends critically on the reactivity of both reaction partners: the donor and acceptor glycoside. The impact of the reactivity of the donor glycoside on the stereochemical outcome has been studied extensively, and the effect of functional and protecting groups on glycosyl donor reactivity is well documented.^5–10^ In contrast, the influence of the reactivity of the nucleophile (the acceptor) on the outcome of a glycosylation reaction remains poorly understood.^11–18^ We here present a systematic study to determine the effect of acceptor nucleophilicity on the stereochemical course of a glycosylation reaction. We show how a simple “toolset” of partially fluorinated alcohols^13^ can be used to dissect reaction mechanisms that are operational during a glycosylation reaction. It is revealed that the stereoselectivity of some glycosylation systems varies more with changing acceptor nucleophilicity than others and we relate these differences to changes in reaction pathways that are followed. A panel of model carbohydrate acceptors is scrutinized to place the reactivity of these building blocks in the context of the nucleophilicity scale set by the series of fluorinated ethanols.

## Results and discussion

**Fig. 2:**
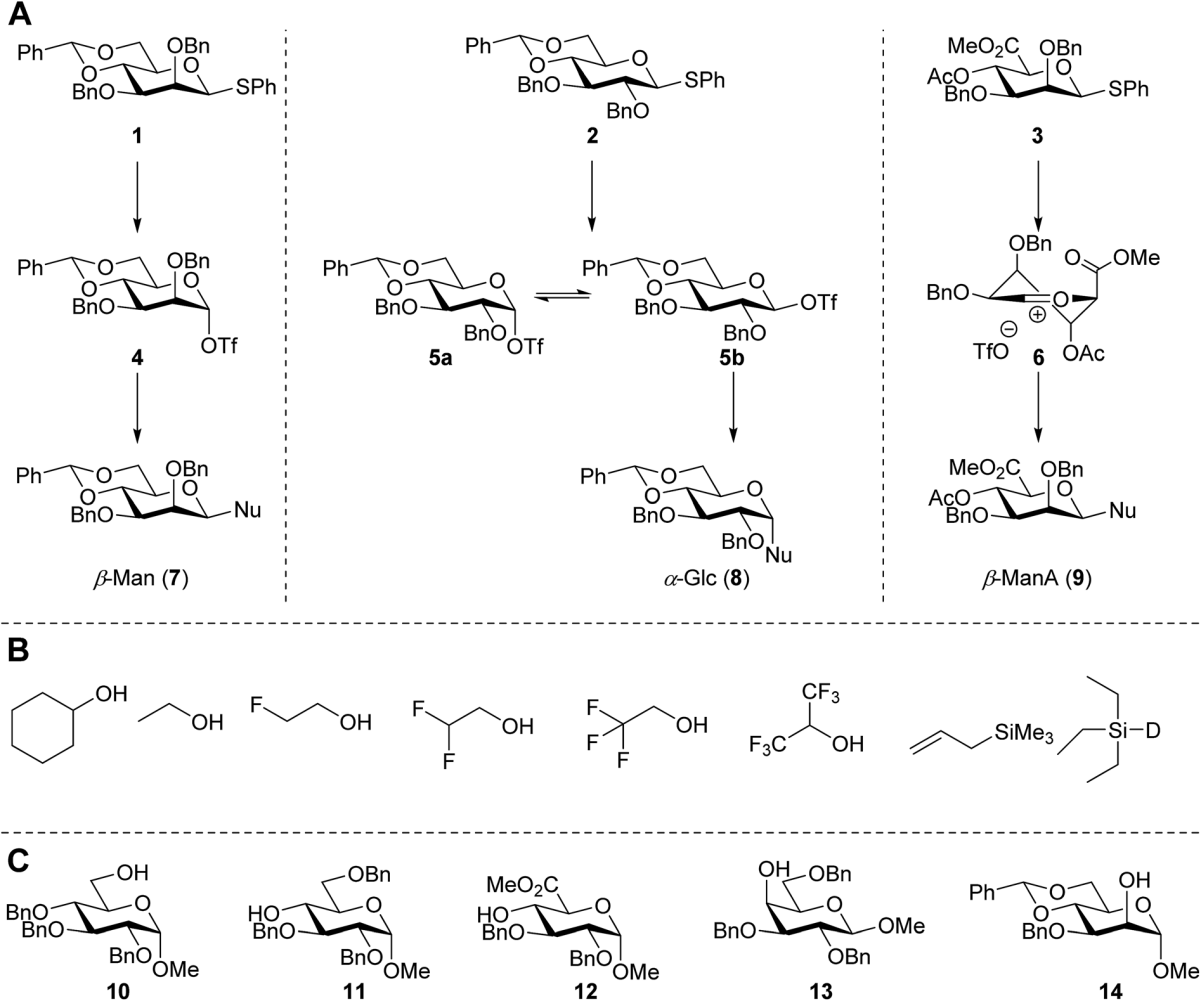
(A) The benzylidene mannose, benzylidene glucose and mannuronic acid glycosylation systems studied and the major glycosylation pathways of these donors. (B) Set of model nucleophiles used in this study. (C) Set of carbohydrate alcohols used.

In this study the effect of acceptor nucleophilicity on the glycosylation selectivity is systematically investigated by the hand of a set of model *O*-nucleophiles, encompassing ethanol, monofluoroethanol (MFE), difluoroethanol (DFE), trifluoroethanol (TFE), hexafluoro-*iso*-propanol (HFIP) and cyclohexanol, as well as a *C*-nucleophile, allyltrimethylsilane (allyl-TMS), and a deuterium nucleophile, deuterated triethylsilane (TES-D).^12,13^ Next a series of carbohydrate acceptors is used to place the reactivity of these alcohols in the context of the reactivity of the ethanol model acceptors (see Fig. 2B and C). Three glycosylation systems have been investigated with these acceptors: the benzylidene mannose and analogous benzylidene glucose system as well as the mannuronic acid system (see Fig. 2A). These systems have been selected because they have previously been studied in depth to provide insight into the major reaction pathways that operate during glycosylation reactions of these donors (*vide infra*). Although these three glycosylation systems all selectively provide 1,2-*cis*-products, the major product-forming pathways significantly differ.

The benzylidene mannose system, introduced by Crich and co-workers for the stereoselective construction of β-mannosidc linkages, represents the best studied glycosylation system to date.^19,20^ It has been found that benzylidene mannose donors can be transformed into the corresponding α-anomeric triflate **4** upon activation. These triflates have been extensively characterized in variable temperature NMR studies.^21–24^ A significant body of evidence has been gathered through a vast amount of glycosylation reactions,^19–23,25–33^ the establishment of kinetic isotope effects in combination with computational methods,^34,35^ and the application of cation clock methodology,^36–38^ to indicate that these triflates can be substituted in an S_N_2-manner to provide β-mannosides. However, an alternative hypothesis to account for the β-selectivity of benzylidene mannose glycosylations has also been forwarded. This hypothesis is based on a *B*_2,5_-oxocarbenium ion as product forming intermediate.^39–42^

The closely related benzylidene glucose system provides α-selective glycosylation reactions.^21,22,29,40,43–47^ It has been proposed that this selectivity originates from an *in situ* anomerization kinetic scheme, in which the initially formed α-triflate **5α** anomerizes into its more reactive β-couterpart **5β**.^21^ Substitution of this species provides the α-glucosyl products. Mechanistic studies, amongst others kinetic isotope effect and cation clock experiments, using the reactive nucleophile *iso*-propanol have provided support for this pathway.^34,37,38^

Glycosylations of mannuronic acids have been shown to proceed in a highly selective manner to provide β-mannuronic acid products. Based on the conformational behavior of the donors and the intermediate α-triflates **18α**, adopting an ^1^ *C*_4_ conformation,^48,49^ the high reactivity of these donors^50,51^ and a large variety of glycosylation reactions, both in solution,^50,52–55^ and on fluorous^56^ and solid supports,^57^ it has been postulated that the selectivity in these glycosylation reactions can be related to the intermediacy of an ^4^ *H*_3_ oxocarbenium ion-like intermediate.^53,54,58^

The experimental setup that we used in this study is based on pre-activation of the thioglycoside donors **1**,^59^ **2** ^21^ and **3** using a slight excess of diphenyl sulfoxide and triflic anhydride (Ph_2_SO/Tf_2_O) at low temperature. This transforms all three donors into the corresponding anomeric triflates,^21–24,48,60^ prior to addition of the acceptor nucleophiles. The pre-activation set-up generates a pool of reactive intermediates in the absence of the acceptor, thereby eliminating product forming pathways that originate from direct displacement reactions on the activated parent donor species. Table 1 summarizes the results obtained with the three donor systems and the set of model acceptors. As a measure for the reactivity of the used acceptors, Mayr's nucleophilicity parameters have been tabularized where available.^61–63^ The field inductive parameters for the –CH_3_, –CH_2_F, –CHF_2_ and –CF_3_ groups have also been shown to indicate the gradual increase of electron withdrawing character of these groups.^64^

**Table 1:** Model acceptor glycosylations

Acceptor	*N* ^*a*^	*F* ^*b*^	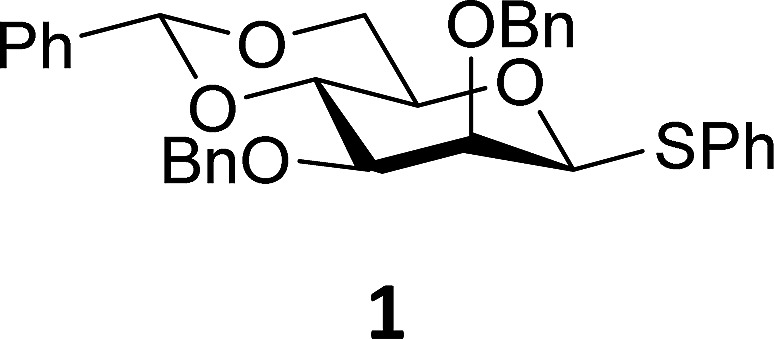	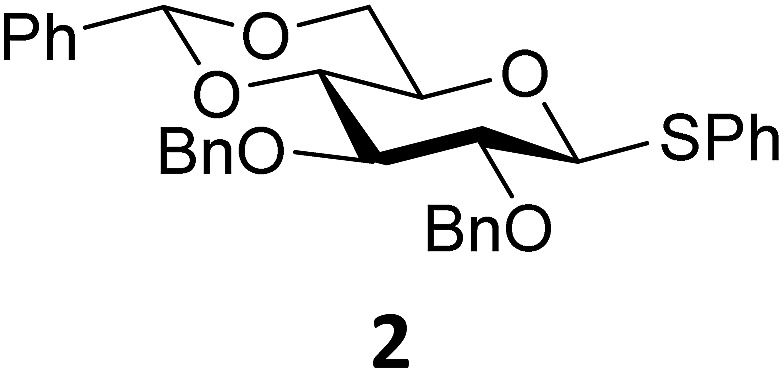	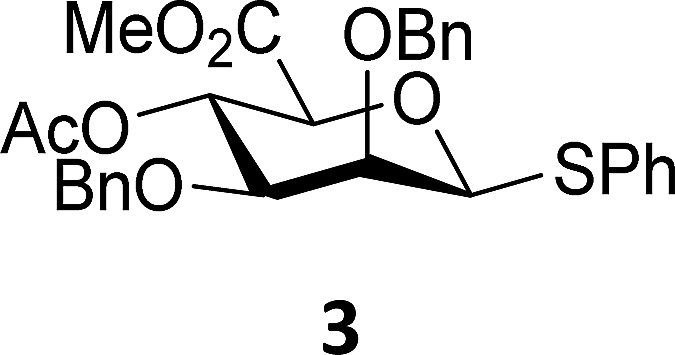
			Product, α : β (yield)^*c*^	Product, α : β (yield)^*c*^	Product, α : β (yield)^*c*^
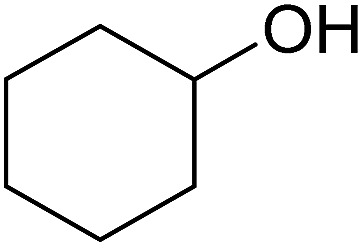	—	—	**1A**	**2A**	**3A**
			1 : 6	1 : 5	1 : 8
			(96%)	(71%)	(83%)
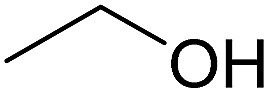	7.44	0.01	**1B**	**2B**	**3B**
			1 : 5	1 : 10	1 : 8
			(70%)	(68%)	(95%)
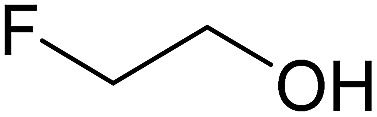	—	0.15	**1C**	**2C**	**3C**
			1 : 5	1 : 3	1 : 6
			(86%)	(70%)	(70%)
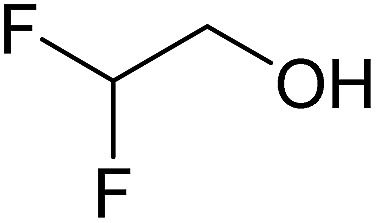	—	0.29	**1D**	**2D**	**3D**
			1 : 5	5 : 1	1 : 5
			(90%)	(70%)	(87%)
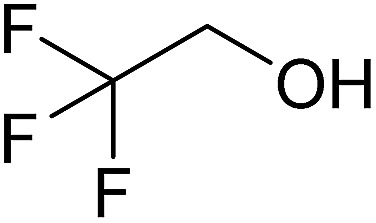	1.11	0.38	**1E**	**2E**	**3E**
			1 : 4	>20 : 1	1 : 2.5
			(78%)	(64%)	(85%)
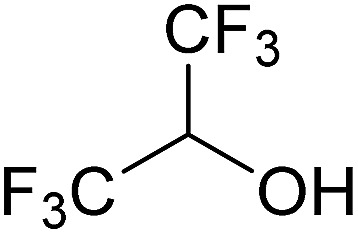	–1.93	—	**1F**	**2F**	**3F**
			3 : 1	>20 : 1	1 : 1
			(56%)	(65%)	(52%)
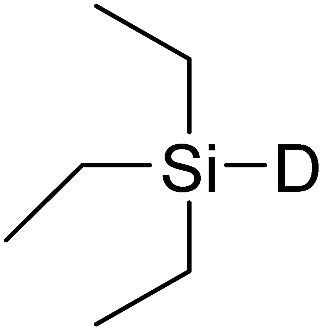	3.58	—	**1G**	**2G**	**3G**
			<1 : 20	>20 : 1	<1 : 20
			(60%)	(79%)	(95%)
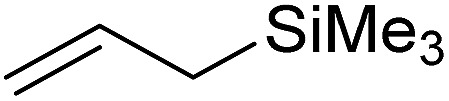	1.68	—	**1H**	**2H**	**3H**
			<1 : 20	>20 : 1	<1 : 20
			(44%)^*d*^	(42%)^*d*^	(40%)^*d*^

^*a*^Mayr's nucleophilicity parameters.

^*b*^Field inductive parameters.

^*c*^α/β-Ratios were established by NMR spectroscopy of the crude and purified reaction mixtures.

^*d*^Both anomers of donor glycoside were also found after the glycosylation reaction. Literature yields of **1H** ^40^: 57% and **2H** ^40^: 56%.

**Fig. 3:**
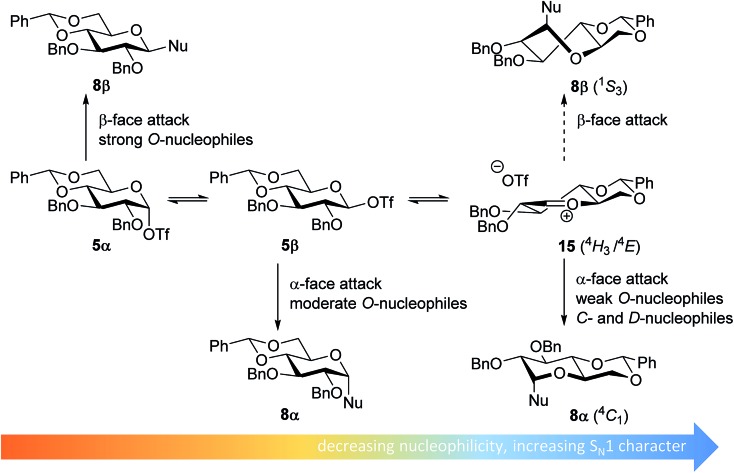
Mechanistic pathways to account for the selectivity in glycosylations of benzylidene glucose donors.

From the results depicted in Table 1 it becomes immediately apparent that the stereoselectivity of the benzylidene mannose and mannuronic acid systems shows relatively little variation with changing nucleophilicity, where the stereoselectivity of the glycosylations involving the benzylidene glucose donor changes significantly depending on the reactivity of the used nucleophile. Reactive nucleophiles such as ethanol, cyclohexanol and MFE predominantly provide β-linked products (**2A**, **2B** and **2C**), where the use of less reactive nucleophiles such as DFE, TFE, HFIP, TES-D and allyl-TMS leads to the preferential formation of the α-glucosyl products (**2D–2H**). A clear trend becomes apparent between the reactivity of the non-fluorinated and partially fluorinated ethanols and the stereoselectivity of the glucosylations involving these acceptors. The formation of the β-linked products **2A**,^65^ **2B** and **2C** can be explained to originate from an S_N_2-like substitution on the intermediate α-triflate **5α** (see Fig. 3). The α-products in these glucosylations (**α-2A**, **α-2B**, **α-2C**) may be formed from the corresponding β-glucosyl triflate **5β**, as postulated by Crich and co-workers and as supported by kinetic isotope effect and cation clock studies.^34,35,37,66^ It is however less likely that the unreactive *O*-nucleophiles, such as TFE and HFIP, and the weak *C*- and *D*-nucleophiles, are capable of displacing the anomeric triflate **5** in an S_N_2-manner. Woerpel and co-workers have previously shown that TFE requires a glycosylating agent bearing significant oxocarbenium ion character.^13^ An explanation for the observed α-selectivity in the glucosylations of these nucleophiles may be found in the S_N_1-like substitution on the benzylidene glucose oxocarbenium ion **15**. This ion preferentially adopts a ^4^ *H*_3_/^4^ *E*-structure, as verified by several computational studies,^67,68^ that is attacked in a diastereoselective fashion from the bottom face, leading *via* a chair-like transition state to the α-linked products. As the reactivity of the nucleophile diminishes, it is likely that the amount of S_N_2-character in the substitution of the β-triflate **5β** gradually decreases and the amount of S_N_1-character with the intermediacy of the corresponding CIP and SSIP (**15**) increases.^13^ The least reactive nucleophiles require the most “naked” oxocarbenium ions, with the triflate counterions significantly, if not completely, dissociated from the carbohydrate ring.

**Fig. 4:**
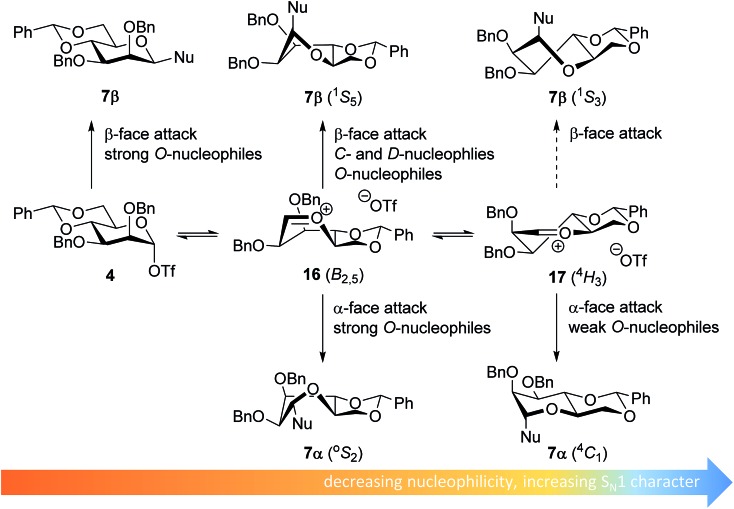
Mechanistic pathways to account for the selectivity in glycosylations of benzylidene mannose donors.

The stereoselectivity of the benzylidene mannose systems seems to be less sensitive to variation in nucleophilicity of the acceptor. Donor **1** provides β-selective glycosylations with the range of acceptors studied. There is a slight decrease in selectivity going from the reactive *O*-nucleophiles to the weak *O*-nucleophiles and the condensation of benzylidene mannose **1** with HFIP proceeds with moderate α-selectivity. The most likely explanation for the β-selectivity observed with the reactive *O*-nucleophiles is an associative S_N_2-type substitution of the intermediate α-triflate **4** (see Fig. 4). As discussed above, it is unlikely that unreactive acceptors such as TFE and HFIP react in an S_N_2-type reaction, directly displacing the α-mannosyl triflate **4**. Formation of the β-linked products formed from the unreactive acceptors and donor **1** may be better explained with an oxocarbenium ion-like product forming intermediate. Various theoretical studies have indicated that the *B*_2,5_-oxocarbenium ion **16** is the most stable benzylidene mannose oxocarbenium ion conformer.^67,68^ This oxocarbenium ion is preferentially attacked from the convex top-face, as attack from the bottom face would lead to unfavorable interactions with the pseudo-axial H-2 and to an eclipsed C-1–C-2 configuration upon rehybridization.^36,40,69,70^

The α-products formed in the condensations of donors **1** likely originate from an oxocarbenium ion intermediate. Reactive *O*-nucleophiles may react with an oxocarbenium ion in a relatively indiscriminative manner leading to the formation of both α- and β-products.^11–13^ Because unreactive *O*-nucleophiles are expected to react in a more diastereoselective fashion with an oxocarbenium ion, it is unlikely that the α-products derived from the weak *O*-nucleophiles, such as TFE and HFIP, originate from the *B*_2,5_-oxocarbenium ion **15**. Instead, α-face attack on the ^4^ *H*_3_ half chair conformer **17** may be a plausible reaction pathway to account for the α-products of the less reactive *O*-nucleophiles. In a later transition state, product development control plays a more important role and the developing anomeric effect and the low energy chair conformation that results from the α-face attack on the ^4^ *H*_3_ half chair **17**, make this trajectory favorable.^71^ For the weak *C*- and *D*-nucleophiles, which react in a highly selective β-manner, this latter pathway does not play a major role, and these nucleophiles attack the *B*_2,5_-oxocarbenium ion **16** selectively from the top face.^40,72^

**Fig. 5:**
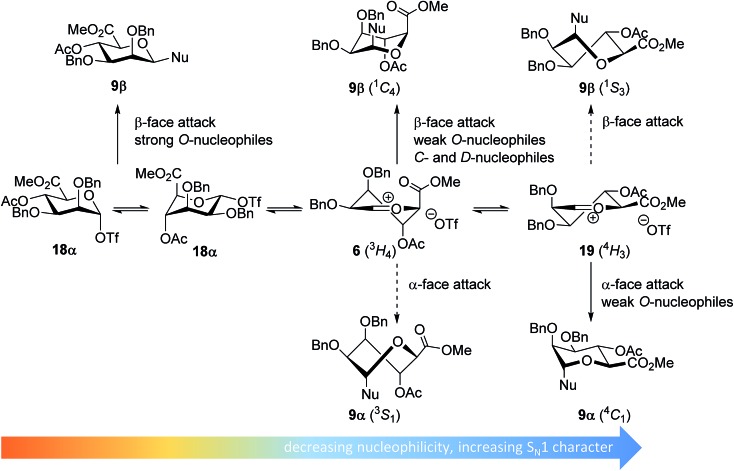
Mechanistic pathways to account for the selectivity in glycosylations of mannuronic acid donors.

In line with the benzylidene mannose system, the mannuronic acid donor provides β-selective condensations with all acceptors explored, except with the very unreactive *O*-nucleophile HFIP where both anomers were formed in equal amounts. Where reactions with nucleophilic *O*-nucleophiles can be expected to form from the α-triflate **18α**,^34–37^ the weaker *O*-nucleophiles and allyl-TMS and TES-D will react preferentially with an oxocarbenium ion (Fig. 5). We have previously postulated that the ^3^H_4_ half chair mannuronic acid oxocarbenium ion **6** is the most stable oxocarbenium ion conformer.^51,54,55^ To substantiate this hypothesis, we have calculated the energy associated with a range of mannuronic acid oxocarbenium ion conformers (see Fig. 5 and ESI†) using DFT-calculations at the B3LYP/6-311G level.^73^ From these calculations the ^3^ *H*_4_ conformer **6** appears to be significantly more stable (by >5 kcal mol^–1^) than other conformers such as the alternative ^4^ *H*_3_ half chair **19** and the *B*_2,5_ boat conformers. The relative stability of the ^3^ *H*_4_ half chair oxocarbenium ion can be explained by favorable interaction of the ring substituents with the electron depleted carbocation. Hyperconjugative stabilization of the C-2–H-2 bond and through space stabilization of the pseudo-axial C-3, C-4 oxygen atoms and the axial C-5 carboxylate each contribute to the stability of the half chair oxocarbenium ion.^51,54,74–76^ This oxocarbenium ion is preferentially attacked from the top face to provide the β-linked products *via* a chair-like transition state. For the weaker *O*-nucleophiles, a later transition state leads to significant steric interactions with the axial substituents in the ^3^ *H*_4_ half chair oxocarbenium **6** and a reaction pathway, involving attack of the nucleophiles on the higher energy ^4^ *H*_3_ half chair oxocarbenium ion **19** becomes relevant. In line with the discussion above, product development control is favorable for the formation of α-*O*-mannuronic acids.

Next, we explored the set of carbohydrate acceptors depicted in Fig. 2C. The results of these condensation reactions are summarized in Table 2. Where it can be reasoned that the secondary carbohydrate acceptors **11**,^77^ **12**,^78^ **13** ^77^ and **14** ^79^ electronically resemble DFE and TFE, because of the amount of electron withdrawing β- and/or γ- and δ-substituents, the size of the carbohydrate acceptors obviously differs significantly from the small ethanol based acceptors. The picture that emerges from Table 2 follows in broad lines the results described in Table 1 and corroborates this analysis. The benzylidene glucose donor system **2** shows most variation in stereoselectivity, where both the benzylidene mannose and mannuronic acid donors **1** and **3** provide β-selective reactions with all carbohydrate acceptors studied. The series of benzylidene glucose condensations again reveals that reactive *O*-nucleophiles can provide β-selective glycosylations, while less reactive *O*-nucleophiles give the α-linked products. The electron withdrawing effect of the C-5 carboxylate in acceptor **12**, makes this acceptor less reactive and more α-selective than its C-5-benzyloxymethylene counterpart **11**. In line with the discussion above, formation of the β-linked products can be explained with triflate **5α** as product forming intermediate. Less reactive acceptors require a glycosylating species that is more electrophilic and react in a more dissociative substitution reaction, with a substantial amount of oxocarbenium ion character and the glucose ring taking up a ^4^ *H*_3_-like structure (**15**).

**Table 2:** Glycosylation of donors **1–3** with carbohydrate acceptors

Acceptor	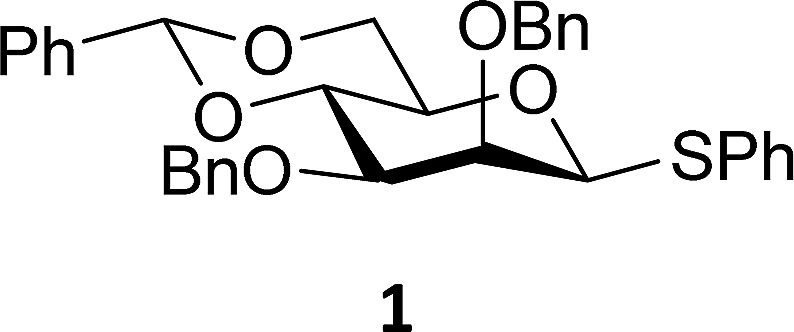	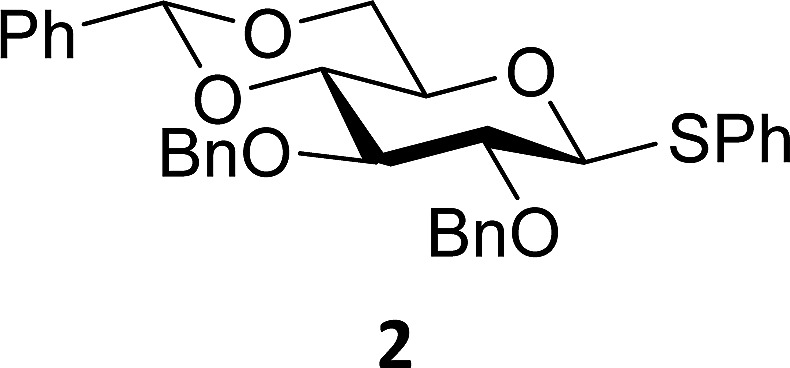	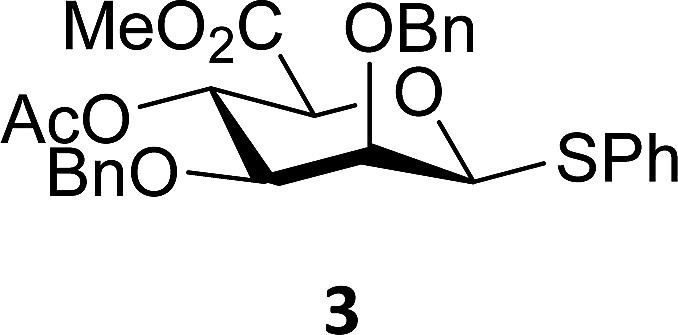
	Product, α : β (yield)^*c*^	Product, α : β (yield)^*c*^	Product, α : β (yield)^*c*^
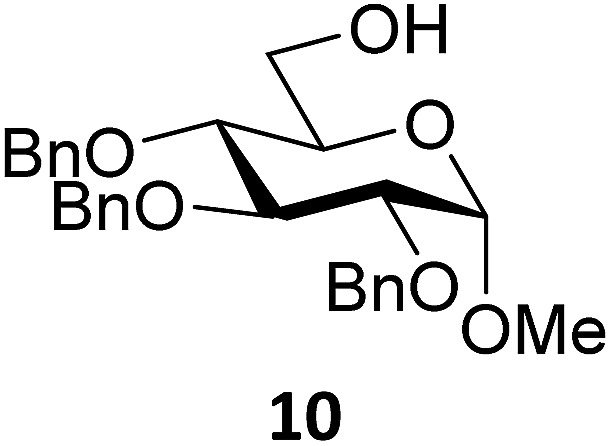	**20**	**25**	**30**
	1 : 10	1 : 3	<1 : 20
	(97%)	(81%)	(71%)
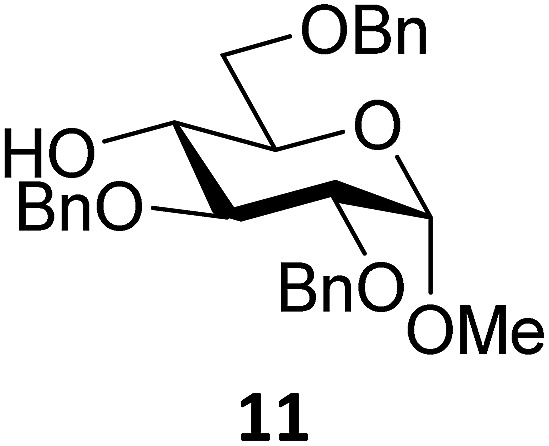	**21**	**26**	**31**
	1 : 9	1 : 1	<1 : 20
	(75%)	(79%)	(61%)
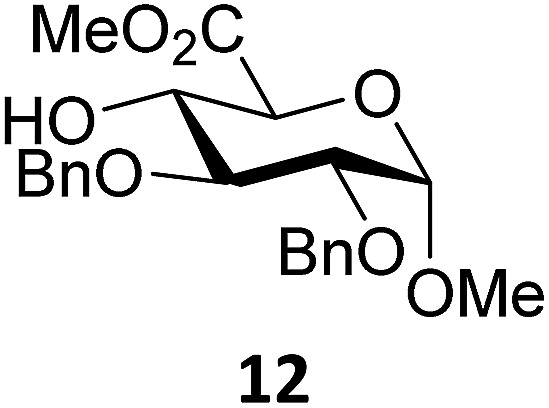	**22**	**27**	**32**
	1 : 10	5 : 1	1 : 10
	(87%)	(90%)	(71%)
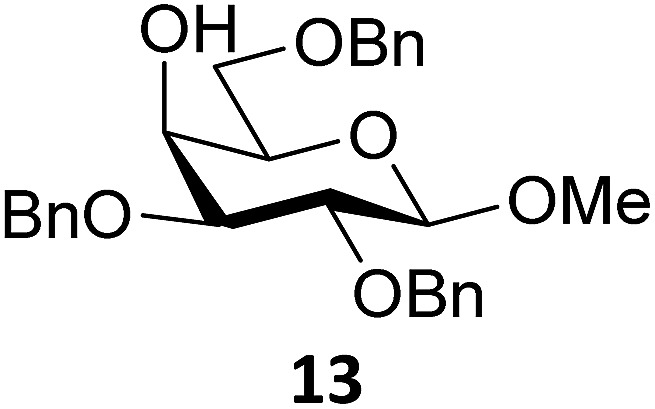	**23**	**28**	**33**
	<1 : 20	>20 : 1	<1 : 20
	(70%)	(83%)	(76%)
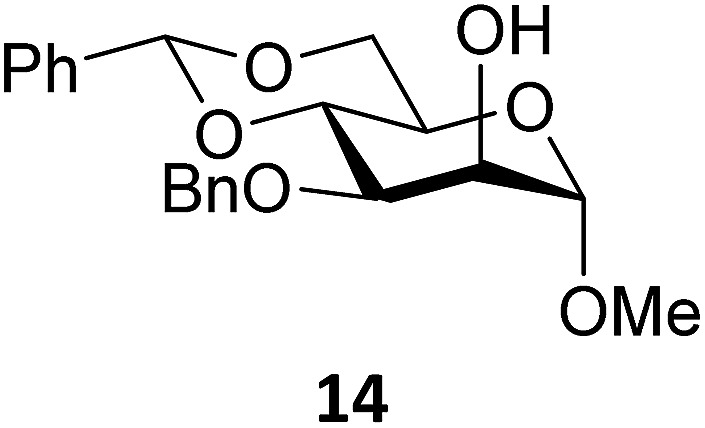	**24**	**29**	**34**
	<1 : 20	>20 : 1	1 : 7
	(87%)	(80%)	(80%)

The benzylidene mannose and mannuronic acid donors **1** and **3** provide very β-selective condensation reactions, in line with the vast amount of previously reported glycosylations of these two donors. Based on the results presented here and in previous work the following picture emerges. Reactive carbohydrate acceptors react in a reaction with significant S_N_2-character, displacing the anomeric α-triflate (**4** and **18α**). Weaker nucleophiles, such as most secondary carbohydrate acceptors, will react with a species that bears more carbocation character. For the benzylidene mannose donor, this species will resemble *B*_2,5_ boat oxocarbenium ion **16**, where the reactive mannuronic acid reactive intermediate will be structurally close to ^3^ *H*_4_ oxocarbenium ion **6**. The minor α-products in these condensations likely arise from a higher energy ^4^ *H*_3_ oxocarbenium ion **19**, through a transition state that benefits from a developing anomeric effect and favorable conformational properties.

## Conclusions

The influence of structural changes in a glycosyl donor on the outcome of a glycosylation reaction, in terms of yield and stereoselectivity, has received considerable attention over the years and many ingenious donor systems have been developed for the stereoselective construction of glycosidic bonds. The influence of the reactivity of the acceptor in glycosylation reactions, on the other hand, is less well understood. Here we have investigated in a systematic manner how the outcome of a glycosylation system can change depending on the gradually changing reactivity of the nucleophile. We have shown that a series of partially fluorinated alcohols of gradually decreasing nucleophilicity, can be used to map how the stereoselectivity of a glycosylation system varies with changing acceptor reactivity. The simple “toolset” of partially fluorinated ethanols represents a rapid and easy means to dissect S_N_2-type (for ethanol) and S_N_1-type (for trifluoroethanol and hexafluoro-*iso*-propanol) glycosylation reaction mechanisms.^80^ It is expected that application of this set of model nucleophiles to newly developed glycosylation methodology or re-investigation of already established methods will bring detailed insight into the complex and intriguing glycosylation reaction mechanism. This will allow for more directed optimization of glycosylation reactions, taking away the trial and error component and ill-understood reaction protocols that have plagued carbohydrate chemistry for so long.
