# Supplementary material for: The influence of acceptor nucleophilicity on the glycosylation reaction mechanism
Source: Chem Sci. 2016 Nov 9;8(3):1867–75. doi: 10.1039/c6sc04638j (PMC5424809; doi:10.1039/c6sc04638j)
Supplement: Supplementary file 1 [file SC-008-C6SC04638J-s001.pdf]

Electronic Supplementary Material (ESI) for Chemical Science.
This journal is © The Royal Society of Chemistry 2016

The Influence of Acceptor Nucleophilicity on the Glycosylation Reaction Mechanism

S. van der Vorm, T. Hansen, H. S. Overkleeft, G. A. van der Marel, J. D. C. Codée

Leiden Institute of Chemistry, Leiden University, Einsteinweg 55, 2333 CC Leiden, The Netherlands

Supplementary Information

General experimental procedures S1

General procedure for Tf_2O/Ph_2SO mediated glycosylations S1

General computational procedure S1

Preparation of donor **1** S2

Preparation of donor **2** S3

Preparation of donor **3** S5

Preparation of acceptor **10** S7

Preparation of acceptor **11** S8

Preparation of acceptor **12** S9

Preparation of acceptor **13** S11

Preparation of acceptor **14** S13

Glycosylation data for donor **1** S14

Glycosylation data for donor **2** S21

Glycosylation data for donor **3** S28

NMR spectra of new compounds S36

Methyl 2,3,4-tri-*O*-methyl mannopyranosyl uronate oxocarbenium ion atom coordinates and energies of the $^3H_4$, $^4H_3$, $B_{2,5}$ conformations S110

General experimental procedures: All chemicals (Acros, Aldrich, Alfa Aesar, Fluka, Merck) were of commercial grade and used as received unless stated otherwise. DCM was stored over activated 4 Å molecular sieves (beads, 8-12 mesh, Sigma-Aldrich) for at least 24 h before use. Tri-*tert*-butylpyrimidine (TTBP) was synthesized as described by Crich *et al.*[1] All non-glycoside acceptors were stored in stock solutions (DCM, 0.5 M) over activated 4 Å molecular sieves. Glycoside acceptors were diluted to 0.5 M in DCM prior to use. Trifluoromethanesulfonic anhydride (Tf_2O) was distilled over P_2O_5 and stored at -20°C under an argon atmosphere. Triethylamine (Et_3N) was distilled over CaH_2 and stored over KOH pellets. Overnight temperature control was achieved by a FT902 Immersion Cooler (Julabo). Flash column chromatography was performed on silica gel 60 Å (0.04 – 0.063 mm, Screening Devices B.V.). Size exclusion chromatography was performed on Sephadex™ (LH-20, GE Healthcare Life Sciences) by isocratic elution with DCM/MeOH (1/1, v/v). TLC-analysis was conducted on TLC Silica gel 60 (Kieselgel 60 F_{254}, Merck) with UV detection by (254 nm) and by spraying with 20% sulfuric acid in ethanol followed by charring at ±150 °C or by spraying with a solution of $(NH_4)_6Mo_7O_{24}\cdot H_2O$ (25 g/L) and $(NH_4)_4Ce(SO_4)_4\cdot 2H_2O$ (10 g/L) in 10% sulfuric acid in water followed by charring at ±150 °C. TLC-MS analysis was performed on a Camag TLC-MS Interface combined with an API165 (SCIEX) mass spectrometer (eluted with *tert*-butylmethylether/EtOAc/MeOH, 5/4/1, v/v/v + 0.1% formic acid, flow rate 0.12 mL/min). LC-MS analysis was conducted on a Finnigan LCQ Advantage Max mass spectrometer with a Finnigan Surveyor HPLC system eluted with a gradient solvent (8 min, 1 mL/min, 10%-90% CH_3CN in H_2O + 1% TFA, total sample run 12 min). High-resolution mass spectra were recorded on a Thermo Finnigan LTQ Orbitrap mass spectrometer equipped with an electrospray ion source in positive mode (source voltage 3.5 kV, sheath gas flow 10, capillary temperature 275 ^{0}C) with resolution R=60.000 at m/z=400 (mass range = 150-4000). ^1H, ^2H and ^{13}C NMR spectra were recorded on a Bruker AV-400 NMR instrument (400, 61 and 101 MHz respectively), or a Bruker DMX-400 NMR instrument. For samples measured in $CDCl_3$ chemical shifts (δ) are given in ppm relative to tetramethylsilane as internal standard or the residual signal of the deuterated solvent. Coupling constants (J) are given in Hz. All given ^{13}C-APT spectra are proton decoupled. NMR peak assignments were made using COSY and HSQC. If necessary additional NOESY, HMBC and (HMBC-)GATED experiments were used to further elucidate the structure. The anomeric product ratios were based on careful analysis of the crude reaction mixture and the purified reaction product by integration of representative ^1H NMR signals. IR spectra were recorded on a Shimadzu FTIR-8300 IR spectrometer and are reported in cm^{-1}. Specific rotations were measured on a Propol automatic polarimeter in $CHCl_3$ (10 mg/ml) at 589 nm unless otherwise stated.

General procedure for Tf_2O/Ph_2SO mediated glycosylations: Donor (0.1 mmol), Ph_2SO (26 mg, 0.13 mmol, 1.3 eq.) and TTBP (62 mg, 0.25 mmol, 2.5 eq.) were coevaporated twice with dry toluene (4 Å molecular sieves) and dissolved in DCM (2 mL, 0.05 M donor). Activated 3Å molecular sieves (rods, size 1/16 in.) were added and the reaction mixture stirred for 30 min at room temperature. The solution was cooled to -78°C and Tf_2O (22 µl, 0.13 mmol, 1.3 eq.) was slowly added. The reaction mixture was allowed to warm to -60°C in approximately 45 min, followed by recooling to -78°C and addition of the acceptor (0.2 mmol, 2 eq.) in DCM (0.4 mL, 0.5 M). The reaction mixture was allowed to warm to -40°C in approximately 60 min and stirred for an additional 0-18 h depending on the acceptor. The reaction was quenched with Et_3N (0.1 mL, 0.72 mmol, 5.5 eq.) at -40 ^{0}C and diluted with DCM. The solution was transferred to a separatory funnel and water was added, the layers were separated and the water phase extracted once more with DCM. The combined organic layers were dried over $MgSO_4$, filtered, and concentrated *in vacuo*. Purification by silica gel flash column chromatography and when needed, sephadex™ LH-20 size exclusion chromatography yielded the glycosylation product as a mixture of anomers.

General computational procedure: Density functional theory (DFT) *ab initio* calculations were performed with the B3LYP model. Conformations were generated from a conformer distribution search option included in the Spartan 04 program[2] in the gas phase at the 6-31G* basis set level. All generated geometries were further optimized with Gaussian 03[3] at the 6-311G** level, their zero-point energy corrections calculated and further optimized with incorporated polarizable continuum model (PCM) to correct for solvation in dichloromethane.

Preparation of donor 1

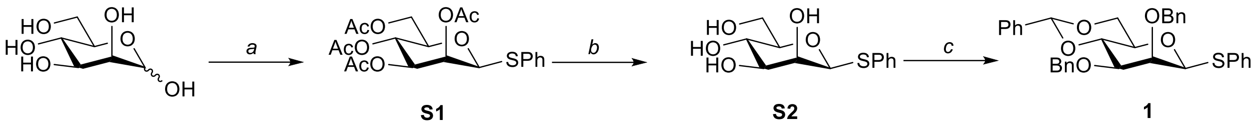

Scheme S-1: Mannose donor **1** synthesis. *Reagents and conditions:* a) *i.* Ac_2O, $HClO_4$; *ii.* HBr, AcOH; *iii.* PhSH, NaH, DMF, **S1**: 65% (three steps); b) NaOMe, MeOH, **S2**: 99%; c) *i.* $PhCH(OMe)_2$, CSA; *ii.* BnBr, NaH, DMF, **1**: 51% (two steps).

Phenyl 2,3,4,6-tetra-*O*-acetyl-1-thio-β-D-mannopyranoside (S1)

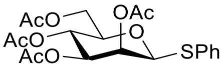

To a mixture of HBr (33 wt% in AcOH, 35 mL, 200 mmol, 1 eq.) and Ac_2O (93 mL, 1020 mmol, 5.1 eq.) and 10 drops of 70% aq. $HClO_4$, D-mannose (36.0 g, 200 mmol) was added portion wise at 0 ^{0}C. After 20 minutes an additional amount of HBr (33 wt% in AcOH, 70 mL, 400 mmol, 2 eq.) was added. After stirring for 16 h at r.t. the reaction mixture was concentrated *in vacuo* at 30 ^{0}C. The resulting black oil was co-evaporated with toluene until neutral pH was reached and was used in the following step without further purification. To a solution of the crude product in DMF (400 mL), thiophenol (21.5 mL, 210 mmol, 1.05 eq.) was added. The reaction mixture was cooled to 0 ^{0}C and NaH (60% dispersion in mineral oil, 8.4 g, 210 mmol, 1.05 eq.) was added portion wise. After 2 h stirring at r.t. the reaction was quenched by the addition of aq. HCl (1 M). To the resulting black suspension, 4 L of water was added and extracted 10 times with Et_2O. The combined organic layers were washed with water, dried with $MgSO_4$ and concentrated *in vacuo*. Flash column chromatography (9/1 to 7/3 pentane/EtOAc) afforded the title compound as an orange oil (57.3 g, 130 mmol, 65%). R_f: 0.70 (1/1 pentane/EtOAc). Spectroscopic data were in accord with those previously reported.[4,5] $[\alpha]_D^{26}$ = -44.4° (*c* = 0.5, $CHCl_3$); IR (neat): 1047, 1213, 1368, 1742; ^{1}H NMR (400 MHz, $CDCl_3$, HH-COSY, HSQC): δ 7.56 – 7.47 (m, 2H, CH_{arom} SPh), 7.34 – 7.30 (m, 3H, CH_{arom} SPh), 5.66 (dd, 1H, *J* = 3.5, 0.8 Hz, H-2), 5.28 (t, 1H, *J* = 10.0 Hz, H-4), 5.07 (dd, 1H, *J* = 10.1, 3.5 Hz, H-3), 4.94 (d, 1H, *J* = 1.0 Hz, H-1), 4.29 (dd, 1H, *J* = 12.2, 6.5 Hz, H-6), 4.17 (dd, 1H, *J* = 12.2, 2.4 Hz, H-6), 3.72 (ddd, 1H, *J* = 10.0, 6.4, 2.5 Hz, H-5), 2.20 (s, 3H, CH_3 OAc), 2.09 (s, 3H, CH_3 OAc), 2.04 (s, 3H, CH_3 OAc), 1.98 (s, 3H CH_3, OAc); ^{13}C-APT NMR (101 MHz, $CDCl_3$, HSQC): δ 170.5, 170.1, 167.0, 169.6 (C=O Ac), 133.2 (C_q SPh), 131.9, 129.1, 128.1 (CH_{arom} SPh), 85.5 (C-1), 76.4 (C-5), 71.8 (C-3), 70.6 (C-2), 65.8 (C-4), 62.8 (C-6), 20.7, 20.7, 20.6, 20.6 (CH_3 Ac); ^{13}C-GATED NMR (101 MHz, $CDCl_3$): δ 85.5 ($J_{C1,H1}$ = 153 Hz, C-1 β); HRMS: $[M+Na]^+$ calcd for $C_{20}H_{24}O_9SNa$ 463.10332, found 463.10305.

Phenyl 1-thio-β-D-mannopyranoside (S2)

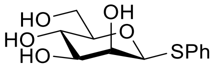

To a solution of **S1** (24.9 g, 56.5 mmol) in MeOH (280 mL), NaOMe (0.31 g, 5.7 mmol, 0.1 eq.) was added. The reaction mixture was stirred for 16 h. Amberlite IR120 H^+ was added until pH 6 was reached and the mixture was filtered and concentrated *in vacuo*. This afforded the title compound (15.3 g, 56.4 mmol, 99%) as a white foam. R_f: 0.20 (9/1 DCM/MeOH). Spectroscopic data were in accord with those previously reported.[6,7] IR (neat, cm^{-1}): 880, 1085, 1636, 2974, 3312; ^{1}H NMR (400 MHz, MeOD, HH-COSY, HSQC): δ 7.53 – 7.46 (m, 2H, CH_{arom} SPh), 7.34 – 7.17 (m, 3H, CH_{arom} SPh), 5.00 (s, 1H, H-1), 4.05 (dd, 1H, *J* = 3.4, 1.0 Hz, H-2), 3.88 (dd, 1H, *J* = 11.9, 2.4 Hz, H-6), 3.73 (dd, 1H, *J* = 12.1, 5.7 Hz, H-6), 3.63 (t, 1H, *J* = 9.5 Hz, H-4), 3.51 (dd, 1H, *J* = 9.5, 3.4 Hz, H-3), 3.29 (m, 1H, *J* = 5.8 Hz, H-5); ^{13}C-APT NMR (101 MHz, MeOD, HSQC):

δ 131.0, 130.0, 127.7 (CH_{arom} SPh), 88.8 (C-1), 82.4 (C-5), 76.2 (C-3), 74.3 (C-2), 68.3 (C-4), 62.9 (C-6); HRMS: $[M+Na]^+$ calcd for $C_{12}H_{16}O_5SNa$ 295.06107, found 295.06107.

Phenyl 2,3-di-*O*-benzyl-4,6-*O*-benzylidene-1-thio-β-D-mannopyranoside (1)

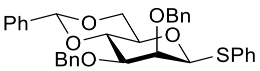

To a solution of **S2** (1.36 g, 5 mmol) in DMF (50 mL), benzaldehyde dimethyl acetal (3.0 mL, 20 mmol, 4 eq.) and CSA (0.25 g, 1 mmol, 0.2 eq.) were added. After the solution was stirred for 16 h, benzyl bromide (2.4 mL, 20 mmol, 4 eq.) and NaH (60% dispersion in mineral oil, 0.48 g, 20 mmol, 4 eq.) were added at 0 ^{0}C. The suspension was allowed to warm up until r.t. and stirred for an additional 2 h. The reaction mixture was quenched with MeOH, followed by the addition of DCM (250 mL) and ice water (500 mL). The water layer was extracted once with DCM and the combined organic layers were washed with water and brine. The combined organic layers were dried over $MgSO_4$, filtered and concentrated under reduced pressure. Flash column chromatography (1/0 to 9/1 pentane/EtOAc) and subsequent recrystallization from EtOAc and pentane afforded the title compound as a white solid (1.38 g, 2.56 mmol, 51% over 2 steps). R_f: 0.27 (9/1 pentane/EtOAc). Spectroscopic data were in accord with those previously reported.[8] IR (neat): 733, 1026, 1069, 1452, 2864; ^{1}H NMR (400 MHz, $CDCl_3$, HH-COSY, HSQC, HMBC): δ 7.64 – 7.14 (m, 20H, CH_{arom}), 5.64 (s, 1H, C*H*Ph), 5.12 (d, 1H, *J* = 11.1 Hz, C*H*H Bn), 4.89 (d, 1H, *J* = 12.3 Hz, CH*H* Bn), 4.86 (d, 1H, *J* = 11.1 Hz, C*H*H Bn), 4.85 (d, 1H, *J* = 1.3 Hz, H-1), 4.74 (d, 1H, *J* = 12.3 Hz, CH*H* Bn), 4.36 – 4.26 (m, 2H, H-4, H-6), 4.18 (dd, 1H, *J* = 3.1, 1.3 Hz, H-2), 3.95 (t, 1H, *J* = 10.3 Hz, H-6), 3.74 (dd, 1H, *J* = 9.8, 3.1 Hz, H-3), 3.42 (td, 1H, *J* = 9.7, 4.9 Hz, H-5); ^{13}C-APT NMR (101 MHz, $CDCl_3$, HSQC, HMBC): δ 138.1, 137.6, 135.1 (C_q), 131.2, 129.1, 129.1, 128.8, 128.6, 128.4, 127.9, 127.9, 127.8, 127.6, 126.2 (CH_{arom}), 101.6 (CHPh), 89.2 (C-1), 79.9 (C-3), 79.1 (C-2), 78.8 (C-4), 76.0, 73.3 (CH_2 Bn), 71.8 (C-5), 68.6 (C-6); ^{13}C-GATED NMR (101 MHz, $CDCl_3$) δ 89.2 ($J_{C1,H1}$ = 152 Hz, C-1 β); HRMS: $[M+NH_4]^+$ calcd for $C_{33}H_{36}NO_5S$ 558.23087, found 558.23071.

Preparation of donor 2

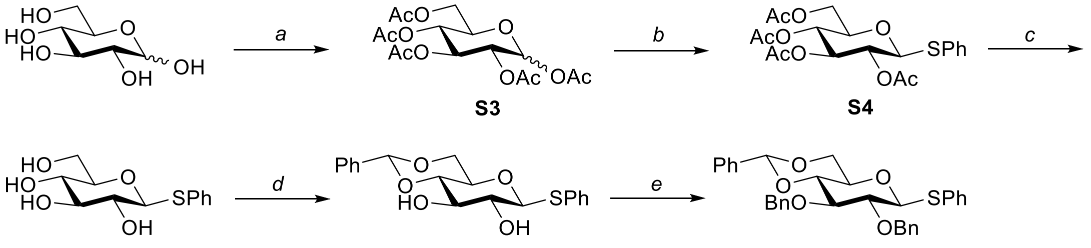

Scheme S-2: Glucose donor **2** synthesis. *Reagents and conditions:* a) Ac_2O, NaOAc, **S3**: 89%; b) PhSH, BF_3•OEt_2, DCM, **S4**: 75%; c) NaOMe, MeOH, **S5**: 85%; d) $PhCH(OMe)_2$, *p*-TsOH•H_2O, **S6**: 94%; e) BnBr, NaH, DMF, **2**: 86%.

1,2,3,4,6-Penta-*O*-acetyl-α/β-D-glucopyranoside (S3)

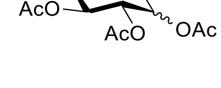

To a 140°C solution of NaOAc (8.2 g, 100 mmol, 0.5 eq) in Ac_2O (190 mL, 2 mol, 10 eq.) D-glucose was added portionwise and the reaction mixture was refluxed for an additional 15 min. The solution was cooled to r.t. and poured over crushed ice. The product was filtered, taken up in DCM, concentrated *in vacuo* and recrystallized from hot EtOH (750 mL) to give the product as a white solid (69.4 g, 178 mmol, 89%). Spectroscopic data were in accord with those previously reported.[9,10] Data for the β-anomer: ^{1}H NMR ($CDCl_3$, 400 MHz, HH-COSY, HSQC): δ 5.72 (d, 1H, *J* = 8.3 Hz, H-1), 5.26 (t, 1H, *J* = 9.4 Hz, H-3), 5.19 – 5.08 (m, 2H, H-2, H-4), 4.30 (dd, 1H, *J* = 12.7, 3.8 Hz, H-6), 4.15 – 4.08 (m, 1H, H-6), 3.88 – 3.81 (m, 1H, H-5), 2.12 (s, 3H, CH_3 OAc), 2.09 (s, 3H, CH_3 OAc), 2.04 (s, 6H, 2xCH_3 OAc), 2.02 (s, 3H, CH_3 OAc); ^{13}C-APT NMR ($CDCl_3$, 101 MHz, HSQC): δ 170.7, 170.2, 169.5, 169.4, 169.1 (C_q OAc), 91.8 (C-1), 72.9, 72.8 (C-3, C-5), 70.3, 67.9 (C-2, C-4), 61.6 (C-6), 21.0, 20.8, 20.7 (CH_3 OAc);

Phenyl 2,3,4,6-tetra-*O*-acetyl-1-thio-β-D-glucopyranoside (S4)

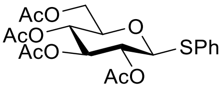

D-glucose pentaacetate **S3** (20 g, 51 mmol) was dissolved in DCM (100 mL) and cooled to 0°C. Thiophenol (7.8 mL, 76.5 mmol, 1.5 eq.) was added followed by addition of boron trifluoride diethyl etherate (10.9 mL, 76.5 mmol, 1.5 eq.) and the mixture was refluxed overnight. Sat. aq. $NaHCO_3$ (300 mL) and Et_2O (100 mL) were added and the mixture was extracted three times with Et_2O. The organic layer was washed with brine, dried with $MgSO_4$, filtered and concentrated under reduced pressure. The crude product was purified by crystallization from EtOAc/hexane (1/10) to obtain the title compound as a white solid. (16.8 g, 38.2 mmol, 75%). Spectroscopic data were in accord with those previously reported.[11–14] ^{1}H NMR ($CDCl_3$, 400 MHz, HH-COSY, HSQC): δ 7.50 (dd, 2H, *J* = 6.6, 3.0 Hz, CH_{arom}), 7.34 – 7.28 (m, 3H, CH_{arom}), 5.24 (t, 1H, *J* = 9.3 Hz, H-3), 5.04 (t, 1H, *J* = 9.8 Hz, H-4), 4.97 (t, 1H, *J* = 9.6 Hz, H-2), 4.75 (d, 1H, *J* = 10.0 Hz, H-1), 4.23 (dd, 1H, *J* = 12.3, 5.1 Hz, H-6), 4.17 (dd, 1H, *J* = 12.3, 2.5 Hz, H-6), 3.76 (ddd, 1H, *J* = 10.0, 5.1, 2.5 Hz, H-5), 2.07 (s, 3H, CH_3 OAc), 2.06 (s, 3H, CH_3 OAc), 2.01 (s, 3H, CH_3 OAc), 1.98 (s, 3H, CH_3 OAc); ^{13}C-APT NMR ($CDCl_3$, 101 MHz, HSQC): δ 170.2, 169.8, 169.2, 169.0 (C=O Ac), 132.8 (CH_{arom}), 131.5 (C_q), 128.8, 128.2 (CH_{arom}), 85.3 (C-1), 75.5 (C-5), 73.8 (C-3), 69.8 (C-2), 68.1 (C-4), 61.9 (C-6), 20.5, 20.5, 20.4, 20.4 (CH_3 Ac);

Phenyl 1-thio-β-D-glucopyranoside (S5)

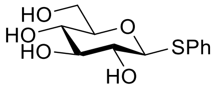

To a solution of **S4** (16.3 g, 37.0 mmol) in MeOH (200 mL) was added Na(s) (89 mg, 3.7 mmol, 0.1 eq) and the reaction was stirred for 18 h at r.t. The reaction mixture was neutralized with Amberlite H^+, filtered and Celite® was added to the filtrate and the mixture concentrated *in vacuo*. The residue was purified by flash column chromatography (1% to 12% EtOH in EtOAc) to obtain a white solid (8.6 g, 31.6 mmol, 85%). Spectroscopic data were in accord with those previously reported.[15] ^{1}H NMR (MeOD, 400 MHz, HH-COSY, HSQC): δ 7.61 – 7.54 (m, 2H, CH_{arom}), 7.33 – 7.24 (m, 3H, CH_{arom}), 4.60 (d, 1H, *J* = 9.8 Hz, H-1), 3.87 (dd, 1H, *J* = 12.1, 1.8 Hz, H-6), 3.67 (dd, 1H, *J* = 12.2, 5.2 Hz, H-6), 3.39 (t, 1H, *J* = 8.5 Hz, H-3), 3.35 – 3.26 (m, 2H, H-4, H-5), 3.22 (dd, 1H, *J* = 9.8, 8.6 Hz, H-2); ^{13}C-APT NMR (MeOD, 101 MHz, HSQC): δ 135.3 (C_q), 132.7, 129.9, 128.3 (CH_{arom}), 89.4 (C-1), 82.0 (C-4), 79.7 (C-3), 73.7 (C-2), 71.3 (C-5), 62.8 (C-6);

Phenyl 4,6-*O*-benzylidene-1-thio-β-D-glucopyranoside (S6)

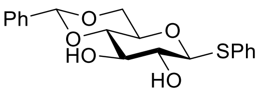

To a solution of **S5** (12.81 g, 47 mmol) and *p*-TsOH•H_2O (100 mg, 0.5 mmol, 0.01 eq.) in DMF (25 mL) and CH_3CN (100 mL) was added benzyldehyde dimethyl acetal (9.9 mL, 65.8 mmol, 1.4 eq.). The reaction was heated to 50°C at 250 mbar for 5 hours and subsequently quenched with Et_3N (2 mL) and diluted with EtOAc (250 mL). The solution was washed with H_2O (2x 100 mL) and brine (100 mL). The organic layer was dried ($MgSO_4$) and concentrated *in vacuo*. Percipitation from EtOAc/petroleum ether formed a waxy material (12.0 g, 33.3 mmol) and the remaining mother liquors were purified by column chromatography (3/1 to 1/3 pentane/EtOAc) to give another batch of product (3.86 g, 10.7 mmol). Total yield 15.9 g, 44 mmol, 94%. R_f: 0.50 (1/2 pentane/EtOAc). Spectroscopic data were in accord with those previously reported.[12–14] ^1H NMR ($CDCl_3$, 400 MHz, HH-COSY, HSQC): δ 7.59 – 7.51 (m, 2H, CH_{arom}), 7.51 – 7.44 (m, 2H, CH_{arom}), 7.40 – 7.31 (m, 6H, CH_{arom}), 5.54 (s, 1H, C*H*Ph), 4.64 (d, 1H, *J* = 9.8 Hz, H-1), 4.39 (dd, 1H, *J* = 10.5, 4.4 Hz, H-6), 3.91 – 3.74 (m, 2H, H-4, H-6), 3.59 – 3.43 (m, 3H, H-2, H-3, H-5); ^{13}C-APT NMR ($CDCl_3$, 101 MHz, HSQC): δ 137.0 (C_q), 133.2 (CH_{arom}), 131.4 (C_q), 129.5, 129.3, 128.7, 128.5, 126.4 (CH_{arom}), 102.1 (CHPh), 88.8 (C-1), 80.4 (C-3), 74.7 (C-4), 72.7 (C-2), 70.7 (C-5), 68.7 (C-6);

Phenyl 2,3-di-*O*-benzyl-4,6-*O*-benzylidene-1-thio-β-D-glucopyranoside (2)

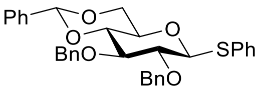

Diol **S6** (7.21 g, 20 mmol) was dissolved in DMF (100 mL) and cooled to 0°C. Benzyl bromide (5.75 mL, 48 mmol, 2.4 eq.) and NaH (60% dispersion in mineral oil, 2.4 g, 60 mmol, 3 eq.) were added and the reaction mixture was allowed to stir overnight. MeOH was added to quench the reaction followed by H_2O (500 mL) and EtOAc (300 mL). The organic layer was washed with brine and dried with MgSO4. After concentration of the organic layer under reduced pressure, the crude product was crystallized from EtOAc (50 mL) and hexane (100 mL) to obtain the title compound as a white solid (9.49 g, 17.4 mmol, 86%). Spectroscopic data were in accord with those previously reported.[12–14,16] ^1H NMR ($CDCl_3$, 400 MHz, HH-COSY, HSQC): δ 7.56 – 7.51 (m, 2H, CH_{arom}), 7.51 – 7.46 (m, 2H, CH_{arom}), 7.42 – 7.26 (m, 16H, CH_{arom}), 5.59 (s, 1H, C*H*Ph), 4.94 (d, 1H, *J* = 11.1 Hz, C*H*H Bn), 4.86 (d, 1H, *J* = 10.2 Hz, C*H*H Bn), 4.81 (d, 1H, *J* = 10.3 Hz, CH*H* Bn), 4.80 – 4.73 (m, 2H, CH*H* Bn, H-1), 4.39 (dd, 1H, *J* = 10.5, 5.0 Hz, H-6), 3.84 (dd, 1H, *J* = 9.3, 8.3 Hz, H-3), 3.80 (t, 1H, *J* = 10.3 Hz, H-6), 3.71 (t, 1H, *J* = 9.3 Hz, H-4), 3.56 – 3.42 (m, 2H, H-2, H-5); ^{13}C-APT NMR ($CDCl_3$, 101 MHz, HSQC): δ 138.4, 138.1, 137.4, 133.2 ($C_{q\text{-}arom}$), 132.5, 129.1, 129.1, 128.5, 128.5, 128.4, 128.3, 128.2, 128.0, 128.0, 127.9, 126.1 (CH_{arom}), 101.3 (CHPh), 88.4 (C-1), 83.1 (C-3), 81.6 (C-4), 80.6 (C-2), 76.0, 75.5 (CH_2 Bn), 70.4 (C-5), 68.8 (C-6); HRMS: $[M+H]^+$ calcd for $C_{33}H_{33}O_5S$ 541.20432, found 541.20392.

Preparation of donor 3

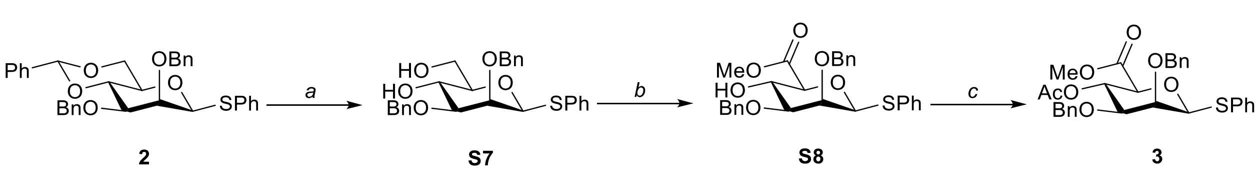

Scheme S-3: Mannuronic acid ester donor **3** synthesis. *Reagents and conditions:* a) *p*-TsOH•H_2O, MeOH, **S7**: 93%; b) *i*. TEMPO, BAIB, DCM, H_2O, AcOH; *ii*. MeI, K_2CO_3, DMF, **S8**: 63% (two steps); c) Ac_2O, pyridine, **3**: 92%.

Phenyl 2,3-di-*O*-benzyl-1-thio-β-D-mannopyranoside (S7)

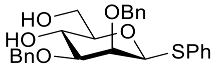

To a solution of **2** (1.62 g, 3 mmol) in MeOH (30 mL), *p*-TsOH•H_2O (60 mg, 0.3 mmol, 0.1 eq.) was added. The suspension was stirred for 1 h at 50 ^{0}C and subsequently quenched with Et_3N. After concentration *in vacuo* the resulting product was purified by flash column chromatography (9/1 to 1/1 pentane/EtOAc) to yield the title compound as a colourless foam (1.26 g, 2.78 mmol, 93%). R_f: 0.10 (4/1 pentane/EtOAc). Spectroscopic data were in accord with those previously reported.[17] $[\alpha]_D^{26}$ = -62.4° (*c* = 0.5, $CHCl_3$); IR (neat): 734, 1026, 1119, 1454, 924, 3391; ^{1}H NMR (400 MHz, $CDCl_3$, HH-COSY, HSQC): δ 7.54 – 7.19 (m, 15H, CH_{arom}), 4.97 (d, 1H, *J* = 11.3 Hz, C*H*H Bn), 4.85 (s, 1H, H-1), 4.85 (d, 1H, *J* = 11.3 Hz, CH*H* Bn), 4.75 (d, 1H, *J* = 11.7 Hz, C*H*H Bn), 4.53 (d, 1H, *J* = 11.7 Hz, CH*H* Bn), 4.20 (d, 1H, *J* = 2.1 Hz, H-2), 4.05 (td, 1H, *J* = 9.5, 2.3 Hz, H-4), 3.93 (ddd, 1H, *J* = 11.0, 7.2, 3.6 Hz, H-6), 3.82 (dt, 1H, *J* = 12.1, 6.3 Hz, H-6), 3.46 (dd, 1H, *J* = 9.5, 2.8 Hz, H-3), 3.38 (ddd, 1H, *J* = 9.5, 6.0, 3.6 Hz, H-5), 2.33 (s, 1H, 4-OH), 2.14 (t, 1H, *J* = 6.4 Hz, 6-OH); ^{13}C-APT NMR (101 MHz, $CDCl_3$, HSQC): δ 138.0, 137.6, 135.1 (C_q), 130.7, 129.2, 128.8, 128.5, 128.4, 128.3, 127.9, 127.9, 127.5 (CH_{arom}), 87.9 (C-1), 83.6 (C-3), 80.1 (C-5), 76.7 (C-2), 75.3, 72.3 (CH_2 Bn), 67.5 (C-4), 63.1 (C-6); ^{13}C-GATED NMR (101 MHz, $CDCl_3$): δ 87.9 (*J* = 152 Hz, C-1 β); HRMS: $[M+Na]^+$ calcd for $C_{26}H_{28}O_5SNa$ 475.15497, found 475.15430.

Methyl (phenyl 2,3-di-*O*-benzyl-1-thio-β-D-mannopyranosyl uronate) (S8)

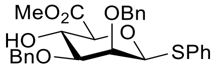

To a two phase system of **S7** (1.25 g, 2.76 mmol) in DCM (10 mL) and H_2O (5 mL), TEMPO (86 mg, 0.55 mmol, 0.2 eq.), BAIB (2.22 g, 6.9 mmol, 2.5 eq.) and AcOH (50 µL) were added. The reaction mixture was stirred for 6 h and was quenched with sat. aq. $Na_2S_2O_3$. The resulting suspension was concentrated under reduced pressure and co-evaporated three times with toluene. The formed solid was dissolved in DMF (15 mL), K_2CO_3 (1.14 g, 8.28 mmol, 3 eq.) and methyl iodide (0.52 mL, 8.28 mmol, 3 eq.) were added. The suspension was stirred for 16 h at r.t. and followed by the addition of H_2O (150 mL). The aqueous layer was extracted three times with Et_2O and subsequently washed with sat. aq. $NaHCO_3$ and brine. The combined organic layers were dried over $MgSO_4$, filtered and concentrated under reduced pressure. Flash column chromatography (1/0 to 3/1 pentane/EtOAc) followed by recrystallization in EtOAc and pentane afforded the title compound as a white solid (0.48 g, 1.75 mmol, 63% over 2 steps). R_f: 0.70 (1/1 pentane/EtOAc). Spectroscopic data were in accord with those previously reported.[17] $[\alpha]_D^{26}$ = -72,0° (*c* = 0.5, $CHCl_3$); IR (neat): 696, 735, 1026, 1064, 1123, 1429, 1454, 1744, 2855, 2924, 3462; ^{1}H NMR (400 MHz, $CDCl_3$, HH-COSY, HSQC): δ 7.51 – 7.25 (m, 20H, CH_{arom}), 5.02 (d, 1H, *J* = 11.4 Hz, C*H*H Bn), 4.86 (d, 1H, *J* = 11.3 Hz, CH*H* Bn), 4.79 – 4.74 (m, 3H, C*H*H Bn, CH*H* Bn, H-1), 4.41 (td, 1H, *J* = 9.5, 2.1 Hz, H-4), 4.12 (dd, 1H, *J* = 3.0, 1.0 Hz, H-2), 3.84 – 3.77 (m, 4H, H-5, CH_3 CO_2Me), 3.50 (dd, 1H, *J* = 9.5, 2.9 Hz, H-3), 3.11 (d, 1H, *J* = 2.3 Hz, 4-OH); ^{13}C-APT NMR (101 MHz, $CDCl_3$, HSQC): δ 138.0, 135.1 (C_q), 131.3, 129.1, 128.7, 128.6, 128.3, 128.1, 127.9, 127.9, 127.6 (CH_{arom}), 89.0 (C-1), 82.4 (C-3), 78.3 (C-5), 77.0 (C-2), 75.4, 73.1 (CH_2 Bn), 68.6 (C-4), 52.9 (CH_3 CO_2Me); ^{13}C- GATED NMR (101 MHz, $CDCl_3$): δ 89.0 ($J_{C1,H1}$ = 154 Hz, C-1 β). HRMS: $[M+H]^+$ calcd for $C_{27}H_{29}O_6S$ 481,16794, found 481,16812.

Methyl (phenyl 4-*O*-acetyl-2,3-di-*O*-benzyl-thio-β-D-mannopyranosyl uronate) (3)

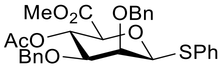

To a suspension of **S8** (1.20 g, 2.5 mmol) in pyridine (3.0 mL, 37.5 mmol, 15 eq.), Ac_2O (0.30 mL, 3.1 mmol, 1.25 eq.) was added. After stirring for 16 h at r.t., the reaction mixture was quenched with H_2O (25 mL). To the quenched reaction mixture, EtOAc was added and the layers were separated. The water layer was extracted for an additional 2 times with EtOAc. The combined organic layers were washed with sat. aq. $NaHCO_3$ and brine. The resulting organic layer was dried over $MgSO_4$, filtered and concentrated under reduced pressure. Flash column chromatography (9/1 to 7/3 pentane/EtOAc) afforded the title compound as a white solid (1.2 g, 2.3 mmol, 92%). R_f: 0.42 (7/3 pentane/EtOAc). $[\alpha]_D^{26}$ = -84.0° (*c* = 0.5, $CHCl_3$); IR (neat): 733, 1024, 1053, 1089, 1746, 2870; ^1H NMR (400 MHz, $CDCl_3$, HH-COSY, HSQC): δ 7.50 – 7.23 (m, 15H, CH_{arom}), 5.61 (t, 1H, *J* = 9.6 Hz, H-4), 5.03 (d, 1H, *J* = 11.6 Hz, C*H*H Bn), 4.85 (d, 1H, *J* = 11.6 Hz, CH*H* Bn), 4.78 (d, 1H, *J* = 1.1 Hz, H-1), 4.67 (d, 1H, *J* = 12.2 Hz, C*H*H Bn), 4.57 (d, 1H, *J* = 12.2 Hz, CH*H* Bn), 4.15 (dd, 1H, *J* = 2.8, 1.0 Hz, H-2), 3.88 (d, 1H, *J* = 9.6 Hz, H-5), 3.74 (s, 3H, CH_3 CO_2Me), 3.61 (dd, 1H, *J* = 9.7, 2.9 Hz, H-3), 2.01 (s, 3H, CH_3 OAc); ^{13}C-APT NMR (101 MHz, $CDCl_3$, HSQC): δ 169.7, 167.8 (C=O CO_2Me, Ac), 137.9, 137.7, 135.1 (C_q), 131.3, 129.1, 128.7, 128.6, 128.3, 128.1, 127.9, 127.7, 127.7 (CH_{arom}), 88.7 (C-1), 80.4 (C-3), 77.3 (C-5), 76.4 (C-2), 75.1, 72.6 (CH_2 Bn), 68.9 (C-4), 52.8 (CH_3 CO_2Me), 21.0 (CH_3 Ac); ^{13}C-GATED NMR (101 MHz, $CDCl_3$): δ 88.7 ($J_{C1,H1}$ = 152 Hz, C-1 β); HRMS: $[M+NH_4]^+$ calcd for $C_{29}H_{34}NO_7S$ 540.20505, found 540.20515.

Preparation of acceptor 10

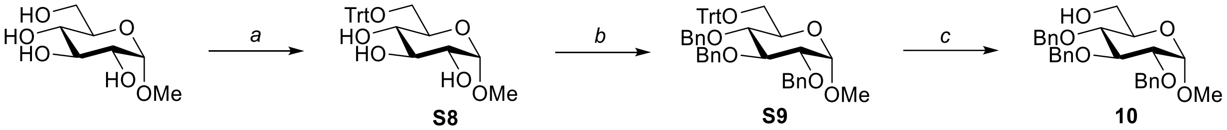

Scheme S-4: Glucose acceptor **10** synthesis. *Reagents and conditions:* a) TrtCl, Et_3N, DMAP, DMF; b) BnBr, NaH, DMF; c) *p*-TsOH•H_2O, MeOH, **10**: 78% (three steps).

Methyl 2,3,4-tri-*O*-benzyl-α-D-glucopyranoside (10)

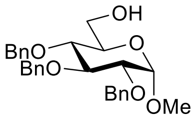

To a solution of methyl-α-D-glucopyranoside in DMF (50 mL), trityl chloride (3.1 g, 11 mmol, 1.1 eq.), Et_3N (2.1 mL, 15 mmol, 1.5 eq.) and DMAP (0.12 g, 1 mmol, 0.1 eq.) were added. After stirring for 6 h at 60 ^{0}C the reaction was cooled to 0 ^{0}C and followed by the addition of benzyl bromide (4.8 mL, 40 mmol, 4 eq.), NaH (2 g, 50 mmol, 5 eq.). The suspension was stirred for 16 h at r.t. and subsequently quenched with MeOH. The reaction mixture was concentrated under reduced pressure and the remaining oil was transferred to a separation funnel. Et_2O and H_2O were added and the layers were separated. The water layer was extracted three more times with Et_2O. The combined organic layers were washed with water, sat. aq. $NaHCO_3$ and brine. The resulting organic layer was dried over $MgSO_4$, filtered and concentrated under reduced pressure. The crude product **S9** was suspended in MeOH (100 mL) followed by the addition of *p*-TsOH·H_2O (0.19 g, 1 mmol, 0.1 eq.). After stirring for 1 h at 50 ^{0}C the reaction mixture was quenched with sat. aq. $NaHCO_3$ and concentrated *in vacuo*. Flash column chromatography (1/0 to 7/3 pentane/EtOAc) afforded the title compound as a waxy solid (3.6 g, 7.7 mmol, 78% over 3 steps). Spectroscopic data were in accord with those previously reported.[11,18] R_f: 0.57 (7/3 pentane/EtOAc). IR (neat): 880, 1043, 1086, 1381, 1636, 2893, 2974, 3312; ^1H NMR (400 MHz, $CDCl_3$, HH-COSY, HSQC): δ 7.42 – 7.24 (m, 15H, CH_{arom}), 4.99 (d, 1H, *J* = 10.8 Hz, C*H*H Bn), 4.92 – 4.77 (m, 3H, C*H*H Bn, CH*H* Bn, C*H*H Bn), 4.72 – 4.60 (m, 2H, CH*H* Bn, CH*H* Bn), 4.56 (d, 1H, *J* = 3.5 Hz, H-

1), 4.01 (t, 1H, J = 9.3 Hz, H-4), 3.82 – 3.73 (m, 1H, H-6), 3.73 – 3.61 (m, 2H, H-6, H-5), 3.58 – 3.45 (m, 2H, H-6, H-2, H-3), 3.37 (s, 3H, CH_3 OMe), 1.61 (dd, 1H, J = 7.3, 5.4 Hz, 6-OH); ^{13}C-APT NMR (101 MHz, $CDCl_3$, HSQC, HMBC): δ 138.8, 138.2 (C_q), 128.6, 128.6, 128.6, 128.3, 128.2, 128.1, 128.1, 128.0, 127.8 (CH_{arom}), 98.3 (C-1), 82.1 (C-4), 80.0 (C-2), 77.4 (C-3), 75.9, 75.2, 73.6 (CH_2 Bn), 70.7 (C-5), 62.0 (C-6), 55.3 (CH_3 OMe); ^{13}C-GATED NMR (101 MHz, $CDCl_3$): δ 98.3 ($J_{C1,H1}$ = 164 Hz, C-1 α); HRMS: $[M+Na]^+$ calcd for $C_{28}H_{32}O_6Na$ 487.20911, found 487.20851.

Preparation of acceptor 11

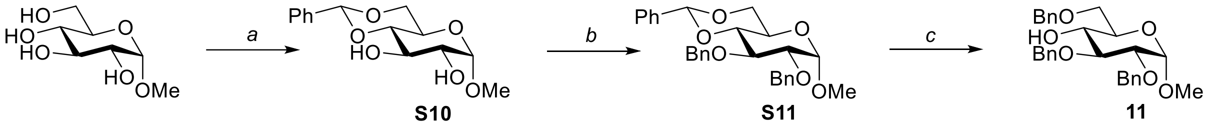

Scheme S-5: Glucose acceptor **11** synthesis. *Reagents and conditions:* a) $PhCH(OMe)_2$, *p*-TsOH•H_2O, **S10**: 87%; b) BnBr, NaH, DMF, **S11**: 87%; c) $NaCNBH_3$, THF, HCl, dioxane, **11**: 87%.

Methyl 4,6-*O*-benzylidene-α-D-glucopyranoside (S10)

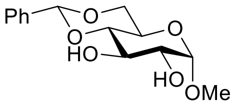

To a solution of methyl α-D-glucopyranoside (38.8 g, 200 mmol) in acetonitrile (800 mL) was added $PhCH(OMe)_2$ (36 mL, 240 mmol, 1.2 eq.) and *p*-TsOH•H_2O (3.8 g, 20 mmol, 0.1 eq.). The solution was stirred overnight at ambient temperature followed by concentration *in vacuo* (60°C, 600 mbar, 1.5 h) to a quarter of its original volume. The reaction mixture was treated with Et_3N (3 mL), diluted with EtOAc (500 mL) and subsequently washed with H_2O (2x 150 mL), sat. aq. $NaHCO_3$ (50 mL) and brine (2x 100 mL). The organic layer was dried ($MgSO_4$), filtered and concentrated in vacuo. The resulting crude residue was crystalized from EtOAc/petroleum ether to give the title product in two crops (49.2 g, 174 mmol, 87%, white solid). Spectroscopic data were in accord with those previously reported.[11,19] ^{1}H NMR ($CDCl_3$, 400 MHz, HH-COSY, HSQC): δ 7.51 – 7.45 (m, 2H, CH_{arom}), 7.36 (dd, 3H, J = 5.1, 2.0 Hz, CH_{arom}), 5.51 (s, 1H, C*H*Ph), 4.75 (d, 1H, J = 3.8 Hz, H-1), 4.28 (dd, 1H, J = 9.6, 4.3 Hz, H-6), 3.91 (t, 1H, J = 9.2 Hz, H-3), 3.84 – 3.68 (m, 2H, H-5, H-6), 3.60 (dd, 1H, J = 9.2, 3.9 Hz, H-2), 3.47 (t, 1H, J = 9.3 Hz, H-4), 3.43 (s, 3H, CH_3 OMe), 2.87 (bs, 2H, OH); ^{13}C-APT NMR ($CDCl_3$, 101 MHz, HSQC): δ 137.2 (C_q), 129.4, 128.4, 126.4 (CH_{arom}), 102.0 (CHPh), 99.9 (C-1), 81.1 (C-4), 72.9 (C-2), 71.7 (C-3), 69.0 (C-6), 62.5 (C-5), 55.7 (OMe);

Methyl 2,3-di-*O*-benzyl-4,6-*O*-benzylidene-α-D-glucopyranoside (S11)

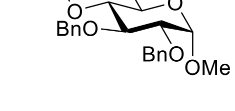

Benzyl bromide (10.5 mL, 88 mmol, 2.2 eq.) and sodium hydride (60% dispersion, 4.16 g, 104 mmol, 2.6 eq.) were added to a 0°C solution of diol **S10** (11.29 g, 40 mmol) in DMF (200 mL) and the solution was stirred overnight. The reaction mixture was quenched by slow addition of MeOH, diluted with EtOAc (500 mL) and washed with H_2O (200 mL) and brine (200 mL). The organic layer was dried with $MgSO_4$, filtered and concentrated *in vacuo*. The solid residue was recrystallization from EtOAc/pentane to yield the title compound as a white solid (16.0 g, 34.6 mmol, 87%). R_f: 0.57 (4/1 pentane/EtOAc).

Spectroscopic data were in accord with those previously reported.[11,16] ^{1}H NMR ($CDCl_3$, 400 MHz, HH-COSY, HSQC): δ 7.52 – 7.46 (m, 2H, CH_{arom}), 7.41 – 7.25 (m, 13H, CH_{arom}), 5.55 (s, 1H, C*H*Ph), 4.92 (d, 1H, *J* = 11.3 Hz, C*H*H Bn), 4.85 (d, 1H, *J* = 12.1 Hz, C*H*H Bn), 4.84 (d, 1H, *J* = 11.3 Hz, CH*H* Bn), 4.70 (d, 1H, *J* = 12.1 Hz, CH*H* Bn), 4.59 (d, 1H, *J* = 3.7 Hz, H-1), 4.26 (dd, 1H, *J* = 10.1, 4.7 Hz H-6), 4.05 (t, 1H, *J* = 9.3 Hz, H-3), 3.83 (td, 1H, *J* = 9.9, 4.7 Hz, H-5), 3.70 (t, 1H, *J* = 10.2 Hz, H-6), 3.60 (t, 1H, *J* = 9.4 Hz, H-4), 3.56 (dd, 1H, *J* = 9.3, 3.7 Hz, H-2), 3.40 (s, 3H, CH_3 OMe); ^{13}C-APT NMR ($CDCl_3$, 101 MHz, HSQC): δ 138.8, 138.3, 137.5 (C_q), 129.0, 128.6, 128.4, 128.3, 128.2, 128.1, 128.0, 127.7, 126.1 (CH_{arom}), 101.4 (CHPh), 99.3 (C-1), 82.2 (C-4), 79.3 (C-2), 78.7 (C-3), 75.5, 73.9 (CH_2 Bn), 69.2 (C-6), 62.4 (C-5), 55.5 (OMe);

Methyl 2,3,6-tri-*O*-benzyl-α-D-glucopyranoside (11)

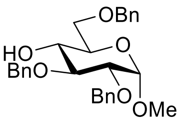

Fully protected compound **S11** (3.24 g, 7.0 mmol) was dissolved in THF (100 mL) and $NaCNBH_3$ (4.0 g, 63 mmol, 9 eq.) was added. To this solution 4.0 M HCl in 1,4-dioxane (18 mL, 72 mmol, 10.3 eq.) was slowly added and the reaction was stirred for an additional hour. Ice cold H_2O (300 mL) was added and the mixture extracted with DCM (2x 120 mL). The combined organic layers were washed with sat. aq. $NaHCO_3$ (100 mL) and brine (100 mL), dried with $MgSO_4$ and concentrated *in vacuo*. Flash column chromatography (6/1 to 4/1 pentane/EtOAc,) gave the title compound as a colorless oil (2.7 g, 5.85 mmol, 87%). R*f*: 0.37 (4/1 pentane/EtOAc). Spectroscopic data were in accord with those previously reported.[11] IR (neat): 695, 732, 1027, 1047, 1453, 2910, 3477; ^{1}H NMR ($CDCl_3$, 400 MHz, HH-COSY, HSQC): δ 7.41 – 7.23 (m, 15H, CH_{arom}), 4.99 (d, 1H, *J* = 11.4 Hz, C*H*H Bn), 4.75 (d, 1H, *J* = 12.1 Hz, C*H*H Bn), 4.73 (d, 1H, *J* = 11.4 Hz, CH*H* Bn), 4.68 – 4.60 (m, 2H, CH*H* Bn, H-1), 4.57 (d, 1H, *J* = 12.1 Hz, C*H*H Bn), 4.52 (d, 1H, *J* = 12.2 Hz, CH*H* Bn), 3.78 (dd, 1H, *J* = 9.6, 8.8 Hz, H-3), 3.74 – 3.63 (m, 3H, H-5, H-6, H-6), 3.59 (t, 1H, *J* = 9.2 Hz, H-4), 3.52 (dd, 1H, *J* = 9.6, 3.5 Hz, H-2), 3.37 (s, 3H, CH_3 OMe), 2.44 (bs, 1H, 4-OH); ^{13}C-APT NMR ($CDCl_3$, 101 MHz, HSQC): δ 138.8, 138.1, 138.0 (C_q), 128.6, 128.5, 128.4, 128.1, 128.0, 128.0, 127.8, 127.6, 127.6 (CH_{arom}), 98.2 (C-1), 81.5 (C-3), 79.6 (C-2), 75.4, 73.6, 73.1 (CH_2 Bn), 70.7 (C-4), 69.9 (C-5), 69.5 (C-6), 55.2 (OMe); HRMS: $[M+NH_4]^+$ calcd for $C_{28}H_{36}NO_6$ 482.25371, found 482.25357.

Preparation of acceptor 12

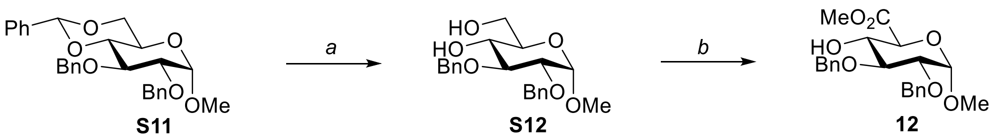

Scheme S-6: Glucuronic acid ester acceptor **12** synthesis. *Reagents and conditions:* a) *p*-TsOH•H_2O, MeOH, **S12**: 99%; b) *i*. TEMPO, BAIB, DCM, H_2O, AcOH; *ii*. MeI, K_2CO_3, DMF, **12**: 52% (two steps).

Methyl 2,3-di-*O*-benzyl-α-D-glucopyranoside (S12)

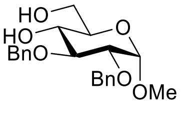

Fully protected **S11** (9.25 g, 20 mmol) and *p*-TsOH•H_2O (380 mg, 2 mmol, 0.1 eq.) were added to MeOH (100 mL) and heated at 60°C for 15 min after all solids were dissolved and TLC analysis showed full conversion to a lower running spot. The reaction mixture was quenched with Et_3N (1 mL) and concentrated *in vacuo*. The crude product was purified by flash column chromatography (8/1 to 3/2 pentane/acetone) to give the tile compound as a white solid (7.4 g, 19.8 mmol, 99%) as a white solid. R_f: 0.33 (2/1 pentane/acetone). Spectroscopic data were in accord with those previously reported.[11,20] ^{1}H NMR ($CDCl_3$, 400 MHz, HH-COSY, HSQC): δ 7.37 – 7.26 (m, 10H, CH_{arom}), 5.00 (d, 1H, *J* = 11.4 Hz, C*H*H Bn), 4.75 (d, 1H, *J* = 12.2 Hz, C*H*H Bn), 4.71 (d, 1H, *J* = 11.5 Hz, CH*H* Bn), 4.64 (d, 1H, *J* = 12.1 Hz, CH*H* Bn), 4.59 (d, 1H, *J* = 3.5 Hz, H-1), 3.78 (dd, 1H, *J* = 9.6, 8.6 Hz, H-3), 3.78 – 3.69 (m, 2H, H-6), 3.61 – 3.56 (m, 1H, H-5), 3.51 (dd, 1H, *J* = 9.8, 8.6 Hz, H-4), 3.48 (dd, 1H, *J* = 9.5, 3.5 Hz, H-2), 3.36 (s, 3H, CH_3 OMe), 2.46 (bs, 2H, OH); ^{13}C-APT NMR ($CDCl_3$, 101 MHz, HSQC): δ 138.8, 138.1 ($C_{q\text{-}arom}$), 128.7, 128.6, 128.2, 128.1, 128.0, 127.9 (CH_{arom}), 98.2 (C-1), 81.4 (C-3), 79.9 (C-2), 75.5, 73.2 (CH_2 Bn), 70.8 (C-5), 70.3 (C-4), 62.3 (C-6), 55.3 (OMe);

Methyl (methyl 2,3-di-*O*-benzyl-α-D-glucopyranosyl uronate) (12)

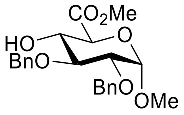

To a solution of diol **S12** (6.95 g, 18.6 mmol) in DCM (70 mL) and AcOH (0.1 mL) was added BAIB (14.95 g, 46.4 mmol, 2.5 eq.), TEMPO (580 mg, 3.7 mmol, 0.2 eq.) and H_2O (30 mL). The solution was stirred vigorously for 2.5 hours at room temperature, quenched by the addition of $Na_2S_2SO_3$ (10% aq.) and this suspension stirred for 15 min. The mixture was extracted two times with EtOAc and the combined organic fractions were dried ($MgSO_4$), filtered, concentrated *in vacuo* and coevaporated with toluene once. The crude carboxylic acid was dissolved in DMF (75 mL) and cooled to 0°C. K_2CO_3 (7.7 g, 55.7 mmol, 3 eq.) and MeI (3.5 mL, 55.7 mmol, 3 eq.) were added and the reaction mixture stirred overnight. H_2O was added and the reaction mixture was extracted twice with EtOAc. The combined organic layers where dried ($MgSO_4$), filtered and concentrated in vacuo. Purification by flash column chromatography (1/0 to 9/1 toluene/acetone) followed by recrystallization from DCM/EtOAc/petroleum ether (1/1/23) gave the title product as white needles (3.84 g, 9.54 mmol, 52%, 2 steps). Spectroscopic data were in accord with those previously reported.[21] $[\alpha]_D^{23}$ = +19.0° (c=1.0, $CHCl_3$), lit.: $[\alpha]_D^{30}$ = +17.9° (c=0.5, $CHCl_3$)[21] ; IR: 700, 738, 1040, 1061, 1738, 2918, 3532; ^{1}H NMR ($CDCl_3$, 400 MHz, HH-COSY, HSQC): δ 7.39 – 7.26 (m, 10H, CH_{arom}), 4.92 (d, 1H, *J* = 11.3 Hz, C*H*H Bn), 4.81 (d, 1H, *J* = 11.4 Hz, CH*H* Bn), 4.79 (d, 1H, *J* = 12.1 Hz, C*H*H Bn), 4.67 – 4.62 (m, 2H, CH*H* Bn, H-1), 4.15 (d, 1H, *J* = 8.9 Hz, H-5), 3.87 – 3.76 (m, 5H, H-3, H-4, CH_3 CO_2Me), 3.53 (dd, 1H, *J* = 8.9, 3.4 Hz, H-2), 3.42 (s, 3H, CH_3 OMe), 2.89 (bs, 1H, 4-OH); ^{13}C-APT NMR ($CDCl_3$, 101 MHz, HSQC): δ 170.8 (C=O CO_2Me), 138.7, 138.0 ($C_{q\text{-}arom}$), 128.6, 128.3, 128.1, 128.0, 127.9 (CH_{arom}), 98.8 (C-1), 80.5 (C-3), 78.6 (C-2), 75.6, 73.7 (CH_2 Bn), 71.9 (C-4), 70.6 (C-5), 56.0 (OMe), 52.8 (CO_2Me); HRMS: $[M+Na]^+$ calcd for $C_{22}H_{26}O_7Na$ 425.15707, found 425.15649.

Preparation of acceptor 13

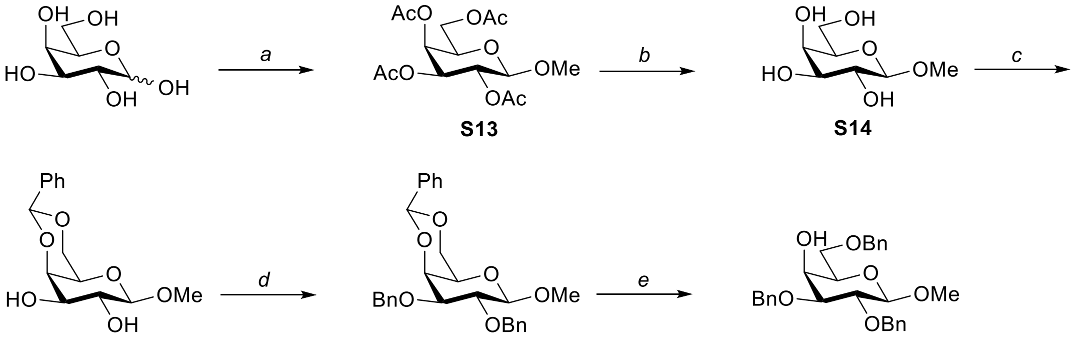

Scheme S-7: Galactose acceptor **13** synthesis. *Reagents and conditions:* a) *i.* Ac_2O, $HClO_4$; *ii.* HBr, AcOH; *iii.* I_2, MeOH, **S13**: 48% (three steps); b) NaOMe, MeOH; c) $PhCH(OMe)_2$, *p*-TsOH•H_2O, **S15**: 61% (two steps); d) BnBr, NaH, DMF, **S16**: 75%; e) $NaCNBH_3$, THF, HCl, dioxane, **13**: 74%.

Methyl 2,3,4,6-tetra-*O*-acetyl-β-D-galactopyranoside (S13)

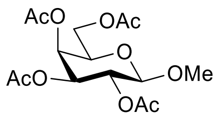

Following the procedure of Kartha *et al.*[22] D-galactose (4.5 g, 25 mmol) was portionwise added to a mixture of 70% aq. $HClO_4$ (cat., 5 drops) and Ac_2O (14.2 mL, 150 mmol, 6 eq.). After stirring overnight a 33 wt% solution of HBr in AcOH (13.1 mL, 75 mmol, 3 eq.) was added and the reaction stirred at r.t. for 5 h. Solvents were evaporated by a water aspirator and the crude product was dissolved in EtOAc and washed with cold sat.aq. $NaHCO_3$ and brine. The organic phase was dried Na_2SO_4 and concentrated *in vacuo*. The crude bromide was dissolved in MeOH (100 mL) and cooled to 0°C. Iodine (3.17 g, 12.5 mmol, 0.5 eq.) was added and the reaction was stirred for 2 h. The reaction mixture was quenched by sat. aq. $Na_2S_2O_4$ and extracted with Et_2O twice. The organic layer was washed with sat. aq. $NaHCO_3$ and brine, dried ($MgSO_4$), filtered, and concentrated *in vacuo*. Flash column chromatography (8/1 to 1/1 pentane/EtOAc) gave the methyl galactoside as an anomerically pure yellow oil (4.31 g, 11.9 mmol, 48% over three steps). Spectroscopic data were in accord with those previously reported.[23,24] ^1H NMR ($CDCl_3$, 400 MHz, HH-COSY, HSQC): δ 5.40 (dd, 1H, *J* = 3.4, 1.2 Hz, H-4), 5.20 (dd, 1H, *J* = 10.5, 7.9 Hz, H-2), 5.03 (dd, 1H, *J* = 10.5, 3.4 Hz, H-3), 4.42 (d, 1H, *J* = 7.9 Hz, H-1), 4.25 – 4.11 (m, 2H, H-6), 3.93 (td, 1H, *J* = 6.7, 1.2 Hz, H-5), 3.52 (s, 3H, CH_3 OMe), 2.16 (s, 3H, CH_3 Ac), 2.07 (s, 3H, CH_3 Ac), 2.06 (s, 3H, CH_3 Ac), 1.99 (s, 3H, CH_3 Ac); ^{13}C-APT NMR ($CDCl_3$, 101 MHz, HSQC): δ 170.4, 170.2, 170.1, 169.5 (C=O Ac), 102.0 (C-1), 71.0 (C-3), 70.6 (C-5), 68.8 (C-2), 67.1 (C-4), 61.3 (C-6), 57.0 (OMe), 20.8, 20.7, 20.7, 20.6 (CH_3 Ac);

Methyl β-D-galactopyranoside (S14)

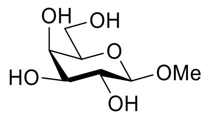

Acetylated **S13** (4.31 g, 11.9 mmol) was dissolved in MeOH (50 mL) and NaOMe (325 mg, 6.0 mmol, 0.5 eq.) was added. After 30 min Amberlite H^+ was added until neutral pH was achieved and the resin was subsequently filtered off. The solution

was concentrated *in vacuo* to give the crude tetra-ol. Spectroscopic data were in accord with those previously reported.[25] ^{1}H NMR (D_2O, 400 MHz, HSQC): δ 4.79 (bs, 4H, OH), 4.30 (d, 1H, *J* = 7.9 Hz, H-1), 3.90 (d, 1H, *J* = 3.5 Hz, H-4), 3.81 – 3.73 (m, 2H, H-6), 3.75 – 3.64 (m, 1H, H-5), 3.63 (dd, 1H, *J* = 9.9, 3.5 Hz, H-3), 3.55 (s, 3H, CH_3 OMe), 3.48 (dd, 1H, *J* = 9.9, 7.9 Hz, H-2); ^{13}C NMR (D_2O, 101 MHz, HSQC): δ 103.8 (C-1), 75.2 (C-5), 72.8 (C-3), 70.8 (C-2), 68.7 (C-4), 61.0 (C-6), 57.2 (OMe);

Methyl 4,6-*O*-benzylidene-β-D-galactopyranoside (S15)

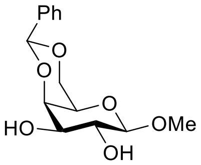

Crude **S14** (1.94 g, 10 mmol) and *p*-TsOH•H_2O were dissolved in CH_3CN (50 mL) and DMF (15 mL) and the poorly soluble reaction mixture was stirred at 60°C, 350 mbar for 3 h. Et_3N (0.8 mL) was added and the reaction mixture was portioned between EtOAc and H_2O. The organic layer did not contain observable product, therefore the water layer was evaporated to give the crude product. Column chromatography (1:0 to 9/1 DCM/MeOH) gave the benzylidene protected galactoside as a waxy solid (1.73 g, 6.1 mmol, 61%). Spectroscopic data were in accord with those previously reported.[11,19,26] ^{1}H NMR ($CDCl_3$, 400 MHz): δ 7.55 – 7.46 (m, 2H), 7.40 – 7.34 (m, 3H), 5.55 (s, 1H), 4.36 (dd, 1H, *J* = 12.5, 1.5 Hz), 4.24 – 4.20 (m, 2H), 4.12 – 4.07 (m, 1H), 3.79 – 3.67 (m, 2H), 3.59 (d, 3H, *J* = 0.7 Hz), 3.49 (t, 1H, *J* = 1.6 Hz);

Methyl 2,3-di-*O*-benzyl-4,6-*O*-benzylidene-β-D-galactopyranoside (S16)

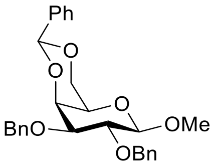

Compound **S15** was coevaporated with dry toluene twice before being dissolved in DMF (30 mL). Benzyl bromide (3.2 mL, 18.4 mmol, 3 eq.) and NaH (60% dispersion in mineral oil, 736 mg, 18.4 mmol, 3 eq.) were added and the reaction mixture was stirred overnight. H_2O was added and the mixture was extracted with EtOAc twice. The organic layer was washed with brine twice and dried ($MgSO_4$), filtered, and concentrated *in vacuo*. The crude product was purified by flash column chromatography (8/1 to 3/1 pentane/EtOAc) to afford the benzylated product (2.11 g, 4.56 mmol, 75%). R_f: 0.63 (3/2 pentane/EtOAc). Spectroscopic data were in accord with those previously reported.[11] ^{1}H NMR ($CDCl_3$, 400 MHz, HH-COSY, HSQC): δ 7.59 – 7.53 (m, 2H, CH_{arom}), 7.42 – 7.26 (m, 13H, CH_{arom}), 5.50 (s, 1H, C*H*Ph), 4.91 (d, 1H, *J* = 10.9 Hz, C*H*H Bn), 4.81 – 4.71 (m, 3H, CH*H* Bn, CH_2 Bn), 4.36 – 4.27 (m, 2H, H-1, H-6), 4.11 (dd, 1H, *J* = 3.7, 1.1 Hz, H-4), 4.02 (dd, 1H, *J* = 12.3, 1.8 Hz, H-6), 3.84 (dd, 1H, *J* = 9.7, 7.7 Hz, H-2), 3.60 – 3.53 (m, 4H, H-3, CH_3 OMe), 3.32 (d, 1H, *J* = 1.3 Hz, H-5); ^{13}C-APT NMR ($CDCl_3$, 101 MHz, HSQC): δ 139.0, 138.5, 137.9 (C_q), 129.1, 128.5, 128.4, 128.2, 128.2, 127.9, 127.8, 127.6, 126.7 (CH_{arom}), 104.8 (C-1), 101.5 (CHPh), 79.3 (C-3), 78.6 (C-2), 75.4 (CH_2 Bn), 74.1 (C-4), 72.1 (CH_2 Bn), 69.4 (C-6), 66.5 (C-5), 57.2 (OMe);

Methyl 2,3,6-tri-*O*-benzyl-β-D-galactopyranoside (13)

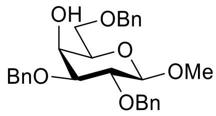

To a solution of **S16** (2.10 g, 4.54 mmol) and $NaCNBH_3$ (1.7 g, 27.2 mmol, 6 eq.) in THF (60 mL), 4.0 M HCl in 1,4-dioxane (9 mL, 36 mmol, 7.9 eq.) was added. The reaction mixture was stirred for 1 h and then H_2O was added. The solution was extracted twice with DCM and the organic layer was washed with brine, dried with $MgSO_4$ en concentrated *in vacuo*. Flash column chromatography (9/1 to 1/1 pentane/EtOAc) provided the free alcohol as an oil (1.56 g, 3.36 mmol, 74%). R_f: 0.74 (3/2 pentane/EtOAc). Spectroscopic data were in accord with those previously reported.[11,27,28] ^1H NMR ($CDCl_3$, 400 MHz, HH-COSY, HSQC): δ 7.41 – 7.21 (m, 15H, CH_{arom}), 4.88 (d, 1H, J = 11.1 Hz, C*H*H Bn), 4.71 (d, 1H, J = 11.0 Hz, CH*H* Bn), 4.67 (s, 2H, CH_2 Bn), 4.56 (s, 2H, CH_2 Bn), 4.26 (d, 1H, J = 7.7 Hz, H-1), 3.98 (d, 1H, J = 3.4 Hz, H-4)), 3.79 (dd, 1H, J = 9.9, 5.9 Hz, H-6), 3.72 (dd, 1H, J = 9.9, 6.0 Hz, H-6), 3.64 (dd, 1H, J = 9.4, 7.7 Hz, H-2), 3.59 – 3.50 (m, 4H, H-5, CH3 OMe), 3.46 (dd, 1H, J = 9.4, 3.4 Hz, H-3), 2.70 (s, 1H, 4-OH); ^{13}C-APT NMR ($CDCl_3$, 101 MHz, HSQC): δ 138.6, 137.9, 137.8 (C_q), 128.4, 128.3, 128.0, 127.8, 127.8, 127.7, 127.7, 127.5 (CH_{arom}), 104.7 (C-1), 80.5 (C-3), 79.0 (C-2), 75.1, 73.6 (CH_2 Bn), 73.1 (C-5), 72.3 (CH_2 Bn), 69.2 (C-6), 66.8 (C-4), 56.9 (OMe); HRMS: $[M+Na]^+$ calcd for $C_{28}H_{33}O_6Na$ 487.20911, found 487.20848.

Preparation of acceptor 14

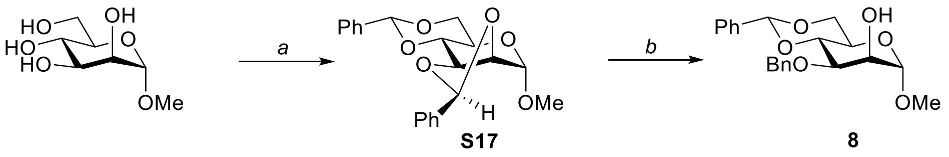

Scheme S-8: Mannose acceptor **14** synthesis. *Reagents and conditions:* a) $PhCH(OMe)_2$, *p*-TsOH•H_2O, CH_3CN, **S17**: 80%; b) $LiAlH_4$, $AlCl_3$, Et_2O, DCM, **14**: 96%.

Methyl 2,3-*exo*;4,6-di-*O*-benzylidene-α-D-mannopyranoside (S17)

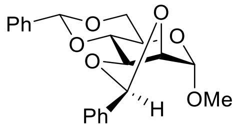

To a solution of methyl α-D-mannoside (19.4 g, 100 mmol) in CH_3CN (120 mL) was added benzylidene dimethyl acetal (36 mL, 240 mmol, 2.4 eq.) and *p*-TsOH•H_2O (475 mg, 2.5 mmol, 0.025 eq.). The reaction mixture was stirred at 60°C and 500 mbar for 3 h and the volume was reduced by half. Sat. aq. $NaHCO_3$ was added to quench the reaction and the precipitate collected and washed with cold H_2O. The solids were recrystallized from EtOH/EtOAc to obtain two crops of white needles (total yield: 29.6 g, 80 mmol, 80% exo only). Spectroscopic data were in accord with those previously reported.[29] ^1H NMR ($CDCl_3$, 400 MHz, HH-COSY, HSQC): δ 7.55 – 7.51 (m, 2H, CH_{arom}), 7.48 – 7.44 (m, 2H, CH_{arom}), 7.41 – 7.34 (m, 6H, CH_{arom}), 6.30 (s, 1H, C*H*$Ph_{2,3\,exo}$), 5.64 (s, 1H, C*H*$Ph_{4,6}$), 5.02 (s, 1H, H-1), 4.63 (dd, 1H, J = 7.8, 5.4 Hz, H-3), 4.40 – 4.32 (m, 1H, H-6), 4.14 (d, 1H, J = 5.4 Hz, H-2), 3.93 – 3.88 (m, 1H, H-4), 3.88 – 3.81 (m, 2H, H-5, H-6), 3.41 (s, 3H, CH_3 OMe); ^{13}C-APT NMR ($CDCl_3$, 101 MHz, HSQC): δ 138.7, 137.3 (C_q), 129.3, 128.5, 128.4, 126.4, 126.2 (CH_{arom}), 103.1 ($CHPh_{2,3\,exo}$), 102.2 ($CHPh_{4,6}$), 99.0 (C-1), 77.6 (C-4), 75.7 (C-3), 75.4 (C-2), 69.1 (C-6), 60.5 (C-5), 55.4 (OMe);

Methyl 3-*O*-benzyl-4,6-*O*-benzylidene-α-D-mannopyranoside (14)

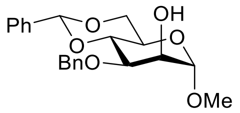

Compound **S17** (5.56 g, 15 mmol) was dissolved in 100 mL DCM and 150 mL Et_2O. A solution of $LiAlH_4$ (2.4 M in THF, 8 mL, 19.2 mmol, 1.3 eq.) was added to the reaction mixture at 0°C followed by addition of $AlCl_3$ (2.2 g, 16.4 mmol, 1.1 eq.). The reaction mixture was allowed to stir for 3 h at r.t. before being quenched by careful addition of EtOAc and H_2O. The mixture was extracted with EtOAc and the organic phase was washed with brine, dried $MgSO_4$ and concentrated under reduced pressure. Purification of the crude product by flash column chromatography (6/1 to 1/1 pentane/EtOAc) gave the title compound as a colorless oil (5.37 g, 14.4 mmol, 96%). R*f*: 0.38 (2/1 pentane/EtOAc). Spectroscopic data were in accord with those previously reported.[29–31] ^{1}H NMR ($CDCl_3$, 400 MHz, HH-COSY, HSQC): δ 7.53 – 7.47 (m, 2H, CH_{arom}), 7.41 – 7.27 (m, 8H, CH_{arom}), 5.60 (s, 1H, C*H*Ph), 4.84 (d, 1H, *J* = 11.9 Hz, C*H*H Bn), 4.73 (d, 1H, *J* = 1.4 Hz, H-1), 4.69 (d, 1H, *J* = 11.9 Hz, CH*H* Bn), 4.27 (dd, 1H, *J* = 9.4, 4.0 Hz, H-6), 4.09 (t, 1H, *J* = 9.2 Hz, H-4), 4.01 (dt, 1H, *J* = 3.3, 1.6 Hz, H-2), 3.93 – 3.75 (m, 3H, H-3, H-5, H-5), 3.35 (s, 3H, CH_3 OMe), 2.82 (d, 1H, *J* = 1.7 Hz, 2-OH); ^{13}C-APT NMR ($CDCl_3$, 101 MHz, HSQC): δ 138.1, 137.7 (C_q), 129.0, 128.6, 128.3, 128.0, 127.9, 126.2 (CH_{arom}), 101.7 (CHPh), 101.2 (C-1), 78.9 (C-4), 75.7 (C-3), 73.1 (CH_2 Bn), 69.9 (C-2), 69.0 (C-6), 63.3 (C-5), 55.0 (OMe); HRMS: $[M+Na]^+$ calcd for C21H24O6Na 395.14651, found 395.14638

Glycosylations with donor 1

Cyclohexyl 2,3-di-*O*-benzyl-4,6-*O*-benzylidene-α/β-D-mannopyranoside (1A).

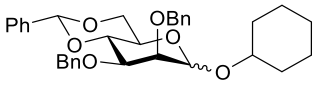

Donor **1** and cyclohexanol were condensed using the general procedure for Tf_2O/Ph_2SO mediated and purified by flash column chromatography (1/0 to 9/1 pentane/EtOAc) to yield glycosylation product **1A** (50.9 mg, 51 µmol, 96%, α:β = 1:5). R*f*: 0.43 (9/1 pentane/EtOAc). Spectroscopic data were in accord with those previously reported.[32,33] IR (neat): 694, 733, 964, 1026, 1047, 1084, 1361, 1452, 2857, 2857, 2930; Data for the β-anomer: ^{1}H NMR (400 MHz, $CDCl_3$, HH-COSY, HSQC): δ 7.55 – 7.23 (m, 15H, CH_{arom}), 5.61 (s, 1H, C*H*Ph), 5.02 (d, 1H, *J* = 12.5 Hz, C*H*H Bn), 4.91 (d, 1H, *J* = 12.5 Hz, CH*H* Bn), 4.67 (d, 1H, *J* = 12.5 Hz, C*H*H Bn), 4.58 (s, 1H, H-1), 4.58 (d, 1H, *J* = 12.5 Hz, CH*H* Bn), 4.30 (dd, 1H, *J* = 10.4, 4.9 Hz, H-6), 4.22 (t, 1H, *J* = 9.6 Hz, H-4), 3.94 (t, 1H, *J* = 10.3 Hz, H-6), 3.87 (d, 1H, *J* = 3.0 Hz, H-2), 3.70 (dt, 1H, *J* = 8.6, 4.7 Hz, CH Cy), 3.58 (dd, 1H, *J* = 9.9, 3.1 Hz, H-3), 3.31 (td, 1H, *J* = 9.9, 4.9 Hz, H-5), 2.06 – 0.99 (m, 10H, CH_2 Cy); ^{13}C-APT NMR (101 MHz, $CDCl_3$, HSQC): δ 138.68, 138.54, 137.79 (C_q), 129.05, 128.92, 128.85, 128.49, 128.40, 128.28, 128.22, 128.19, 127.84, 127.68, 127.62, 127.60, 127.58, 127.53, 126.18, 126.14, 125.21 (CH_{arom}), 101.48 (CHPh), 100.12 (C-1), 78.76 (C-4), 78.25 (C-3), 76.84 (CH Cy), 76.31 (C-2), 74.71 (CH_2 Bn), 72.39 (CH_2 Bn), 68.82 (C-6), 67.68 (C-5), 33.48, 31.57, 25.78, 23.87, 23.72 (CH_2 Cy); ^{13}C-GATED NMR (101 MHz, $CDCl_3$): 100.1 ($J_{C1,H1}$ = 154 Hz, C-1 β); Diagnostic peaks α-anomer: ^{1}H NMR (400 MHz, $CDCl_3$, HH-COSY, HSQC): δ 5.64 (s, 0.20H, C*H*Ph), 4.89 – 4.79 (m, 0.60H, C*H*H Bn, CH*H* Bn, C-1), 4.71 (d, 0.20H, *J* = 12.3 Hz, CH*H* Bn), 4.00 (dd, 0.20H, *J* = 10.0, 3.2 Hz, H-2), 3.78 (dd, 0.20H, *J* = 3.1, 1.6 Hz, H-3), 3.54 – 3.49 (m, 0.20H, CH Cy); ^{13}C-APT NMR (101 MHz, $CDCl_3$, HSQC): δ 97.41 (C-1), 64.36 (C-5), 33.38, 31.31, 25.69, 25.25, 24.11 (CH_2 Cy); HRMS: $[M+Na]^+$ calcd for $C_{33}H_{38}O_6Na$ 553.25606, found 553.25531.

Ethyl 2,3-di-*O*-benzyl-4,6-*O*-benzylidene-α/β-D-mannopyranoside (1B).

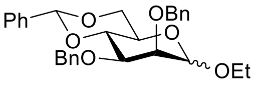

Donor **1** and ethanol were condensed using the general procedure for Tf_2O/Ph_2SO mediated glycosylations and purified by flash column chromatography (1/0 to 9/1 pentane/EtOAc) to yield glycosylation product **1B** (33.5 mg, 70 µmol, 70%, α:β = 1:5). R_f: 0.43 (9/1 pentane/EtOAc). IR (neat): 696, 734, 893, 912, 968, 1004, 1049, 1088, 1373, 1452, 2866, 2926; Data for the β-anomer: ^{1}H NMR (400 MHz, $CDCl_3$, HH-COSY, HSQC): δ 7.53 – 7.28 (m, 15H, CH_{arom}), 5.62 (s, 1H, C*H*Ph), 4.99 (d, 1H, *J* = 12.4 Hz, C*H*H Bn), 4.89 (d, 1H, *J* = 12.4 Hz, CH*H* Bn), 4.68 (d, 1H, *J* = 12.6 Hz, C*H*H Bn), 4.58 (d, 1H, *J* = 12.5 Hz, CH*H* Bn), 4.46 (s, 1H, H-1), 4.31 (dd, 1H, *J* = 10.4, 4.9 Hz, H-6), 4.21 (t, 1H, *J* = 9.6 Hz, H-4), 4.02 – 3.89 (m, 3H, C*H*HCH$_3$ Et, H-2, H-6), 3.58 (dd, 1H, *J* = 9.9, 3.1 Hz, H-3), 3.56 – 3.47 (m, 1H, CH*H*CH$_3$ Et), 3.36 – 3.28 (m, 1H, H-5), 1.27 (t, 3H, *J* = 7.0 Hz, CH_3 Et); ^{13}C-APT NMR (101 MHz, $CDCl_3$, HSQC): δ 138.6, 138.5, 137.8 (C_q), 123.0, 128.9, 128.4, 128.3, 128.2, 127.7, 127.7, 127.6, 126.2, (CH_{arom}) 102.2 (C-1), 101.5 (CHPh), 78.8 (C-4), 78.0 (C-3), 75.9 (C-2), 74.8 (CH_2 Bn), 72.5 (CH_2 Bn), 68.8 (C-6), 67.7 (C-5), 65.7 (CH_2 Et), 15.3 (CH_3 Et); ^{13}C-GATED NMR (101 MHz, $CDCl_3$): δ 102.2 ($J_{C1,H1}$ = 153 Hz, C-1 β); Diagnostic peaks α-anomer: ^{1}H NMR (400 MHz, $CDCl_3$): δ 5.65 (s, 0.20H), 4.86 – 4.81 (m, 0.40H, C*H*H Bn, CH*H* Bn), 4.80 (d, 0.20H, *J* = 1.5 Hz, H-1), 4.74 (d, 0.20H, *J* = 12.3 Hz, C*H*H Bn), 3.74 – 3.66 (m, 0.20H, CH*H*CH$_3$), 3.46 – 3.39 (m, 0.20H, CH*H*CH$_3$ Et); ^{13}C-APT NMR (101 MHz, $CDCl_3$, HSQC): δ 128.9, 128.5, 128.4, 128.3, 128.2, 127.9, 127.6, 126.2 (C_q), 101.6 (CHPh), 99.3 (C-1), 79.4 (C-3), 76.5 (C-4), 76.4 (C-2), 73.7 (CH_2 Bn), 73.3 (CH_2 Bn), 69.3 (C-6), 64.3 (C-5) 63.3 (CH_2 Et), 15.1 (CH_3 Et); HRMS: $[M+Na]^+$ calcd for $C_{29}H_{32}O_6Na$ 499.20911, found 499.20846.

2-Fluoroethyl 2,3-di-*O*-benzyl-4,6-*O*-benzylidene-α/β-D-mannopyranoside (1C).

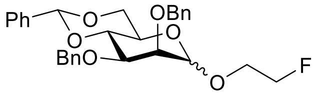

Donor **1** and 2-fluoroethanol were condensed using the general procedure for Tf_2O/Ph_2SO mediated and purified by flash column chromatography (1/0 to 4/1 pentane/EtOAc) to yield glycosylation product **1C** (42.7 mg, 86 µmol, 86%, α:β = 1:5). R_f: 0.18 (9/1 pentane/EtOAc). IR (neat): 696, 738, 802, 887, 1025, 1043, 1066, 1086, 1261, 1371, 1454, 2870; Data for the β-anomer: ^{1}H NMR (400 MHz, $CDCl_3$, HH-COSY, HSQC): δ 7.66 – 7.32 (m, 15H, CH_{arom}), 5.62 (s, 1H, C*H*Ph), 4.99 (d, 1H, *J* = 12.3 Hz, C*H*H Bn), 4.89 (d, 1H, *J* = 12.3 Hz, CH*H* Bn), 4.70 – 4.51 (m, 5H, C*H*H Bn, CH*H* Bn, H-1, CH$_2$C*H*HF, CH$_2$CH*H*F), 4.30 (dd, 1H, *J* = 10.4, 4.8 Hz, H-6), 4.22 (t, 1H, *J* = 9.6 Hz, H-4), 4.08 (ddt, 1H, *J* = 35.7, 12.2, 3.0 Hz, C*H*HCH$_2$F), 3.98 (d, 1H, *J* = 2.9 Hz, H-2), 3.92 (t, 1H, *J* = 10.3 Hz, H-6), 3.80 (dtd, 1H, *J* = 22.6, 11.9, 7.8, 2.4 Hz, CH*H*CH$_2$F), 3.59 (dd, 1H, *J* = 9.9, 3.1 Hz, H-3), 3.33 (td, 1H, *J* = 9.7, 4.9 Hz, H-5); ^{13}C-APT NMR (101 MHz, $CDCl_3$, HSQC): δ 138.4, 138.4, 137.6 (C_q), 131.2, 129.4, 129.0, 128.8, 128.4, 128.3, 128.2, 127.7, 127.7, 126.2, 124.9 (CH_{arom}), 102.3 (C-1), 101.5 (CHPh), 82.8 (d, *J* = 169.74 Hz, CH_2F), 78.6 (C-4), 77.8 (C-3), 75.7 (C-2), 75.0 (CH_2 Bn), 72.5 (CH_2 Bn), 69.0 (d, *J* = 19.7 Hz, *CH$_2$*CH$_2$F), 67.7 (C-6). ^{13}C-GATED NMR (101 MHz, $CDCl_3$): δ 102.3 ($J_{C1,H1}$ = 156 Hz, C-1 β); Diagnostic peaks α-anomer: ^{1}H NMR (400 MHz, $CDCl_3$, HH-COSY, HSQC): δ 5.62 (s, 0.20H, C*H*Ph), 4.93 – 4.80 (m, 0.60H, C*H*H Bn, CH*H* Bn, H-1); ^{13}C-APT NMR (101 MHz, $CDCl_3$, HSQC): δ 99.7 (C-1), 82.5 (d, *J* = 170 Hz, CH_2F), 79.2 (C-4), 76.5 (C-3), 76.4 (C-2), 73.8 (CH_2 Bn), 73.3 (CH_2 Bn), 68.9 (C-6), 66.7 (d, *J* = 19.9 Hz, *CH$_2$*CH$_2$F), 64.4 (C-5); ^{13}C-GATED NMR (101 MHz, $CDCl_3$): δ 99.7 ($J_{C1,H1}$ = 170 Hz, C-1 α); HRMS: $[M+Na]^+$ calcd for $C_{29}H_{31}FO_6Na$ 517.19969, found 517.19888.

2,2-Difluoroethyl 2,3-di-*O*-benzyl-4,6-*O*-benzylidene-α/β-D-mannopyranoside (1D).

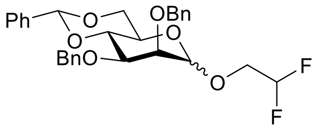

Donor **1** and 2,2-difluoroethanol were condensed using the general procedure for Tf_2O/Ph_2SO mediated glycosylations and purified by flash column chromatography (1/0 to 4/1 pentane/EtOAc) to yield glycosylation product **1D** (46.1 mg, 90 µmol, 90%, α:β = 1:5). R_f: 0.50 (9/1 pentane/EtOAc). IR (neat): 694, 744, 795, 1026, 1094, 1261, 1369, 1454, 2868; Data for the β-anomer: ^{1}H NMR (400 MHz, $CDCl_3$, HH-COSY, HSQC): δ 7.68 – 7.27 (m, 15H, CH_{arom}), 5.90 (dddd, 1H, *J* = 54.8, 5.1, 2.8, 1.5 Hz, CHF_2) 5.62 (s, 1H, C*H*Ph), 4.94 (d, 1H, *J* = 12.2 Hz, C*H*H Bn), 4.86 (d, 1H, *J* = 12.2 Hz, CH*H* Bn), 4.70 (d, 1H, *J* = 12.5 Hz, C*H*H Bn), 4.60 (d, 1H, *J* = 12.4 Hz, CH*H* Bn), 4.51 (s, 1H. H-1), 4.31 (dd, 1H, *J* = 10.4, 4.9 Hz, H-6), 4.22 (t, 1H, *J* = 9.6 Hz, H-4), 4.05 (dtd, 1H, *J* = 20.7, 11.1, 2.9 Hz, C*H*HCHF$_2$), 3.98 – 3.88 (m, 2H, H-2, H-6), 3.82 – 3.65 (m, 1H, CH*H*CHF$_2$), 3.59 (dd, 1H, *J* = 9.9, 3.1 Hz, H-3), 3.33 (td, 1H, *J* = 9.7, 4.8 Hz, H-5); ^{13}C-APT NMR (101 MHz, $CDCl_3$, HSQC): δ 138.3, 138.2, 137.5 (C_q), 128.8, 128.5, 128.3, 127.7, 126.2 (C_{arom}), 115.4 (t, *J* = 241.9, CHF_2), 102.3 (C-1), 101.6 (CHPh), 78.6 (C-4), 77.8 (C-3), 75.5 (C-2), 75.1 (CH_2 Bn), 72.6 (CH_2 Bn), 68.5 (t, *J* = 33.0 Hz, *CH$_2$*CHF$_2$), 68.5 (C-6), 67.8 (C-5); ^{13}C-GATED NMR (101 MHz, $CDCl_3$): 102.3 ($J_{C1,H1}$ = 156 Hz, C-1 β); Diagnostic peaks α-anomer: ^{1}H NMR (400 MHz, $CDCl_3$, HH-COSY, HSQC): δ 5.98 (dt, 0.03 H, *J* = 5.7, 4.1 Hz, CHF_2), 5.84 (dt, 0.10H, *J* = 5.9, 4.1 Hz, CHF_2), 5.70 (dt, 0.03H, *J* = 6.1, 4.1 Hz, CHF_2), 4.84 (m, 0.34H, C-1, C*H*H Bn), 4.72 (d, 0.17H, *J* = 12.1 Hz, CH*H* Bn), 4.66 (d, 0.17H, *J* = 12.2 Hz, C*H*H Bn); ^{13}C-APT NMR (101 MHz, $CDCl_3$, HSQC): δ 115.4 (t, *J* = 240.10, CHF_2), 101.6 (CHPh), 100.1 (C-1), 79.0 (C-4), 76.3 (C-3), 76.2 (C-2), 73.9 (CH_2 Bn), 73.4 (CH_2 Bn), 68.5 (C-6), 64.8 (C-5); HRMS: $[M+Na]^+$ calcd for $C_{29}H_{30}F_2O_6Na$ 535.19027, found 535.18950.

2,2,2-Trifluoroethyl 2,3-di-*O*-benzyl-4,6-*O*-benzylidene-α/β-D-mannopyranoside (1E).

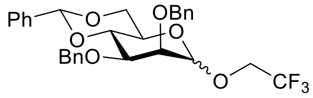

Donor **1** and 2,2,2-trifluoroethanol were condensed using the general procedure for Tf_2O/Ph_2SO mediated glycosylations and purified by flash column chromatography (1/0 to 9/1 pentane/EtOAc) to yield glycosylation product **1E** (41.7 mg, 79 µmol, 79%, α:β = 1:3.4). R_f: 0.60 (9/1 pentane/EtOAc). IR (neat): 696, 737, 1028, 1057, 1085, 1161, 1277, 1454, 2870; Data for the β-anomer: ^{1}H NMR (400 MHz, $CDCl_3$, HH-COSY, HSQC): δ 7.50 – 7.28 (m, 15H, CH_{arom}) 5.62 (s, 1H, C*H*Ph), 4.96 (d, 1H, *J* = 12.2 Hz, C*H*H Bn), 4.87 (d, 1H, *J* = 12.1 Hz, CH*H* Bn), 4.69 (d, 1H, *J* = 12.5 Hz, C*H*H Bn), 4.59 (d, 1H, *J* = 12.5 Hz, CH*H* Bn), 4.57 (s, 1H, H-1), 4.31 (dd, 1H, *J* = 10.4, 4.9 Hz, C-6), 4.28 – 4.17 (m, 2H, C-4, C*H*HCF$_3$), 4.01 – 3.86 (m, 3H, H-2, H-6, CH*H*CF$_3$), 3.59 (dd, 1H, *J* = 9.9, 3.1 Hz, H-3), 3.34 (td, 1H, *J* = 9.8, 4.9 Hz, H-5); ^{13}C-APT NMR (101 MHz, $CDCl_3$, HSQC): δ 138.2, 138.0, 137.5 (C_q), 129.1, 128.9, 128.5, 128.3, 127.9, 127.8, 127.7, 126.2 (C_{arom}), 123.7 (q, *J* = 277.6 Hz, CF_3) 101.9 (C-1), 101.6 (CHPh), 78.4 (C-4), 77.7 (C-3), 77.5 (C-2), 75.1 (CH_2 Bn), 75.0 (CH_2 Bn), 72.6 (C-6), 68.4 (C-5), 66.2 (q, *J* = 34.9 Hz, *CH$_2$*CF$_3$); ^{13}C-GATED NMR (101 MHz, $CDCl_3$): 101.9 ($J_{C1,H1}$ = 157 Hz, C-1 β); Diagnostic peaks α-anomer: ^{1}H NMR (400 MHz, $CDCl_3$, HH-COSY, HSQC): δ 5.64 (s, 0.29H, C*H*Ph), 4.88 – 4.82 (m, 0.87H, C*H*H Bn, CH*H* Bn, H-1), 4.73 – 4.64 (m, 0.58H, C*H*H Bn, CH*H* Bn); ^{13}C-APT NMR (101 MHz, $CDCl_3$, HSQC): δ 101.6 (CHPh), 100.1 (C-1), 78.9 (C-4), 76.2 (C-3), 76.0 (C-2), 74.1 (CH_2 Bn), 73.5 (CH_2 Bn), 68.7 (C-6), 65.0 (C-5); HRMS: $[M+Na]^+$ calcd for $C_{29}H_{29}F_3O_6Na$ 553.18084, found 553.18021.

1,1,1,3,3,3-Hexafluoro-2-propyl 2,3-di-*O*-benzyl-4,6-*O*-benzylidene-α/β-D-mannopyranoside (1F).

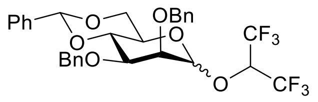

Donor **1** and 1,1,1,3,3,3-hexafluoro-2-propanol were condensed using the general procedure for Tf_2O/Ph_2SO mediated glycosylations (for an additional 120 hours at -40°C) and purified by flash column chromatography (1/0 to 9/1 pentane/EtOAc) to yield glycosylation product **1F** (33.6 mg, 34 µmol, 56%, α:β = 3.3:1). R_f: 0.81 (8/2 pentane/EtOAc). IR (neat): 694, 898, 977, 1058, 1091, 1195, 1217, 1287, 136, 2924; Data for the α-anomer: ^{1}H NMR (400 MHz, $CDCl_3$, HH-COSY, HSQC): δ 7.54 – 7.27 (m, 15H, CH_{arom}), 5.64 (s, 1H, C*H*Ph), 4.95 (s, 1H, H-1), 4.86 (d, 1H, *J* = 5.5 Hz, C*H*H Bn), 4.82 (d, 1H, *J* = 8.9 Hz, CH*H* Bn), 4.69 (d, 1H, *J* = 2.8 Hz, C*H*H Bn), 4.65 (d, 1H, *J* = 7.7 Hz, CH*H* Bn), 4.38 – 4.19 (m, 3H, H-3, H-6, $CH(CF_3)_2$), 3.92 (d, 1H, *J* = 4.9 Hz, H-4), 3.89 – 3.83 (m, 2H, H-6, H-5); ^{13}C-APT NMR (101 MHz, $CDCl_3$, HSQC): δ 138.5, 137.5, 137.4 (C_q), 129.1, 128.7, 128.5, 128.4, 128.3, 127.8, 127.7, 126.1 (CH_{arom}), 121.6 (q, *J* = 282.7 Hz, CF_3), 101.8 (C-1), 101.6 (CHPh), 78.4 (C-3), 76.1 (C-4), 75.5 (C-2), 74.3 (CH_2 Bn), 73.8 (CH_2 Bn), 72.4 (hept, *J* = 32.7 Hz, *CH*$(CF_3)_2$), 72.1 (CH_2 Bn), 68.3 (C-6), 65.8 (C-5);^{13}C-GATED NMR (101 MHz, $CDCl_3$): δ 101.8 ($J_{C1,H1}$ = 175 Hz, C-1 α); Diagnostic peaks β-anomer: ^{1}H NMR (400 MHz, $CDCl_3$, HH-COSY, HSQC, HMBC): δ 5.61 (s, 0.30H, C*H*Ph), 4.82 (d, 0.60H, *J* = 11.9 Hz, C*H*H Bn), 4.79 (s, 0.30H, H-1,), 4.69 (d, 0.30H, *J* = 12.9 Hz, CH*H* Bn), 4.02 (d, 0.30H, *J* = 2.9 Hz, H-2), 3.61 (dd, 0.30H, *J* = 9.9, 3.1 Hz, H-3), 3.36 (td, 0.30H, *J* = 9.9, 4.9 Hz, H-5); ^{13}C-APT NMR (101 MHz, $CDCl_3$, HSQC, HMBC): δ 101.3 (C-1), 72.6 (C-6), 68.2 (C-5); ^{13}C HMBC-GATED NMR (101 MHz, $CDCl_3$): δ 101.3 ($J_{C1,H1}$ = 159 Hz, C-1 β); HRMS: $[M+Na]^+$ calcd for $C_{30}H_{28}F_6O_6Na$ 621.16823, found 621.16790.

1-[^{2}H]-1,5-anhydro-2,3-di-*O*-benzyl-4,6-*O*-benzylidene-α-D-mannitol (1G)

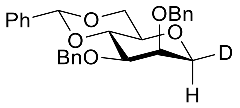

Donor **1** and triethylsilane-D were condensed using the general procedure for Tf_2O/Ph_2SO mediated glycosylations (for an additional 24 hours at -40°C) and purified by flash column chromatography (19/1 to 3/1 pentane/Et_2O) to yield glycosylation product **42** (25.8 mg, 60 µmol, 60%, α:β = < 1:20). R_f: 0.2 (4/1 pentane/Et_2O). Spectroscopic data of the non-dueterated mannitol were in accord with those previously reported.[34] $[\alpha]_D^{22}$ = -27.2° (*c* = 0.5, $CHCl_3$); IR (neat): 694, 733, 1092, 1119, 1452, 2349, 2866; Data for the β-anomer: ^{1}H NMR ($CDCl_3$, 400 MHz, HH-COSY, HSQC): δ 7.54 – 7.48 (m, 2H, CH_{arom}), 7.44 – 7.26 (m, 13H, CH_{arom}), 5.65 (s, 1H, C*H*Ph), 4.82 (d, 1H, *J* = 12.6 Hz, C*H*H Bn), 4.81 (d, 1H, *J* = 12.5 Hz, C*H*H Bn), 4.76 (d, 1H, *J* = 12.5 Hz, CH*H* Bn), 4.68 (d, 1H, *J* = 12.4 Hz, CH*H* Bn), 4.28 (dd, 1H, *J* = 11.2, 4.1 Hz, H-6), 4.25 (t, 1H, *J* = 10.1 Hz, H-4), 3.85 (t, 1H, *J* = 10.3 Hz, H-6), 3.79 (d, 1H, *J* = 3.3 Hz, H-2), 3.68 (dd, 1H, *J* = 9.8, 3.3 Hz, H-3), 3.42 (s, 1H, H-1), 3.34 (td, 1H, *J* = 9.7, 4.9 Hz, H-5); ^{13}C-APT NMR ($CDCl_3$, 101 MHz, HSQC): δ 138.6, 138.3, 137.8 (C_q), 128.9, 128.5, 128.4, 128.3, 128.2, 127.9, 127.7, 127.7, 126.1 (CH_{arom}), 101.5 (CHPh), 79.4 (C-4), 78.6 (C-3), 74.4 (C-2), 72.7 (CH_2 Bn), 72.5 (C-5), 72.4 (CH_2 Bn), 68.8 (t, *J* = 22 Hz, C-1), 68.7 (C-6); ^{2}H NMR ($CHCl_3$, 77 MHz): δ 4.08 (D-1); HRMS: $[M+H]^+$ calcd for $C_{27}H_{28}DO_5$ 434.20723, found 434.20691.

Allyl 2,3-di-*O*-benzyl-4,6-*O*-benzylidene-1-deoxy-β-D-mannopyranose (1H).

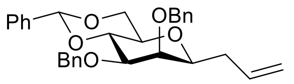

Donor **1** and allyl trimethylsilane were condensed using the general procedure for Tf_2O/Ph_2SO mediated glycosylations (for an additional 96 hours at -40°C) and purified by flash column chromatography (1/0 to 9/1 pentane/EtOAc) to yield glycosylation product **1H** (20.7 mg, 44 µmol, 44%, α:β = < 1:20). R_f: 0.80 (9/1 pentane/EtOAc). Spectroscopic data were in accord with those previously reported.[35] $[\alpha]_D^{26}$ = -19.6° (c = 0.5, $CHCl_3$); IR (neat): 696, 1028, 1097, 1454, 2860, 2924; Data for the β-anomer: ^{1}H NMR (400 MHz, $CDCl_3$, HH-COSY, HSQC, NOESY): δ 7.52 – 7.26 (m, 15H, CH_{arom}), 5.76 – 5.59 (m, 1H, C*H*CH_2 allyl), 5.64 (s, 1H, C*H*Ph), 5.11 – 4.98 (m, 3H, C*H*H Bn, CHC*H₂* allyl), 4.92 (d, 1H, *J* = 12.3 Hz, C*H*H Bn), 4.76 (d, 1H, *J* = 12.3 Hz, CH*H* Bn), 4.69 (d, 1H, *J* = 11.4 Hz, CH*H* Bn), 4.35 – 4.16 (m, 2H, H-4, H-6), 3.84 (t, 1H, *J* = 10.3 Hz, H-6), 3.80 (d, 1H, *J* = 2.2 Hz, H-2), 3.73 (dd, 1H, *J* = 9.8, 2.9 Hz, H-3), 3.45 (t, 1H, *J* = 6.8 Hz, H-1), 3.38 (td, 1H, *J* = 9.8, 4.9 Hz, H-5), 2.46 (dt, 1H, *J* = 13.5, 6.7 Hz, C*H*HCH allylic), 2.25 (dt, 1H, *J* = 14.3, 7.2 Hz, CH*H*CH allylic); ^{13}C-APT NMR (101 MHz, $CDCl_3$, HSQC, HMBC): δ 138.8, 138.6, 137.9 (C_q), 134.4 (*CH*CH_2 allyl), 128.9, 128.6, 128.5, 128.4, 128.3, 127.8, 127.7, 127.7, 126.2 (CH_{arom}), 117.6 (CHCH_2 allyl), 101.5 (CHPh), 80.9 (C-3), 79.8 (C-1), 79.7 (C-4), 76.6 (C-2), 75.1 (CH_2 Bn), 73.3 (CH_2 Bn), 72.1 (C-5), 68.8 (C-6), 35.6 (CH_2CH allylic); HRMS: $[M+H]^+$ calcd for $C_{30}H_{33}O_5$ 473.23225, found 473.23219.

Methyl 6-*O*-(2,3-di-*O*-benzyl-4,6-*O*-benzylidene-α/β-D-mannopyranosyl)-2,3,4-tri-*O*-benzyl-α-D-glucopyranoside (20).

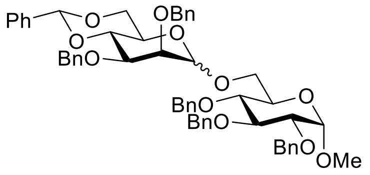

Donor **1** and acceptor **10** were condensed using the general procedure for Tf_2O/Ph_2SO mediated glycosylations (for an additional 16 hours at -40°C) and purified by flash column chromatography (9/1 to 7/3 pentane/EtOAc) to yield glycosylation product **20** (86.6mg, 97 µmol, 97%, α:β = 1:10). R_f: 0.67 (7/3 pentane/EtOAc). Spectroscopic data were in accord with those previously reported.[32,36,37] $[\alpha]_D^{26}$ +5.2° (c = 1, $CHCl_3$, 546 nm), $[\alpha]_D^{26}$ 0.0° (c = 1, $CHCl_3$, 589 nm), (lit:[36] $[\alpha]_D^{20}$ = -1.7° (c = 1.8, $CHCl_3$), lit:[32] $[\alpha]_D^{27}$ = -5.8° (c = 0.94, $CHCl_3$)); IR (neat): 731, 1026, 1049, 1084, 1452, 2872; Data for the β-anomer: ^{1}H NMR (400 MHz, $CDCl_3$, HH-COSY, HSQC): δ 7.58 – 7.05 (m, 30H, CH_{arom}), 5.59 (s, 1H, C*H*Ph), 5.03 (d, 1H, *J* = 10.9 Hz, C*H*H Bn), 4.92 (d, 1H, *J* = 12.3 Hz, C*H*H Bn), 4.86 – 4.76 (m, 4H, C*H*H Bn, C*H*H Bn, CH*H* Bn, CH*H* Bn), 4.72 (d, 1H, *J* = 12.5 Hz, C*H*H Bn), 4.67 (d, 1H, *J* = 12.2 Hz, CH*H* Bn), 4.61 (d, 1H, *J* = 12.6 Hz, CH*H* Bn), 4.58 (d, 1H, *J* = 3.5 Hz, H-1), 4.50 (d, 1H, *J* = 11.6 Hz, CH*H* Bn), 4.25 (dd, 1H, *J* = 10.4, 4.8 Hz, H-6’), 4.18 (t, 1H, *J* = 9.6 Hz, H-4’), 4.08 (m, 2H, H-1’, H-6), 4.02 (t, 1H, *J* = 9.3 Hz, H-4), 3.91 (t, 1H, *J* = 10.3 Hz, H-6’), 3.80 – 3.72 (m, 1H, H-2), 3.69 (d, 1H, *J* = 2.9 Hz, H-2’), 3.47 (m, 4H, H-3, H-3’, H-5, H-6), 3.33 (s, 3H, CH_3 OMe), 3.22 (td, 1H, *J* = 9.8, 4.8 Hz, H-5’); ^{13}C-APT NMR (101 MHz, $CDCl_3$): δ 138.9, 138.5, 138.5, 138.5, 138.1, 137.7 (C_q), 129.0, 128.7, 128.6, 128.5, 128.5, 128.5, 128.3, 128.3, 128.2, 128.1, 128.1, 127.8, 127.8, 127.7, 126.1 (CH_{arom}), 102.1 (H-1’), 101.5 (CHPh), 97.9 (C-1), 82.3 (C-4), 79.9 (C-3), 78.8 (C-4), 77.9 (C-3’), 76.8 (C-5), 75.8 (CH_2 Bn), 75.7 (C-2’), 74.8 (CH_2 Bn), 74.6 (CH_2 Bn), 73.5 (CH_2 Bn), 72.6 (CH_2 Bn), 69.7 (C-2), 68.7 (C-6’), 68.3 (C-6), 67.7 (C-5’), 55.2 (CH_3 OMe); HRMS: $[M+Na]^+$ calcd for $C_{55}H_{58}O_{11}Na$ 917.38713, found 917.38729.

Methyl 4-*O*-(2,3-di-*O*-benzyl-4,6-*O*-benzylidene-α/β-D-mannopyranosyl)-2,3,6-tri-*O*-benzyl-α-D-glucopyranoside (21).

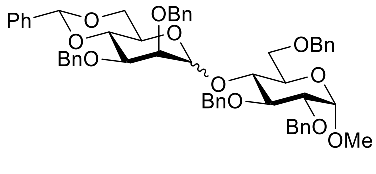

Donor **1** and acceptor **11** were condensed using the general procedure for Tf_2O/Ph_2SO mediated glycosylations (for an additional 16 hours at -40°C) and purified by flash column chromatography (9/1 to 7/3 pentane/EtOAc) to yield glycosylation product **21** (67.4 mg, 75 µmol, 75%, α:β = 1:9). R_f: 0.67 (7/3 pentane/EtOAc). Spectroscopic data were in accord with those previously reported.[32,36–38] $[\alpha]_D^{26}$ –15.8° (c = 1, $CHCl_3$), (lit:[38] $[\alpha]_D^{25}$ = –15.5° (c = 0.8, $CHCl_3$)); IR (neat): 735, 1028, 1083, 1452, 2862; Data for the β-anomer: ^1H NMR (400 MHz, $CDCl_3$, HH-COSY, HSQC): δ 7.46 – 7.21 (m, 25H, CH_{arom}), 5.51 (s, 1H, C*H*Ph), 5.05 (d, 1H, J = 10.6 Hz, C*H*H Bn), 4.84 – 4.70 (m, 5H, CH*H* Bn, C*H*H Bn, CH*H* Bn, C*H*H Bn, C*H*H Bn), 4.70 – 4.52 (m, 4H, CH*H* Bn, CH*H* Bn, C*H*H Bn, H-1), 4.36 (s, 1H, H-1'), 4.28 (d, 1H, J = 12.1 Hz, CH*H* Bn), 4.12 – 4.01 (m, 2H, H-4', H-6), 3.94 – 3.81 (m, 2H, H-3, H-4), 3.63 (d, 1H, J = 2.9 Hz, H-2'), 3.62 – 3.47 (m, 4H, H-5, H-2, H-6, H-6', H-6'), 3.47, 3.40 (s, 3H, CH_3 OMe), 3.32 (dd, 1H, J = 9.8, 3.0 Hz, H-3'), 3.05 (td, 1H, J = 9.7, 4.8 Hz, H-5'); ^{13}C-APT NMR (101 MHz, $CDCl_3$, HSQC): δ 139.5, 138.8, 138.7, 138.4, 137.8, 137.6 (C_q), 128.9, 128.7, 128.6, 128.5, 128.5, 128.4, 128.3, 128.2, 128.1, 127.9, 127.8, 127.6, 127.6, 127.4, 127.3, 126.2 (CH_{arom}), 101.7 (C-1'), 101.4 (CHPh), 98.5 (C-1), 80.4 (C-4), 79.1 (C-2), 78.8 (C-4'), 78.4 (C-3'), 77.8 (C-3), 77.1 (C-2), 75.4 (CH_2 Bn), 75.1 (CH_2 Bn), 73.8 (CH_2 Bn), 73.7 (CH_2 Bn), 72.6 (CH_2 Bn), 69.7 (C-5), 68.7 (C-6), 68.4 (C-6'), 67.4 (C-5), 55.5 (CH_3 OMe); ^{13}C-GATED NMR (101 MHz, $CDCl_3$): 101.7 ($J_{C1,H1}$ = 156 Hz, C-1 β); Diagnostic peaks α-anomer: ^1H NMR (400 MHz, $CDCl_3$, HH-COSY, HSQC): δ 5.60 (s, 0.11H, C*H*Ph), 5.30 (d, 0.11H, J = 1.3 Hz, C-1'); ^{13}C-APT NMR (101 MHz, $CDCl_3$, HSQC): δ 101.5 (C-1'), 101.4 (CHPh); HRMS: $[M+Na]^+$ calcd for $C_{55}H_{58}O_{11}Na$ 917.38713, found 917.38706.

Methyl (methyl [4-*O*-(2,3-di-*O*-benzyl-4,6-*O*-benzylidene-*α*/β-D-mannopyranosyl]-2,3-di-*O*-benzyl-*α*-D-glucopyranosyl uronate) (22).

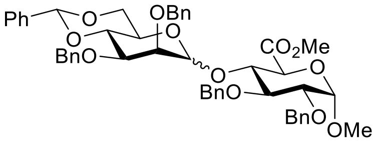

Donor **1** and acceptor **12** were condensed using the general procedure for Tf_2O/Ph_2SO mediated glycosylations (for an additional 48 hours at -40°C) and purified by flash column chromatography (9/1 to 7/3 pentane/EtOAc) to yield glycosylation product **22** (72.8 mg, 87 µmol, 87%, α:β = 1:10). R_f: 0.65 (7/3 pentane/EtOAc); $[\alpha]_D^{26}$ = -19.2° (c = 1, $CHCl_3$); IR (neat): 735, 1045, 1084, 1454, 1748, 2866; Data for the β-anomer: ^1H NMR (400 MHz, $CDCl_3$, HH-COSY, HSQC): δ 7.55 – 7.24 (m, 25H, CH_{arom}), 5.54 (s, 1H, C*H*Ph), 5.06 (d, 1H, J = 10.6 Hz, C*H*H Bn), 4.88 – 4.71 (m, 5H, CH*H* Bn, C*H*H Bn, CH*H* Bn, C*H*HBn, C*H*H Bn), 4.67 – 4.53 (m, 4H, CH*H* Bn, CH*H* Bn, H-1), 4.45 (s, 1H, H-1), 4.17 – 4.01 (m, 3H, H-4, H-4', H-6'), 3.95 – 3.85 (m, 2H, H-3, H-5), 3.82 – 3.75 (m, 1H, H-2'), 3.65 – 3.55 (m, 4H, H-6', CH_3 CO_2Me), 3.55 – 3.47 (m, 2H, H-2, H-3'), 3.44 (s, 3H, CH_3 OMe), 3.19 (td, 1H, J = 9.6, 4.8 Hz, H-5'); ^{13}C-APT NMR (101 MHz, $CDCl_3$, HSQC): δ 170.2 (C=O CO_2Me), 139.2, 138.7, 138.5, 138.1, 137.7 (C_q), 128.6, 128.5, 128.4, 128.3, 128.2, 128.2, 128.1, 127.7, 127.7, 127.6, 127.4, 126.2 (CH_{arom}), 102.5 (H-1'), 101.5 (CHPh), 98.9 (C-1), 80.2 (C-3/C-5), 79.8 (C-3/C-5), 78.7 (C-4'), 78.5 (H-2, H-3'), 77.9 (C-2'), 77.2 (CH_2 Bn), 75.6 (CH_2 Bn), 75.2 (CH_2 Bn), 74.0 (CH_2 Bn), 72.7 (CH_2 Bn), 69.7 (C-6), 68.6 (C-6'), 67.7 (C-5'), 56.0 (CH_3 CO_2Me) , 52.5 (CH_3 OMe); ^{13}C-GATED NMR (101 MHz, $CDCl_3$): δ 102.5 ($J_{C1,H1}$ = 157 Hz, C-1 β); Diagnostic peaks α-anomer: ^1H NMR (400 MHz, $CDCl_3$, HH-COSY, HSQC): δ 5.58 (s, 0.10H, C*H*Ph), 5.27 (s, 0.10H, H-1'), 4.98 (d, 0.10H, J = 11.4 Hz, C*H*H Bn), 4.31 (d, 0.10H, J = 11.9 Hz, CH*H* Bn); ^{13}C-APT NMR (101 MHz, $CDCl_3$): δ 101.54 (CHPh), 100.45 (C-1'), 98.63 (C-1); HRMS: $[M+Na]^+$ calcd for $C_{49}H_{52}O_{12}Na$ 855.33510, found 855.33507.

Methyl 4-*O*-(2,3-di-*O*-benzyl-4,6-*O*-benzylidene-β-D-mannopyranosyl)-2,3,6-tri-*O*-benzyl-β-D-galactopyranoside (23).

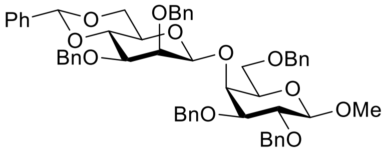

Donor **1** and acceptor **13** were condensed using the general procedure for Tf_2O/Ph_2SO mediated glycosylations (for an additional 16 hours at -40°C) and purified by flash column chromatography (9/1 to 7/3 pentane/EtOAc) to yield glycosylation product **23** (62.7 mg, 70 µmol, 70%, α:β = < 1:20). R_f: 0.80 (7/3 pentane/EtOAc); $[\alpha]_D^{26}$ = -26.8° (*c* = 1, $CHCl_3$); IR (neat): 737, 1072, 1454, 2866; Data for the β-anomer: ^{1}H NMR (400 MHz, $CDCl_3$, HH-COSY, HSQC): δ 7.46 – 7.14 (m, 30H, CH_{arom}), 5.59 (s, 1H, C*H*Ph), 4.96 (d, 1H, *J* = 12.4 Hz, C*H*H Bn), 4.91 (d, 1H, *J* = 11.0 Hz, C*H*H Bn), 4.86 (d, 1H, *J* = 12.4 Hz, CH*H* Bn), 4.79 (s, 1H, H-1'), 4.78 (d, 1H, *J* = 11.6 Hz, C*H*H Bn), 4.68 (d, 1H, *J* = 11.0 Hz, CH*H* Bn), 4.62 – 4.47 (m, 5H, CH*H* Bn, C*H*H Bn, CH*H* Bn, C*H*H Bn, CH*H* Bn), 4.31 (d, 1H, *J* = 7.7 Hz, H-1), 4.21 – 4.09 (m, 3H, H-4', H-6'), 4.01 (d, 1H, *J* = 3.0 Hz, H-2'), 3.90 – 3.81 (m, 2H, H-6, H-6'), 3.72 (dd, 1H, *J* = 9.8, 5.7 Hz, H-6), 3.67 (dd, 1H, *J* = 9.6, 7.7 Hz, H-2), 3.63 – 3.55 (m, 4H, H-5, CH_3 OMe), 3.52 (dd, 1H, *J* = 9.6, 3.0 Hz, H-3), 3.40 (dd, 1H, *J* = 9.9, 3.1 Hz, H-3'), 3.18 (td, 1H, *J* = 9.8, 4.9 Hz, H-5); ^{13}C-APT NMR (101 MHz, $CDCl_3$, HSQC): δ 138.9, 138.8, 138.5, 138.4, 138.2, 137.6 (C_q), 129.0, 128.7, 128.7, 128.5, 128.4, 128.4, 128.3, 128.3, 128.2, 128.0, 127.8, 127.7, 127.6, 127.6, 127.5, 126.1 (CH_{arom}), 105.1 (C-1), 102.6 (C-1'), 101.4 (CHPh), 81.8 (H-3), 79.5 (H-2), 78.5 (C-3'), 78.5 (C-4'), 75.4 (C-2'), 75.1 (CH_2 Bn), 74.7 (CH_2 Bn), 73.7 (CH_2 Bn), 73.6 (H-5), 73.6 (CH_2 Bn), 73.3 (C-4), 72.2(CH_2 Bn), 69.5 (C-6), 68.7 (C-6'), 67.8 (C-5), 57.2 (CH_3 OMe); ^{13}C-GATED NMR (101 MHz, $CDCl_3$): δ 102.6 ($J_{C1,H1}$ = 159 Hz, C-1 β); HRMS: $[M+Na]^+$ calcd for $C_{55}H_{58}O_{11}Na$ 917.38713, found 917.38696.

Methyl 2-*O*-(2,3-di-*O*-benzyl-4,6-*O*-benzylidene-β-D-mannopyranosyl)-3-*O*-benzyl-4,6-*O*-benzylidene-α-D-mannopyranoside (24).

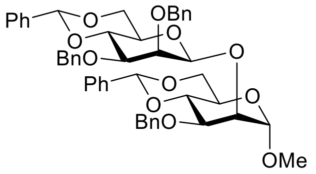

Donor **1** and acceptor **14** were condensed using the general procedure for Tf_2O/Ph_2SO mediated glycosylations (for an additional 16 hours at -40°C) and purified by flash column chromatography (9/1 to 7/3 pentane/EtOAc) to yield glycosylation product **24** (70.2 mg, 87 µmol, 87%, α:β = < 1:20). R_f: 0.68 (7/3 pentane/EtOAc). Spectroscopic data were in accord with those previously reported.[36–39] $[\alpha]_D^{26}$ –44.4° (*c* = 1, $CHCl_3$); (lit:[38] $[\alpha]_D^{25}$ = –44.2° (*c* = 4.2, $CHCl_3$), lit:[39] $[\alpha]_D^{20}$ = –44.8° (*c* = 3.9, $CHCl_3$)); IR (neat): 733, 1002, 1028, 1055, 1083, 1452, 2862; Data for the β-anomer: ^{1}H NMR (400 MHz, $CDCl_3$, HH-COSY, HSQC): δ 7.53 – 7.16 (m, 25H, CH_{arom}), 5.60 (s, 1H, C*H*Ph), 5.51 (s, 1H, C*H*Ph), 5.06 (d, 1H, *J* = 12.3 Hz, C*H*H Bn), 4.97 (d, 1H, *J* = 12.3 Hz, CH*H* Bn), 4.81 – 4.56 (m, 6H, C*H*H Bn, CH*H* Bn, C*H*H Bn, CH*H* Bn, H-1, H-1'), 4.30 – 4.18 (m, 4H, H-2, H-4´, H-6, H-6'), 4.10 (t, 1H, *J* = 9.2 Hz, H-4), 3.98 (d, 1H, *J* = 2.8 Hz, H-2'), 3.94 (dd, 1H, *J* = 10.0, 3.2 Hz, H-3) 3.88 (t, 1H, *J* = 10.3 Hz, H-6'), 3.78 (m, 2H, H-5, H-6), 3.59 (dd, 1H, *J* = 9.9, 3.0 Hz, H-3'), 3.46 – 3.21 (m, 4H, H-5', CH_3 OMe); ^{13}C-APT NMR (101 MHz, $CDCl_3$, HSQC): δ δ 139.0, 138.7, 138.5, 137.7, 137.7 (C_q), 129.0, 128.7, 128.4, 128.3, 128.3, 128.2, 127.7, 127.6, 127.6, 127.4, 126.2, 126.2 (CH_{arom}), 101.7 (CHPh), 101.5 (CHPh), 101.0 (C-1'), 99.6 (C-1), 78.8 (C-4), 78.6 (H-4'), 77.8 (C-3'), 76.1 (C-2'), 75.3 (H-2), 74.7 (H-3), 74.2 (CH_2 Bn), 72.4 (CH_2 Bn), 71.5 (CH_2 Bn), 69.1 (C-6), 68.7 (C-6'), 67.9 (C-5'), 64.2 (C-5), 55.1 (CH_3 OMe); ^{13}C-GATED NMR (101 MHz, $CDCl_3$): δ 101.0 ($J_{C1,H1}$ = 154 Hz, C-1' β); HRMS: $[M+Na]^+$ calcd for $C_{48}H_{50}O_{11}Na$ 825.32453, found 825.32425.

Glycosylations with donor 2.

Cyclohexyl 2,3-di-*O*-benzyl-4,6-*O*-benzylidene- α/β-D-glucopyranoside (2A)

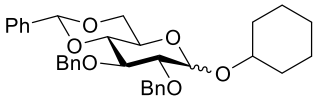

Donor **2** and cyclohexanol were condensed using the general procedure for Tf_2O/Ph_2SO mediated glycosylations and purified by flash column chromatography (1/0 to 0/1 pentane/toluene to 6% EtOAc in toluene) to yield glycosylation product **2A** (37.8 mg, 71 µmol, 71%, α:β = 1:5). R_f: 0.22 (toluene). Spectroscopic data were in accord with those previously reported.[40] IR (neat): 696, 735, 746, 997, 1028, 1049, 1072, 1366, 1452, 1497, 2857, 2930; Data for the β-anomer:^{1}H NMR ($CDCl_3$, 400 MHz, HH-COSY, HSQC): δ 7.51 – 7.46 (m, 2H, CH_{arom}), 7.41 – 7.24 (m, 13H, CH_{arom}), 5.56 (s, 1H, C*H*Ph), 4.94 (d, 1H, *J* = 10.8 Hz, C*H*H Bn), 4.90 (d, 1H, *J* = 11.1 Hz, C*H*H Bn), 4.79 (d, 1H, *J* = 11.5 Hz, CH*H* Bn), 4.76 (d, 1H, *J* = 10.9 Hz, CH*H* Bn), 4.62 (d, 1H, *J* = 7.7 Hz, H-1), 4.33 (dd, 1H, *J* = 10.5, 5.0 Hz, H-6), 3.79 (t, 1H, *J* = 10.3 Hz, H-6), 3.76 – 3.65 (m, 3H, C*H* Cy, H-3, H-4), 3.46 (t, 1H, *J* = 8.1 Hz, H-2), 3.39 (td, 1H, *J* = 9.5, 5.0 Hz, H-5), 2.00 – 1.91 (m, 2H, CH_2 Cy), 1.82 – 1.72 (m, 2H, CH_2 Cy), 1.59 – 1.18 (m, 6H, CH_2 Cy); ^{13}C-APT NMR ($CDCl_3$, 101 MHz, HSQC): δ 138.7, 138.6, 137.5 (C_q), 129.0, 128.4, 128.4, 128.3, 128.3, 128.1, 127.8, 127.7, 126.1 (CH_{arom}), 102.5 (C-1), 101.2 (CHPh), 82.3 (C-2), 81.6, 81.2 (C-3, C-4), 78.3 (CH Cy), 75.5, 75.2 (CH_2 Bn), 69.0 (C-6), 66.1 (C-5), 33.9, 32.1, 25.7, 24.2, 24.1 (CH_2 Cy); Diagnostic peaks α-anomer: ^{1}H NMR ($CDCl_3$, 400 MHz, HH-COSY, HSQC): δ 4.69 (d, 1H, *J* = 12.1 Hz, CH*H* Bn), 4.26 (dd, 1H, *J* = 10.2, 4.9 Hz, H-6), 4.07 (t, 1H, *J* = 9.3 Hz, H-3), 3.96 (td, 1H, *J* = 10.0, 4.9 Hz, H-5), 3.61 (t, 1H, *J* = 9.4 Hz, H-4), 3.58 – 3.50 (m, 2H, CH Cy, H-2); ^{13}C-APT NMR ($CDCl_3$, 101 MHz, HSQC): δ 101.3 (CHPh), 96.1 (C-1), 82.5 (C-4), 79.5 (C-2) 78.8 (C-3), 76.1 (CH Cy), 75.4, 73.4 (CH_2 Bn), 69.2 (C-6), 62.6 (C-5); HRMS: $[M+H]^+$ calcd for $C_{33}H_{39}O_6$ 531.27412, found 531.27400.

Ethyl 2,3-di-*O*-benzyl-4,6-*O*-benzylidene-α/β-D-glucopyranoside (2B)

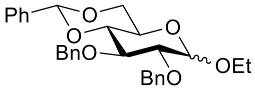

Donor **2** and ethanol were condensed using the general procedure for Tf_2O/Ph_2SO mediated glycosylations and purified by flash column chromatography (1/1 to 0/1 pentane/toluene to 6% EtOAc in toluene) to yield glycosylation product **2B** (32.2 mg, 68 µmol, 68%, α:β = 1:10). R_f: 0.43 (6% EtOAc in toluene). IR (neat): 692, 743, 1006, 1028, 1183, 1364, 1453, 2872; Data for the β-anomer: ^{1}H NMR ($CDCl_3$, 400 MHz, HH-COSY, HSQC): δ 7.52 – 7.46 (m, 2H, CH_{arom}), 7.41 – 7.24 (m, 13H, CH_{arom}), 5.56 (s, 1H, C*H*Ph), 4.93 – 4.88 (m, 2H, 2xC*H*H Bn), 4.83 – 4.74 (m, 2H, 2xCH*H* Bn), 4.51 (d, 1H, *J* = 7.7 Hz, H-1), 4.34 (dd, 1H, *J* = 10.5, 5.0 Hz, H-6), 3.97 (dq, 1H, *J* = 9.6, 7.1 Hz, C*H*H Et), 3.79 (t, 1H, *J* = 9.5 Hz, H-6), 3.76 – 3.63 (m, 3H, H-3, H-4, CH*H* Et), 3.46 (t, 1H, *J* = 8.1 Hz, H-2), 3.40 (ddd, 1H, *J* = 10.0, 9.0, 5.0 Hz, H-5), 1.29 (t, 3H, *J* = 7.0 Hz, CH_3 Et); ^{13}C-APT NMR ($CDCl_3$, 101 MHz, HSQC): δ 138.7, 138.6, 137.5 (C_q), 129.0, 128.5, 128.4, 128.4, 128.2, 128.1, 127.8, 127.7, 126.1 (CH_{arom}), 104.1 (C-1), 101.3 (CHPh), 82.3 (C-2), 81.7 (C-4), 81.0 (C-3), 75.5, 75.2 (CH_2 Bn), 69.0 (C-6), 66.2 (CH_2 Et), 66.2 (C-5), 15.5 (CH_3 Et); Diagnostic peaks α-anomer: ^{1}H NMR ($CDCl_3$, 400 MHz, HH-COSY, HSQC): δ 5.55 (s, 1H, C*H*Ph), 4.92 (d, 1H, *J* = 11.2 Hz, C*H*H Bn), 4.86 – 4.83 (m, 2H, C*H*H Bn, CH*H* Bn), 4.73 (d, 1H, *J* = 3.8 Hz, H-1), 4.68 (d, 1H, *J* = 12.2 Hz, CH*H* Bn), 4.25 (dd, 1H, *J* = 10.2, 4.8 Hz, H-6), 4.06 (t, 1H, *J* = 9.3 Hz, H-3), 3.88 (td, 1H, *J* = 10.0, 4.8 Hz, H-5), 3.63 – 3.60 (m, 1H, H-4), 3.59 – 3.52 (m, 2H, H-2, CH*H* Et); ^{13}C-APT NMR ($CDCl_3$, 101 MHz, HSQC): δ 101.3 (CHPh), 97.9 (C-1), 82.4 (C-4), 79.5 (C-2), 78.8 (C-3), 73.7 (CH_2 Bn), 69.2 (C-6), 63.8 (CH_2 Et), 62.5 (C-5); HRMS: $[M+H]^+$ calcd for $C_{29}H_{33}O_6$ 477.22717, found 477.22699.

2-Fluoroethyl 2,3-di-*O*-benzyl-4,6-*O*-benzylidene-α/β-D-glucopyranoside (2C)

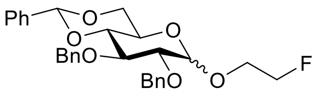

Donor **2** and 2-fluoroethanol were condensed using the general procedure for Tf_2O/Ph_2SO mediated glycosylations and purified by flash column chromatography (1/1 to 0/1 pentane/toluene to 6% EtOAc in toluene) to yield glycosylation product **2C** (34.7 mg, 70 µmol, 70%, α:β = 1:3). R_f: 0.30 and 0.34 (4% EtOAc in toluene). IR (neat): 695, 744, 1000, 1028, 1072, 1085, 1177, 1452, 2868. Reported as a 0.33 : 1.00 mixture of anomers: ^{1}H NMR ($CDCl_3$, 400 MHz, HH-COSY, HSQC): δ 7.52 – 7.44 (m, 2.66H, CH_{arom}), 7.42 – 7.24 (m, 17.29H, CH_{arom}), 5.56 (s, 1H, C*H*$Ph_β$), 5.55 (s, 0.33H, C*H*$Ph_α$), 4.92 (dd, 2.33H, *J* = 11.1, 3.4 Hz, 2xC*H*H $Bn_β$, C*H*H $Bn_α$), 4.87 – 4.73 (m, 2.99H, 2xCH*H* $Bn_β$, CH*H* $Bn_α$, C*H*H $Bn_α$, H-$1_α$), 4.72 – 4.60 (m, 1.66H, CH*H* $Bn_α$, C*H*H-$CH_2F_α$, C*H*H-$CH_2F_β$), 4.59 – 4.49 (m, 2.33H, CH*H*-$CH_2F_α$, CH*H*-$CH_2F_β$, H-$1_β$), 4.34 (dd, 1H, *J* = 10.5, 5.0 Hz, H-$6_β$), 4.26 (dd, 0.33H, *J* = 10.2, 4.9 Hz, H-$6_α$), 4.13 (ddd, 0.50H, *J* = 12.1, 4.7, 2.6 Hz, C*H*$HF_β$), 4.10 – 4.02 (m, 0.83H, C*H*$HF_β$, H-$3_α$), 3.94 – 3.66 (m, 5.32H, CH*H*$F_β$, C*H2*$F_α$, H-$3_β$, H-$4_β$, H-$5_α$, H-$6_α$, H-$6_β$), 3.61 (t, 0.33H, *J* = 9.4 Hz, H-$4_α$), 3.58 (dd, 0.33H, *J* = 9.3, 3.8 Hz, H-$2_α$), 3.50 (t, 1H, *J* = 8.1 Hz, H-$2_β$), 3.41 (ddd, 1H, *J* = 10.0, 9.0, 5.0 Hz, H-$5_β$); ^{13}C-APT NMR ($CDCl_3$, 101 MHz, HSQC): δ 138.9, 138.6, 138.4, 138.3, 137.5, 137.4 (C_q), 129.1, 129.0, 128.6, 128.5, 128.4, 128.4, 128.3, 128.2, 128.1, 128.1, 128.0, 127.7, 126.1 (CH_{arom}), 104.4 (C-$1_β$, 101.4 ($CHPh_α$), 101.3 ($CHPh_β$), 98.4 (C-$1_α$), 82.7 (d, *J* = 170.2 Hz, $CH_2F_α$), 82.6 (d, *J* = 170.2 Hz, $CH_2F_β$), 82.2 (C-$4_α$), 82.1 (C-$2_β$), 81.5 (C-$4_β$), 80.9 (C-$3_β$), 79.4 (C-$2_α$), 78.6 (C-$3_α$), 75.5 (CH_2 $Bn_{α,β}$), 75.2 (CH_2 $Bn_β$), 73.7 (CH_2 $Bn_α$), 69.4 (d, *J* = 20.0 Hz, *CH2*-$CH_2F_β$), 69.1 (C-$6_α$), 68.8 (C-6), 67.3 (d, *J* = 20.2 Hz, *CH2*-$CH_2F_α$), 66.2 (C-$5_β$), 62.6 (C-$5_α$); HRMS: $[M+H]^+$ calcd for $C_{29}H_{32}FO_6$ 495.21774, found 495.21745.

2,2-Difluoroethyl 2,3-di-*O*-benzyl-4,6-*O*-benzylidene-α/β-D-glucopyranoside (2D)

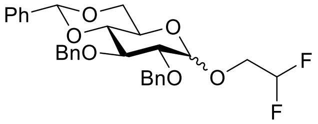

Donor **2** and 2,2-difluoroethanol were condensed using the general procedure for Tf_2O/Ph_2SO mediated glycosylations and purified by flash column chromatography (1/1 to 0/1 pentane/toluene to 6% EtOAc in toluene) to yield glycosylation product **2D** (36 mg, 70 µmol, 70%, α:β = 5:1). R_f: 0.32 and 0.36 (4% EtOAc in toluene). IR (neat): 696, 747, 996, 1028, 1071, 1086, 1369, 1453, 2865. Data for the α-anomer: ^{1}H NMR ($CDCl_3$, 400 MHz, HH-COSY, HSQC): δ 7.48 (m, 2H, CH_{arom}), 7.41 – 7.26 (m, 13H, CH_{arom}), 5.95 (tt, 1H, *J* = 55.4, 4.2 Hz, C*H*F_2), 5.55 (s, 1H, C*H*Ph), 4.92 (d, 1H, *J* = 11.3 Hz, C*H*H Bn), 4.84 (d, 1H, *J* = 12.0 Hz, C*H*H Bn), 4.83 (d, 1H, *J* = 11.4 Hz, CH*H* Bn), 4.75 (d, 1H, *J* = 3.9 Hz, H-1), 4.66 (d, 1H, *J* = 12.0 Hz, CH*H* Bn), 4.25 (dd, 1H, *J* = 10.2, 4.8 Hz, H-6), 4.03 (t, 1H, *J* = 9.3 Hz, H-3), 3.90 – 3.65 (m, 4H, C*H2*-CHF_2, H-5, H-6), 3.62 (t, 1H, *J* = 9.4 Hz, H-4), 3.58 (dd, 1H, *J* = 9.3, 3.8 Hz, H-2); ^{13}C-APT NMR ($CDCl_3$, 101 MHz, HSQC): δ 138.8, 138.2, 137.4 (C_q), 129.1, 128.6, 128.4, 128.4, 128.2, 128.1, 128.1, 127.7, 126.1 (CH_{arom}), 114.2 (t, *J* = 241.5 Hz, CHF_2), 101.4 (CHPh), 98.9 (C-1), 82.0 (C-4), 79.3 (C-2), 78.4 (C-3), 75.5, 74.0 (CH_2 Bn), 69.0 (C-6), 67.4 (t, *J* = 28.8 Hz, *CH2*-CHF_2), 63.0 (C-5); Diagnostic peaks β-anomer: ^{1}H NMR ($CDCl_3$, 400 MHz, HH-COSY, HSQC): δ 5.90 (tdd, 1H, *J* = 55.4, 5.0, 3.4 Hz, C*H*F_2), 5.56 (s, 1H, C*H*Ph), 4.54 (d, 1H, *J* = 7.6 Hz, H-1), 4.34 (dd, 1H, *J* = 10.5, 5.0 Hz, H-6), 3.48 (t, 1H, *J* = 8.1 Hz, H-2), 3.41 (td, 1H, *J* = 9.6, 5.0 Hz, H-5); ^{13}C-APT NMR ($CDCl_3$, 101 MHz, HSQC): δ 104.5 (C-1), 101.3 (CHPh), 81.9 (c-2), 81.4, 80.8 (C-3, C-4), 75.6, 75.3 (CH_2 Bn), 68.7 (C-6), 66.3 (C-5); HRMS: $[M+H]^+$ calcd for $C_{29}H_{31}F_2O_6$ 513.20832, found 513.20808.

2,2,2-Trifluoroethyl 2,3-di-*O*-benzyl-4,6-*O*-benzylidene-α-D-glucopyranoside (2E)

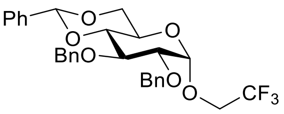

Donor **2** and 2,2,2-trifluoroethanol were condensed using the general procedure for Tf_2O/Ph_2SO mediated glycosylations and purified by flash column chromatography (1/1 to 0/1 pentane/toluene to 6% EtOAc in toluene) to yield glycosylation product **2E** (33.7 mg, 64 µmol, 64%, α:β = > 20:1). R_f: 0.45 (4% EtOAc in toluene). $[\alpha]_D^{23}$ = +7.0° (*c* = 0.67, DCM); IR (neat): 697, 747, 1001, 1029, 1077, 1161, 1279, 1373, 1454, 2864; Data for the α-anomer: ^{1}H NMR ($CDCl_3$, 400 MHz, HH-COSY, HSQC): δ 7.51 – 7.46 (m, 2H, CH_{arom}), 7.41 – 7.26 (m, 13H, CH_{arom}), 5.55 (s, 1H, C*H*Ph), 4.92 (d, 1H, *J* = 11.2 Hz, C*H*H Bn), 4.84 (d, 1H, *J* = 12.0 Hz, C*H*H Bn), 4.83 (d, 1H, *J* = 11.3 Hz, CH*H* Bn), 4.80 (d, 1H, *J* = 3.9 Hz, H-1), 4.67 (d, 1H, *J* = 12.0 Hz, CH*H* Bn), 4.25 (dd, 1H, *J* = 10.2, 4.8 Hz, H-6), 4.05 (t, 1H, *J* = 9.3 Hz, H-3), 3.92 (q, 2H, *J* = 8.7 Hz, C*H2*-CF3), 3.85 (td, 1H, *J* = 9.9, 4.8 Hz, H-5), 3.70 (t, 1H, *J* = 10.3 Hz, H-6), 3.63 (t, 1H, *J* = 9.4 Hz, H-4), 3.59 (dd, 1H, *J* = 9.3, 3.8 Hz, H-2); ^{13}C-APT NMR ($CDCl_3$, 101 MHz, HSQC): δ 138.8, 138.2, 137.4 (C_q), 129.1, 128.6, 128.4, 128.4, 128.2, 128.1, 128.1, 127.8, 126.2 (CH_{arom}), 123.8 (q, *J* = 278.6 Hz, CF_3), 101.4 (CHPh), 99.0 (C-1), 81.9 (C-4), 79.2 (C-2), 78.3 (C-3), 75.5, 73.9 (CH_2 Bn), 68.9 (C-6), 65.2 (q, *J* = 35.0 Hz, *CH2*-CF3), 63.3 (C-5); Diagnostic peaks β-anomer: ^{1}H NMR ($CDCl_3$, 400 MHz, HH-COSY, HSQC): δ 5.56 (s, 1H, C*H*Ph), 4.73 (d, 1H, *J* = 10.7 Hz, CH*H* Bn), 4.60 (d, 1H, *J* = 7.7 Hz, H-1), 4.34 (dd, 1H, *J* = 10.5, 5.0 Hz, H-6), 3.51 (t, 1H, *J* = 8.0 Hz), 3.41 (td, 1H, *J* = 9.6, 5.2 Hz, H-5); HRMS: $[M+H]^+$ calcd for $C_{29}H_{30}F_3O_6$ 531.19890, found 531.19857.

1,1,1,3,3,3-Hexafluoro-2-propyl 2,3-di-*O*-benzyl-4,6-*O*-benzylidene-α-D-glucopyranoside (2F)

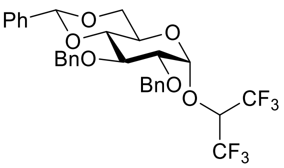

Donor **2** and 1,1,1,3,3,3-hexafluoroisopropanol were condensed using the general procedure for Tf_2O/Ph_2SO mediated glycosylations (for an additional 144 hours at -40°C) and purified by flash column chromatography (1/1 to 0/1 pentane/toluene to 10% EtOAc in toluene) to yield glycosylation product **2F** (39 mg, 65 µmol, 65%, α:β = > 20:1). R_f: 0.31 (4/1 pentane/Et_2O). $[\alpha]_D^{25}$ = -40.9° (*c* = 0.68, $CHCl_3$); IR (neat): 689, 746, 997, 1086, 1196, 1219, 1368, 1454, 2868; ^{1}H NMR ($CDCl_3$, 400 MHz, HH-COSY, HSQC): δ 7.51 – 7.47 (m, 2H, CH_{arom}), 7.40 – 7.27 (m, 13H, CH_{arom}), 5.55 (s, 1H, C*H*Ph), 5.07 (d, 1H, *J* = 4.0 Hz, H-1), 4.93 (d, 1H, *J* = 11.1 Hz, C*H*H Bn), 4.83 (d, 1H, *J* = 11.1 Hz, CH*H* Bn), 4.79 (d, 1H, *J* = 11.7 Hz, C*H*H Bn), 4.73 (d, 1H, *J* = 11.7 Hz, CH*H* Bn), 4.41 (hept, 1H, *J* = 5.9 Hz, CH HFIP), 4.24 (dd, 1H, *J* = 10.2, 5.0 Hz, H-6), 4.06 (t, 1H, *J* = 9.4 Hz, H-3), 3.94 (td, 1H, *J* = 10.0, 4.9 Hz, H-5), 3.70 (t, 1H, *J* = 10.2 Hz, H-6), 3.75 – 3.60 (m, 2H, H-2, H-4); ^{13}C-APT NMR ($CDCl_3$, 101 MHz, HSQC): δ 138.6, 137.7, 137.2 (C_q), 129.2, 128.6, 128.5, 128.4, 128.2, 128.1, 127.8, 126.1 (CH_{arom}), 121.7 (q, *J* = 285 Hz, CF_3), 121.2 (q, *J* = 285 Hz, CF_3), 101.4 (CHPh), 100.4 (C-1), 81.5 (C-4), 78.3, 78.3 (C-2, C-3), 75.6, 74.1 (CH_2 Bn), 73.4 (hept, *J* = 32.9 Hz, CH HFIP), 68.5 (C-6), 64.0 (C-5); ^{13}C-HMBC NMR ($CDCl_3$, 101 MHz): $^3J(H_{HFIP}$-C1) observed; HRMS: $[2M-2(CF_3)_2CHO+H_2O+NH_4]^+$ calcd for $(C_{27}H_{27}O_5)_2O$ 896.40044, found 896.40115; LC-MS: Rt = 10.09, no conclusive mass. TLC-MS: $[M+Na]^+$ calcd for $C_{30}H_{28}F_6O_6Na$ 621.17 found 621.2, and $[M+H_2O\text{-benzaldehyde}+Na]^+$ calcd for $C_{23}H_{24}F_6O_6Na$ 533.14 found 533.0.

1-[^{2}H]-1,5-anhydro-2,3-di-*O*-benzyl-4,6-*O*-benzylidene-α-D-glucitol (2G)

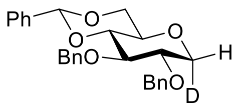

Donor **2** and triethylsilane-D were condensed using the general procedure for Tf_2O/Ph_2SO mediated glycosylations (for an additional 144 hours at -40°C) and purified by flash column chromatography (19/1 to 4/1 Et_2O/pentane) to yield glycosylation product **2G** (34 mg, 79 µmol, 79%, α:β = > 20:1). R*f*: 0.38 (4/1 pentane/Et_2O). Spectroscopic data of the non-dueterated glucitol were in accord with those previously reported.[41] $[\alpha]_D^{23}$ = +5.4° (*c* = 0.78, $CHCl_3$); IR (neat): 696, 748, 1009, 1028, 1088, 1368, 1454, 2868; ^{1}H NMR ($CDCl_3$, 400 MHz, HH-COSY, HSQC): δ 7.51 – 7.48 (m, 2H, CH_{arom}), 7.41 – 7.34 (m, 5H, CH_{arom}), 7.34 – 7.26 (m, 7H, CH_{arom}), 5.55 (s, 1H, C*H*Ph), 4.96 (d, 1H, *J* = 11.4 Hz, C*H*H Bn), 4.83 (d, 1H, *J* = 11.6 Hz, CH*H* Bn), 4.80 (d, 1H, *J* = 11.7 Hz, C*H*H Bn), 4.66 (d, 1H, *J* = 11.6 Hz, CH*H* Bn), 4.31 (dd, 1H, *J* = 10.4, 5.0 Hz, H-6), 3.98 (d, 1H, *J* = 5.6 Hz, H-1), 3.75 (t, 1H, *J* = 8.8 Hz, H-3), 3.70 – 3.63 (m, 2H, H-2, H-6), 3.61 (t, 1H, *J* = 9.2 Hz, H-4), 3.36 (ddd, 1H, *J* = 10.1, 9.2, 5.0 Hz, H-5); ^{13}C-APT NMR ($CDCl_3$, 101 MHz, HSQC): δ 138.8, 138.3, 137.5 (C_q), 129.0, 128.6, 128.4, 128.4, 128.1, 128.0, 128.0, 127.7, 126.1 (CH_{arom}), 101.3 (CHPh), 82.5 (C-3), 82.2 (C-4), 77.7 (C-2, 75.1, 74.0 (CH_2 Bn), 71.4 (C-5), 69.0 (C-6), 68.7 (t, $J_{C1,D1}$ = 22.3 Hz); ^{2}H NMR ($CHCl_3$, 61 MHz): 3.34 (s, 1D, D-1); HRMS: $[M+H]^+$ calcd for $C_{27}H_{28}DO_5S$ 434.20723, found 434.20714.

Allyl 2,3-di-*O*-benzyl-1-deoxy-4,6-*O*-benzylidene-α-D-glucopyranoside (2H)

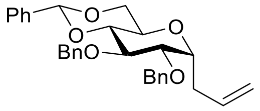

Donor **2** and allyl trimethylsilane were condensed using the general procedure for Tf_2O/Ph_2SO mediated glycosylations (for an additional 16 hours at -40°C) and purified by flash column chromatography (19/1 to 9/1 pentane/EtOAc) to yield glycosylation product **X12** (20 mg, 42 µmol, 42%, α:β = > 1:20). Contaminated with a 1-OTMS glycoside by-product. α-Thio glycoside **2*α*** was formed as a by-product. R*f*: 0.60 (9/1 pentane/EtOAc). Spectroscopic data were in accord with those previously reported.[35,42] ^{1}H NMR ($CDCl_3$, 400 MHz, HH-COSY, HH-NOESY, HSQC): δ 7.53 – 7.47 (m, 2H, CH_{arom}), 7.42 – 7.27 (m, 13H, CH_{arom}), 5.77 (ddt, 1H, *J* = 17.1, 10.2, 6.9 Hz, C*H* allyl), 5.57 (s, 1H, C*H*Ph), 5.18 – 5.05 (m, 2H, CH_2 allyl), 4.93 (d, 1H, *J* = 11.4 Hz, C*H*H Bn), 4.81 (d, 1H, *J* = 11.4 Hz, CH*H* Bn), 4.78 (d, 1H, *J* = 11.7 Hz, C*H*H Bn), 4.64 (d, 1H, *J* = 11.7 Hz, CH*H* Bn), 4.27 – 4.21 (m, 1H, H-6), 4.08 (td, 1H, *J* = 7.7, 5.7 Hz, H-1), 3.92 – 3.85 (m, 1H, H-3), 3.76 (dd, 1H, *J* = 8.6, 5.7 Hz, H-2), 3.69 – 3.63 (m, 3H, H-4, H-5, H-6), 2.57 – 2.51 (m, 2H, CH_2 allylic); ^{13}C-APT NMR ($CDCl_3$, 101 MHz, HSQC, HMBC): δ 138.7, 138.3, 137.5 ($C_{q\text{-}arom}$), 134.4 (CH allyl), 129.0, 128.6, 128.5, 128.4, 128.1, 128.0, 127.9, 127.8, 126.1 (CH_{arom}), 117.4 (CH_2 allyl), 101.3 (CHPh), 82.9 (C-4), 79.5 (C-2), 78.9 (C-3), 75.0 (C-1), 75.0, 73.7 (CH_2 Bn), 69.6 (C-6), 63.5 (C-5), 30.8 (CH_2 allylic); HRMS: $[M+H]^+$ calcd for $C_{30}H_{33}O_5$ 473.23225, found 473.23228.

Phenyl 2,3-di-*O*-benzyl-4,6-*O*-benzylidene-1-thio-α-D-glucopyranoside (2*α*)

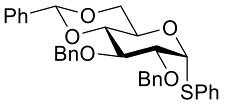

R*f*: 0.38 (4/1 pentane/Et_2O). Spectroscopic data were in accord with those previously reported.[16] ^{1}H NMR ($CDCl_3$, 400 MHz, HH-COSY, HSQC): δ 7.53 – 7.44 (m, 4H, CH_{arom}), 7.43 – 7.36 (m, 7H, CH_{arom}), 7.36 – 7.27 (m, 9H, CH_{arom}), 5.59 (d, 1H, *J* =

5.5 Hz, H-1), 5.57 (s, 1H, C*H*Ph), 4.92 (d, 1H, *J* = 11.3 Hz, CH*H* Bn), 4.86 (d, 1H, *J* = 11.3 Hz, CH*H* Bn), 4.81 (d, 1H, *J* = 11.8 Hz, C*H*H Bn), 4.76 (d, 1H, *J* = 11.8 Hz, CH*H* Bn), 4.39 (td, 1H, *J* = 9.9, 4.9 Hz, H-5), 4.19 (dd, 1H, *J* = 10.3, 5.0 Hz, H-6), 3.98 (t, 1H, *J* = 9.2 Hz, H-3), 3.90 (dd, 1H, *J* = 9.3, 5.5 Hz, H-2), 3.71 (t, *J* = 10.3 Hz, H-6), 3.66 (t, *J* = 9.3 Hz, H-4); HRMS: $[M+NH_4]^+$ calcd for $C_{33}H_{36}NO_5$ 558.23087, found 558.23075.

Methyl 6-*O*-(2,3-di-*O*-benzyl-4,6-*O*-benzylidene-α/β-ᴅ-glucopyranosyl)-2,3,4-tri-*O*-benzyl-α-ᴅ-glucopyranoside (25)

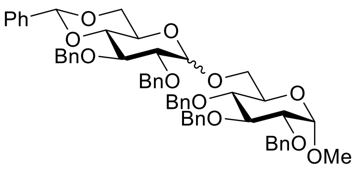

Donor **2** and acceptor **10** were condensed using the general procedure for Tf_2O/Ph_2SO mediated glycosylations (for an additional 16 hours at -40°C) and purified by flash column chromatography (9/1 to 3/1 pentane/EtOAc) to yield glycosylation product **25** (72.1 mg, 81 µmol, 81%, α:β = 1:2.7). R_f: 0.83 (6/4 pentane/EtOAc). Spectroscopic data were in accord with those previously reported.[36] ^{1}H NMR ($CDCl_3$, 400 MHz, HH-COSY, HSQC, HMBC): δ 7.51 – 7.44 (m, 2H, CH_{arom}), 7.41 – 7.12 (m, 28H, CH_{arom}), 5.54 (s, 1H, C*H*Ph), 4.97 (d, 1H, *J* = 10.8 Hz, C*H*H Bn), 4.93 – 4.88 (m, 2H, 2xC*H*H Bn), 4.84 – 4.76 (m, 4H, 3xCH*H* Bn, C*H*H Bn), 4.73 – 4.63 (m, 2H, C*H*H Bn, CH*H* Bn), 4.61 (d, 1H, *J* = 3.6 Hz, H-1), 4.49 (d, 1H, *J* = 11.2 Hz, CH*H* Bn), 4.44 (d, 1H, *J* = 7.7 Hz, H-1'), 4.31 (dd, 1H, *J* = 10.5, 5.0 Hz, H-6'), 4.11 (dd, 1H, *J* = 10.7, 2.0 Hz, H-6), 3.99 (t, *J* = 9.3 Hz, 1H, H-3), 3.82 – 3.65 (m, 5H, H-3', H-4', H-5, H-6, H-6'), 3.56 – 3.48 (m, 3H, H-2, H-2', H-4), 3.40 – 3.34 (m, 1H, H-5'), 3.33 (s, 3H, CH_3 OMe); ^{13}C-APT NMR ($CDCl_3$, 101 MHz, HSQC, HMBC): δ 138.9, 138.5, 138.4, 138.3, 138.2, 137.4 (C_q), 128.6, 128.5, 128.5, 128.4, 128.4, 128.4, 128.4, 128.3, 128.3, 128.3, 128.2, 128.1, 128.1, 128.1, 128.1, 128.0, 128.0, 128.0, 127.7, 127.7, 127.7, 127.7, 126.1 (CH_{arom}), 104.2 (C-1'), 101.2 (CHPh), 98.2 (C-1), 82.1 (C-3), 81.9 (C-2'), 81.5, 81.2 (C-3', C-4'), 79.8 (C-2), 77.9 (C-4), 75.8, 75.5, 75.2, 75.0, 73.5 (CH_2 Bn), 69.8 (C-5), 68.8 (C-6, C-6'), 66.2 (C-5'), 55.3 (OMe); Diagnostic peaks α-anomer: ^{1}H NMR ($CDCl_3$, 400 MHz): δ 5.53 (s, 0.33H), 4.57 (d, 0.33H, *J* = 3.6 Hz), 4.20 (dd, 0.33H, *J* = 10.1, 4.8 Hz), 3.89 (td, 0.33H, *J* = 10.0, 4.8 Hz), 3.43 (dd, 0.33H, *J* = 9.6, 3.6 Hz), 3.34 (s, 1H); ^{13}C-APT NMR ($CDCl_3$, 101 MHz): δ 138.9, 138.8, 138.5, 138.3, 137.6, 129.0-126.2, 101.4, 98.3, 98.1, 82.3, 82.2, 80.2, 79.4, 78.0, 77.8, 75.8, 75.1, 75.1, 73.5, 73.0, 70.5, 69.2, 66.4, 62.6, 55.3; HRMS: $[M+Na]^+$ calcd for $C_{55}H_{58}O_{11}Na$ 917.38713, found 917.38678.

Methyl 4-*O*-(2,3-di-*O*-benzyl-4,6-*O*-benzylidene-α/β-ᴅ-glucopyranosyl)-2,3,6-tri-*O*-benzyl-α-ᴅ-glucopyranoside (26)

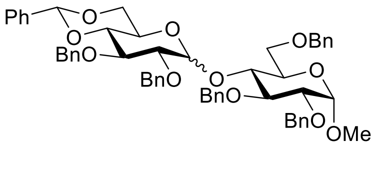

Donor **2** and acceptor **11** were condensed using the general procedure for Tf_2O/Ph_2SO mediated glycosylations (for an additional 16 hours at -40°C) and purified by flash column chromatography (19/1 to 4/1 pentane/EtOAc) to yield glycosylation product **26** (71 mg, 79 µmol, 79%, α:β = 1:1). R_f: 0.54 (4/1 pentane/EtOAc). Spectroscopic data were in accord with those previously reported for the α-anomer.[36] ^{1}H NMR ($CDCl_3$, 400 MHz, HH-COSY, HSQC, HMBC): δ 7.52 – 7.45 (m, 4H, CH_{arom}), 7.44 – 7.18 (m, 56H, CH_{arom}), 5.75 (d, 1H, *J* = 3.8 Hz, H-1'$_α$), 5.52 (s, 1H, C*H*Ph$_α$), 5.49 (s, 1H, C*H*Ph$_β$), 5.04 (d, 1H, *J* = 11.7 Hz, C*H*H Bn$_α$), 4.95 – 4.87 (m, 3H, 3xC*H*H Bn), 4.84 – 4.51 (m, 17H, 6xC*H*H Bn, 9xCH*H* Bn, H-1, H-1$_β$), 4.36 (d, 1H, *J* = 7.8 Hz, H-1'$_β$), 4.30 (d, 1H, *J* = 12.0 Hz, CH*H* Bn$_β$), 4.19 (dd, 1H, *J* = 10.5, 5.0 Hz, H-6'$_β$), 4.15 – 4.09 (m, 3H, H-3$_α$, H-4$_α$, H-6'$_α$), 3.99 (t, 1H, *J* = 9.3 Hz, H-3'$_α$), 3.94 (t, 1H, *J* = 9.4 Hz, H-4$_β$), 3.90 – 3.78 (m, 5H, H-2$_β$, H-5$_α$, H-5$_α$', H-6$_α$, H-6$_β$), 3.69 – 3.41 (m, 11H, H-2$_α$, H-2'$_α$, H-3$_β$, H-3'$_β$, H-4'$_α$, H-4'$_β$, H-5$_β$, H-6$_α$, H-6$_β$, H-6'$_α$, H-6'$_β$), 3.40 – 3.31 (m, 7H, CH_3 OMe,

CH_3 OMe$_{\beta}$, H-2'$_{\beta}$), 3.10 (td, 1H, *J* = 9.5, 4.9 Hz, H-5'$_{\beta}$); ^{13}C-APT NMR ($CDCl_3$, 101 MHz, HSQC, HMBC): δ 139.4, 139.0, 138.7, 138.6, 138.5, 138.4, 138.2, 138.0, 137.9, 137.9, 137.6, 137.5 (C_q), 129.0, 128.9, 128.6, 128.5, 128.5, 128.4, 128.3, 128.3, 128.3, 128.2, 128.2, 128.2, 128.1, 128.1, 128.0, 128.0, 127.9, 127.8, 127.7, 127.7, 127.5, 127.5, 127.4, 127.3, 126.8, 126.1, 126.1 (CH_{arom}), 102.9 (C-1'$_{\beta}$), 101.2 (CHPh), 98.5, 97.8 (C-1$_{\alpha}$, C-1$_{\beta}$), 97.2 (C-1'$_{\alpha}$), 82.7 (C-2'$_{\beta}$), 82.4 (C-4'$_{\alpha}$), 82.2 (C-3$_{\alpha}$), 81.8 (C-4'$_{\beta}$), 81.0 (C-3'$_{\beta}$), 80.3 (C-2$_{\beta}$), 80.3, 78.9 (C-2$_{\alpha}$, C-3'$_{\alpha}$), 78.8 (C-2'$_{\alpha}$, C-3$_{\beta}$), 76.9 (C-4$_{\beta}$), 75.6, 75.5, 75.4, 75.0, 74.4, 73.9, 73.7, 73.4, 73.4 (CH_2 Bn), 71.6 (C-4$_{\alpha}$), 70.0 (C-5$_{\beta}$), 69.4 (C-5$_{\alpha}$), 69.0, 68.9, 68.8 (C-6$_{\alpha}$, C-6'$_{\alpha}$, C-6'$_{\beta}$), 67.7 (C-6$_{\beta}$), 65.8 (C-5'$_{\beta}$), 63.4 (C-5'$_{\alpha}$), 55.5 (OMe$_{\beta}$), 55.3 (OMe$_{\alpha}$); HRMS: $[M+NH_4]^+$ calcd for $C_{55}H_{62}O_{11}N$ 912.43174, found 912.43282.

Methyl (methyl 4-*O*-[2,3-di-*O*-benzyl-4,6-*O*-benzylidene-α/β-ᴅ-glucopyranosyl]-2,3,6-tri-*O*-benzyl-α-ᴅ-glucopyranosyl uronate) (27)

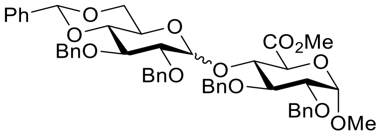

Donor **2** and acceptor **12** were condensed using the general procedure for Tf_2O/Ph_2SO mediated glycosylations (for an additional 24 hours at -40°C) and purified by flash column chromatography (19/1 to 4/1 pentane/EtOAc) to yield glycosylation product **27** (75.2 mg, 90 µmol, 90%, α:β = 5:1). R_f: 0.77 (7/3 pentane/EtOAc). IR (neat): 694, 732, 912, 988, 1026, 1043, 1074, 1086, 1358, 1454, 1749, 28866, 2932; Data for the α-anomer: ^{1}H NMR ($CDCl_3$, 400 MHz, HH-COSY, HSQC, HMBC): δ 7.48 – 7.43 (m, 2H, CH_{arom}), 7.40 – 7.16 (m, 23H, CH_{arom}), 5.51 (s, 1H, C*H*Ph), 5.44 (d, 1H, *J* = 3.8 Hz, H-1'), 4.95 – 4.86 (m, 3H, CH_2 Bn, C*H*H Bn), 4.78 (d, 1H, *J* = 11.2 Hz, CH*H* Bn), 4.71 (d, 1H, *J* = 12.1 Hz, C*H*H Bn), 4.67 (d, 1H, *J* = 12.0 Hz, C*H*H Bn), 4.59 – 4.53 (m, 3H, 2xCH*H* Bn, H-1), 4.28 (dd, 1H, *J* = 6.5, 3.8 Hz, H-6'), 4.25 (d, 1H, *J* = 9.5 Hz, H-5), 4.11 (t, 1H, *J* = 9.1 Hz, H-4), 4.05 (t, 1H, *J* = 8.9 Hz, H-3), 3.98 (t, 1H, *J* = 9.1 Hz, H-3'), 3.76 (s, 3H, CH_3 CO_2Me), 3.64 (t, 1H, *J* = 10.0 Hz, H-6'), 3.61 – 3.54 (m, 3H, H-2, H-4', H-5'), 3.48 (dd, 1H, *J* = 5.6, 3.9 Hz, H-2'), 3.40 (s, 3H, CH_3 OMe); ^{13}C-APT NMR ($CDCl_3$, 101 MHz, HSQC, HMBC): δ 170.1 (C=O CO_2Me), 139.0, 138.6, 138.0, 137.8, 137.6 (C_q), 129.0, 128.6, 128.4, 128.4, 128.3, 128.3, 128.3, 128.2, 128.1, 128.1, 127.8, 127.7, 127.7, 127.3, 127.0, 126.1 (CH_{arom}), 101.3 (CHPh), 98.6 (C-1), 98.4 (C-1'), 82.0 (C-4'), 80.8 (C-3), 79.2 (C-2), 78.7 (C-2'), 78.4 (C-3'), 76.1 (C-4), 75.3, 75.0, 73.7, 73.7 (CH_2 Bn), 70.3 (C-5), 68.6 (C-6'), 63.1 (C-5'), 55.8 (CH_3 OMe), 52.9 (CH_3 CO_2Me); ^{13}C-HMBC NMR ($CDCl_3$, 101 MHz): δ 98.4 ($J_{C1',H1'}$ = 174 Hz, C-1' α); Diagnostic peaks β-anomer: ^{1}H NMR ($CDCl_3$, 400 MHz): δ 5.47 (s, 0.18H, CHPh), 4.62 (d, 0.18H, *J* = 12.1 Hz), 3.87 (dd, 0.18H, *J* = 9.6, 8.4 Hz), 3.50 (s, 0.54H, CH_3 CO_2Me), 3.44 (s, 0.54H, CH_3 OMe), 3.38 – 3.28 (m, 0.36H, H-2', H-5'); ^{13}C-APT NMR ($CDCl_3$, 101 MHz): δ 170.1, 139.2, 138.6, 138.2, 137.4, 129.0, 128.5, 128.3, 128.1, 127.7, 127.5, 126.1, 102.9 (C-1'), 101.2 (CHPh), 99.0 (C-1), 82.3, 81.8, 81.3, 79.6, 78.5, 78.2, 75.6, 75.5, 75.2, 73.9, 70.0, 68.8, 65.9, 55.9, 52.7; ^{13}C-HMBC NMR ($CDCl_3$, 101 MHz): δ 102.9 ($J_{C1',H1'}$ = 164 Hz, C-1' β); HRMS: $[M+Na]^+$ calcd for $C_{49}H_{52}O_{12}Na$ 855.33510, found 855.33496.

Methyl 4-*O*-(2,3-di-*O*-benzyl-4,6-*O*-benzylidene-α-ᴅ-glucopyranosyl)-2,3,6-tri-*O*-benzyl-β-ᴅ-galactopyranoside (28)

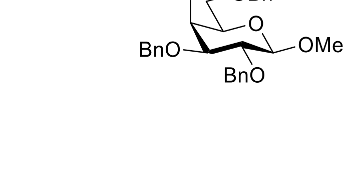

Donor **2** and acceptor **13** were condensed using the general procedure for Tf_2O/Ph_2SO mediated glycosylations (for an additional 16 hours at -40°C) and purified by flash column chromatography (19/1 to 4/1 pentane/EtOAc) to yield glycosylation product **28** (74 mg, 83 µmol, 83%, α:β = > 20:1). R*f*: 0.50 (4/1 pentane/EtOAc). $[\alpha]_D^{23}$ = +38.4° (*c* = 1.0, $CHCl_3$); IR (neat): 696, 735, 997, 1028, 1072, 1366, 1452, 1497, 2859, 2922; ^1H NMR ($CDCl_3$, 400 MHz, HH-COSY, HSQC, HMBC): δ 7.49 (dd, 2H, *J* = 7.9, 1.8 Hz, CH_{arom}), 7.42 – 7.16 (m, 28H, CH_{arom}), 5.50 (s, 1H, C*H*Ph), 4.98 (d, *J* = 3.7 Hz, H-1'), 4.97 (d, *J* = 11.0 Hz, C*H*H Bn), 4.93 – 4.87 (m, 2H, 2xC*H*H Bn), 4.85 – 4.74 (m, 3H, 2xCH*H* Bn, C*H*H Bn), 4.72 – 4.63 (m, 2H, 2xCH*H* Bn), 4.31 – 4.22 (m, 4H, CH_2 Bn, H-1, H-5'), 4.18 (t, 1H, *J* = 9.4 Hz, H-3'), 4.06 – 3.97 (m, 2H, H-4, H-6), 3.84 (dd, 1H, *J* = 10.1, 4.9 Hz, H-6'), 3.72 (dd, 1H, *J* = 10.0, 7.6 Hz, H-2), 3.63 – 3.45 (m, 8H, CH_3 OMe, H-2', H-4', H-5, H-6, H-6'), 3.42 (dd, 1H, *J* = 10.0, 2.9 Hz, H-3); ^{13}C-APT NMR ($CDCl_3$, 101 MHz, HSQC, HMBC): δ 138.9, 138.7, 138.6, 138.4, 138.2, 137.8 (C_q), 128.9, 128.5, 128.4, 128.4, 128.3, 128.3, 128.1, 128.0, 127.8, 127.7, 127.6, 126.2 (CH_{arom}), 105.1 (C-1), 101.2 (CHPh), 100.7 (C-1'), 83.0 (C-4'), 80.6 (C-3), 79.7 (C-2'), 78.9 (C-2, C-3'), 75.9 (C-4), 75.3, 75.2, 74.0 (CH_2 Bn), 73.5 (C-5), 73.2, 72.8 (CH_2 Bn), 69.1 (C-6'), 68.0 (C-6), 63.0 (C-5'), 57.2 (OMe); ^{13}C-HMBC NMR ($CDCl_3$, 101 MHz): δ 105.1 ($J_{C1',H1'}$ = 159 Hz, C-1 β), 100.7 ($J_{C1',H1'}$ = 170 Hz, C-1' α); HRMS: $[M+NH_4]^+$ calcd for $C_{55}H_{62}O_{11}N$ 912.43174, found 912.43266.

Methyl 2-*O*-(2,3-di-*O*-benzyl-4,6-*O*-benzylidene-α/β-D-glucopyranosyl)-3-*O*-benzyl-4,6-*O*-benzylidene-α-D-mannopyranoside (29)

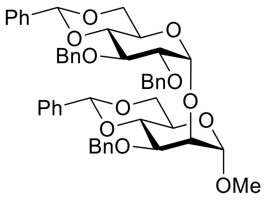

Donor **2** and acceptor **14** were condensed using the general procedure for Tf_2O/Ph_2SO mediated glycosylations (for an additional 16 hours at -40°C) and purified by flash column chromatography (19/1 to 4/1 pentane/EtOAc) to yield glycosylation product **29** (64.3 mg, 80 µmol, 80%, α:β = > 20:1). R*f*: 0.27 (8/1 pentane/EtOAc). Spectroscopic data were in accord with those previously reported.[36] IR (neat): 696, 748, 999, 1028, 1074, 1088, 1369, 1454, 1498, 2864, 2911; ^1H NMR ($CDCl_3$, 400 MHz, HH-COSY, HSQC, HMBC): δ 7.49 (ddd, 4H, *J* = 8.9, 5.8, 1.9 Hz, CH_{arom}), 7.44 – 7.35 (m, 8H, CH_{arom}), 7.35 – 7.24 (m, 10H, CH_{arom}), 7.17 (dp, 3H, *J* = 4.4, 1.6 Hz, CH_{arom}), 5.60 (d, 1H, *J* = 3.9 Hz, H-1'), 5.57 (s, 1H, CHPh'), 5.43 (s, 1H, CHPh), 4.95 – 4.85 (m, 3H, C*H*H Bn, CH_2 Bn), 4.78 (d, 1H, *J* = 11.2 Hz, C*H*H Bn), 4.72 (d, 1H, *J* = 11.7 Hz, CH*H* Bn), 4.71 (d, 1H, *J* = 1.7 Hz, H-1), 4.47 (d, 1H, *J* = 11.1 Hz, CH*H* Bn), 4.33 – 4.26 (m, 2H, H-4, H-6'), 4.24 – 4.18 (m, 2H, H-2, H-6), 4.08 (t, 1H, *J* = 9.3 Hz, H-3'), 4.02 (dd, 1H, *J* = 9.9, 2.9 Hz, H-3), 3.93 – 3.70 (m, 4H, H-5, H-5', H-6, H-6'), 3.63 (t, 1H, *J* = 9.4 Hz, H-4'), 3.56 (dd, 1H, *J* = 9.3, 3.9 Hz, H-2'), 3.36 (s, 3H, CH_3 OMe); ^{13}C-APT NMR ($CDCl_3$, 101 MHz, HSQC, HMBC): δ 139.1, 138.6, 138.5, 137.9, 137.5 (C_q), 129.1, 129.0, 128.5, 128.4, 128.3, 128.3, 128.3, 128.0, 127.9, 127.9, 127.8, 127.6, 127.6, 126.1, 126.1 (CH_{arom}), 101.3, 101.3, 101.2 (CHPh, C-1'), 98.0 (C-1), 82.1 (C-4'), 79.4 (C-2'), 79.3 (C-4), 77.9 (C-3'), 76.9 (C-3), 75.3 (CH_2 Bn), 74.4 (C-2), 74.0, 71.9 (CH_2 Bn), 69.1 (C-6'), 68.8 (C-6), 64.4 (C-5), 63.0 (C-5'), 54.9 (OMe); ^{13}C-HMBC NMR ($CDCl_3$, 101 MHz): δ 101.3 ($J_{C1',H1'}$ = 168 Hz, C-1' α), 98.0 ($J_{C1',H1'}$ = 170 Hz, C-1' α); HRMS: $[M+NH_4]^+$ calcd for $C_{48}H_{54}NO_{11}$ 820.36914, found 820.36958.

Glycoslyations with donor 3

Methyl (cyclohexyl 4-*O*-acetyl-2,3-di-*O*-benzyl-α/β-D-mannopyranosyl uronate) (3A).

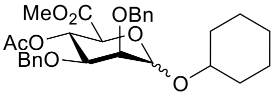

Donor **3** and cyclohexanol were condensed using the general procedure for Tf_2O/Ph_2SO mediated glycosylations (for an additional 16 hours at -40°C) and purified by flash column chromatography (9/1 to 4/1 pentane/EtOAc) to yield glycosylation product **3A** (42.5 mg, 83 µmol, 83%, α:β = 1:8.3). R_f: 0.46 (7/3 pentane/EtOAc). IR (neat): 1026, 1047, 1105, 1238, 1368, 1452, 1740, 1751, 2855, 2930; Data for the β-anomer: ^{1}H NMR (400 MHz, $CDCl_3$, HH-COSY, HSQC): δ 7.48 – 7.18 (m, 10H, CH_{arom}), 5.50 (t, 1H, *J* = 9.7 Hz, H-4), 5.00 (d, 1H, *J* = 12.8 Hz, C*H*H Bn), 4.88 (d, 1H, *J* = 12.8 Hz, CH*H* Bn), 4.54 (s, 1H, H-1), 4.46 (d, 1H, *J* = 12.4 Hz, C*H*H Bn), 4.31 (d, 1H, *J* = 12.4 Hz, CH*H* Bn), 3.84 (d, 1H, *J* = 2.8 Hz, H-2), 3.82 (d, 1H, *J* = 9.7 Hz, H-5), 3.73 (s, 3H, CH_3 CO_2Me), 3.73 – 3.65 (m, 1H, CH Cy), 3.46 (dd, 1H, *J* = 9.8, 2.9 Hz, H-2), 2.02 (s, 3H, CH_3 OAc), 1.99 – 1.21 (m, 15H, CH_2 Cy); ^{13}C-APT NMR (101 MHz, $CDCl_3$, HSQC): δ 169.7, 168.3 (C=O CO_2Me, Ac), 138.6, 138.0 (C_q), 128.7, 128.5, 128.2, 127.8, 127.5, 127.5 (CH_{arom}), 99.5 (C-1), 78.7 (C-3), 77.0 (CH Cy), 74.0 (C-5), 73.9 (CH_2 Bn), 73.4 (C-2), 71.4 (CH_2 Bn), 69.1 (C-4), 52.7 (CH_3 CO_2Me), 33.4, 31.4, 25.8, 23.9 (CH_2 Cy), 21.0 (CH_3 Ac); Diagnostic peaks α-anomer: ^{1}H NMR (400 MHz, $CDCl_3$, HH-COSY, HSQC): δ 5.50 (t, 0.12H, *J* = 9.7 Hz, H-4), 5.24 (d, 0.12H, *J* = 3.3 Hz, H-1), 4.78 (d, 0.12H, *J* = 12.0 Hz, C*H*H Bn), 4.68 (d, 0.12H, *J* = 12.4 Hz, CH*H* Bn); ^{13}C-APT NMR (101 MHz, $CDCl_3$, HSQC): δ 69.7 (C-1); HRMS: $[M+NH_4]^+$ calcd for $C_{29}H_{40}NO_8$ 530.27484, found 530.27495.

Methyl (ethyl 4-*O*-acetyl-2,3-di-*O*-benzyl-α/β-D-mannopyranosyl uronate) (3B).

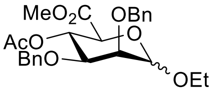

Donor **3** and ethanol were condensed using the general procedure for Tf_2O/Ph_2SO mediated glycosylations (for an additional 16 hours at -40°C) and purified by flash column chromatography (9/1 to 4/1 pentane/EtOAc) to yield glycosylation product **3B** (43.4 mg, 95 µmol, 95%, α:β = 1:8.3). R_f: 0.36 (7/3 pentane/EtOAc). IR (neat): 735, 1026, 1047, 1103, 1229, 1369, 1454, 1744, 2924; Data for the β-anomer: ^{1}H NMR (400 MHz, $CDCl_3$, HH-COSY, HSQC): δ 7.68 – 7.15 (m, 10H, CH_{arom}), 5.51 (t, 1H, *J* = 9.5 Hz, H-4), 4.96 (d, 1H, *J* = 12.6 Hz, C*H*HBn), 4.84 (d, 1H, *J* = 12.6 Hz, CH*H* Bn), 4.48 (d, 1H, *J* = 12.4 Hz, C*H*H Bn), 4.43 (s, 1H, H-1), 4.33 (d, 1H, *J* = 12.4 Hz, CH*H* Bn), 4.02 (dq, 1H, *J* = 9.2, 7.1 Hz, C*H*HCH_3 Et), 3.88 (d, 1H, *J* = 2.8 Hz, H-2), 3.84 (d, 1H, *J* = 9.5 Hz, H-5), 3.73 (s, 3H, CH_3 CO_2Me), 3.54 – 3.44 (m, 2H, H-3, CH*H*CH_3 Et, H-3), 2.02 (s, 3H, CH_3 OAc), 1.27 (t, 3H, *J* = 7.0 Hz, CH_3 Et); ^{13}C-APT NMR (101 MHz, $CDCl_3$, HSQC): δ 169.7, 168.2 (C=O CO_2Me, Ac), 138.5, 137.9 (C_q), 129.4, 128.6, 1285, 128.2, 124.9 (CH_{arom}), 101.5 (C-1), 78.2 (C-3), 73.9 (CH_2 Bn), 73.9 (C-5), 73.1 (C-2), 71.4 (CH_2 Bn), 69.1 (C-4), 65.9 (CH_2 Et), 52.7 (CH_3 CO_2Me), 21.0 (CH_3 Ac), 15.2 (CH_3 Et); ^{13}C-GATED NMR (101 MHz, $CDCl_3$): δ 101.5 ($J_{C1,H1}$ = 160 Hz, C-1 β); Diagnostic peaks α-anomer: ^{1}H NMR (400 MHz, $CDCl_3$, HH-COSY, HSQC): δ 5.51 (t, 0.12H, H-4), 5.13 (d, 0.12H, *J* = 4.3 Hz, H-1), 4.78 (d, 0.12H, *J* = 12.4 Hz, C*H*H Bn), 4.68 (d, 0.12H, *J* = 12.3 Hz, CH*H* Bn), 3.67 (s, 0.36H, CH_3 CO_2Me). ^{13}C-APT NMR (101 MHz, $CDCl_3$) δ 98.4 (C-1), 77.4 (C-3), 74.6 (C-5), 73.2 (CH_2 Bn), 72.5 (CH_2 Bn), 69.5 (CH_2 Et), 52.6 (CH_3 CO_2Me); HRMS: $[M+Na]^+$ calcd for $C_{25}H_{30}O_8Na$ 481.18329, found 481.18250.

Methyl (2-fluoroethyl 4-*O*-acetyl-2,3-di-*O*-benzyl-α/β-D-mannopyranosyl uronate) (3C).

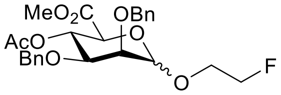

Donor **3** and 2-fluoroethanol were condensed using the general procedure for Tf_2O/Ph_2SO mediated glycosylations (for an additional 16 hours at -40°C) and purified by flash column chromatography (9/1 to 7/3 pentane/EtOAc) to yield glycosylation product **3C** (33.2 mg, 70 µmol, 70%, α:β = 1:5). R*f*: 0.18 (7/3 pentane/EtOAc). IR (neat): 1045, 1103, 1231, 1369, 1454, 1746, 2895, 2924; Data for the β-anomer: ^{1}H NMR (400 MHz, $CDCl_3$, HH-COSY, HSQC): δ 7.69 – 7.18 (m, 10H, CH_{arom}), 5.52 (t, 1H, *J* = 9.3 Hz, H-4), 4.95 (d, 1H, *J* = 12.5 Hz, C*H*H Bn), 4.83 (d, 1H, *J* = 12.5 Hz, CH*H* Bn), 4.76 – 4.94 (m, 2H, CH_2F), 4.54 (s, 1H, H-1), 4.49 (d, 1H, *J* = 12.4 Hz, C*H*H Bn), 4.34 (d, 1H, *J* = 12.4 Hz, CH*H* Bn) 4.13 (dddd, 1H, *J* = 37.0, 12.2, 3.3, 2.2 Hz, C*H*HCH$_2$F), 3.95 (d, 1H, *J* = 2.6 Hz, H-2), 3.86 (d, 1H, *J* = 9.2 Hz, H-5), 3.84 – 3.74 (m, 1H, CH*H*CH$_2$F), 3.72 (s, 3H, CH_3 CO_2Me), 3.49 (dd, 1H, *J* = 9.4, 2.9 Hz, H-3), 2.10 – 1.94 (s, 3H, CH_3 OAc); ^{13}C-APT NMR (101 MHz, $CDCl_3$, HSQC): δ 169.7, 168.1 (C=O CO_2Me, Ac), 138.3, 137.81 (C_q), 131.2, 129.46, 128.6, 128.5, 128.4, 128.3, 128.0, 127.9, 127.7, 127.7, 127.6, 124.9 (CH_{arom}), 101.6 (C-1), 82.96 (d, *J* = 169.5 Hz, CH_2F), 77.9 (C-3), 74.5 (CH_2 Bn), 74.1 (C-5), 73.8 (C-2), 72.9 (CH_2 Bn), 69.01 (d, *J* = 19.5 Hz, *CH$_2$*CH$_2$F), 68.9 (C-4), 52.8 (CH_3 CO_2Me), 21.0 (CH_3 Ac). ^{13}C-GATED NMR (101 MHz, $CDCl_3$) δ 101.6 ($J_{C1,H1}$ = 156 Hz, C-1); Diagnostic peaks α-anomer: ^{1}H NMR (400 MHz, $CDCl_3$, HH-COSY, HSQC): δ 5.52 (t, 0.20H, *J* = 9.3 Hz, H-4), 5.19 (d, 0.20H, *J* = 4.7 Hz, H-1), 3.66 (s, 0.60H, CH_3 CO_2Me), 2.04 (s, 0.6H, CH_3 OAc); ^{13}C-APT NMR (101 MHz, $CDCl_3$): δ 98.9 (C-1); HRMS: $[M+Na]^+$ calcd for $C_{25}H_{29}FO_8Na$ 499.17387, found 499.17297.

Methyl (2,2-difluoroethyl 4-*O*-acetyl-2,3-di-*O*-benzyl-α/β-D-mannopyranosyl uronate) (3D).

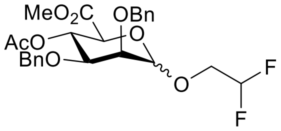

Donor **3** and 2,2-difluoroethanol were condensed using the general procedure for Tf_2O/Ph_2SO mediated glycosylations (for an additional 16 hours at -40°C) and purified by flash column chromatography (9/1 to 7/3 pentane/EtOAc) to yield glycosylation product **3D** (43.1 mg, 87 µmol, 87%, α:β = 1:4.2). R*f*: 0.51 (7/3 pentane/EtOAc). IR (neat): 737, 1026, 1051, 1078, 1232, 1439, 1454, 1741, 2870, 2924; Data for the β-anomer: ^{1}H NMR (400 MHz, $CDCl_3$, HH-COSY, HSQC): δ 7.57 – 7.12 (m, 10H, CH_{arom}), 5.94 (dddd, 1H, *J* = 56.7, 54.4, 5.7, 2.5 Hz, CH$_2$C*H*F$_2$), 5.54 (t, 1H, *J* = 8.9 Hz, H-4), 4.89 (d, 1H, *J* = 12.4 Hz, C*H*H Bn), 4.78 (d, 1H, *J* = 12.4 Hz, C*H*H Bn), 4.56 (s, 1H, H-1), 4.53 (d, 1H, *J* = 12.3 Hz, C*H*H Bn), 4.38 (d, 1H, *J* = 12.4 Hz, C*H*H Bn), 4.19 – 4.04 (m, 1H, C*H*HCHF$_2$), 3.92 (d, 1H, *J* = 2.1 Hz, H-2), 3.89 (d, 1H, *J* = 8.7 Hz, H-5), 3.80 – 3.67 (m, 1H, C*H*HCHF$_2$), 3.70 (s, 3H, CH_3 CO_2Me), 3.52 (dd, 1H, *J* = 9.0, 2.9 Hz, H-3), 2.03 (s, 3H, CH_3 OAc); ^{13}C-APT NMR (101 MHz, $CDCl_3$, HSQC):δ 169.7, 167.9 (C=O CO_2Me, Ac), 138.1, 137.8 (C_q), 131.2, 129.5, 128.5, 128.3, 127.9, 127.8, 127.6, 124.9 (CH_{arom}), 114.3 (dd, *J* = 242.2, 239.7 Hz, CHF_2), 101.3 (C-1), 77.4 (C-3), 74.0 (CH_2 Bn), 73.6 (C-5), 72.8 (C-2), 71.7 (CH_2 Bn), 69.0 (C-4) 68.5 (dd, *J* = 31.2, 25.6 Hz, *CH$_2$*CHF$_2$), 52.8 (CH_3 CO_2Me), 21.0 (CH_3 Ac); ^{13}C-GATED NMR (101 MHz, $CDCl_3$): δ 101.3 ($J_{C1,H1}$ = 160 Hz, C-1 β); Diagnostic peaks α-anomer: ^{1}H NMR (400 MHz, $CDCl_3$, HH-COSY, HSQC): δ 5.52 (t, 0.24H, *J* = 11.2 Hz, H-4), 5.23 (d, 0.24H, *J* = 5.6 Hz, H-1), 3.64 (s, 0.72H, CH_3 CO_2Me); ^{13}C-APT NMR (101 MHz, $CDCl_3$, HSQC):δ 99.31 (C-1), 75.45 (C-3), 74.47 (C-2), 73.41 (CH_2 Bn), 69.46 (C-4), 52.61 (CH_3 CO_2Me); HRMS: $[M+NH_4]^+$ calcd for $C_{25}H_{32}F_2NO_8$ 512.20905, found 512.20889.

Methyl (2,2,2-trifluoroethyl 4-*O*-acetyl-2,3-di-*O*-benzyl-α/β-D-mannopyranosyl uronate) (3E).

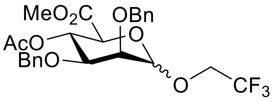

Donor **3** and 2,2,2-trifluoroethanol were condensed using the general procedure for Tf_2O/Ph_2SO mediated glycosylations (for an additional 24 hours at -40°C) and purified by flash column chromatography (9/1 to 7/3 pentane/EtOAc) to yield glycosylation product **3E** (43.7 mg, 85 µmol, 85%, α:β = 1:2.6). R_f: 0.60 (9/1 pentane/EtOAc). IR (neat): 741, 1058, 1161, 1234, 1280, 1371, 1443, 1748, 2854, 2924; Data for the β-anomer: ^1H NMR (400 MHz, $CDCl_3$, HH-COSY, HSQC): δ 7.83 – 6.86 (m, 10H, CH_{arom}), 5.55 (t, 1H, J = 8.6 Hz, H-4), 4.90 (d, 1H, J = 12.3 Hz, C*H*H Bn), 4.78 (d, 1H, J = 12.4 Hz, CH*H* Bn), 4.65 (s, 1H, H-1), 4.54 (d, 1H, J = 12.3 Hz, C*H*H Bn), 4.37 (d, 1H, J = 12.3 Hz, CH*H* Bn), 4.35 – 4.23 (m, 1H, C*H*HCF_3), 3.98 – 3.94 (m, 2H, CH*H*CF_3, H-2), 3.92 (d, 1H, J = 8.3 Hz, H-5), 3.69 (s, 3H, CH_3 CO_2Me), 3.54 (dd, 1H, J = 8.8, 2.8 Hz, H-3), 2.03 (s, 3H, CH_3 OAc); ^{13}C-APT NMR (101 MHz, $CDCl_3$, HSQC): δ 169.7, 167.8 (C=O CO_2Me, Ac), 137.9, 137.8 (C_q), 131.2, 129.5, 129.5, 128.5, 128.5, 128.3, 128.0, 127.9, 127.8, 127.5 (CH_{arom}), 123.8 (q, J = 278.7 Hz, CF_3), 100.8 (C-1), 77.1 (C-3), 73.8 (CH_2 Bn), 73.6 (C-5), 72.4 (C-2), 71.7 (CH_2 Bn), 69.0 (C-4), 66.1 (q, J = 34.8 Hz, *CH_2*CF_3), 52.8 (CH_3 CO_2Me), 21.0 (CH_3 Ac); ^{13}C-GATED NMR (101 MHz, $CDCl_3$): δ 100.8 ($J_{C1,H1}$ = 160 Hz, C-1 β); Diagnostic peaks α-anomer: ^1H NMR (400 MHz, $CDCl_3$, HH-COSY, HSQC): δ 5.51 (t, 0.38H, J = 5.5 Hz, H-4), 5.27 (d, 0.38H, J = 5.6 Hz, H-1), 4.23 – 4.12 (m, 0.38H, C*H*HCHF_2), 3.86 (dd, 0.38H, J = 6.2, 3.0 Hz, H-3); ^{13}C-APT NMR (101 MHz, $CDCl_3$, HSQC): δ 99.1 (C-1), 75.4 (C-3), 74.3 (C-2), 73.5 (CH_2 Bn), 72.9 (CH_2 Bn), 69.4 (C-4), 52.6 (CH_3 CO_2Me), 21.0 (CH_3 Ac); ^{13}C-GATED NMR (101 MHz, $CDCl_3$) δ 99.1 ($J_{C1,H1}$ = 172 Hz, C-1 α); HRMS: $[M+Na]^+$ calcd for $C_{25}H_{27}F_3O_8Na$ 535.15502, found 535.15415.

Methyl (1,1,1,3,3,3-hexafluoro-2-propyl 4-*O*-acetyl-2,3-di-*O*-benzyl-α/β-D-mannopyranosyl uronate) (3F).

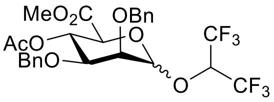

Donor **3** and 1,1,1,3,3,3-hexafluoro-2-propanol were condensed using the general procedure for Tf_2O/Ph_2SO mediated glycosylations (for an additional 240 hours at -40°C) and purified by flash column chromatography (9/1 to 4/1 pentane/EtOAc) to yield glycosylation product **3F** (30.1 mg, 52 µmol, 52%, α:β = 1:1). R_f: 0.85 (α), 0.75 (β) (7/3 pentane/EtOAc). IR (neat): 1105, 1371, 1454, 1751, 2872, 2924; Data for the β-anomer: ^1H NMR (400 MHz, $CDCl_3$, HH-COSY, HSQC): δ 7.72 – 7.13 (m, 10H, CH_{arom}), 5.55 (t, 1H, J = 9.5 Hz, H-4), 4.92 (d, 1H, J = 12.3 Hz, C*H*H Bn), 4.78 (d, 1H, J = 12.1 Hz, CH*H* Bn), 4.74 (s, 1H, H-1), 4.51 (d, 1H, J = 12.3 Hz, C*H*H Bn), 4.72 – 4.56 (m, 1H, $CH(CF_3)_2$) 4.36 (d, 1H, J = 12.7 Hz, CH*H* Bn), 3.99 (d, 1H, J = 2.5 Hz, H-2), 3.87 (d, 1H, J = 9.4 Hz, H-5), 3.73 (s, 3H, CH_3 CO_2Me), 3.50 (dd, 1H, J = 9.6, 2.8 Hz, H-3), 2.02 (s, 3H, CH_3 OAc); ^{13}C-APT NMR (101 MHz, $CDCl_3$, HSQC): δ 169.6, 167.3 (C=O CO_2Me, Ac), 137.8, 137.5 (C_q), 128.6, 128.5, 128.4, 128.0, 128.0, 127.8, 127.6, (CH_{arom}), 120.8 (q, J = 281.0 Hz, CF_3), 100.3 (C-1), 78.0 (C-3), 74.3 (CH_2 Bn), 73.9 (C-5), 72.8 (C-2), 72.4 (CH_2 Bn), 71.8 (hept, J = 33.0 Hz, *$CH(CF_3)_2$*), 68.5 (C-4), 53.0 (CH_3 CO_2Me), 20.9 (CH_3 Ac); ^{13}C-GATED NMR (101 MHz, $CDCl_3$): δ 100.3 ($J_{C1,H1}$ = 165 Hz, C-1 β); Diagnostic peaks α-anomer: ^1H NMR (400 MHz, $CDCl_3$, HH-COSY, HSQC): δ 5.49 (t, 0.90H, J = 5.6 Hz, H-4), 5.39 (d, 0.90H, J = 5.5 Hz, H-1), 4.72 – 4.56 (m, 4.5H, C*H*H Bn, CH*H* Bn, C*H*H Bn, CH*H* Bn, $CH(CF_3)_2$), 4.37 – 4.35 (m, 0.90H, H-5), 3.84 (dd, 0.90H, J = 6.0, 2.9 Hz, H-3), 3.68 (dd, 0.90H, J = 5.5, 2.8 Hz, H-2). ^{13}C-APT NMR (101 MHz, $CDCl_3$, HSQC):δ 169.9, 168.3 (C=O CO_2Me, Ac), 137.8, 137.65 (C_q), 128.6, 128.5, 128.4, 128.0, 128.0, 127.9, 127.8, 127.6 (CH_{arom}), 100.0 (C-1), 75.4 (C-3), 74.3 (CH_2 Bn), 74.3 (C-2), 73.7 (C-2), 73.17 (d, J = 32.8 Hz, *$CH(CF_3)_2$*), 72.7 (C-5), 69.3 (C-4), 52.7 (CH_3 CO_2Me), 21.0 (CH_3 Ac); ^{13}C-GATED NMR (101 MHz, $CDCl_3$): δ 100.0 ($J_{C1,H1}$ = 175 Hz, C-1 α); HRMS: $[M+NH_4]^+$ calcd for $C_{26}H_{30}F_6NO_8$ 598.18707, found 598.18711.

Methyl (4-*O*-acetyl-2,3-di-*O*-benzyl-1-deoxy-β-deuterio-D-mannopyranosyl uronate) (3G).

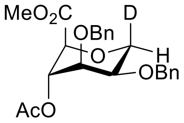

Donor **3** and triethylsilane-D were condensed using the general procedure for Tf_2O/Ph_2SO mediated glycosylations (for an additional 240 hours at -40°C) and purified by flash column chromatography (9/1 to 7/3 pentane/EtOAc) to yield glycosylation product **3G** (39.6 mg, 95 µmol, 95%, α:β = < 1:20). R*f*: 0.45 (7/3 pentane/EtOAc). $[\alpha]_D^{26}$ = -34.4° (*c* = 0.5, $CHCl_3$); IR (neat): 698, 736, 1051, 1136, 1228, 1371, 1454, 1745, 2872, 2924; Data for the β-anomer: ^{1}H NMR (400 MHz, $CDCl_3$, HH-COSY, HSQC, NOESY) δ 7.39 – 7.17 (m, 10H, CH_{arom}), 5.60 (dd, 1H, *J* = 4.9, 3.5 Hz, H-4), 4.63 (s, 2H, CH_2 Bn), 4.53 (s, 2H, CH_2 Bn), 4.19 (d, 1H, *J* = 3.2 Hz, H-5), 3.81 (m, 2H, H-2, H-3), 3.68 (d, 1H, *J* = 3.9 Hz, H-1), 3.61 (s, 3H, CH_3 CO_2Me), 2.06 (s, 3H, CH_3 OAc); ^{2}H NMR (61 MHz, $CHCl_3$) δ 4.73 (D-1); ^{13}C-APT NMR (101 MHz, $CDCl_3$, HSQC, HMBC): δ 169.9, 168.9 (C=O CO_2Me, Ac), 138.1, 137.8 (C_q), 131.2, 129.4, 128.6, 128.5, 128.4, 127.9, 127.8, 127.8, 127.8, 124.9 (CH_{arom}), 73.8 (C-5), 73.4 (C-3), 72.3 (CH_2 Bn), 71.4 (C-2), 71.3 (CH_2 Bn), 70.0 (C-4), 62.4 (C-1), 52.4 (CH_3 CO_2Me), 21.1 (CH_3 Ac). HRMS: $[M+Na]^+$ calcd for $C_{23}H_{25}DO_7Na$ 438.16335, found 438.16264.

Methyl (allyl 4-*O*-acetyl-2,3-di-*O*-benzyl-1-deoxy-β-D-mannopyranosyl uronate) (3H).

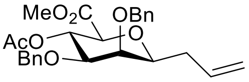

Donor **3** and allyl trimethylsilane were condensed using the general procedure for Tf_2O/Ph_2SO mediated glycosylations (for an additional 96 hours at -40°C) and purified by flash column chromatography (9/1 to 7/3 pentane/EtOAc) to yield glycosylation product **3H** (18.3 mg, 40 µmol, 40%, α:β = < 1:20). R*f*: 0.25 (8/2 pentane/EtOAc). $[\alpha]_D^{20}$ = -38.8° (*c* = 1, $CHCl_3$); IR (neat): 696, 735, 1026, 1055, 1114, 1228, 1368, 1746, 2855, 2924; Data for the β-anomer: ^{1}H NMR (400 MHz, $CDCl_3$, HH-COSY, HSQC, NOESY): δ 7.36 – 7.20 (m, 10H, CH_{arom}), 5.71 – 5.58 (m, 1H, C*H*CH_2 allyl), 5.53 (t, 1H, *J* = 9.8 Hz, H-4), 5.07 – 4.95 (m, 1H, CHC*H2* allyl), 5.01 (d, 1H, *J* = 11.5 Hz, C*H*H Bn), 4.72 (d, 1H, *J* = 12.2 Hz, C*H*H Bn), 4.66 (d, 1H, *J* = 11.6 Hz, CH*H* Bn), 4.61 (d, 1H, *J* = 12.2 Hz, CH*H* Bn), 3.83 (d, 1H, *J* = 9.9 Hz, H-5), 3.79 (d, 1H, *J* = 2.3 Hz, H-2), 3.71 (s, 3H, CH_3 CO_2Me), 3.60 (dd, 1H, *J* = 9.9, 2.7 Hz, H-3), 3.36 (t, 1H, *J* = 7.1 Hz, H-1), 2.56 – 2.48 (m, 1H, C*H*HCH allylic), 2.40 – 2.26 (m, 1H, CH*H*CH), 2.01 (s, 3H, CH_3 OAc); ^{13}C-APT NMR (101 MHz, $CDCl_3$, HSQC, HMBC): δ 169.9, 168.5 (C=O CO_2Me, Ac), 138.3, 138.0 (C_q), 134.0 (*CH*CH_2 allyl), 131.3, 131.2, 129.4, 129.1, 128.6, 128.6, 128.6, 128.5, 128.4, 128.3, 128.1, 128.1, 128.0, 127.9, 127.8, 127.7, 127.6, 127.6, 124.9 (CH_{arom}), 117.9 (CH*CH2* allyl), 88.6 (H-3), 79.1 (H-1), 77.6 (C-5), 74.5 (CH_2 Bn), 73.7 (C-2), 72.6 (CH_2 Bn), 69.5 (C-4), 52.7 (CH_3 CO_2Me), 35.4 (*CH2*CH allyl), 21.0 (CH_3 Ac); HRMS: $[M+NH_4]^+$ calcd for $C_{26}H_{34}NO_7$ 472.23298, found 472.23294.

Methyl 6-*O*-(methyl [4-*O*-acetyl-2,3-di-*O*-benzyl-β-D-mannopyranosyl uronate])-2,3,4-tri-*O*-benzyl-α-D-glucopyranoside (30).

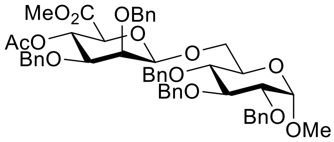

Donor **3** and acceptor **10** were condensed using the general procedure for Tf_2O/Ph_2SO mediated glycosylations (for an additional 16 hours at -40°C) and purified by flash column chromatography (9/1 to 7/3 pentane/EtOAc) to yield glycosylation product **30** (57.5 mg, 66 µmol, 66%, α:β = < 1:20). R_f: 0.54 (7/3 pentane/EtOAc). Spectroscopic data were in accord with those previously reported.[43] $[\alpha]_D^{26}$ = -11,6° (*c* = 1, $CHCl_3$), (lit:[43] $[\alpha]_D^{22}$ = -11.0° (*c* = 0.6, $CHCl_3$)). IR (neat): 733, 906, 1028, 1055, 1101, 1242, 1361, 1452, 1748, 2908; Data for the β-anomer: ^{1}H NMR (400 MHz, $CDCl_3$, HH-COSY, HSQC, HMBC): δ 7.40 – 7.19 (m, 25H, CH_{arom}), 5.48 (t, 1H, *J* = 9.6 Hz, H-4'), 5.02 (d, 1H, *J* = 10.9 Hz, C*H*H Bn), 4.91 (d, 1H, *J* = 12.6 Hz, C*H*H Bn), 4.83 (d, 1H, *J* = 10.9 Hz, CH*H* Bn), 4.82 (d, 1H, *J* = 11.7 Hz, C*H*H Bn), 4.80 – 4.71 (m, 3H, CH*H* Bn, CH*H* Bn, C*H*H Bn), 4.67 (d, 1H, *J* = 12.2 Hz, CH*H* Bn), 4.57 (d, 1H, *J* = 3.5 Hz, H-1), 4.50 (d, 1H, *J* = 4.4 Hz, CH*H* Bn), 4.47 (d, 1H, *J* = 5.3 Hz, C*H*H Bn), 4.36 (d, 1H, *J* = 12.4 Hz, CH*H* Bn), 4.16 – 4.09 (m, 2H, H-6, H-1'), 4.01 (t, 1H, *J* = 9.2 Hz, H-3), 3.79 (dq, 1H, *J* = 7.3, 2.8, 1.7 Hz, H-5), 3.74 (d, 1H, *J* = 9.5 Hz, H-5'), 3.72 – 3.66 (m, 4H, CH_3 CO_2Me, H-2'), 3.50 (dd, 1H, *J* = 9.7, 3.5 Hz, H-2), 3.45 – 3.34 (m, 3H, H-4, H-6, H-3'), 3.31 (s, 3H, CH_3 OMe), 2.02 (s, 3H, CH_3 OAc); ^{13}C-APT NMR (101 MHz, $CDCl_3$, HSQC, HMBC): δ 169.7, 168.1 (C=O CO_2Me, Ac), 138.9, 138.4, 138.1, 137.8 (C_q), 128.6, 128.5, 128.5, 128.5, 128.3, 128.3, 128.2, 128.1, 128.1, 127.9, 127.8, 127.8, 127.6, 127.6 (CH_{arom}), 101.7 (C-1'), 97.9 (C-1), 82.2 (H-3), 79.9 (H-2), 78.3 (H-3'), 77.7 (H-4), 75.9 (CH_2 Bn), 74.9 (CH_2 Bn), 73.8 (C-5'), 73.7 (CH_2 Bn), 73.5 (CH_2 Bn), 72.9 (C-2'), 71.6 (CH_2 Bn), 69.8 (C-5), 69.0 (C-4'), 68.8 (C-6), 55.2 (CH_3 OMe), 52.7 (CH_3 CO_2Me), 21.0 (CH_3 Ac); ^{13}C-GATED NMR (101 MHz, $CDCl_3$) δ 101.7 ($J_{C1,H1}$ = 155 Hz, C-1' β); HRMS: $[M+Na]^+$ calcd for $C_{51}H_{56}O_{13}Na$ 899.36131, found 899.36111.

Methyl 4-*O*-(methyl [4-*O*-acetyl-2,3-di-*O*-benzyl-β-D-mannopyranosyl uronate])-2,3,6-tri-*O*-benzyl-α-D-glucopyranoside (31).

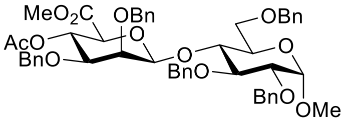

Donor **3** and acceptor **11** were condensed using the general procedure for Tf_2O/Ph_2SO mediated glycosylations (for an additional 16 hours at -40°C) and purified by flash column chromatography (9/1 to 7/3 pentane/EtOAc) to yield glycosylation product **31** (53.1 mg, 61 µmol, 61%, α:β = < 1:20). R_f: 0.65 (7/3 pentane/EtOAc); $[\alpha]_D^{26}$ = -30.2° (*c* = 1, $CHCl_3$). IR (neat): 733, 1026, 1096, 1366, 1454, 1746, 2920; Data for the β-anomer: ^{1}H NMR (400 MHz, $CDCl_3$, HH-COSY, HSQC, HMBC): δ 7.39 – 7.19 (m, 25H, CH_{arom}), 5.41 (t, 1H, *J* = 9.7 Hz, H-4'), 5.17 (d, 1H, *J* = 11.3 Hz, C*H*H Bn), 4.78 – 4.72 (m, 4H, CH*H* Bn, C*H*H Bn, CH*H* Bn, C*H*H Bn), 4.64 – 4.56 (m, 3H, CH*H* Bn, C*H*H Bn, H-1), 4.44 (d, 1H, *J* = 12.3 Hz, CH*H* Bn), 4.40 (s, 1H, H-1'), 4.36 (d, 1H, *J* = 12.3 Hz, C*H*H Bn), 4.28 (d, 1H, *J* = 12.1 Hz, CH*H* Bn), 3.89 (m, 2H, H-3, H-5), 3.68 – 3.63 (m, 1H,), 3.62 (d, 1H, *J* = 2.7 Hz, H-2'), 3.57 (d, 1H, *J* = 9.7 Hz, H-5'), 3.55 – 3.45 (m, 5H, H-2, CH_3 CO_2Me, C-6), 3.38 (s, 3H, CH_3 OMe), 3.18 (dd, 1H, *J* = 9.7, 2.8 Hz, H-3'), 2.01 (s, 3H, CH_3 OAc); ^{13}C-APT NMR (101 MHz, $CDCl_3$, HSQC, HMBC): δ 169.7, 167.9 (C=O CO_2Me, Ac), 139.6, 138.5, 138.3, 138.1, 137.9 (C_q), 128.7, 128.5, 128.5, 128.2, 128.2, 128.2, 128.2, 128.1, 128.0, 127.9, 127.9, 127.8, 127.5, 127.4, 127.1 (CH_{arom}), 101.1 (C-1'), 98.4 (C-1), 80.4 (C-3), 79.3 (H-2), 78.8 (C-3'), 78.2 (C-5), 75.4 (CH_2 Bn), 74.6 (C-2'), 74.2 (C-5'), 73.7 (CH_2 Bn), 73.7 (CH_2 Bn), 73.6 (CH_2 Bn), 71.8 (CH_2 Bn), 69.5 (H-4), 69.0 (C-4'), 68.7 (C-6), 55.4 (CH_3 OMe), 52.5 (CH_3 CO_2Me), 20.9 (CH_3 Ac); ^{13}C-GATED NMR (101 MHz, $CDCl_3$): δ 101.1 ($J_{C1,H1}$ = 158 Hz, C-1' β); HRMS: $[M+Na]^+$ calcd for $C_{51}H_{56}O_{13}Na$ 899.36131, found 899.36094.

Methyl (methyl 4-*O*-[methyl (4-*O*-acetyl-2,3-di-*O*-benzyl-α/β-D-mannopyranosyl uronate)]-2,3-di-*O*-benzyl-α-D-glucopyranosyl uronate) (32).

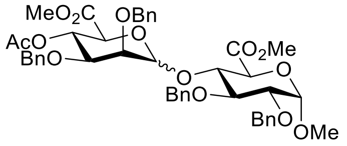

Donor **3** and acceptor **12** were condensed using the general procedure for Tf_2O/Ph_2SO mediated glycosylations (for an additional 48 hours at -40°C) and purified by flash column chromatography (9/1 to 7/3 pentane/EtOAc) to yield glycosylation product **32** (58.0 mg, 71 µmol, 71%, α:β = 1:10). R_f: 0.38 (7/3 pentane/EtOAc); $[\alpha]_D^{26}$ = -31.2° (*c* = 1, $CHCl_3$). IR (neat): 733, 1026, 1043, 1229, 1454, 1744, 2855, 2926; Data for the β-anomer: ^{1}H NMR (400 MHz, $CDCl_3$, HH-COSY, HSQC, HMBC): δ 7.42 – 7.23 (m, 20H, CH_{arom}), 5.44 (t, 1H, *J* = 9.8 Hz, H-4'), 5.19 (d, 1H, *J* = 11.3 Hz, C*H*H Bn), 4.88 – 4.69 (m, 4H, CH*H* Bn, CH*H* Bn, C*H*H Bn, CH*H* Bn), 4.62 – 4.38 (m, 5H, CH*H* Bn, C*H*H Bn, C*H*H Bn, H-1', H-1), 4.08 (d, 1H, *J* = 9.3 Hz, H-5), 3.96 – 3.85 (m, 2H, H-3, H-4), 3.76 (d, 1H, *J* = 2.7 Hz, H-2'), 3.70 (d, 1H, *J* = 9.7 Hz, H-5'), 3.59 (s, 3H, CH_3 CO_2Me), 3.53 – 3.46 (m, 4H, CH_3 CO_2Me, H-2), 3.46 – 3.37 (m, 4H, CH_3 OMe, H-3'), 2.00 (s, 3H, CH_3 OAc); ^{13}C-APT NMR (101 MHz, $CDCl_3$, HSQC, HMBC): δ 170.2, 169.8, 167.8 (C=O CO_2Me, Ac), 139.4, 138.6, 138.1, 137.9 (C_q), 128.6, 128.5, 128.5, 128.5, 128.4, 128.3, 128.3, 128.2, 128.2, 128.1, 127.9, 127.8, 127.7, 127.6, 127.5, 127.2 (CH_{arom}), 102.3 (C-1'), 98.9 (C-1), 80.7 (C-4), 80.0 (C-3), 78.6 (C-2), 78.4 (C-3'), 75.7 (CH_2 Bn), 74.7 (C-2'), 74.5 (CH_2 Bn), 73.9 (CH_2 Bn, C-5'), 71.8 (CH_2 Bn), 69.6 (C-5), 69.0 (C-4'), 56.0 (CH_3 OMe), 52.6 (CH_3 CO_2Me), 52.5 (CH_3 CO_2Me), 20.9 (CH_3 Ac); ^{13}C-GATED NMR (101 MHz, $CDCl_3$): δ 102.3 ($J_{C1,H1}$ = 154 Hz, C-1' β); Diagnostic peaks α-anomer: ^{1}H NMR (400 MHz, $CDCl_3$, HH-COSY, HSQC): 5.44 (m, 0.20H, H-1', H-4'), 2.00 (s, 0.30H, CH_3 OAc); HRMS: $[M+Na]^+$ calcd for $C_{45}H_{50}O_{14}Na$ 837.30928, found 837.30903.

Methyl 4-*O*-(methyl [4-*O*-acetyl-2,3-di-*O*-benzyl-β-D-mannopyranosyl uronate])-2,3,6-tri-*O*-benzyl-β-D-galactopyranoside (33).

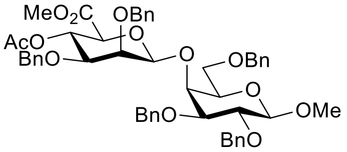

Donor **3** and acceptor **13** were condensed using the general procedure for Tf_2O/Ph_2SO mediated glycosylations (for an additional 16 hours at -40°C) and purified by flash column chromatography (9/1 to 7/3 pentane/EtOAc) to yield glycosylation product **33** (66.8 mg, 76 µmol, 76%, α:β = < 1:20). R_f: 0.39 (7/3 pentane/EtOAc); $[\alpha]_D^{26}$ = -31.6° (*c* = 1, $CHCl_3$). IR (neat): 735, 1026, 1051 1231, 1748, 2870; Data for the β-anomer: ^{1}H NMR (400 MHz, $CDCl_3$, HH-COSY, HSQC, HMBC): δ 7.35 – 7.22 (m, 25H, CH_{arom}), 5.45 (t, 1H, *J* = 9.8 Hz, H-4'), 4.95 (d, 1H, *J* = 12.7 Hz, C*H*H Bn), , 4.93 (d, 1H, *J* = 10.9 Hz, CH*H* Bn), 4.85 (d, 1H, *J* = 12.7 Hz, CH*H* Bn), 4.78 (d, 1H, *J* = 11.7 Hz, C*H*H Bn), 4.75 (s, 1H, H-1'), 4.67 (d, 1H, *J* = 11.0 Hz, CH*H* Bn), 4.60 (d, 1H, *J* = 11.7 Hz, C*H*H Bn), 4.58 (d, 1H, *J* = 11.7 Hz, CH*H* Bn), 4.51 (d, 1H, *J* = 11.7 Hz, CH*H* Bn), 4.43 (d, 1H, *J* = 12.3 Hz, C*H*H Bn), 4.30 (d, 1H, *J* = 7.6 Hz, H-1), 4.22 (d, 1H, *J* = 12.3 Hz, CH*H* Bn), 4.15 (d, 1H, *J* = 2.5 Hz, H-2), 3.95 (d, 1H, *J* = 2.8 Hz, H-2'), 3.90 (dd, 1H, *J* = 9.7, 6.3 Hz, H-6), 3.74 (dd, 1H, *J* = 9.6, 5.7 Hz, H-6), 3.70 – 3.63 (m, 2H, H-5, H-5'), 3.63 – 3.56 (m, 7H, H-4, CH_3 OMe, CH_3 CO_2Me), 3.56 – 3.50 (m, 1H, H-3), 3.24 (dd, 1H, *J* = 9.8, 2.9 Hz, H-3'), 1.99 (s, 3H, CH_3 OAc); ^{13}C-APT NMR (101 MHz, $CDCl_3$, HSQC, HMBC): δ 169.8, 168.1 (C=O CO_2Me, Ac), 138.8, 138.7, 138.3, 138.0 (C_q), 128.7, 128.6, 128.5, 128.3, 128.2, 128.0, 127.9, 127.8, 127.8, 127.7, 127.5, 127.5 (CH_{arom}), 105.1 (C-1), 101.9 (C-1'), 81.8 (C-3), 79.6 (C-5), 79.0 (C-3'), 75.1 (CH_2 Bn), 73.9 (C-5), 73.8 (CH_2 Bn), 73.6 (CH_2 Bn), 73.6 (C-4), 73.2 (C-2), 72.5 (C-

2'), 71.3 (CH_2 Bn), 69.4 (C-6), 68.9 (C-4'),57.2 (CH_3 CO_2Me), 52.6 (CH_3 OMe), 20.9 (CH_3 Ac); HRMS: $[M+Na]^+$ calcd for $C_{51}H_{56}O_{13}Na$ 899.36131, found 899.36109.

Methyl 2-*O*-(methyl [4-*O*-acetyl-2,3-di-*O*-benzyl-α/β-D-mannopyranosyl uronate])-3-*O*-benzyl-4,6-bezylidene-α-D-mannopyranoside (34).

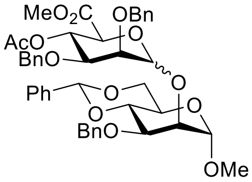

Donor **3** and acceptor **14** were condensed using the general procedure for Tf_2O/Ph_2SO mediated glycosylations (for an additional 16 hours at -40°C) and purified by flash column chromatography (9/1 to 7/3 pentane/EtOAc) to yield glycosylation product **34** (60.4 mg, 77 μmol, 77%, α:β = 1:7.1). R*f*: .34 (7/3 pentane/EtOAc); $[\alpha]_D^{26}$ = -48.6° (*c* = 1, $CHCl_3$). IR (neat): 735, 1026, 1053, 1230, 1369, 1454, 1748, 2868, 2924; Data for the β-anomer: ^{1}H NMR (400 MHz, $CDCl_3$, HH-COSY, HSQC, HMBC): δ 7.51 – 7.22 (m, 15H, CH_{arom}), 5.60 – 5.53 (m, 2H, H-4', C*H*Ph), 5.02 (d, 1H, *J* = 12.5 Hz, C*H*H Bn), 4.93 (d, 1H, *J* = 12.5 Hz, CH*H* Bn), 4.83 (d, 1H, *J* = 12.1 Hz, C*H*H Bn), 4.72 (d, 1H, *J* = 1.1 Hz, H-1), 4.64 (d, 1H, *J* = 12.1 Hz, CH*H* Bn), 4.62 (s, 1H, H-1'), 4.50 (d, 1H, *J* = 12.4 Hz, C*H*H Bn), 4.36 – 4.31 (m, 2H, CH*H* Bn, H-2), 4.29 – 4.24 (m, 1H, H-6), 4.15 – 4.07 (m, 1H, H-4), 4.01 – 3.91 (m, 2H, H-2', H-3), 3.85 (d, 1H, *J* = 9.7 Hz, H-5'), 3.82 – 3.75 (m, 2H, H-5, H-6), 3.63 (s, 3H, CH_3 CO_2Me), 3.48 (dd, 1H, *J* = 9.7, 2.9 Hz, H-3'), 3.35 (s, 3H, CH_3 OMe), 2.03 (s, 3H, CH_3 OAc); ^{13}C-APT NMR (101 MHz, $CDCl_3$, HSQC, HMBC): δ 169.7, 167.9 (C=O CO_2Me, Ac), 138.8, 138.5, 137.8, 137.7 (C_q), 129.0, 128.7, 128.5, 128.4, 128.4, 128.4, 128.3, 128.3, 127.9, 127.9, 127.8, 127.8, 127.7, 127.6, 127.5, 127.4, 126.3, 126.2 (C_{arom}), 101.8 (CHPh), 99.8 (C-1'), 99.2 (C-1), 78.5 (C-4), 78.2 (C-3), 74.0 (C-5'), 73.9 (H-2), 73.9 (H-3), 73.9 (CH_2 Bn), 73.0 (H-2'), 71.3 (CH_2 Bn), 71.0 (CH_2 Bn), 69.0 (C-6), 68.8 (C-4'), 64.1 (H-5), 55.1 (CH_3 OMe), 52.7 (CH_3 CO_2Me), 21.0 (CH_3 Ac); ^{13}C-GATED NMR (101 MHz, $CDCl_3$): δ 99.8 ($J_{C1,H1}$ = 154 Hz, C-1' β); Diagnostic peaks α-anomer: ^{1}H NMR (400 MHz, $CDCl_3$, HH-COSY, HSQC, HMBC): 5.59 (s, 0.14H, C*H*Ph), 5.50 (t, 0.14H, *J* = 7.5 Hz, H-4'), 5.45 (d, 0.14H, *J* = 4.0 Hz, H-1'), 3.66 (s, 0.42H, CH_3 CO_2Me), 3.35 (s, 0.42H, CH_3 OMe), 2.03 (s, 0.42H, CH_3 OAc); ^{13}C-APT NMR (101 MHz, $CDCl_3$, HSQC, HMBC): δ 100.3 (C-1'), 69.5 (C-4'); HRMS: $[M+NH_4]^+$ calcd for $C_{44}H_{52}NO_{13}$ 802.34332, found 802.34387.

Literature references in Supporting Information

1 D. Crich, M. Smith, Q. Yao and J. Picione, *Synthesis*, 2001, **2001**, 0323–0326.
2 Spartan '04 and J. Kong, *et al.*, 2004, Wavefunction Inc.
3 Gaussian 03 (Revision E.01) and M. J. Frisch, *et al.*, 2004, Gaussian, Inc.
4 V. Pedretti, A. Veyrières and P. Sinaÿ, *Tetrahedron*, 1990, **46**, 77–88.
5 T. Kametani, K. Kawamura and T. Honda, *J. Am. Chem. Soc.*, 1987, **109**, 3010–3017.
6 K. Kohata and H. Meguro, *Agric. Biol. Chem.*, 1982, **46**, 919–925.
7 M. Emmadi and S. S. Kulkarni, *J. Org. Chem.*, 2011, **76**, 4703–4709.
8 Y. J. Lee, J. Y. Baek, B.-Y. Lee, S. S. Kang, H.-S. Park, H. B. Jeon and K. S. Kim, *Carbohydr. Res.*, 2006, **341**, 1708–1716.
9 P. Smid, D. Noort, H. J. G. Broxterman, N. C. R. van Straten, G. A. van der Marel and J. H. van Boom, *Recl. Trav. Chim. Pays-Bas*, 1992, **111**, 524–528.
10 N. Michihata, Y. Kaneko, Y. Kasai, K. Tanigawa, T. Hirokane, S. Higasa and H. Yamada, *J. Org. Chem.*, 2013, **78**, 4319–4328.
11 E. I. Balmond, D. M. Coe, M. C. Galan and E. M. McGarrigle, *Angew. Chem. Int. Ed.*, 2012, **51**, 9152–9155.
12 P. Blom, B. Ruttens, S. Van Hoof, I. Hubrecht, J. Van der Eycken, B. Sas, J. Van hemel and J. Vandenkerckhove, *J. Org. Chem.*, 2005, **70**, 10109–10112.
13 R. Raju, B. F. Castillo, S. K. Richardson, M. Thakur, R. Severins, M. Kronenberg and A. R. Howell, *Bioorg. Med. Chem. Lett.*, 2009, **19**, 4122–4125.

14 A. J. Janczuk, W. Zhang, P. R. Andreana, J. Warrick and P. G. Wang, *Carbohydr. Res.*, 2002, **337**, 1247–1259.
15 R. W. Gantt, R. D. Goff, G. J. Williams and J. S. Thorson, *Angew. Chem. Int. Ed.*, 2008, **47**, 8889–8892.
16 D. Crich and W. Cai, *J. Org. Chem.*, 1999, **64**, 4926–4930.
17 J. D. C. Codée, L. J. van den Bos, A.-R. de Jong, J. Dinkelaar, G. Lodder, H. S. Overkleeft and G. A. van der Marel, *J. Org. Chem.*, 2009, **74**, 38–47.
18 S. Boonyarattanakalin, X. Liu, M. Michieletti, B. Lepenies and P. H. Seeberger, *J. Am. Chem. Soc.*, 2008, **130**, 16791–16799.
19 K. Yoza, N. Amanokura, Y. Ono, T. Akao, H. Shinmori, M. Takeuchi, S. Shinkai and D. N. Reinhoudt, *Chem. – Eur. J.*, 1999, **5**, 2722–2729.
20 K. Michigami and M. Hayashi, *Tetrahedron*, 2013, **69**, 4221–4225.
21 A. B. Ingle, C.-S. Chao, W.-C. Hung and K.-K. T. Mong, *Org. Lett.*, 2013, **15**, 5290–5293.
22 K. P. R. Kartha, M. Aloui and R. A. Field, *Tetrahedron Lett.*, 1996, **37**, 8807–8810.
23 B. Ren, H. Dong and O. Ramström, *Chem. – Asian J.*, 2014, **9**, 1298–1304.
24 W. J. Goux, *Carbohydr. Res.*, 1988, **184**, 47–65.
25 K. Bock, S. Refn, C. Pedersen, R. W. Taft and G. W. Fischer, *Acta Chem. Scand.*, 1987, **41b**, 469–472.
26 C.-H. Wong, F. Moris-Varas, S.-C. Hung, T. G. Marron, C.-C. Lin, K. W. Gong and G. Weitz-Schmidt, *J. Am. Chem. Soc.*, 1997, **119**, 8152–8158.
27 E. Attolino, G. Catelani and F. D'Andrea, *Eur. J. Org. Chem.*, 2006, **2006**, 5279–5292.
28 P. J. Garegg, H. Hultberg and S. Wallin, *Carbohydr. Res.*, 1982, **108**, 97–101.
29 S. C. Ennis, I. Cumpstey, A. J. Fairbanks, T. D. Butters, M. Mackeen and M. R. Wormald, *Tetrahedron*, 2002, **58**, 9403–9411.
30 M. Giordano and A. Iadonisi, *J. Org. Chem.*, 2014, **79**, 213–222.
31 M. Poláková, M. U. Roslund, F. S. Ekholm, T. Saloranta and R. Leino, *Eur. J. Org. Chem.*, 2009, **2009**, 870–888.
32 H. Nagai, K. Sasaki, S. Matsumura and K. Toshima, *Carbohydr. Res.*, 2005, **340**, 337–353.
33 P. Sun, P. Wang, Y. Zhang, X. Zhang, C. Wang, S. Liu, J. Lu and M. Li, *J. Org. Chem.*, 2015, **80**, 4164–4175.
34 J.-R. Ella-Menye, X. Nie and G. Wang, *Carbohydr. Res.*, 2008, **343**, 1743–1753.
35 D. Crich and I. Sharma, *Org. Lett.*, 2008, **10**, 4731–4734.
36 K. S. Kim, D. B. Fulse, J. Y. Baek, B.-Y. Lee and H. B. Jeon, *J. Am. Chem. Soc.*, 2008, **130**, 8537–8547.
37 J. Y. Baek, T. J. Choi, H. B. Jeon and K. S. Kim, *Angew. Chem. Int. Ed.*, 2006, **45**, 7436–7440.
38 K. S. Kim, J. H. Kim, Y. J. Lee, Y. J. Lee and J. Park, *J. Am. Chem. Soc.*, 2001, **123**, 8477–8481.
39 D. Crich and S. Sun, *Tetrahedron*, 1998, **54**, 8321–8348.
40 K. Worm-Leonhard, K. Larsen and K. J. Jensen, *J. Carbohydr. Chem.*, 2007, **26**, 349–368.
41 X. Nie and G. Wang, *J. Org. Chem.*, 2005, **70**, 8687–8692.
42 M. Moumé-Pymbock and D. Crich, *J. Org. Chem.*, 2012, **77**, 8905–8912.
43 L. J. van den Bos, J. Dinkelaar, H. S. Overkleeft and G. A. van der Marel, *J. Am. Chem. Soc.*, 2006, **128**, 13066–13067.

NMR spectra of new compounds

^{1}H NMR, 400 MHz, $CDCl_3$ of compound **1B**

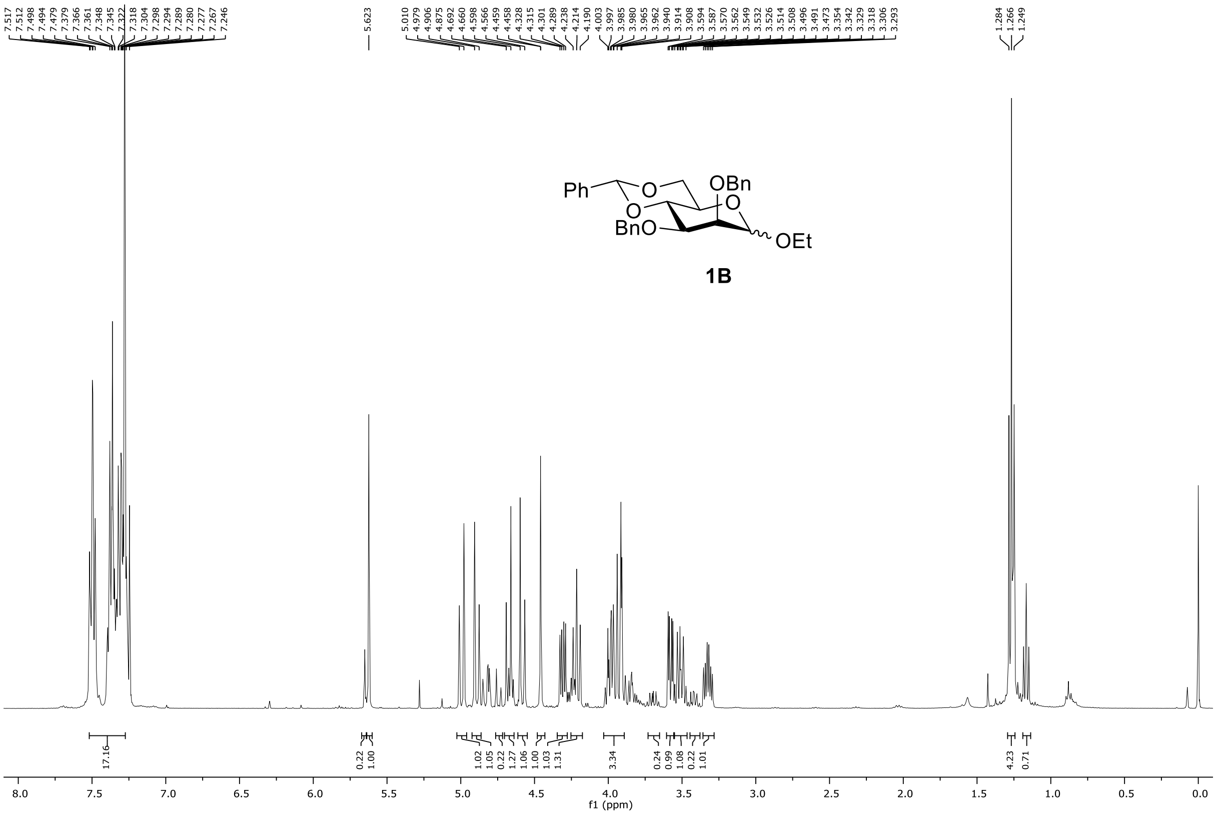

^{13}C-APT NMR, 101 MHz, $CDCl_3$ of compound **1B**

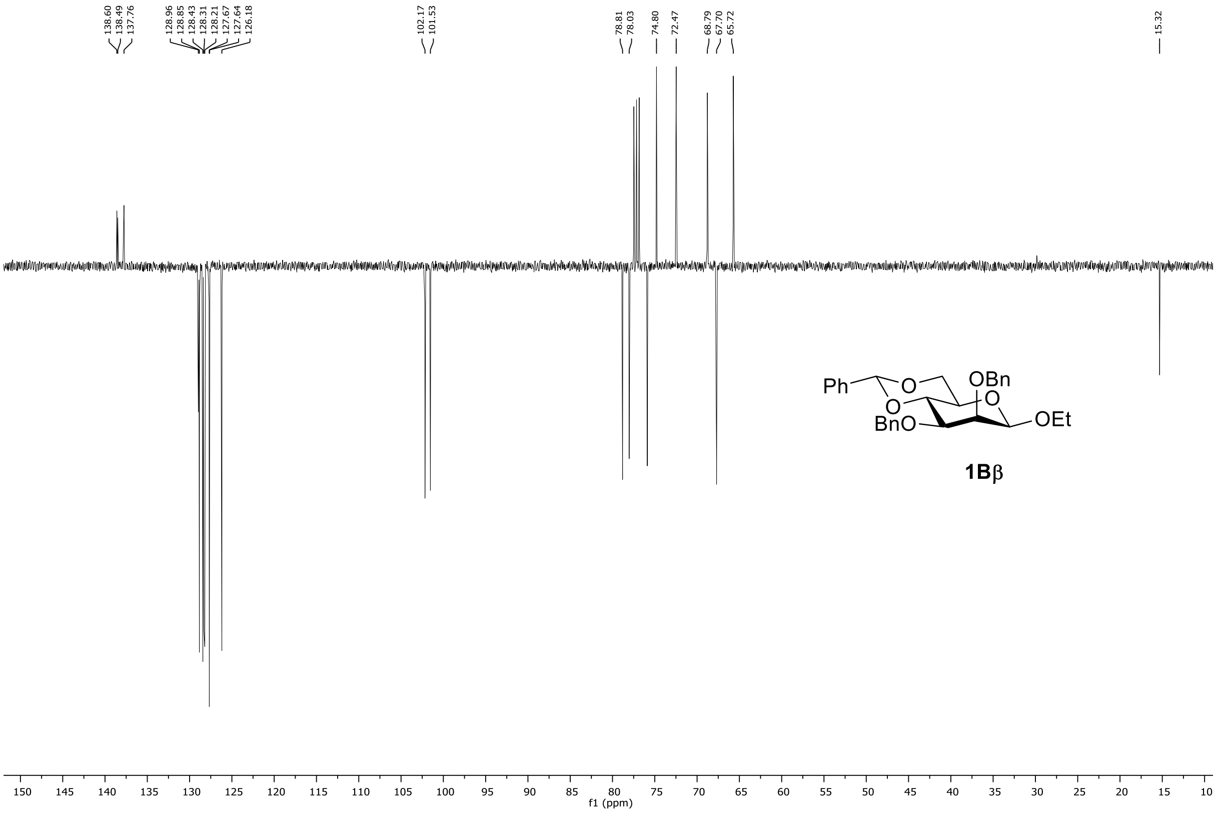

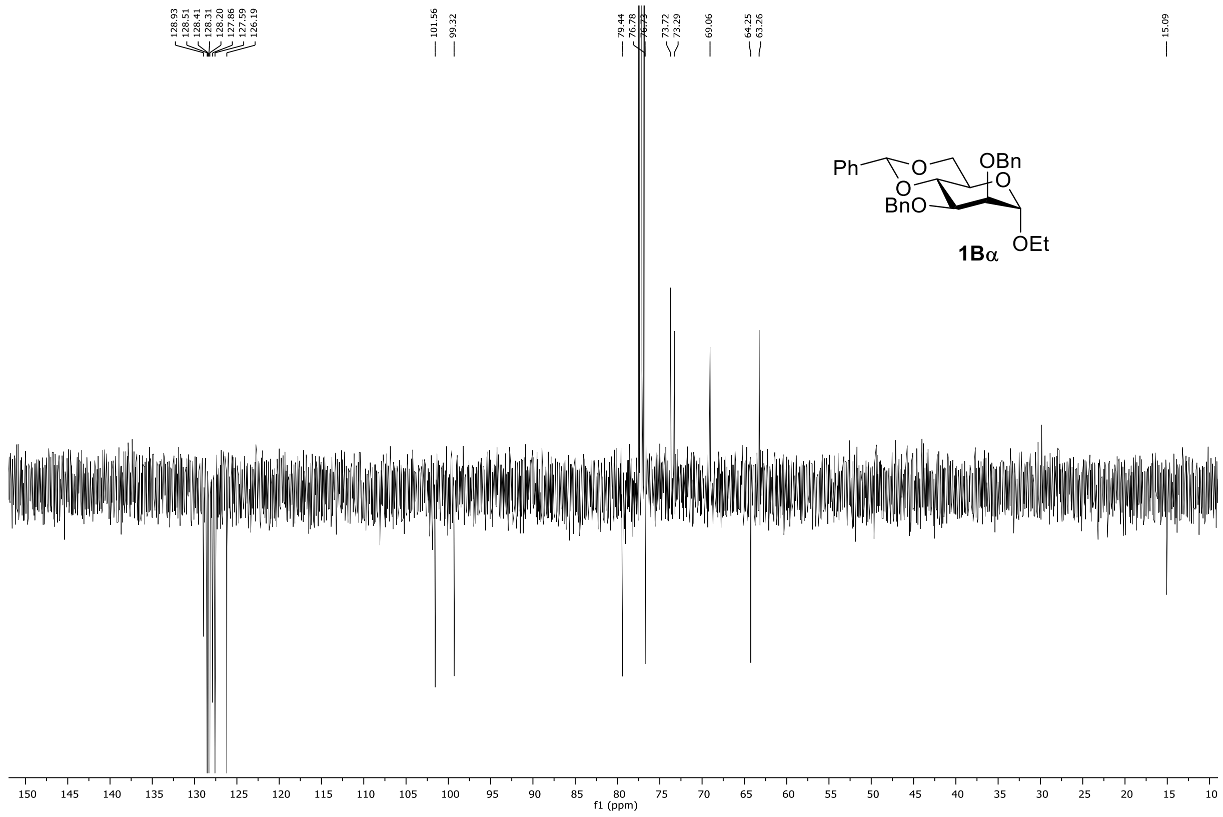

COSY NMR of compound **1B**

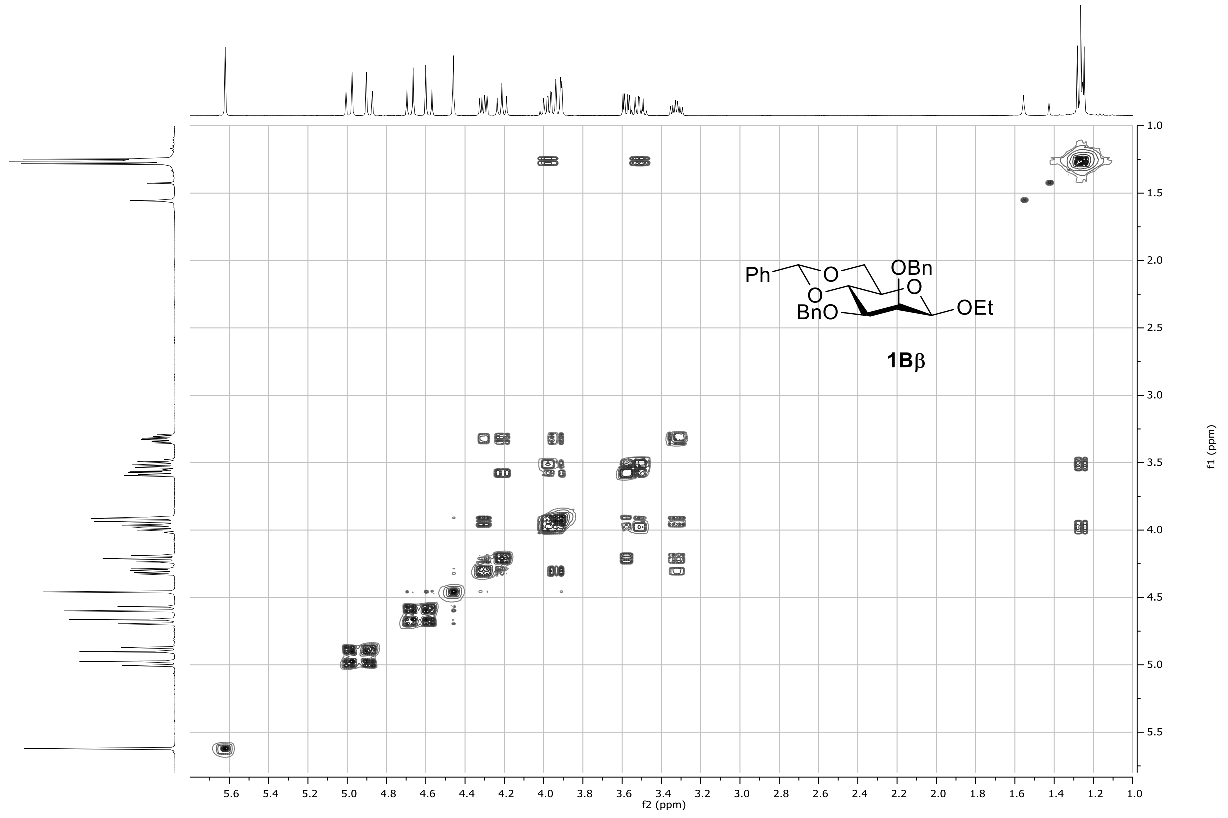

HSQC NMR of compound **1B**

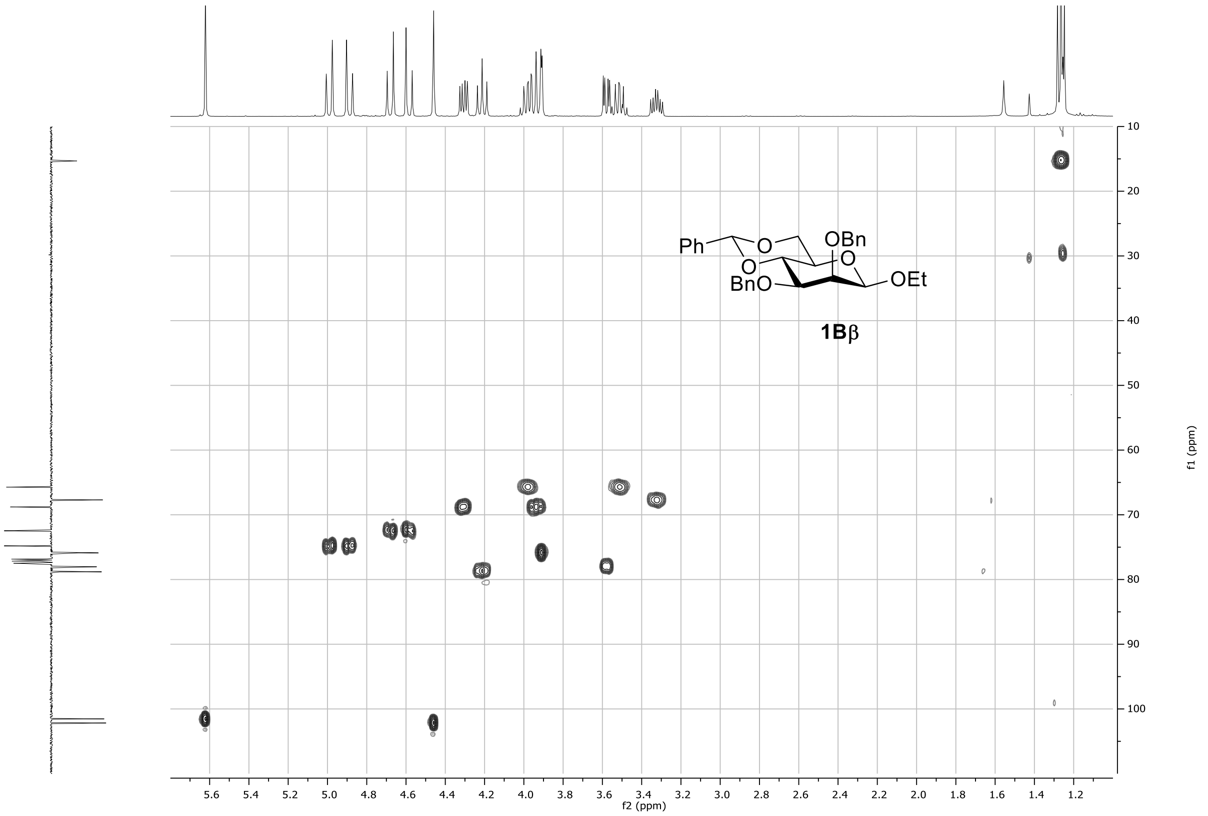

^{13}C GATED NMR, 101 MHz, $CDCl_3$ of compound **1B**

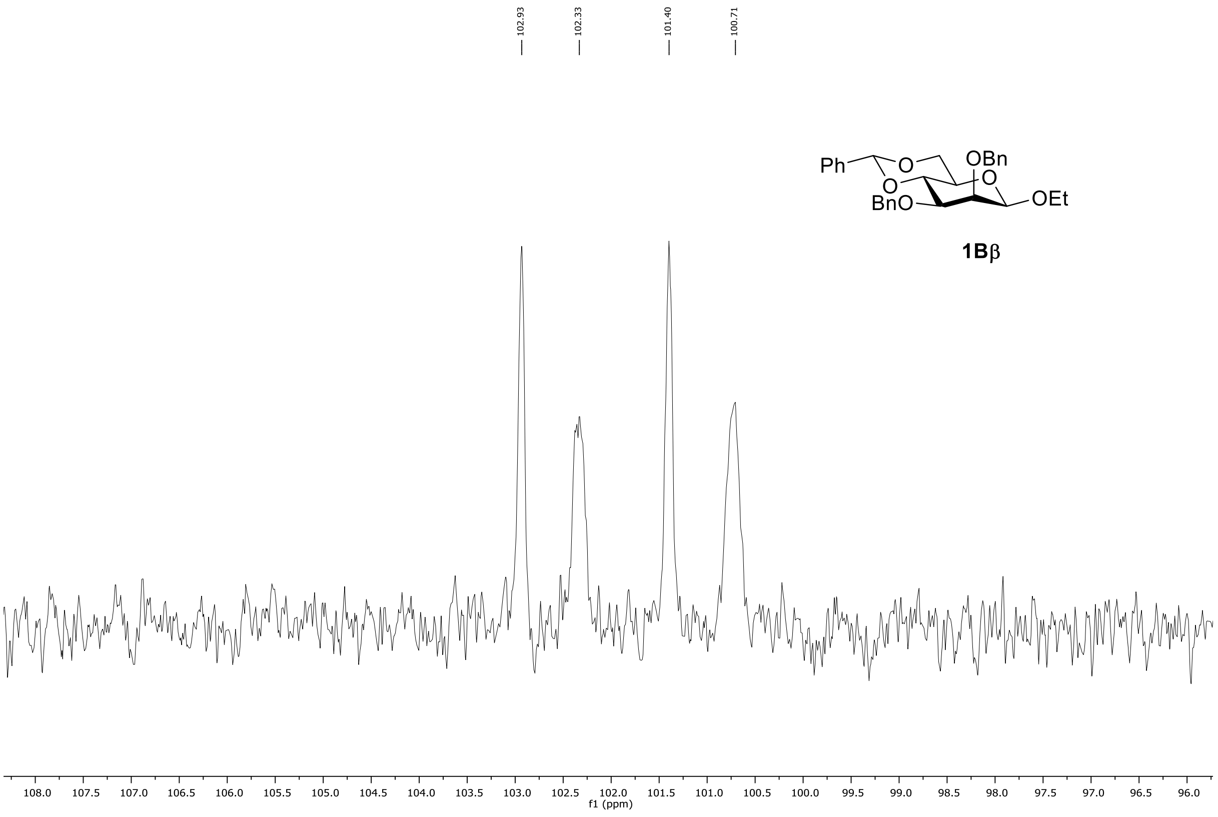

^{1}H NMR, 400 MHz, $CDCl_3$ of compound **1C**

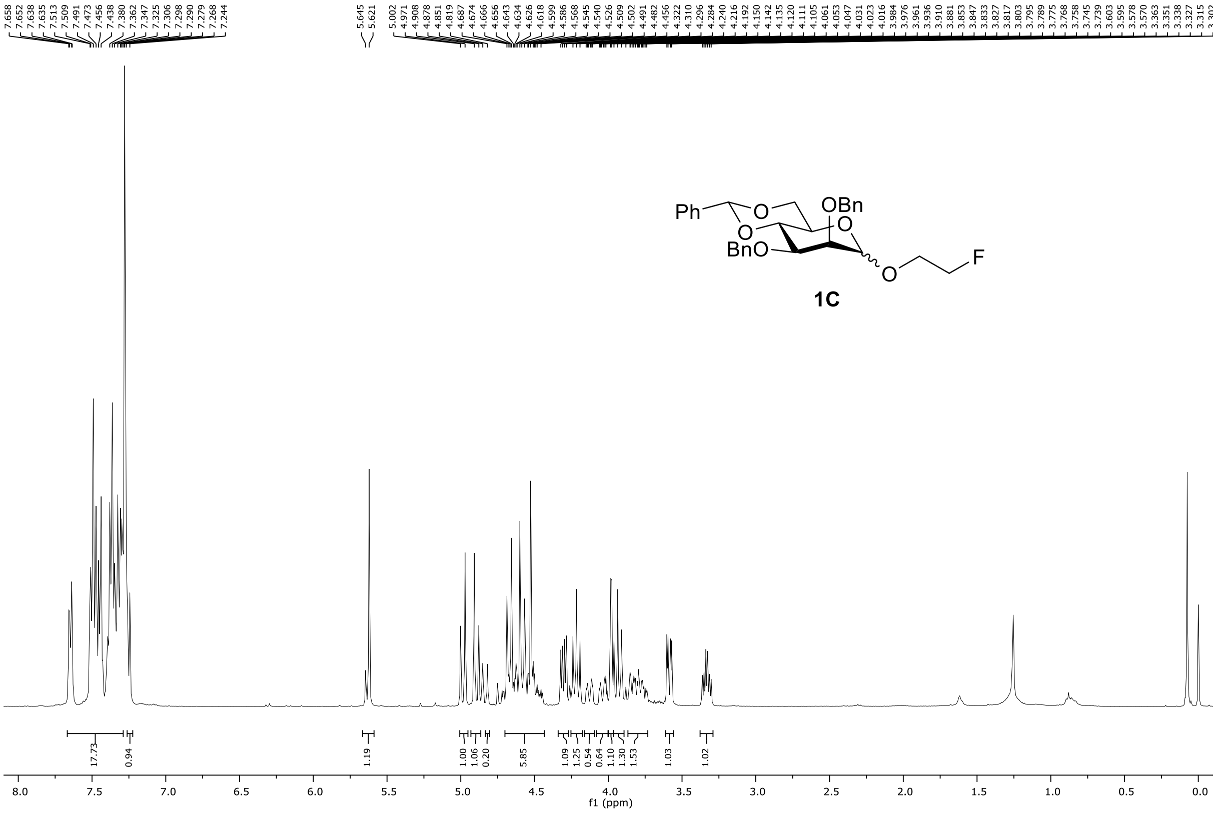

^{13}C-APT NMR, 101 MHz, $CDCl_3$ of compound **1C**

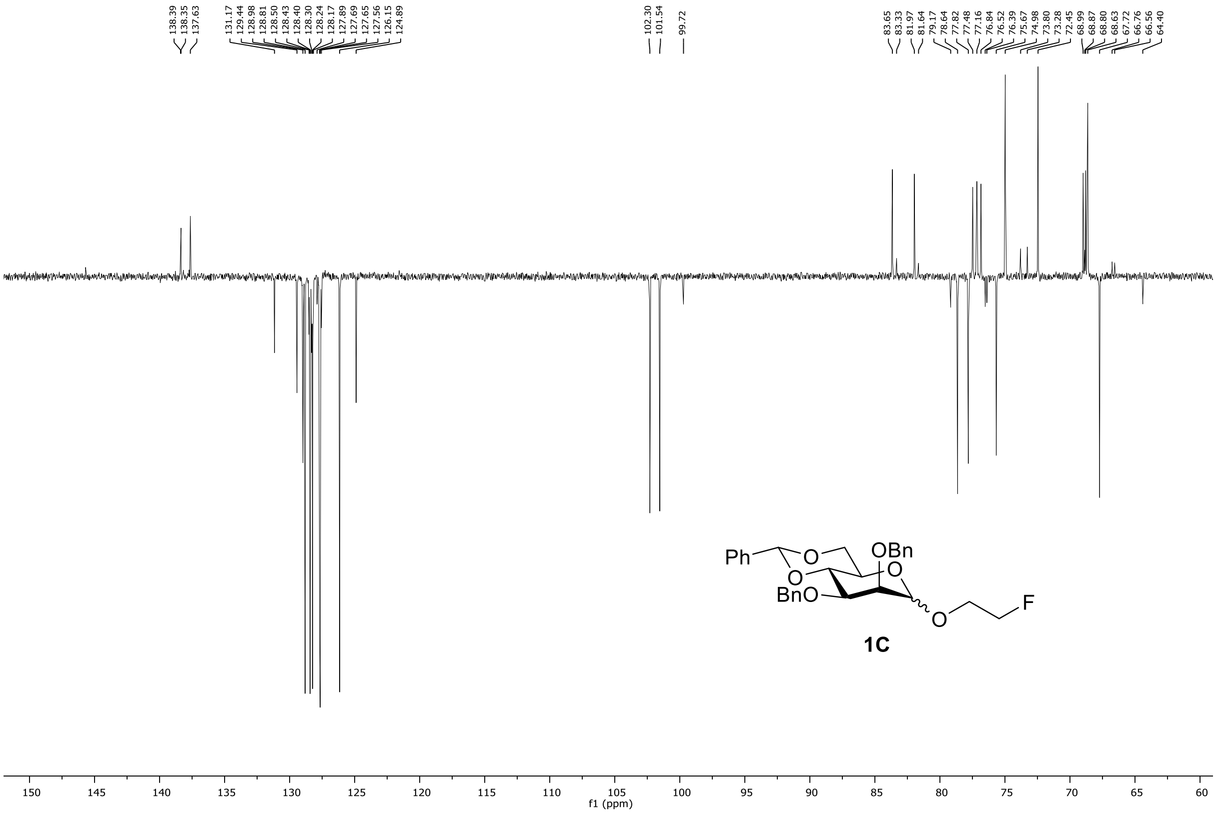

COSY NMR of compound **1C**

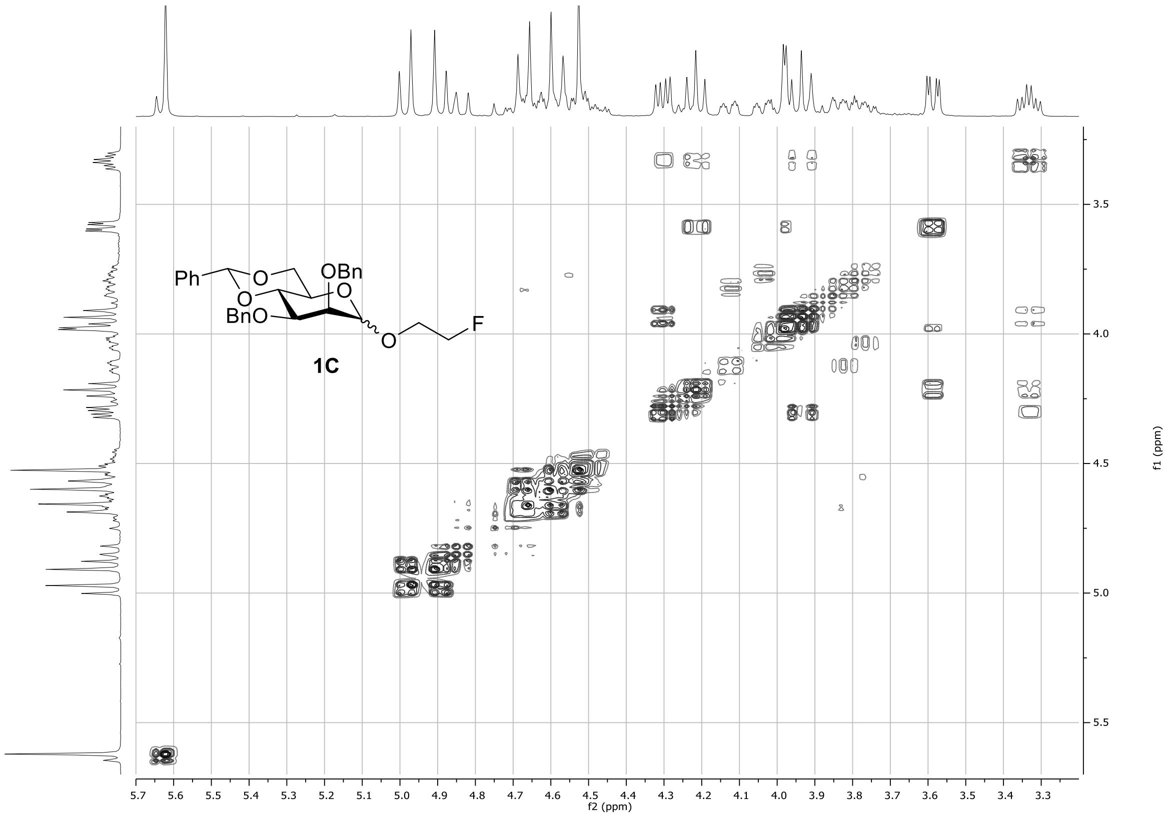

HSQC NMR of compound **1C**

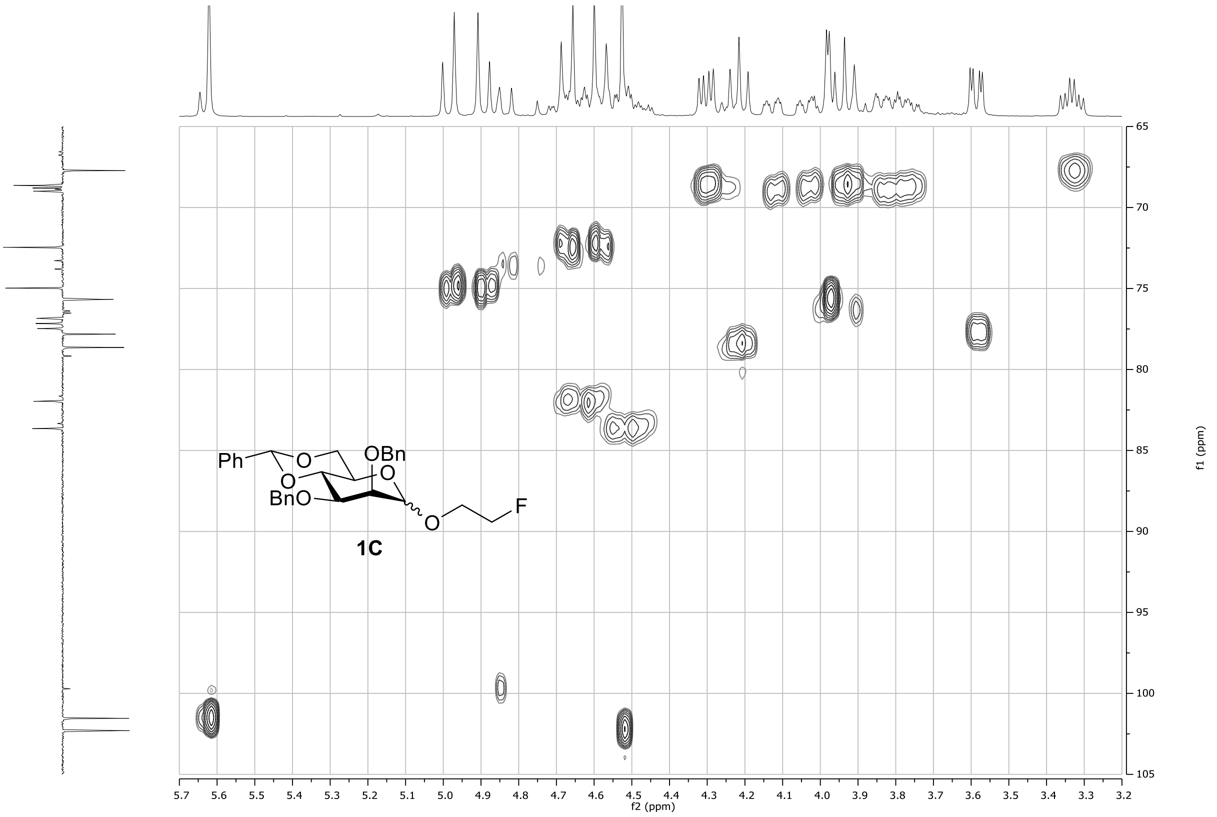

^{13}C GATED NMR, 101 MHz, $CDCl_3$ of compound **1C**

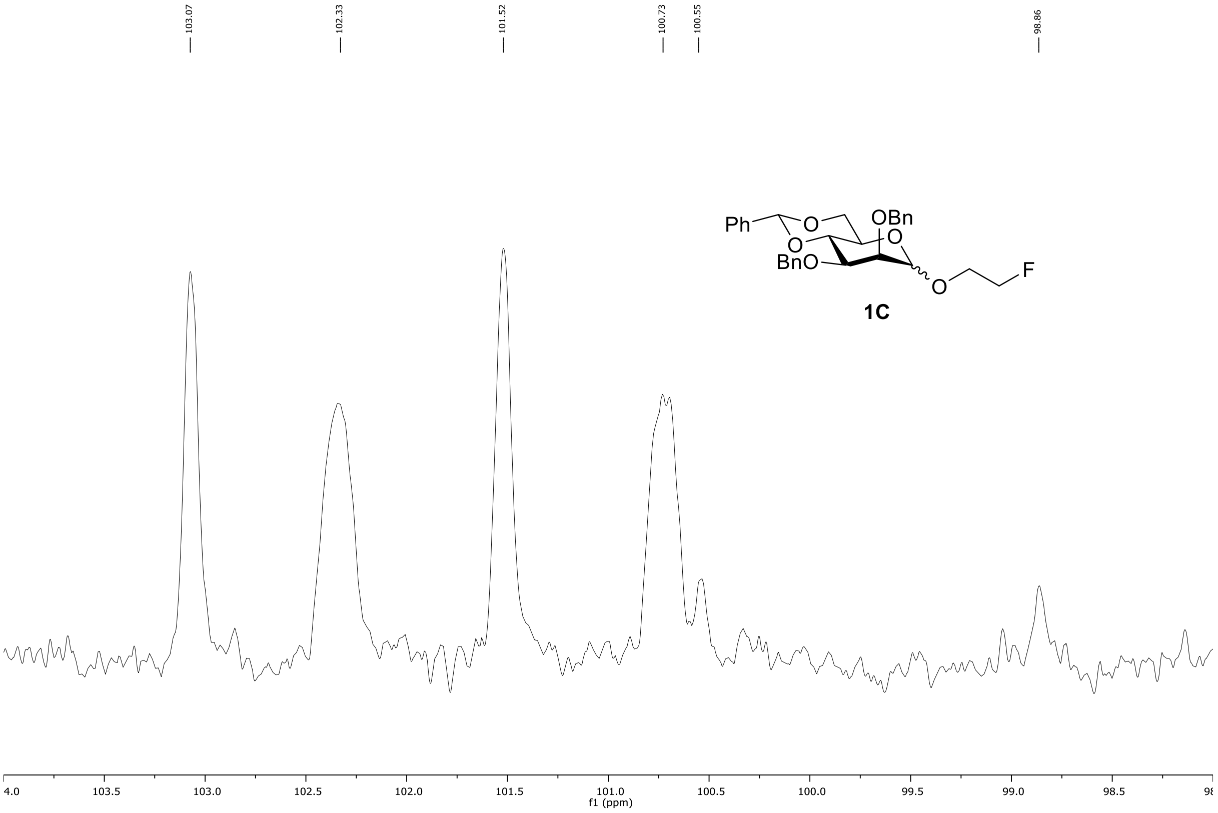

^{1}H NMR, 400 MHz, $CDCl_3$ of compound **1D**

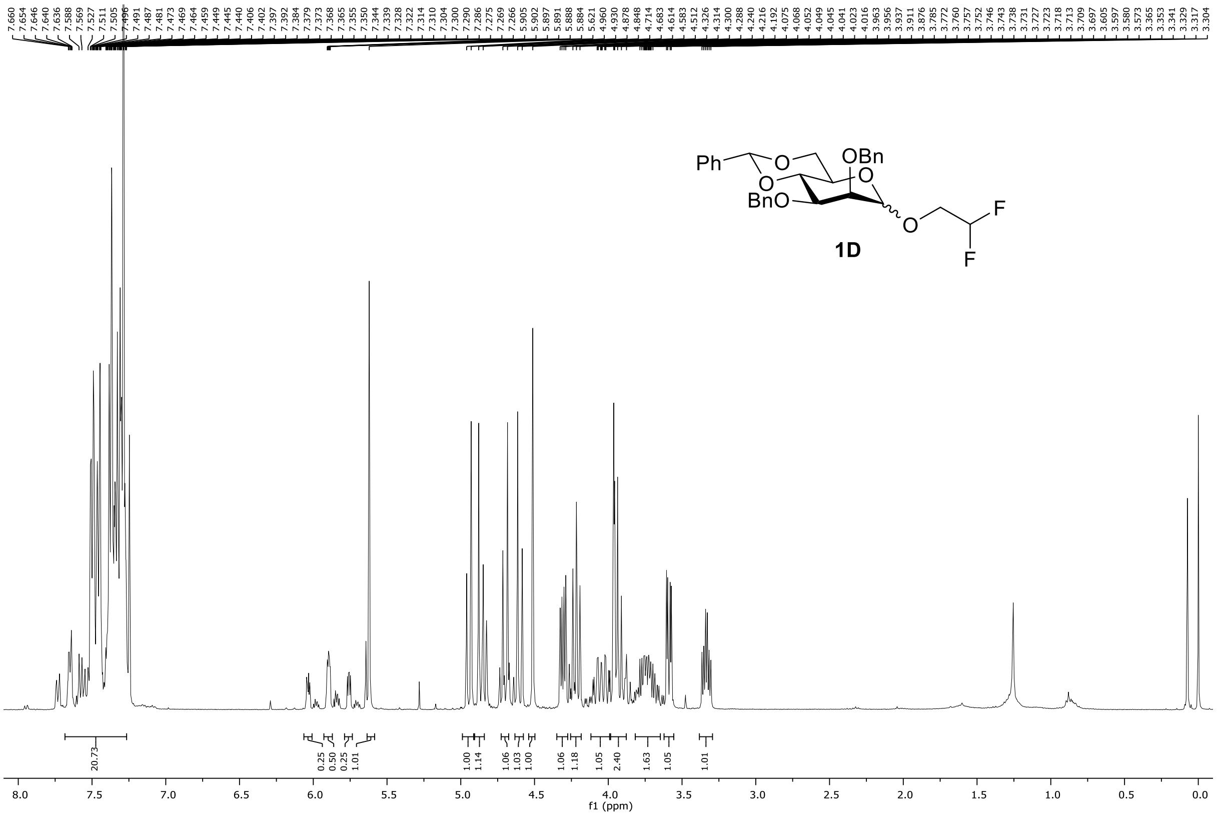

^{13}C-APT NMR, 101 MHz, $CDCl_3$ of compound **1D**

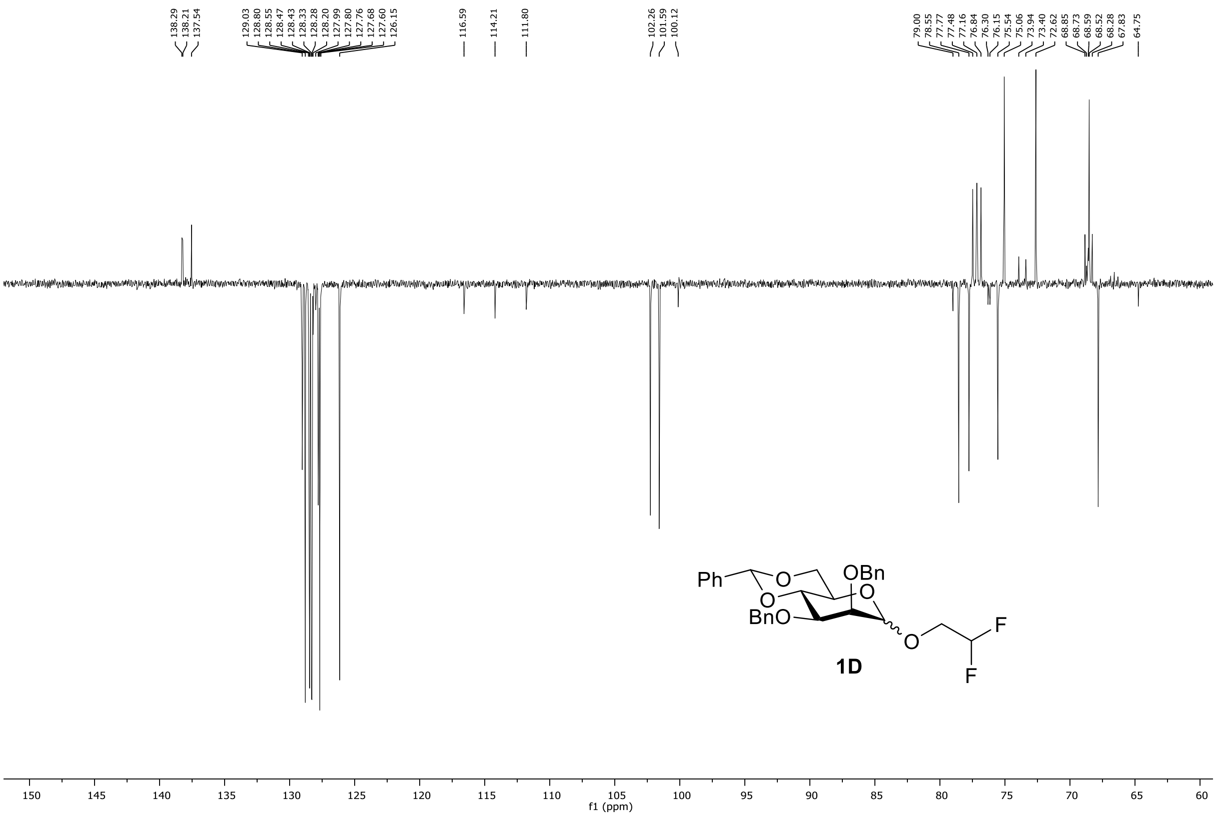

COSY NMR of compound **1D**

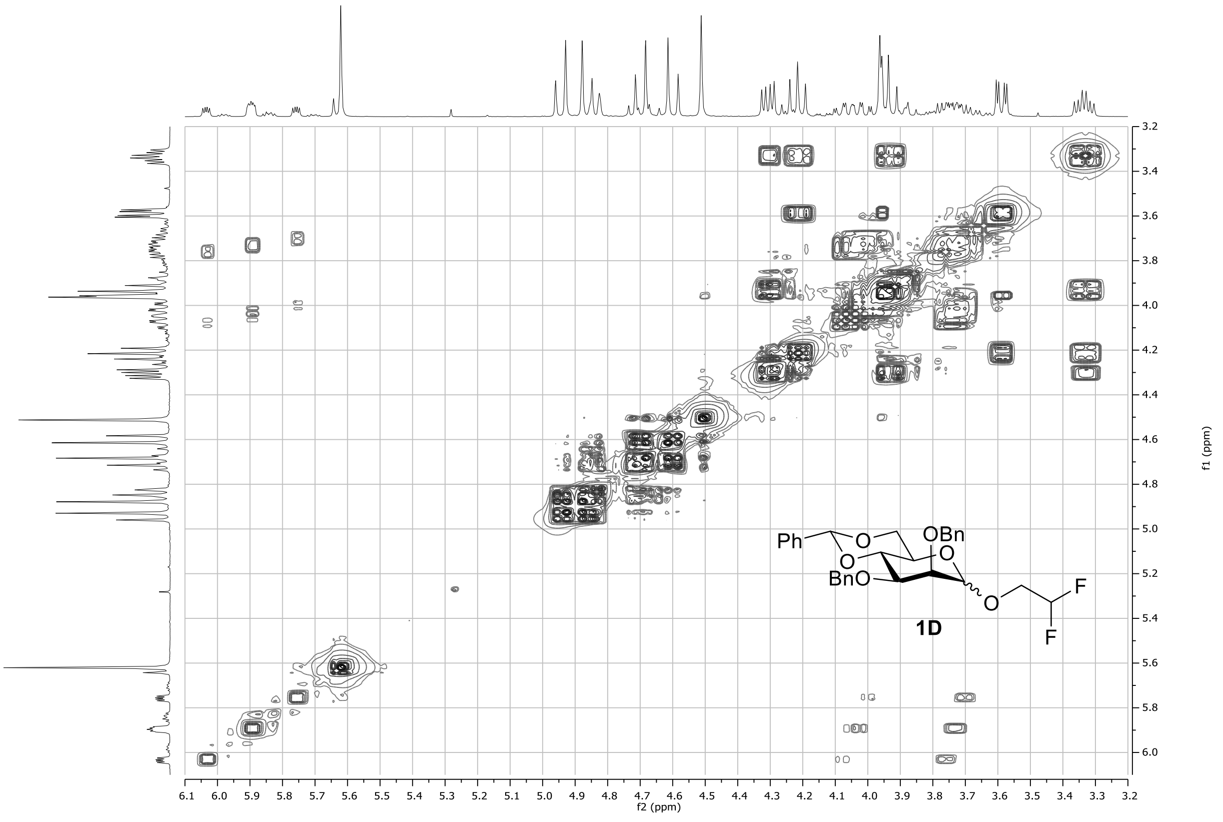

HSQC NMR of compound **1D**

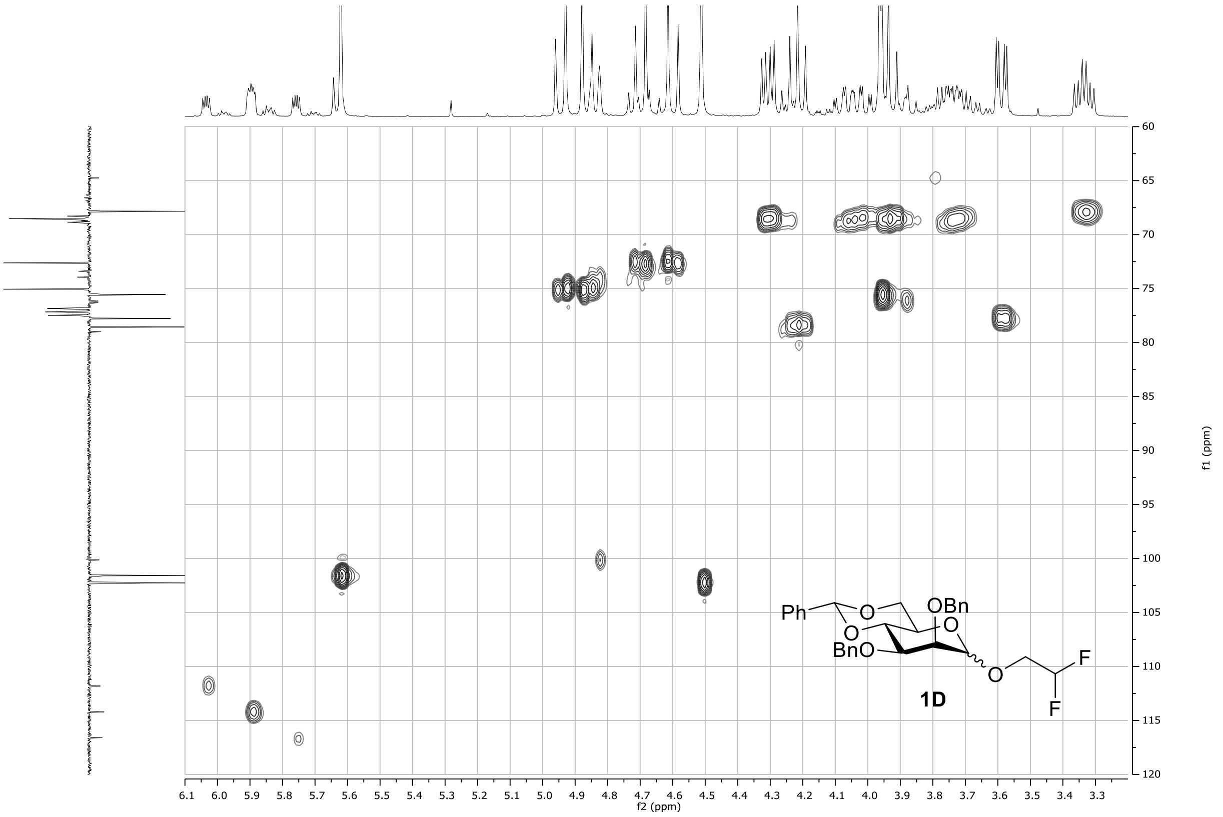

13C GATED NMR, 101 MHz, $CDCl_3$ of compound **1D**

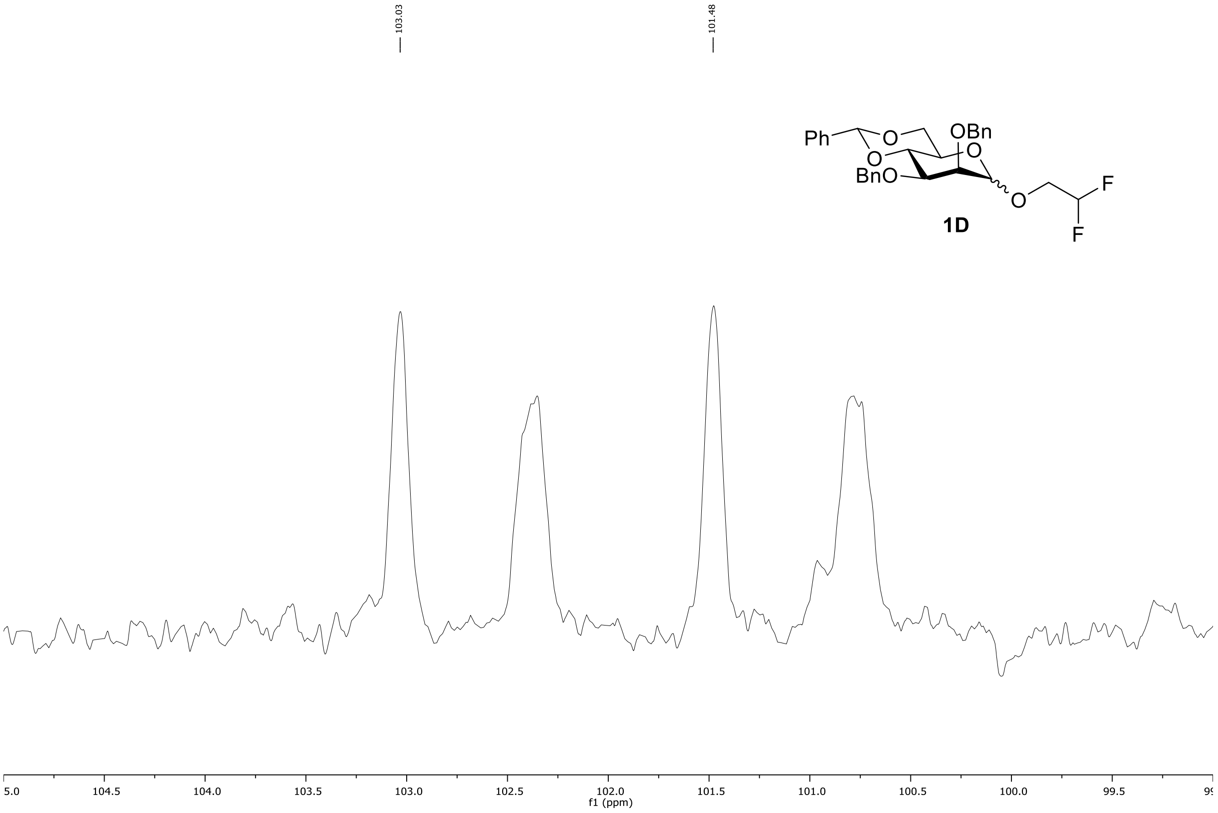

^{1}H NMR, 400 MHz, $CDCl_3$ of compound **1E**

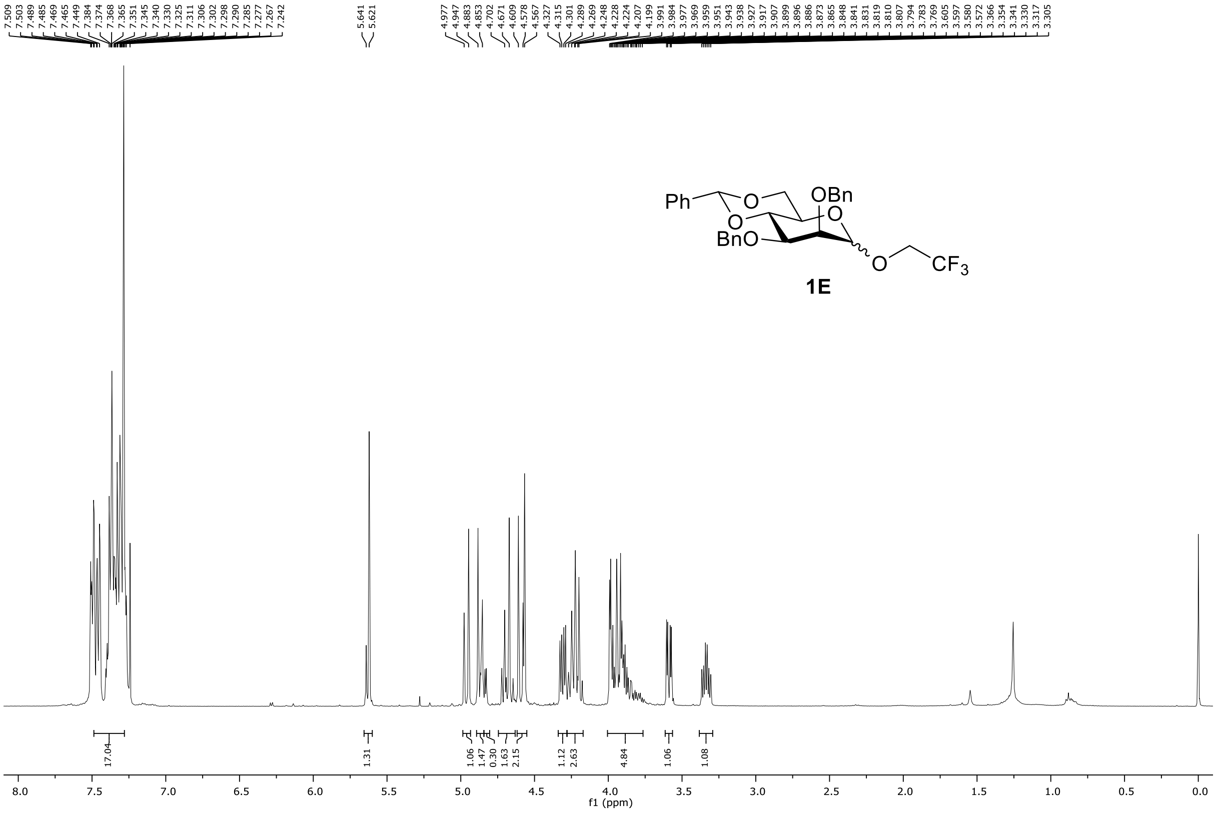

^{13}C-APT NMR, 101 MHz, $CDCl_3$ of compound **1D**

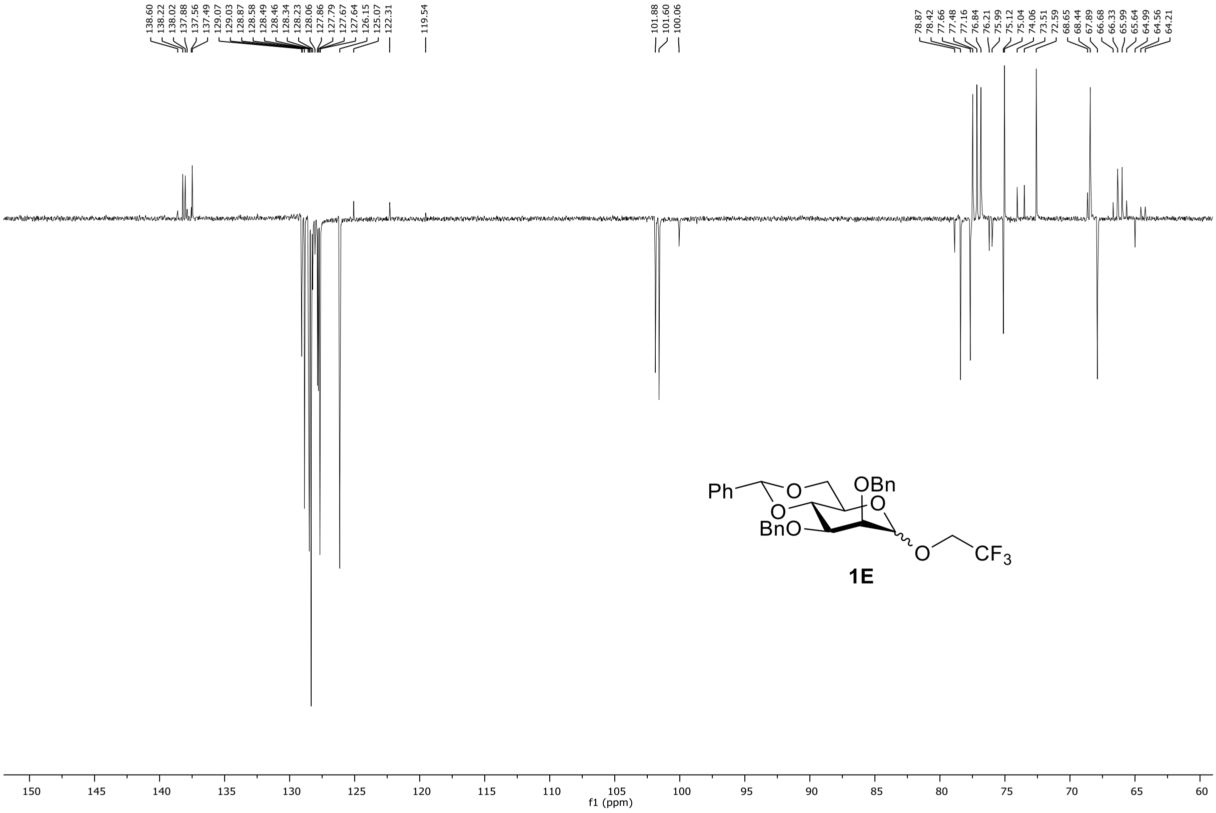

COSY NMR of compound **1E**

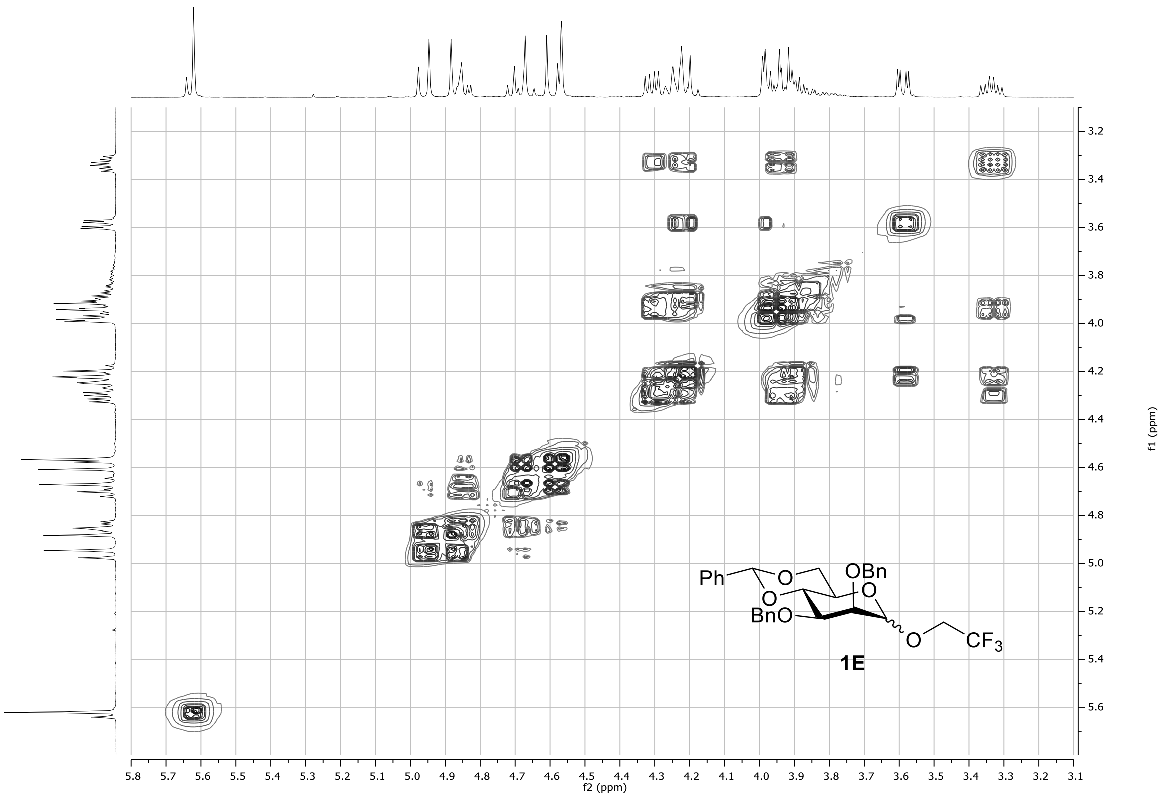

HSQC NMR of compound **1E**

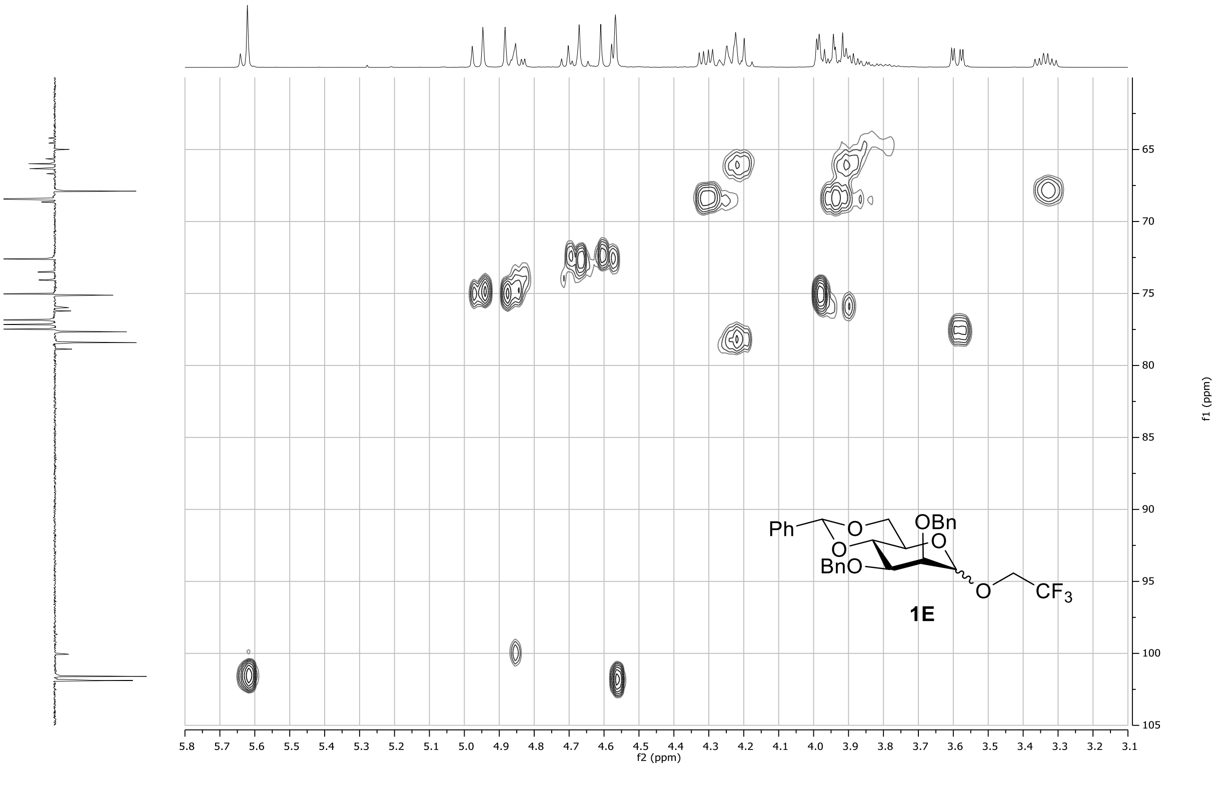

HMBC NMR of compound **1E**

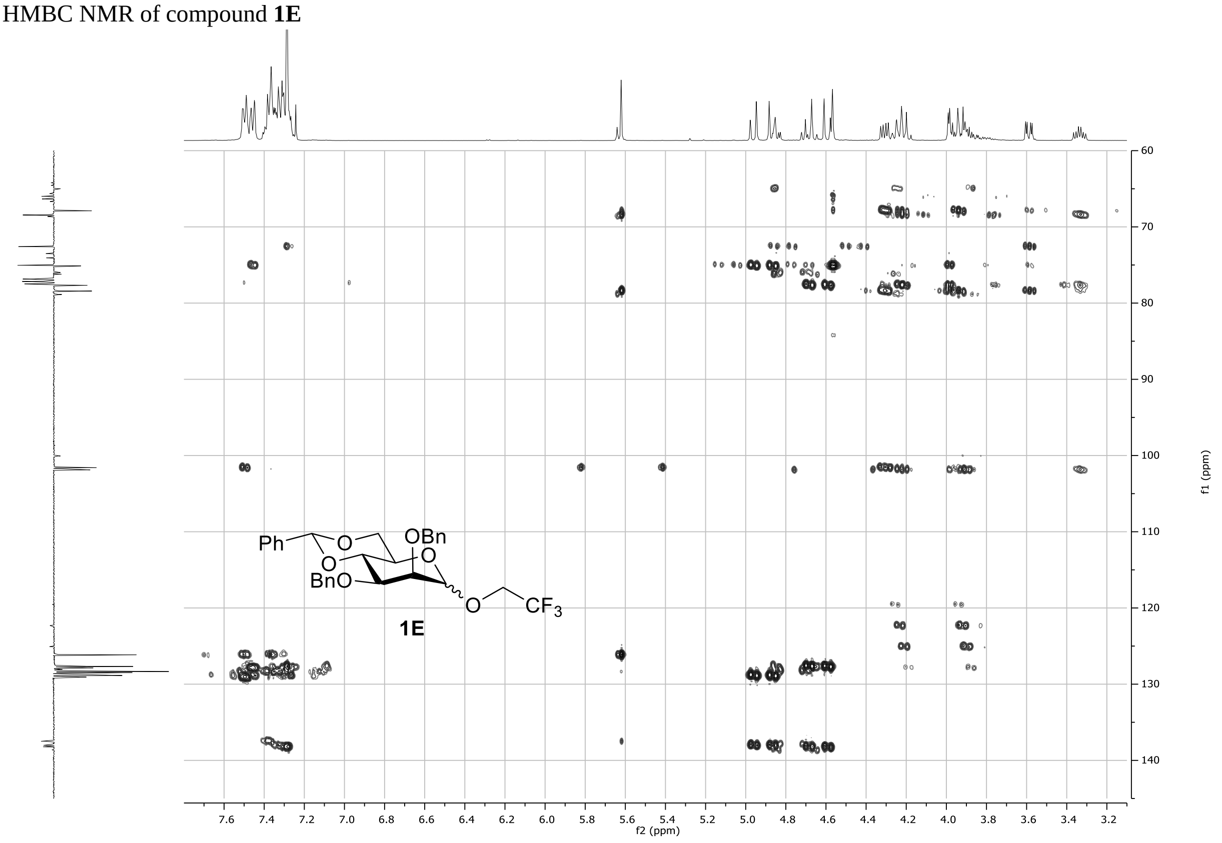

^{13}C GATED NMR, 101 MHz, $CDCl_3$ of compound **1E**

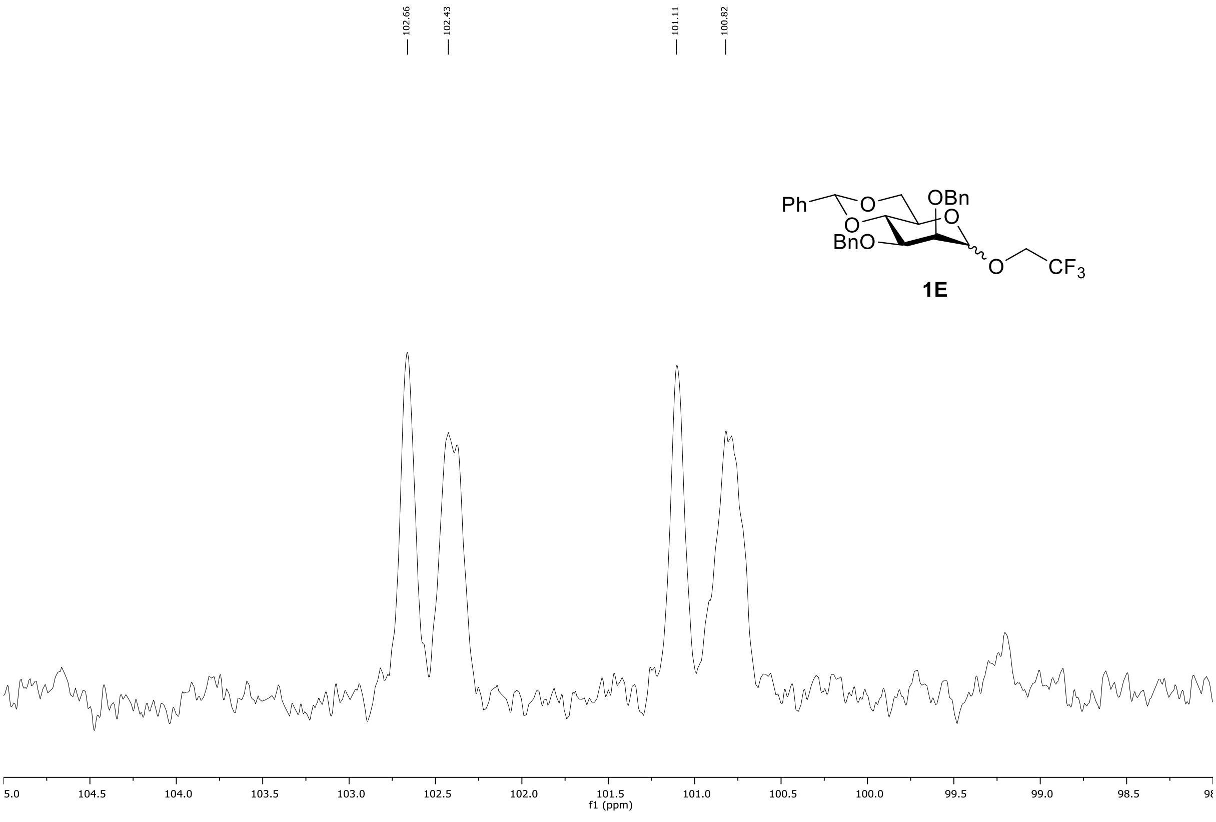

^{1}H NMR, 400 MHz, CDCl3 of compound **1F**

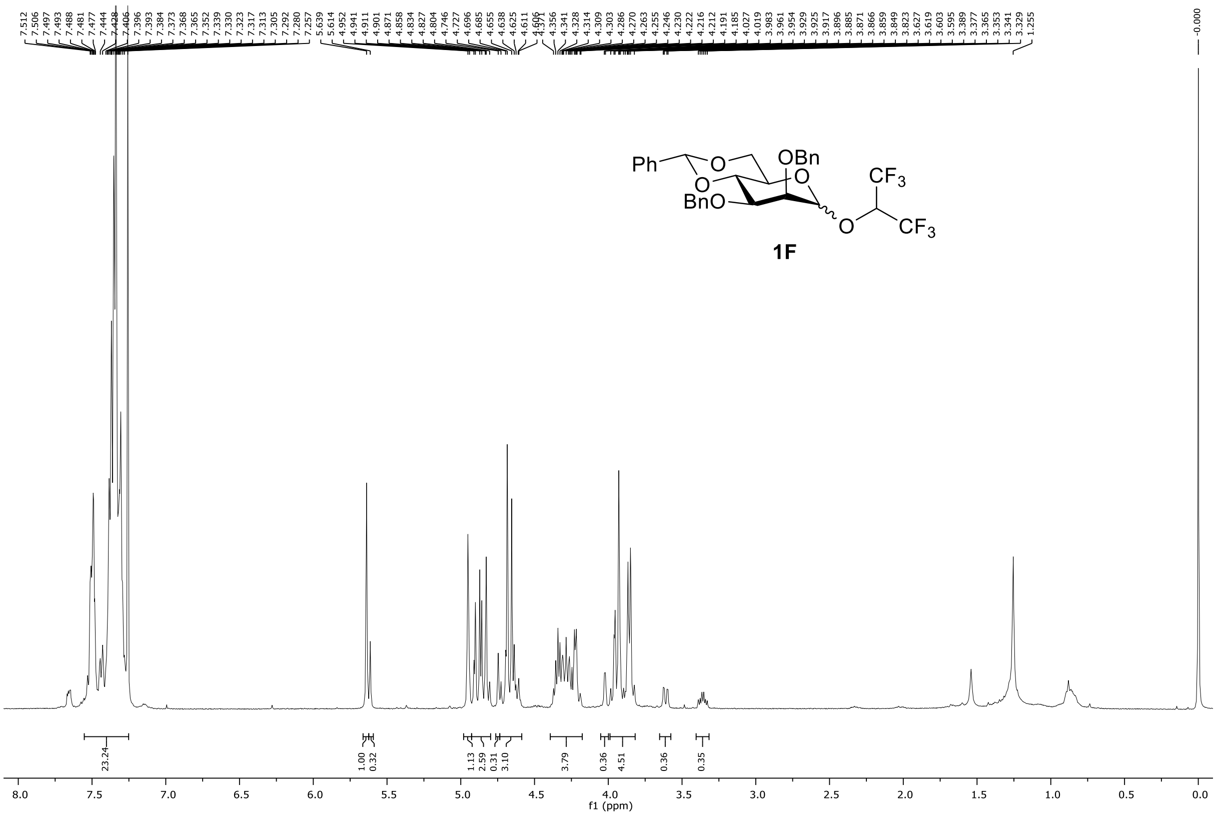

^{13}C-APT NMR, 101 MHz, CDCl3 of compound **1F**

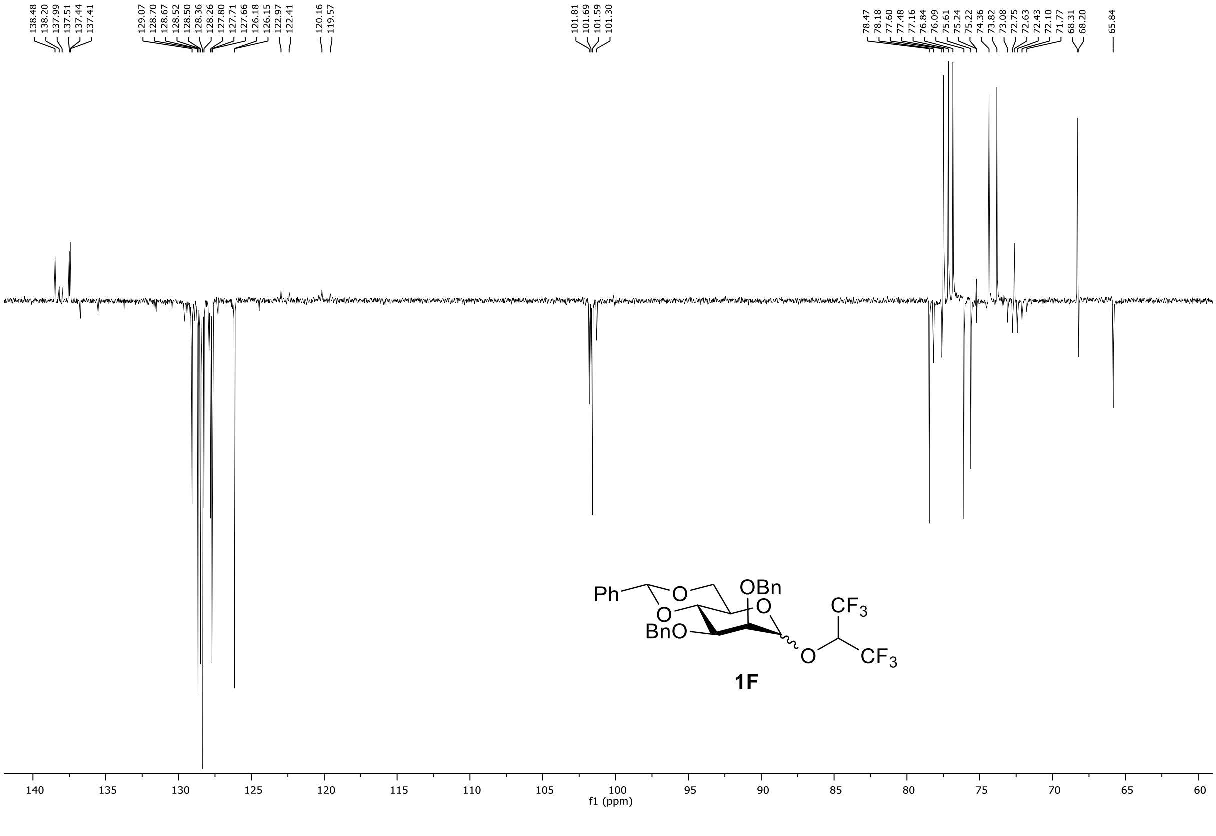

COSY NMR of compound **1F**

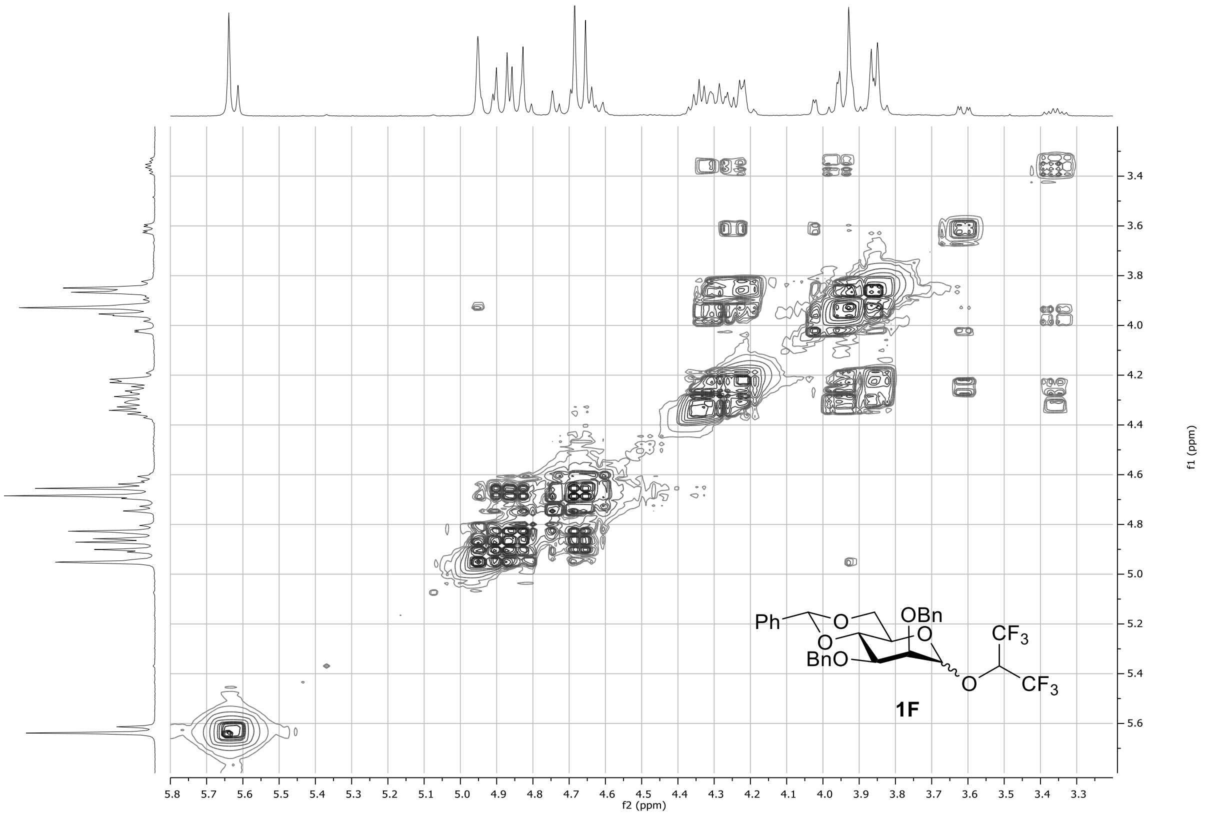

HSQC NMR of compound **1F**

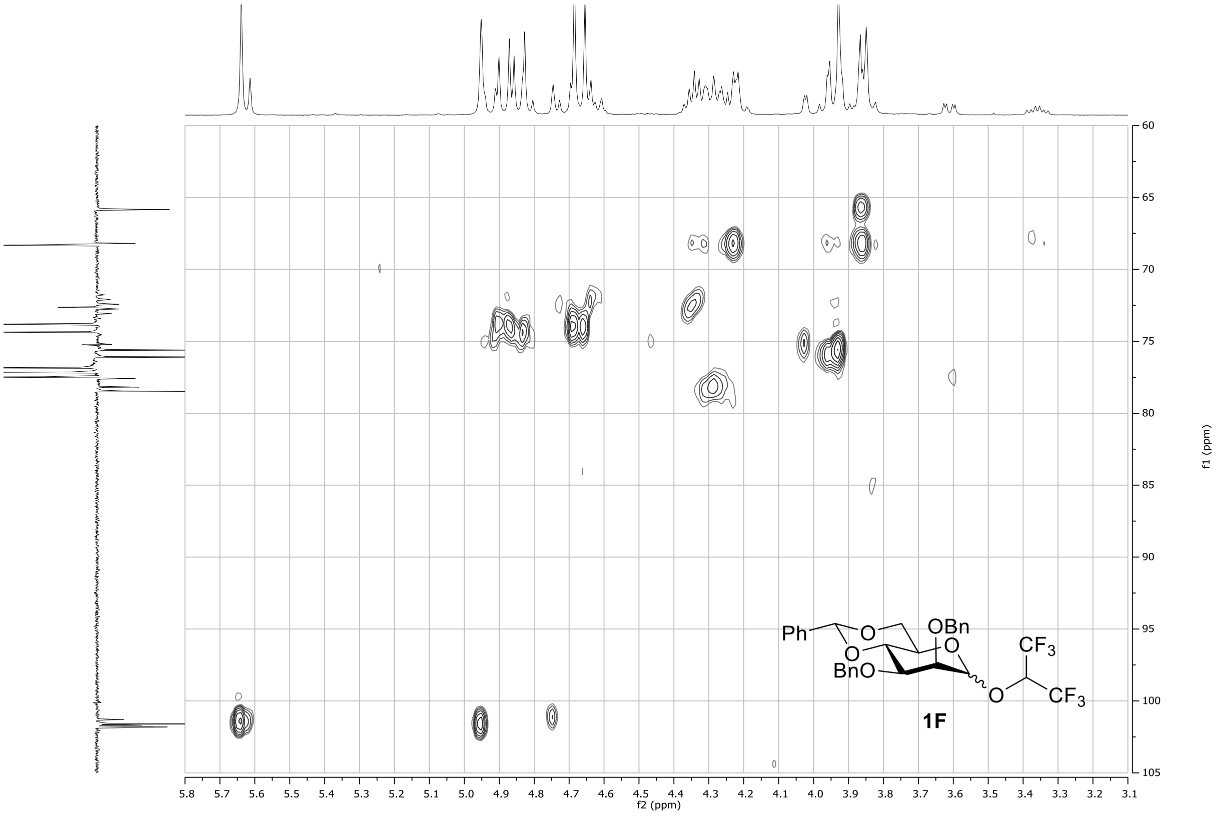

HMBC NMR of compound **1F**

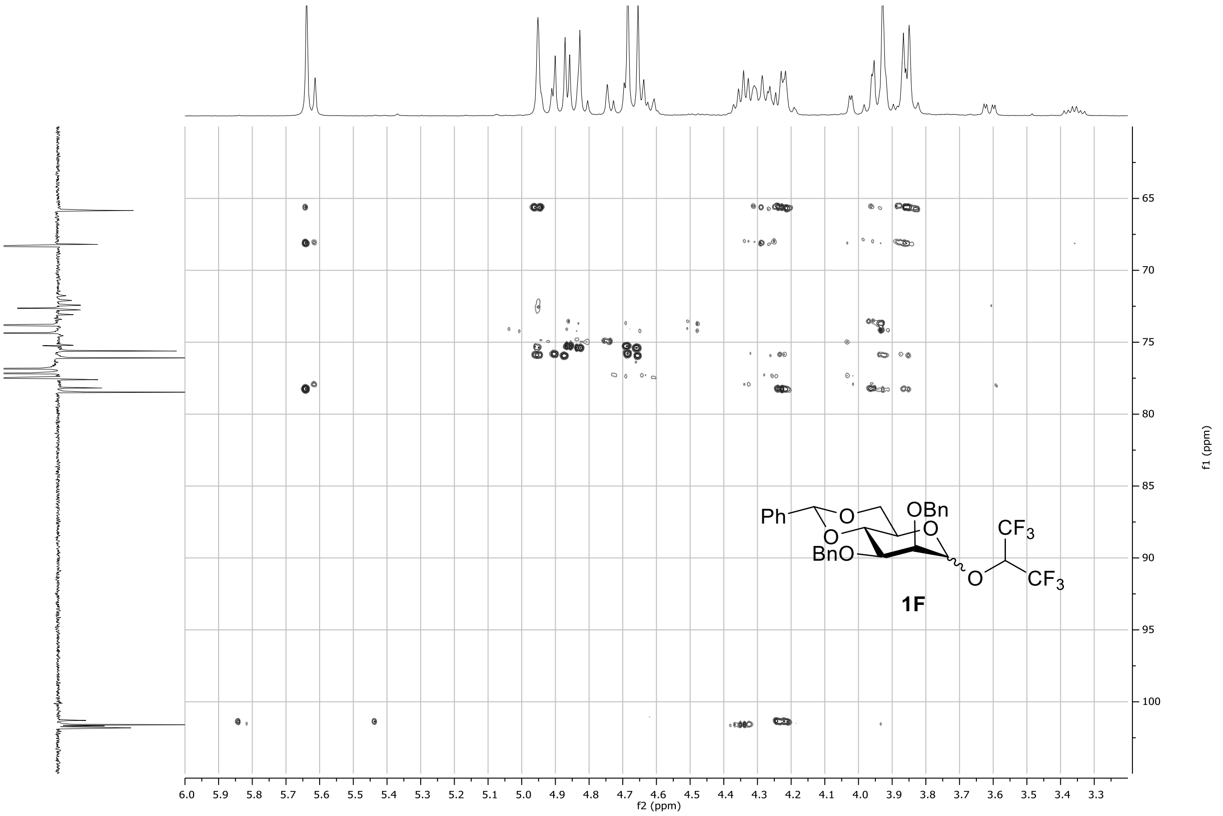

HMBC-GATED NMR of compound **1F**

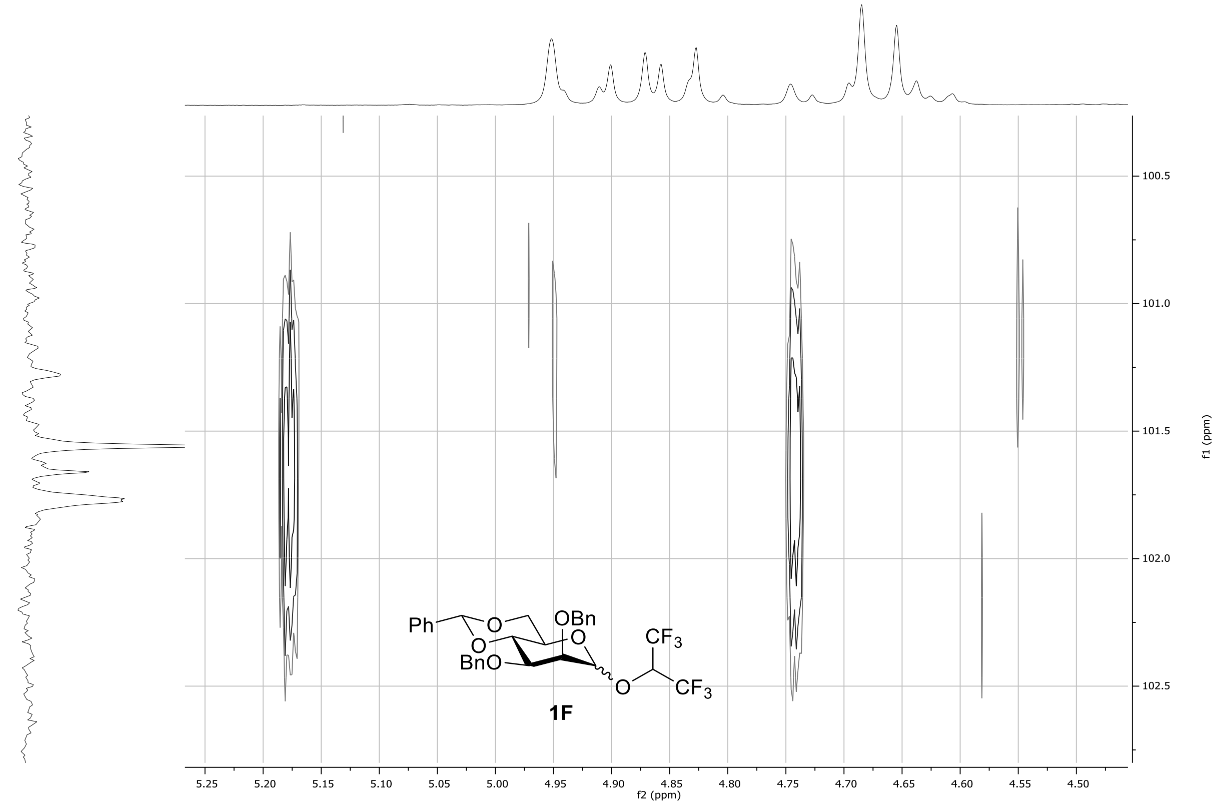

^{1}H NMR, 400 MHz, $CDCl_3$ of compound **1G**

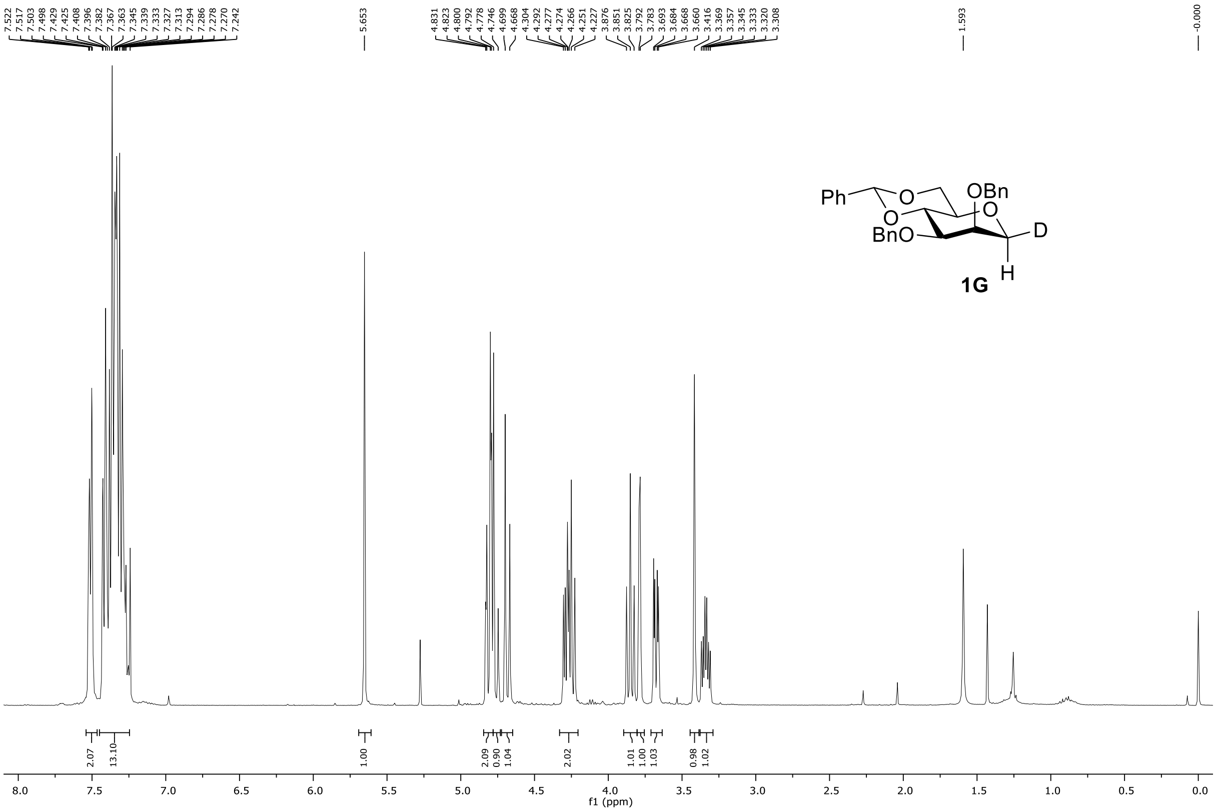

^{13}C-APT NMR, 101 MHz, $CDCl_3$ of compound **1G**

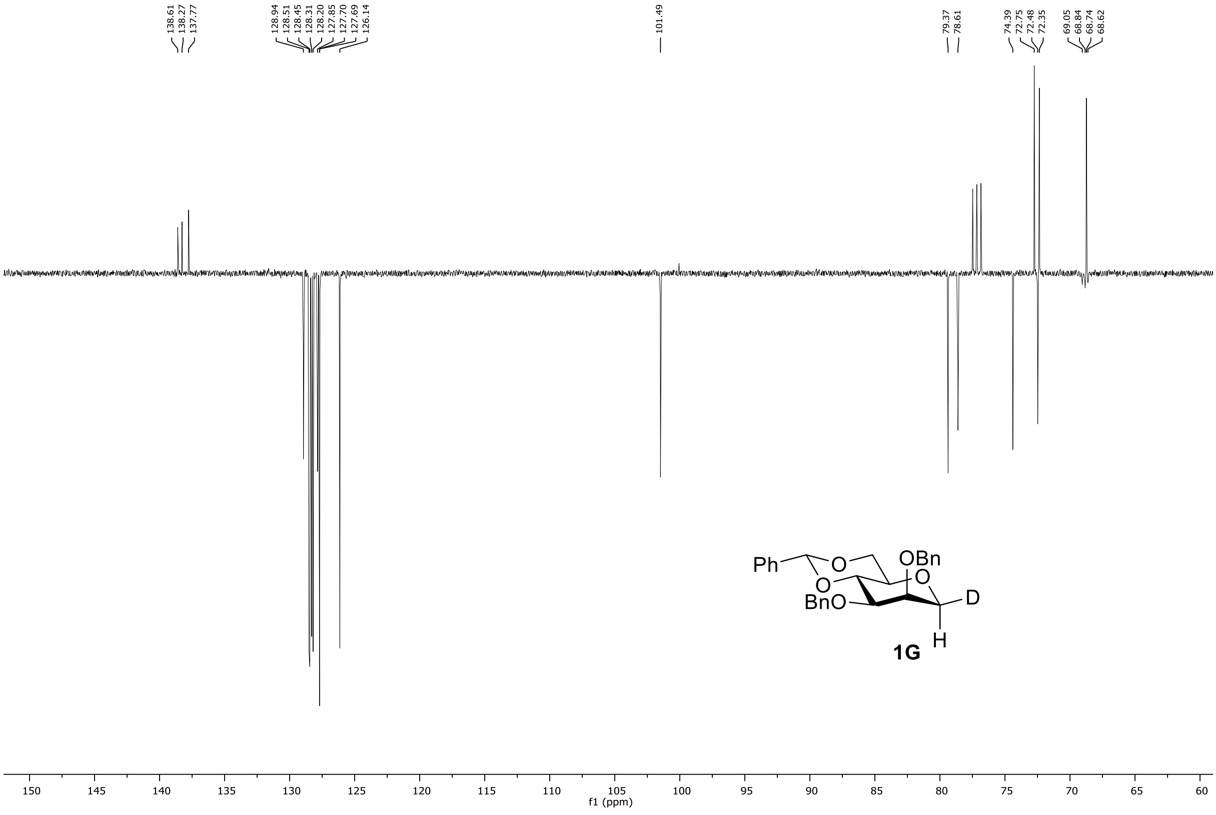

COSY NMR of compound **1G**

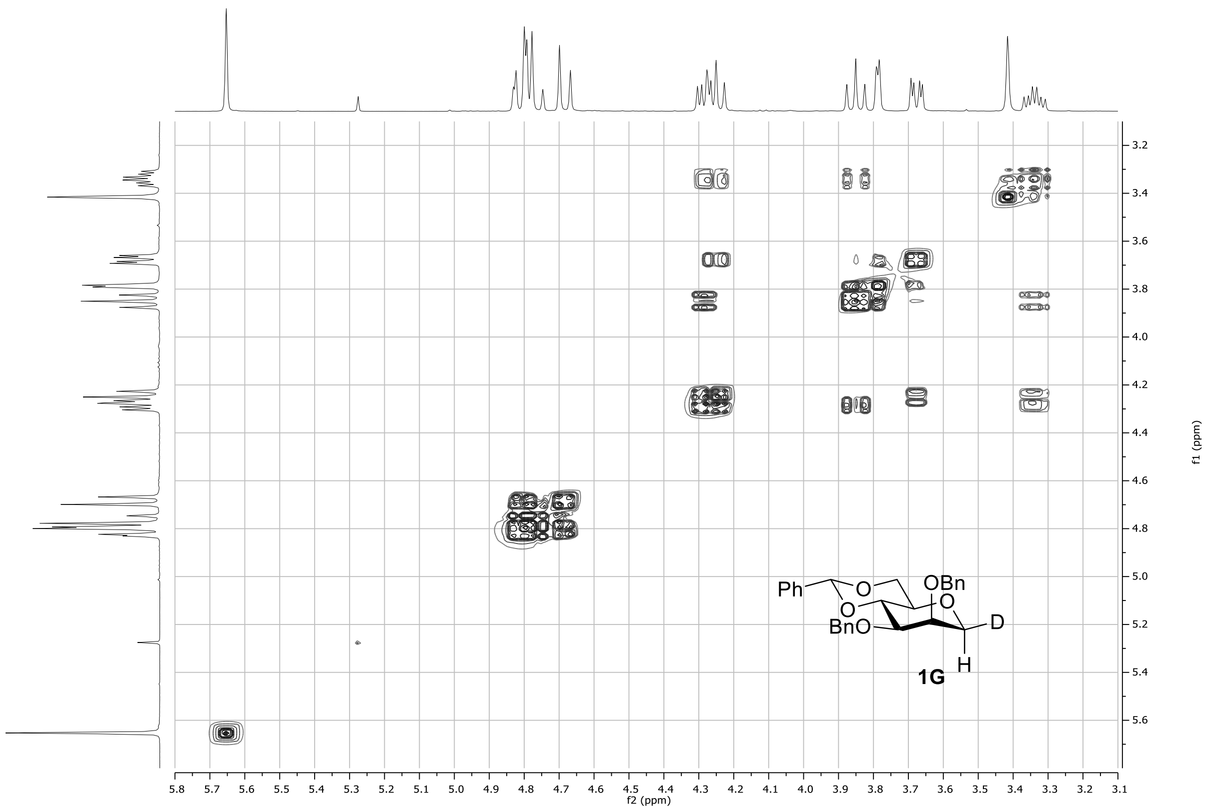

HSQC NMR of compound **1G**

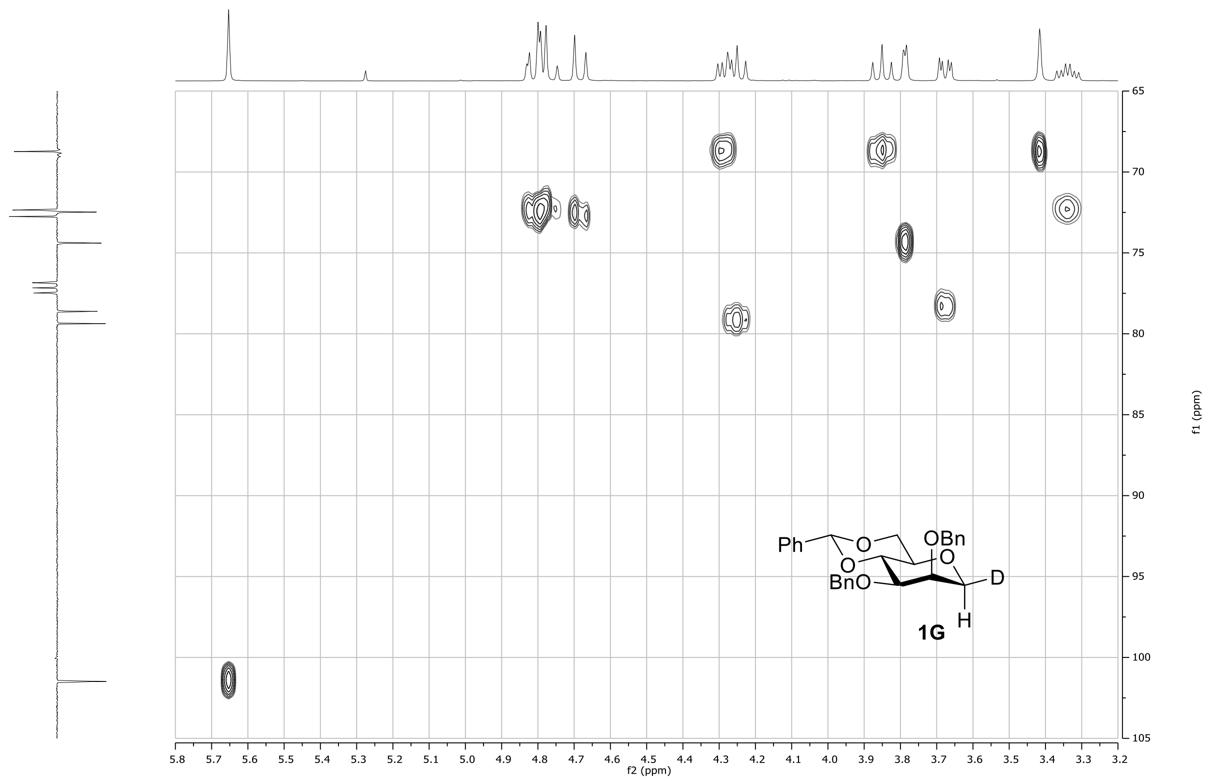

HMBC-GATED NMR of compound **1G**

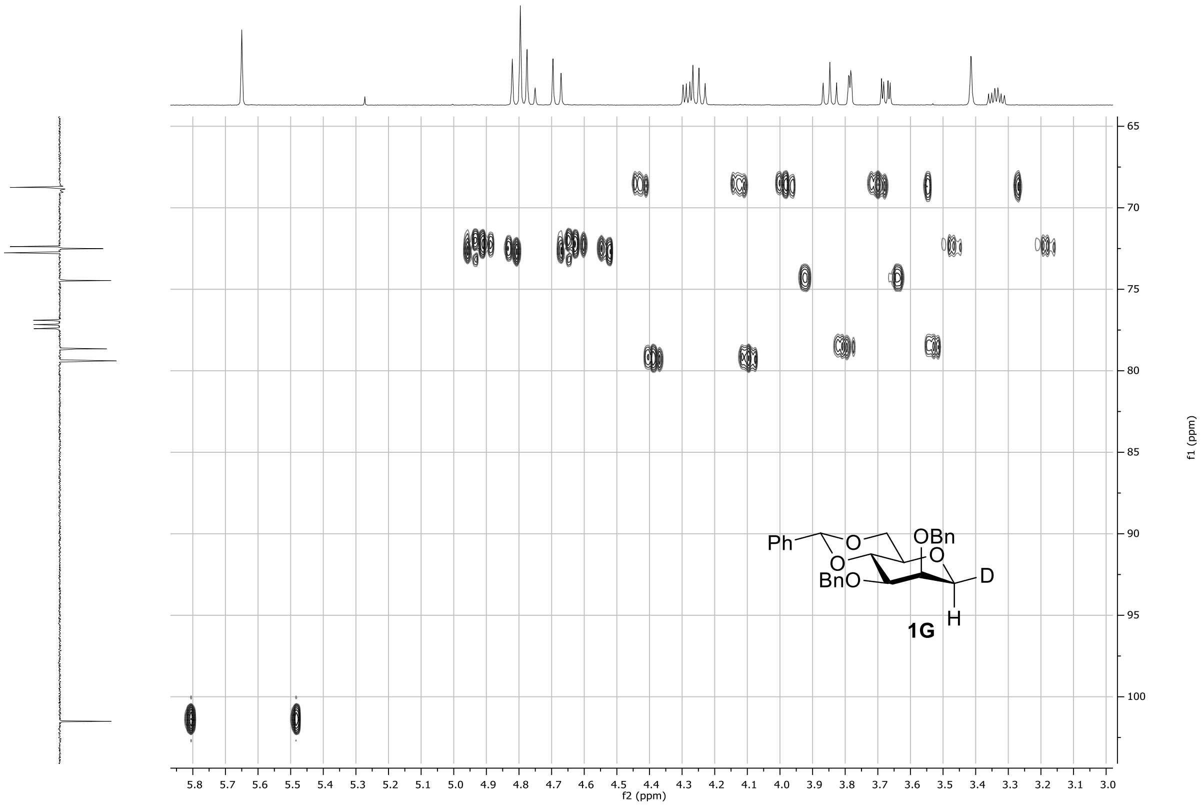

NOESY NMR of compound **1G**

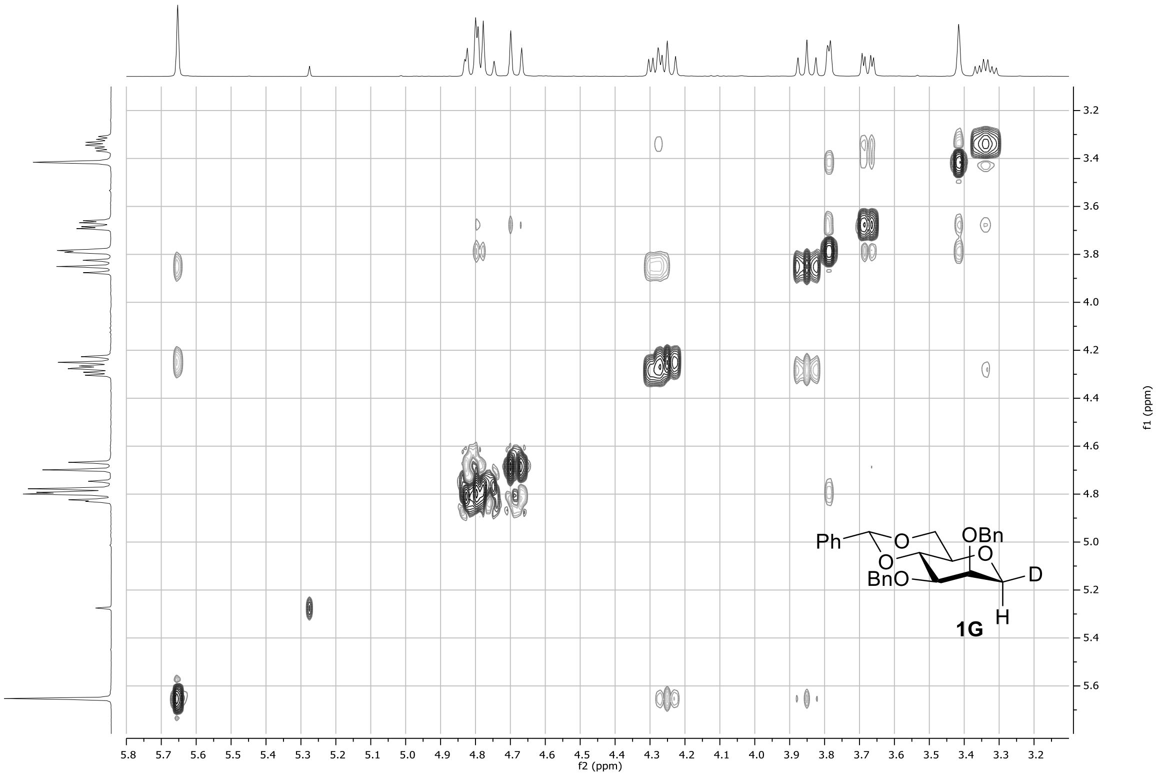

^{2}D NMR, 77 MHz, $CHCl_3$ of compound **1G**

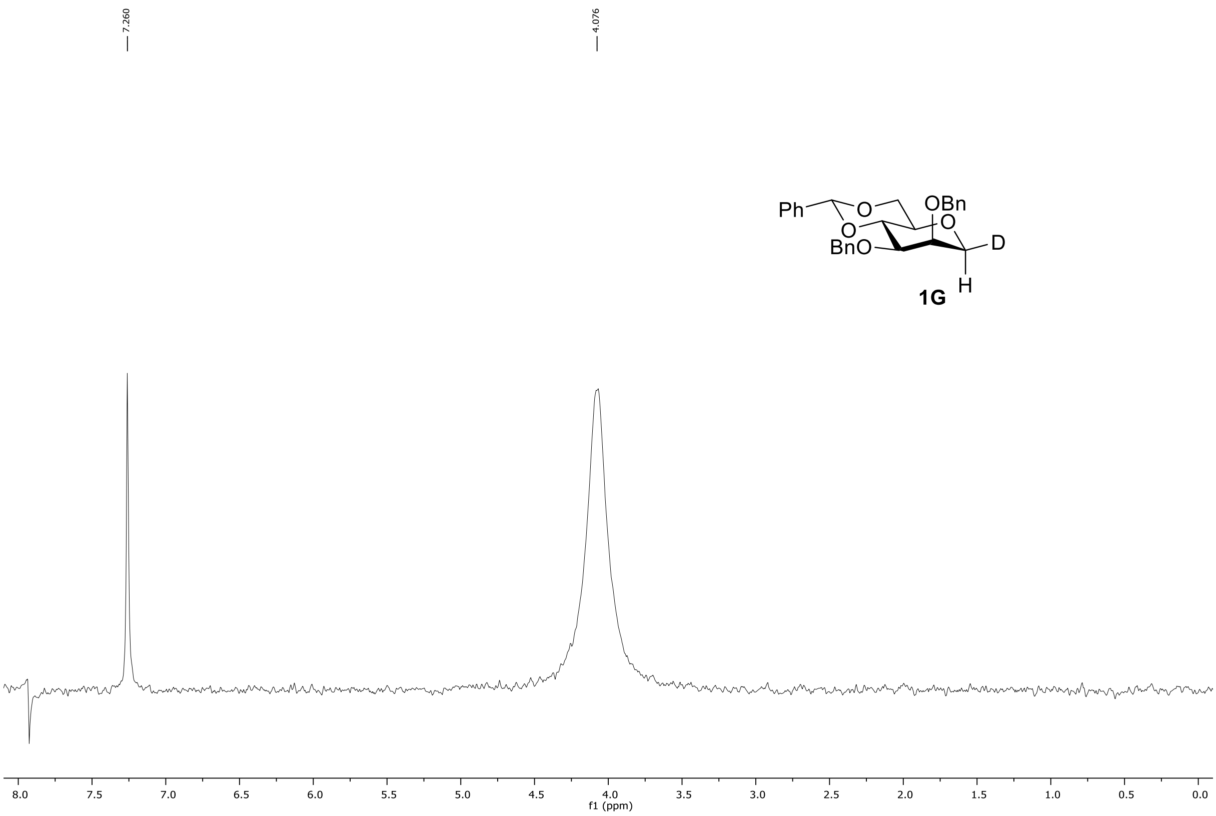

^{1}H NMR, 400 MHz, $CDCl_3$ of compound **22**

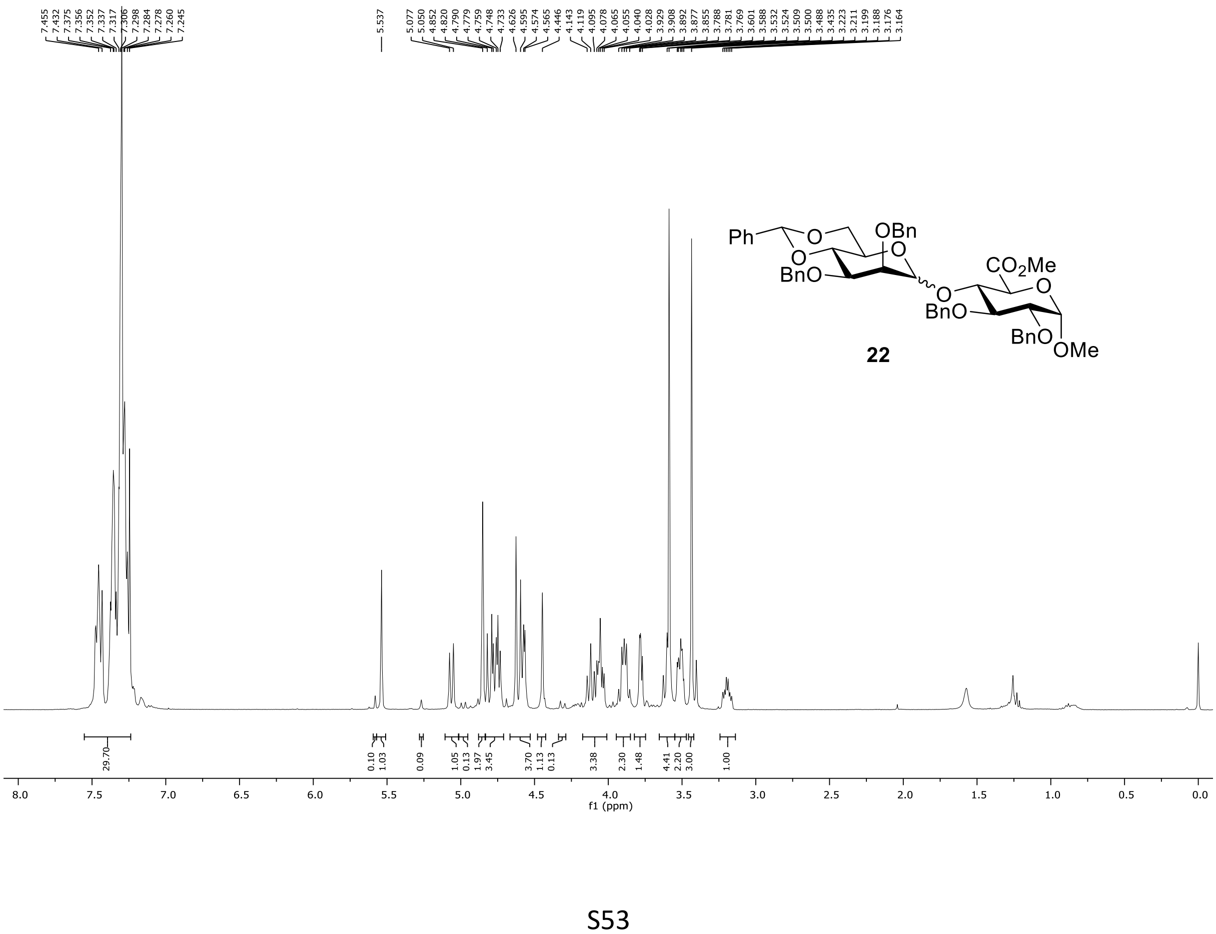

^{13}C-APT NMR, 101 MHz, $CDCl_3$ of compound **22**

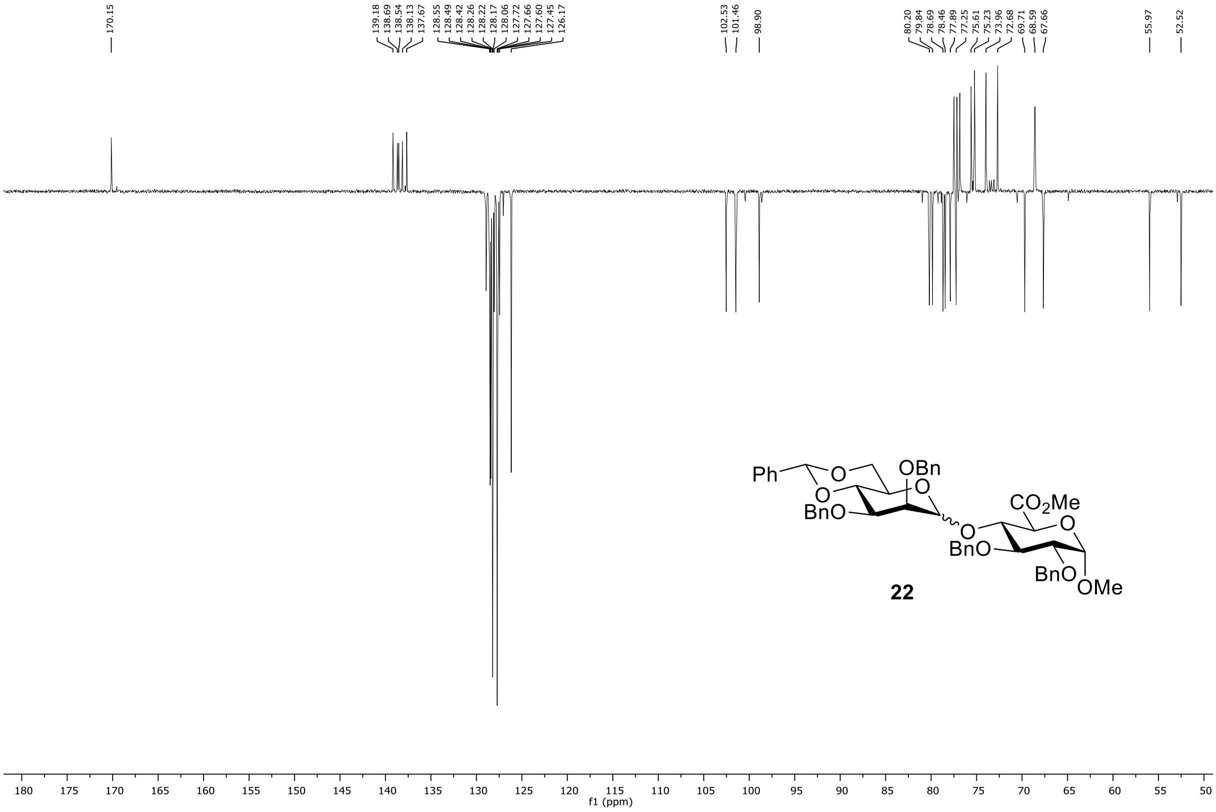

COSY NMR of compound **22**

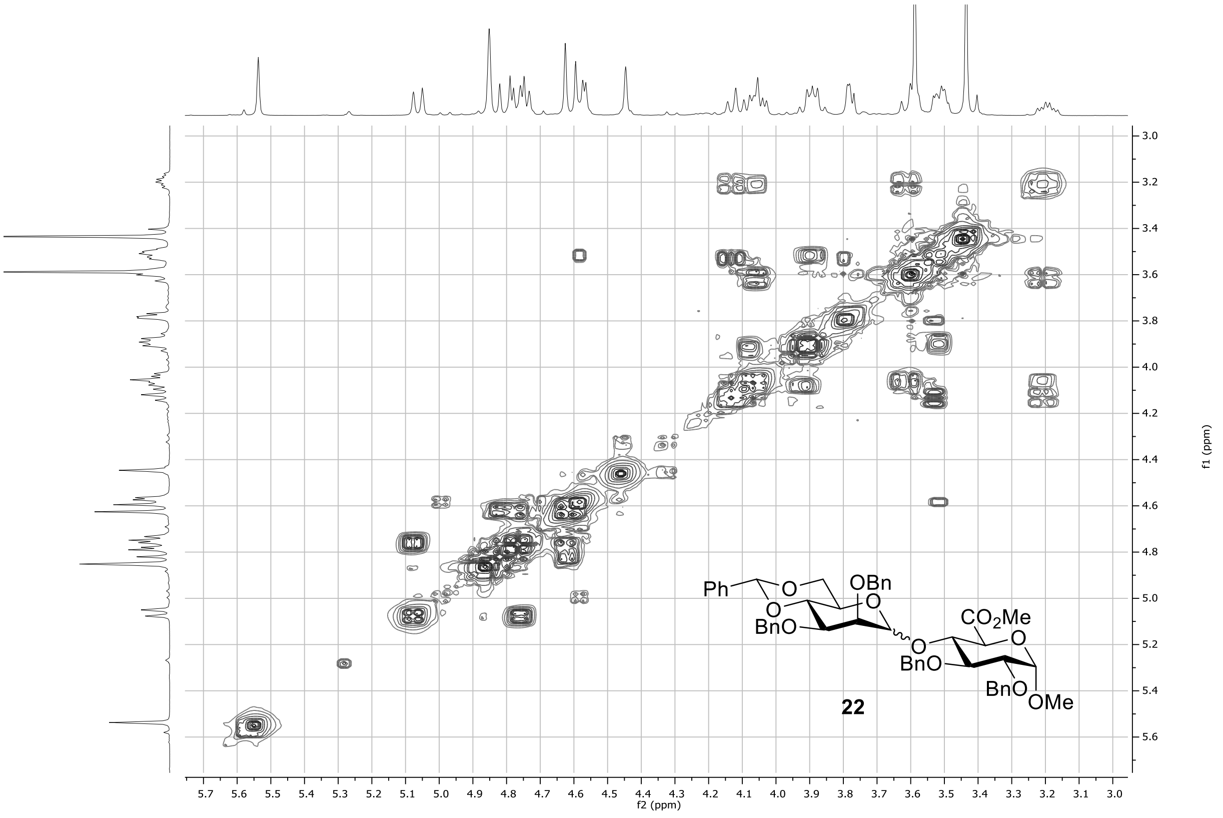

HSQC NMR of compound **22**

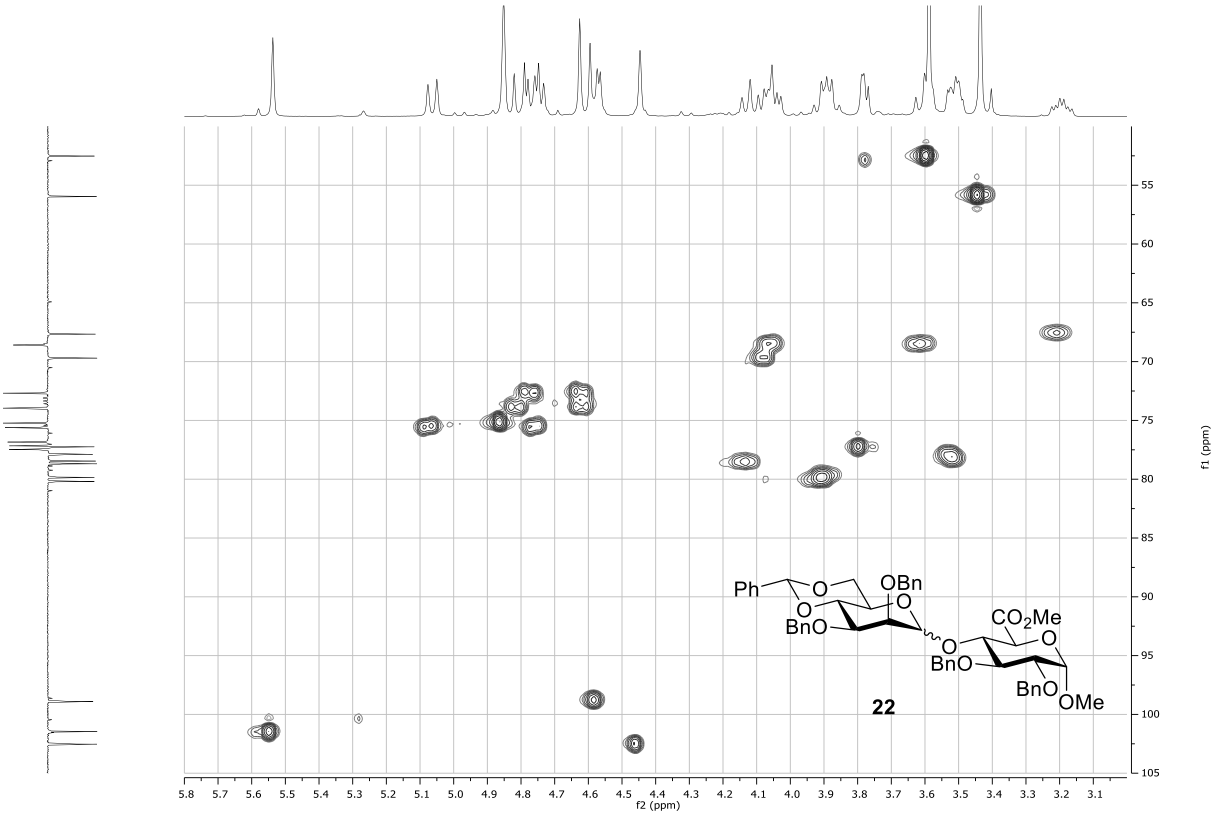

^{13}C GATED NMR, 101 MHz, $CDCl_3$ of compound **22**

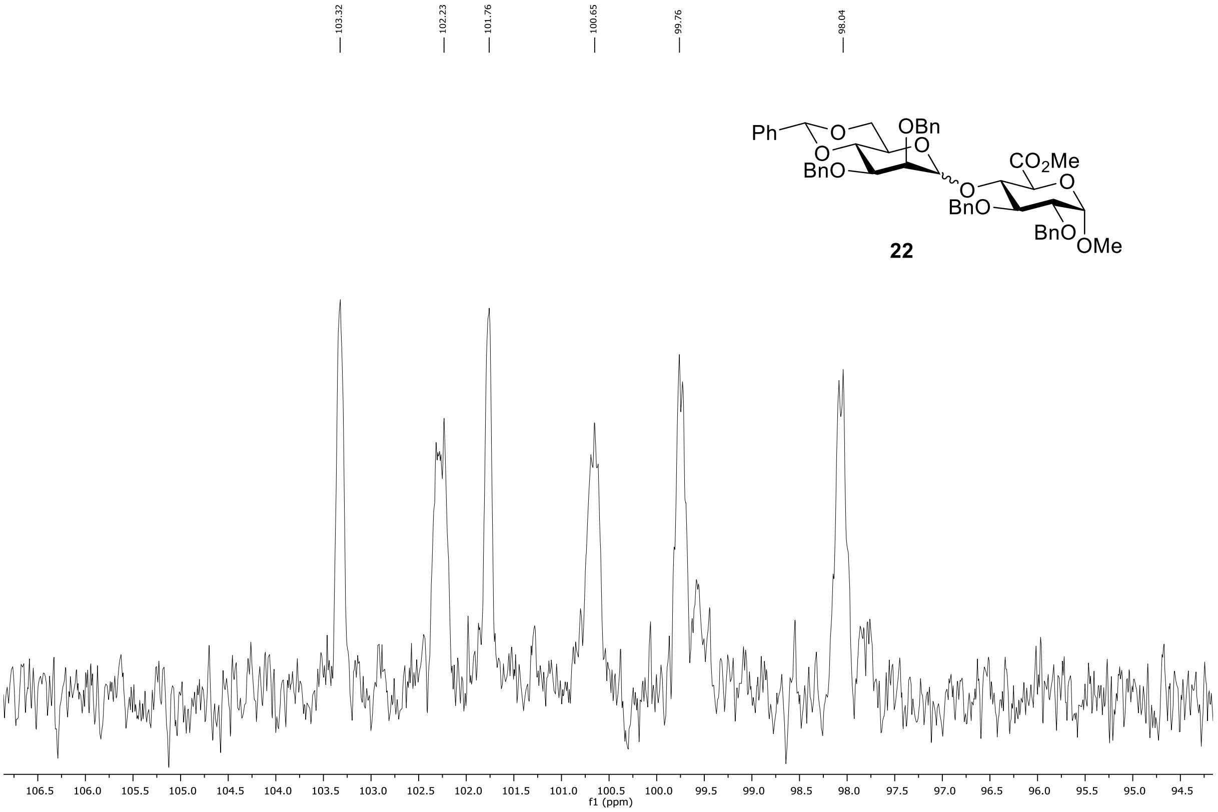

^{1}H NMR, 400 MHz, $CDCl_3$ of compound **23**

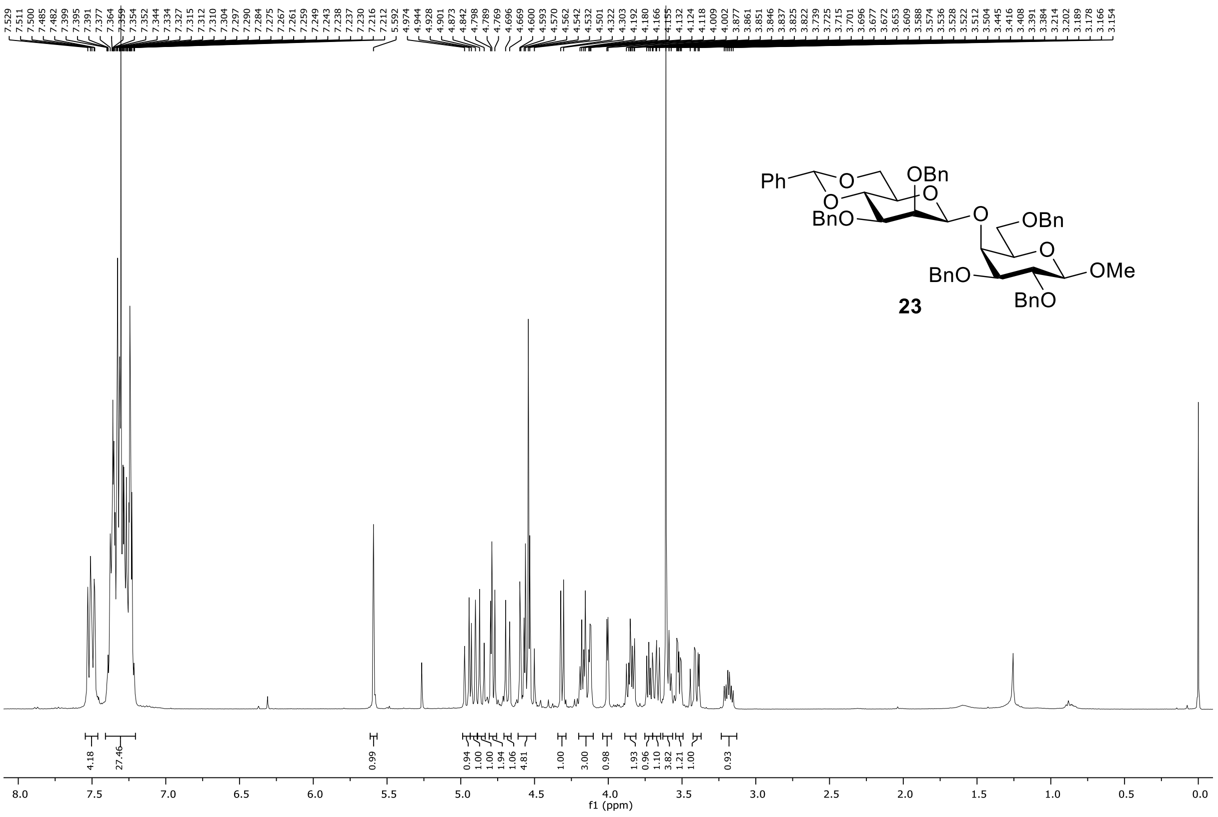

^{13}C-APT NMR, 101 MHz, $CDCl_3$ of compound **23**

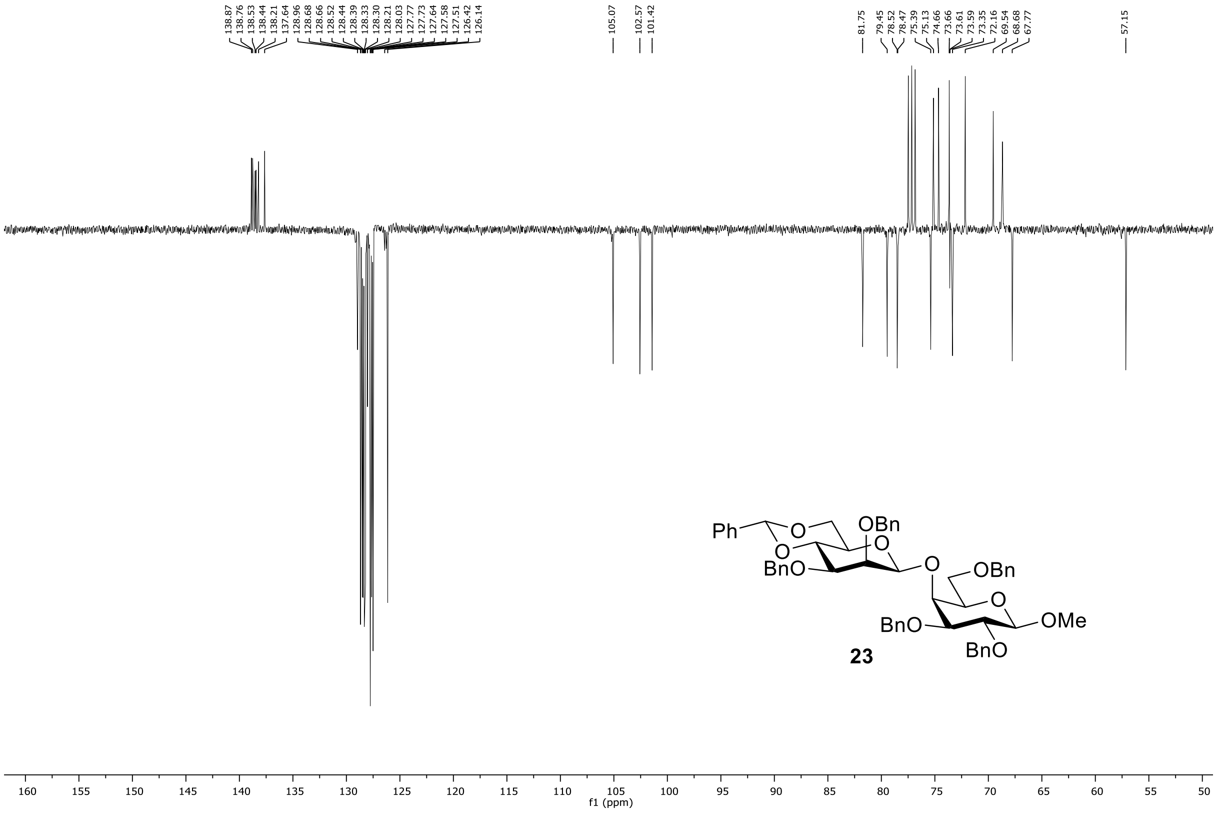

COSY NMR of compound **23**

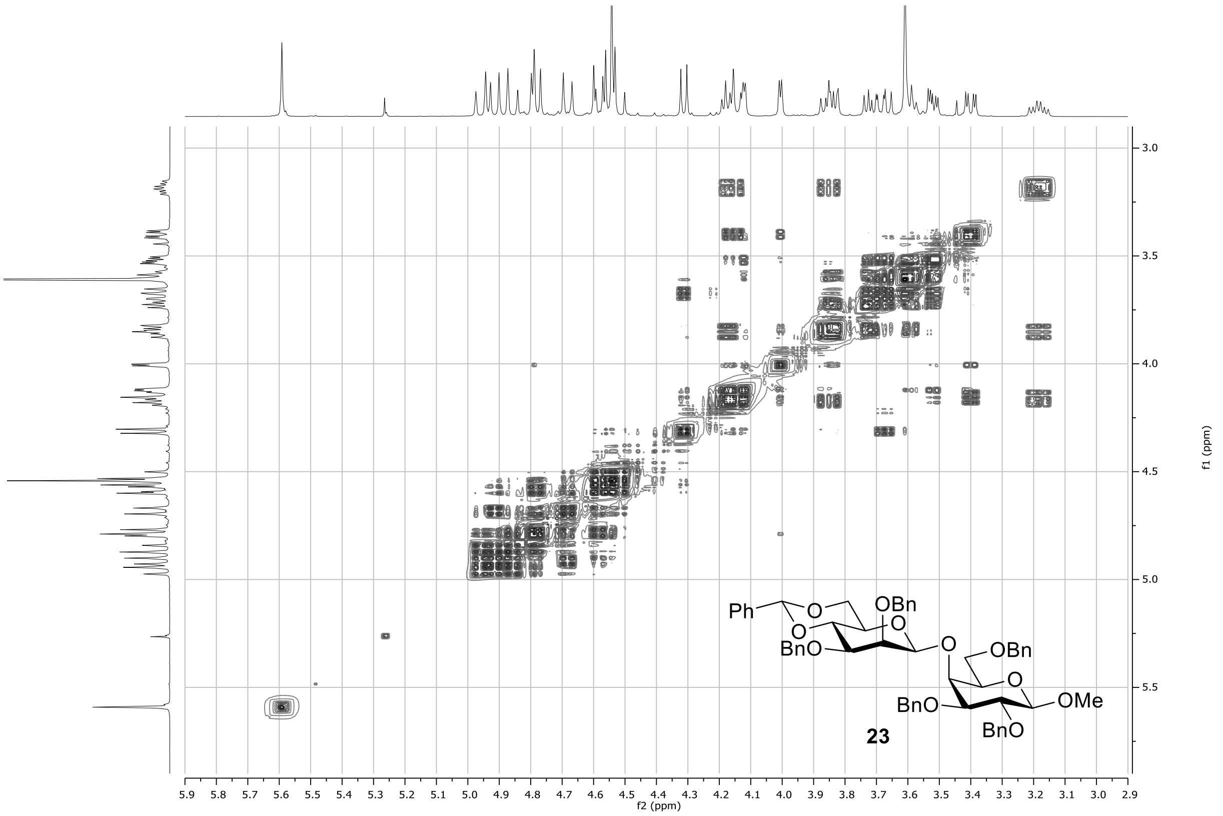

HSQC NMR of compound **23**

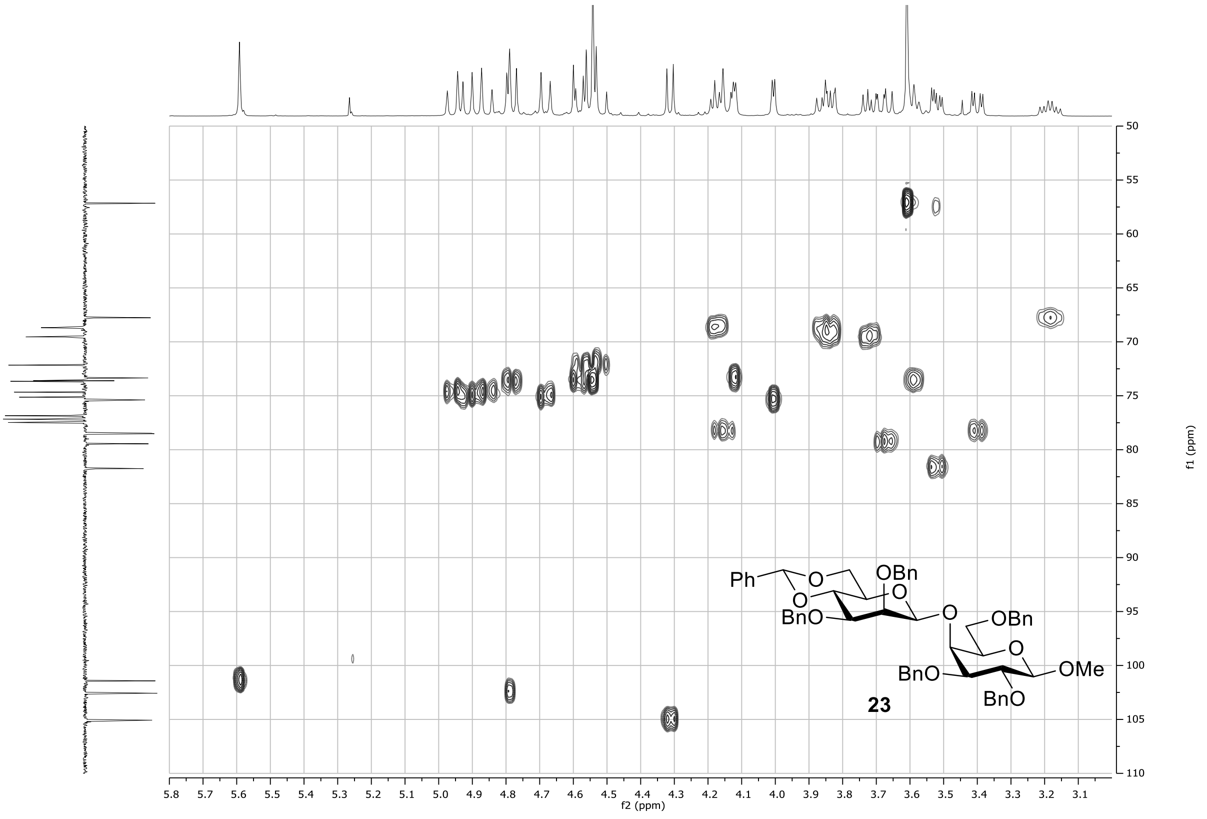

^{13}C GATED NMR of compound **23**

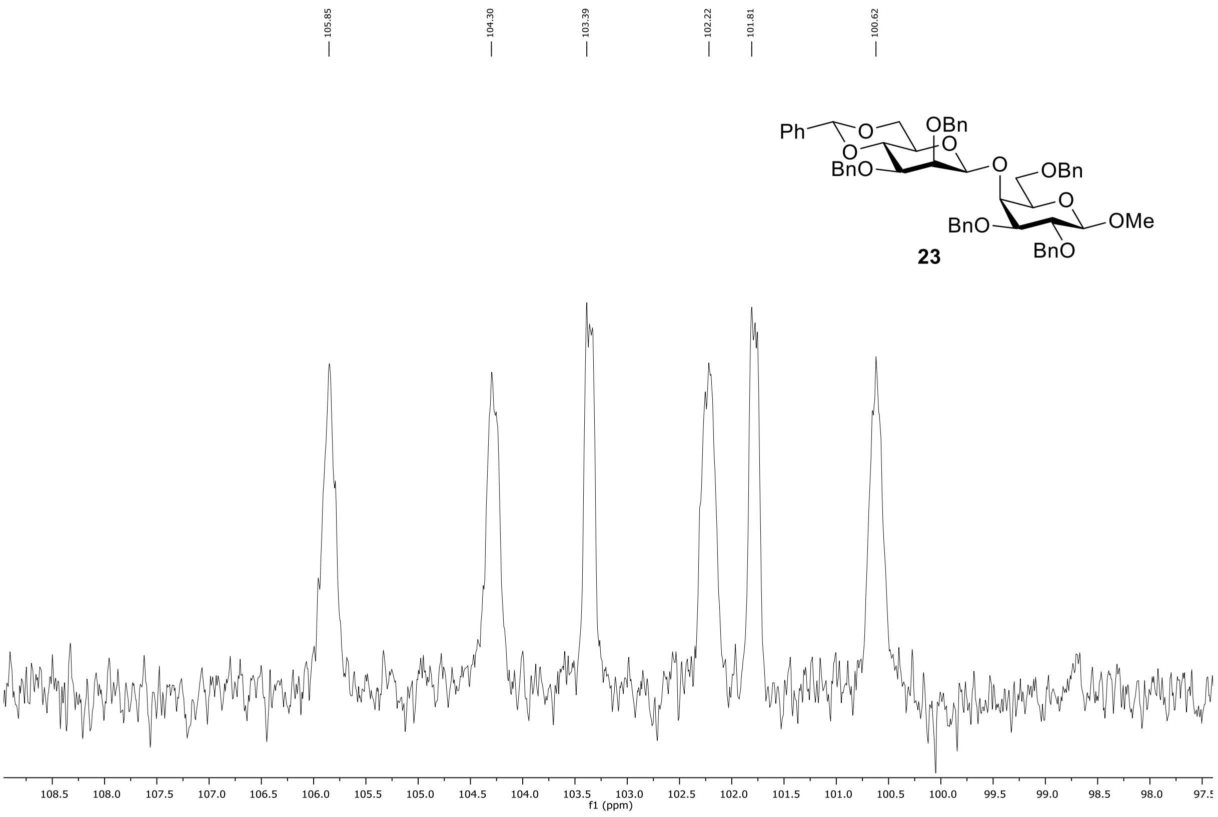

^{1}H NMR, 400 MHz, $CDCl_3$ of compound **2B**

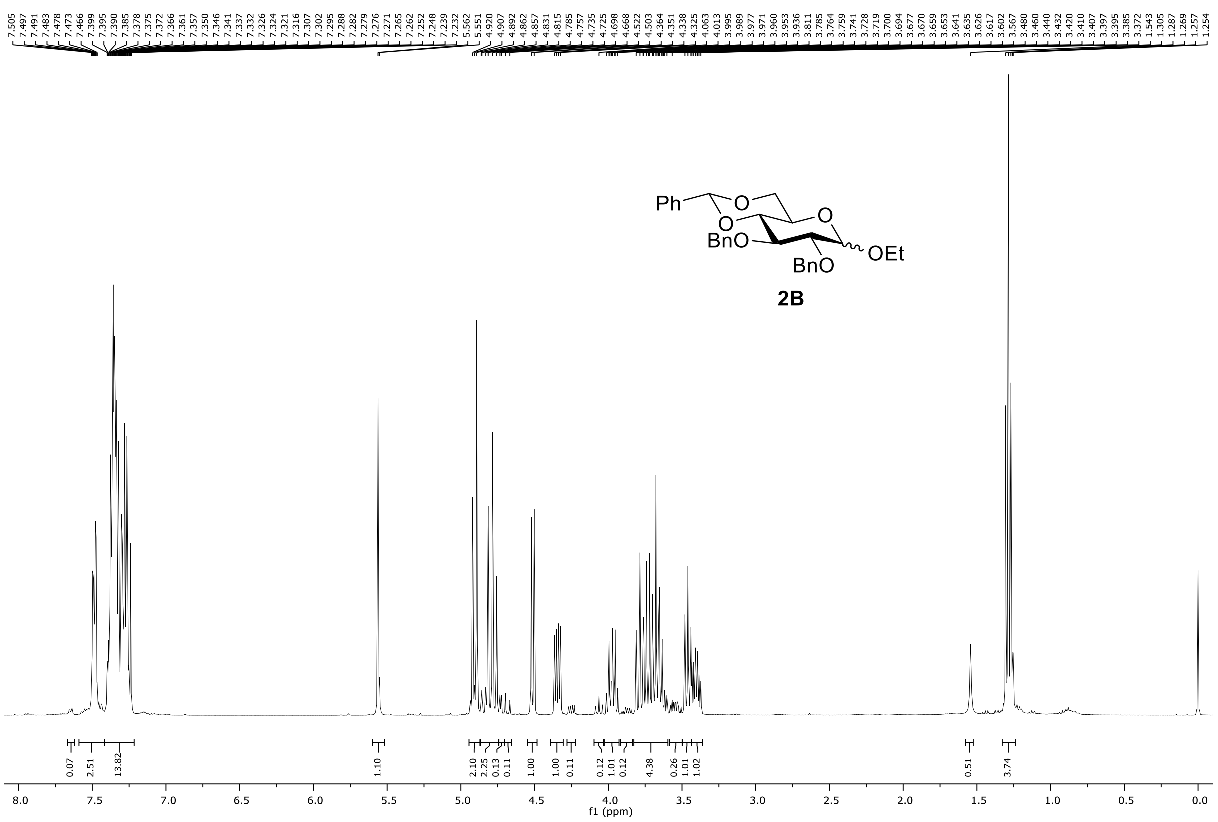

^{13}C-APT NMR, 101 MHz, $CDCl_3$ of compound **2B**

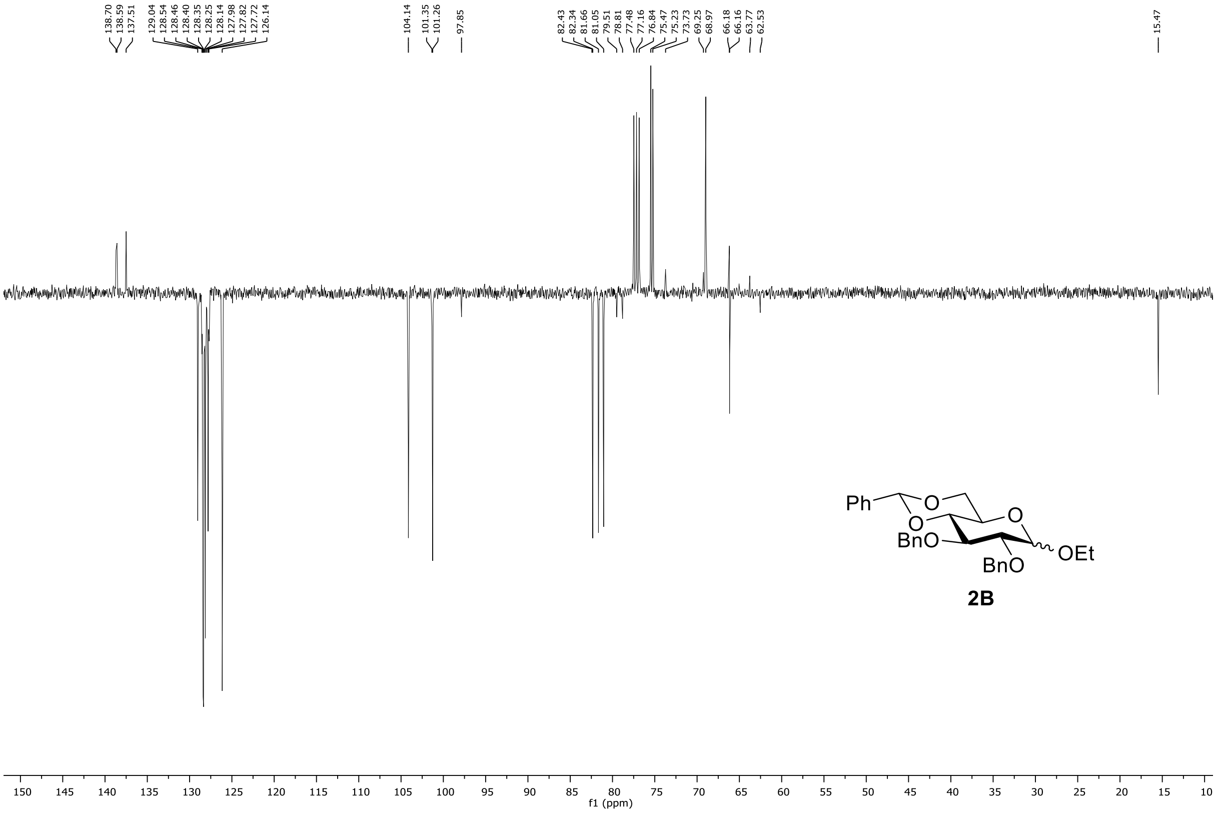

COSY NMR of compound **2B**

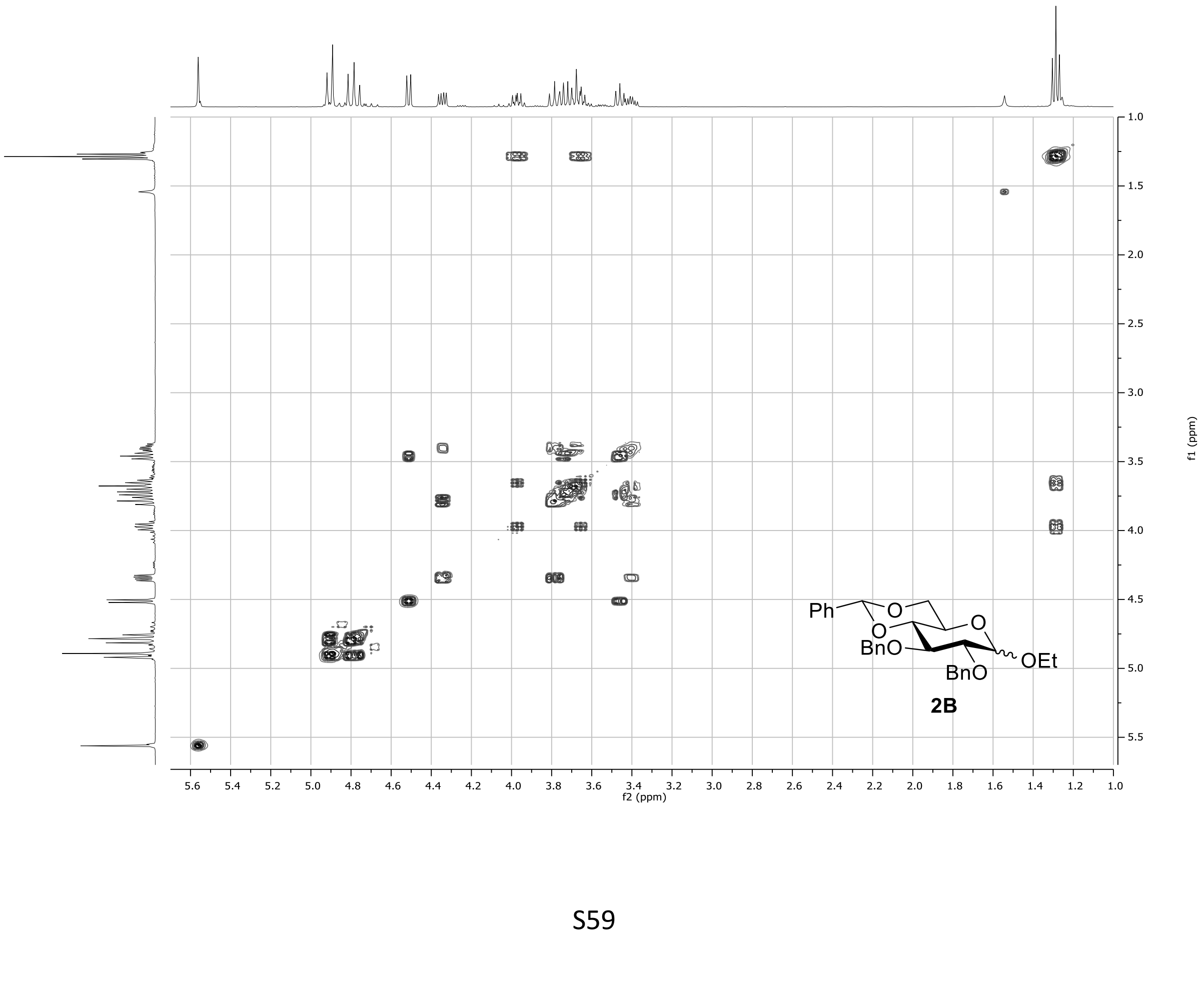

HSQC NMR of compound **2B**

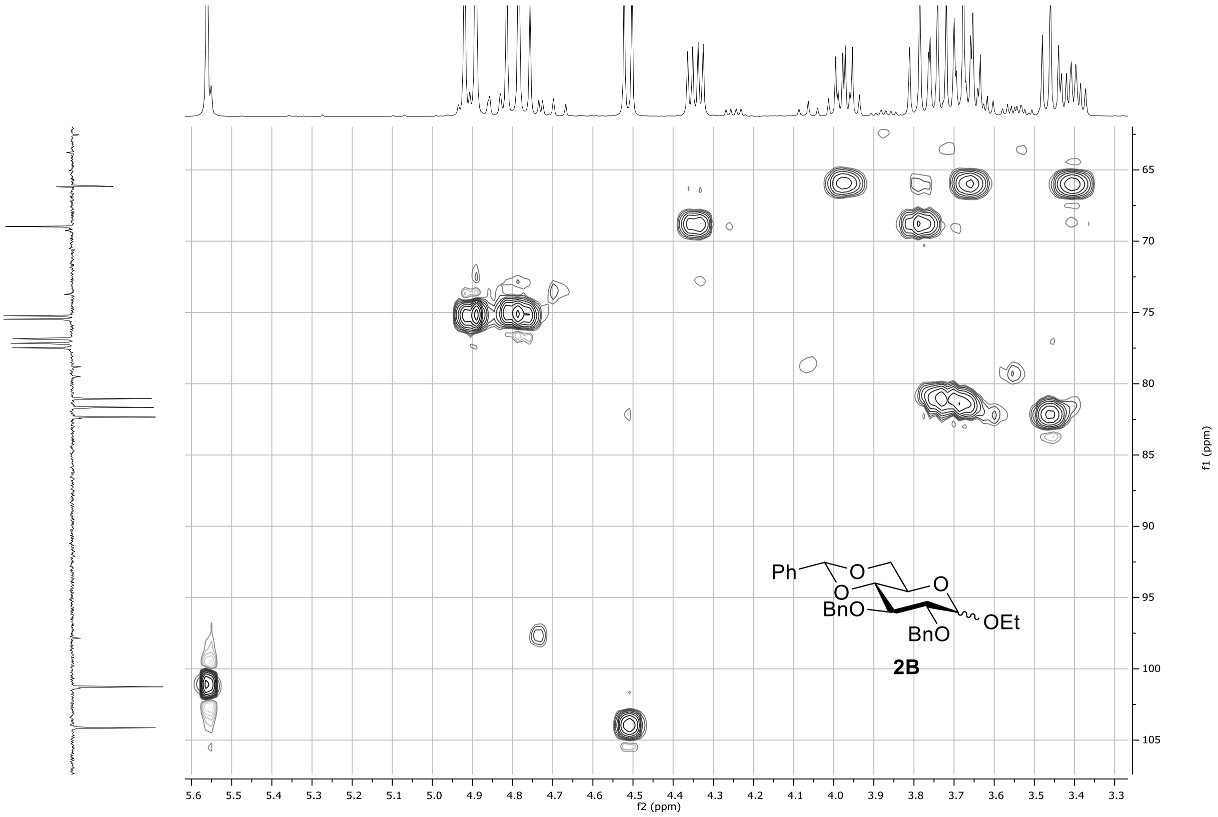

^{1}H NMR, 400 MHz, $CDCl_3$ of compound **2C**

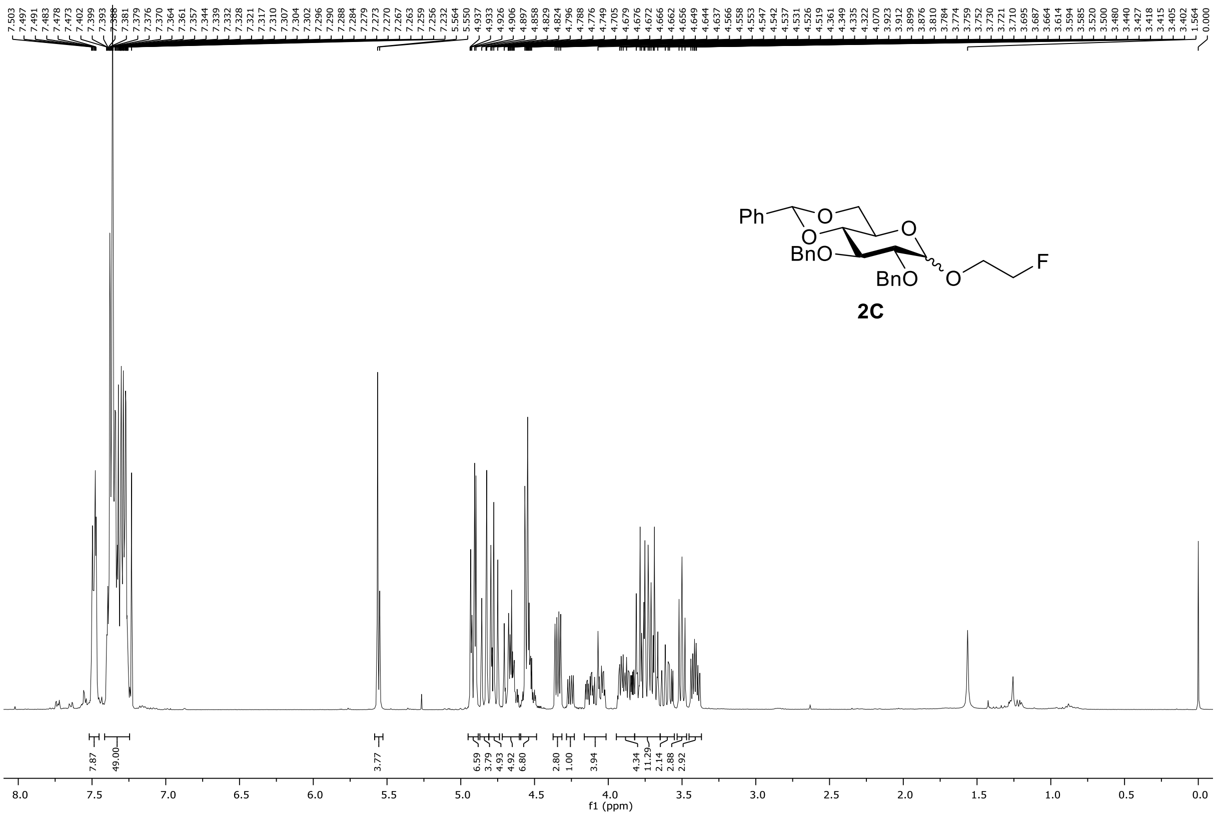

^{13}C-APT NMR, 101 MHz, $CDCl_3$ of compound **2C**

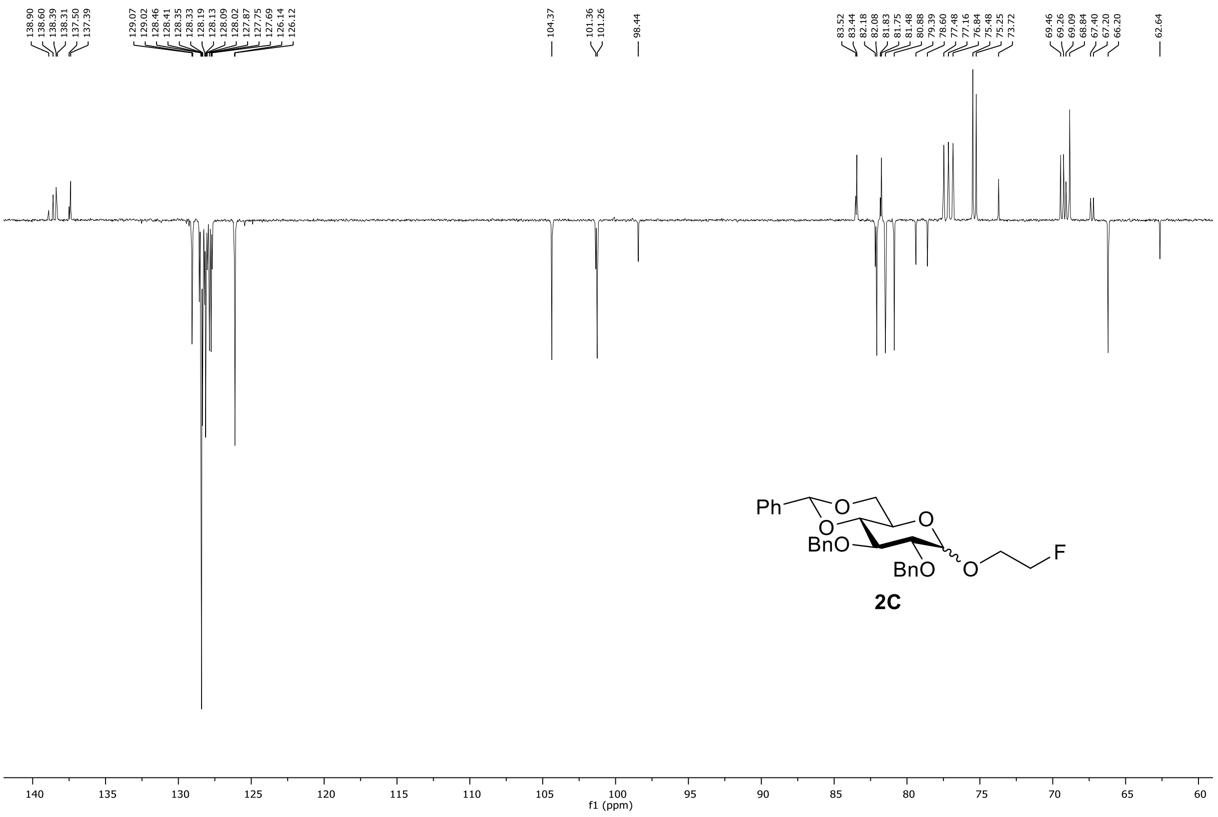

COSY NMR of compound **2C**

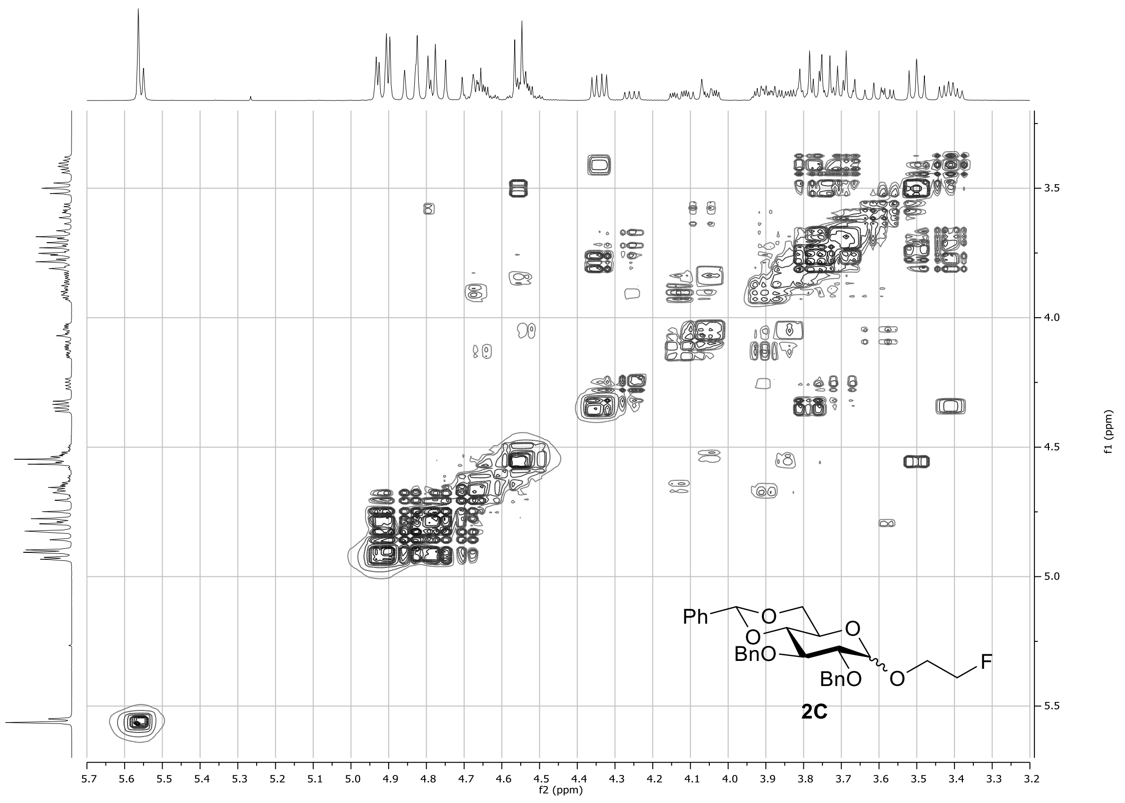

HSQC NMR of compound **2C**

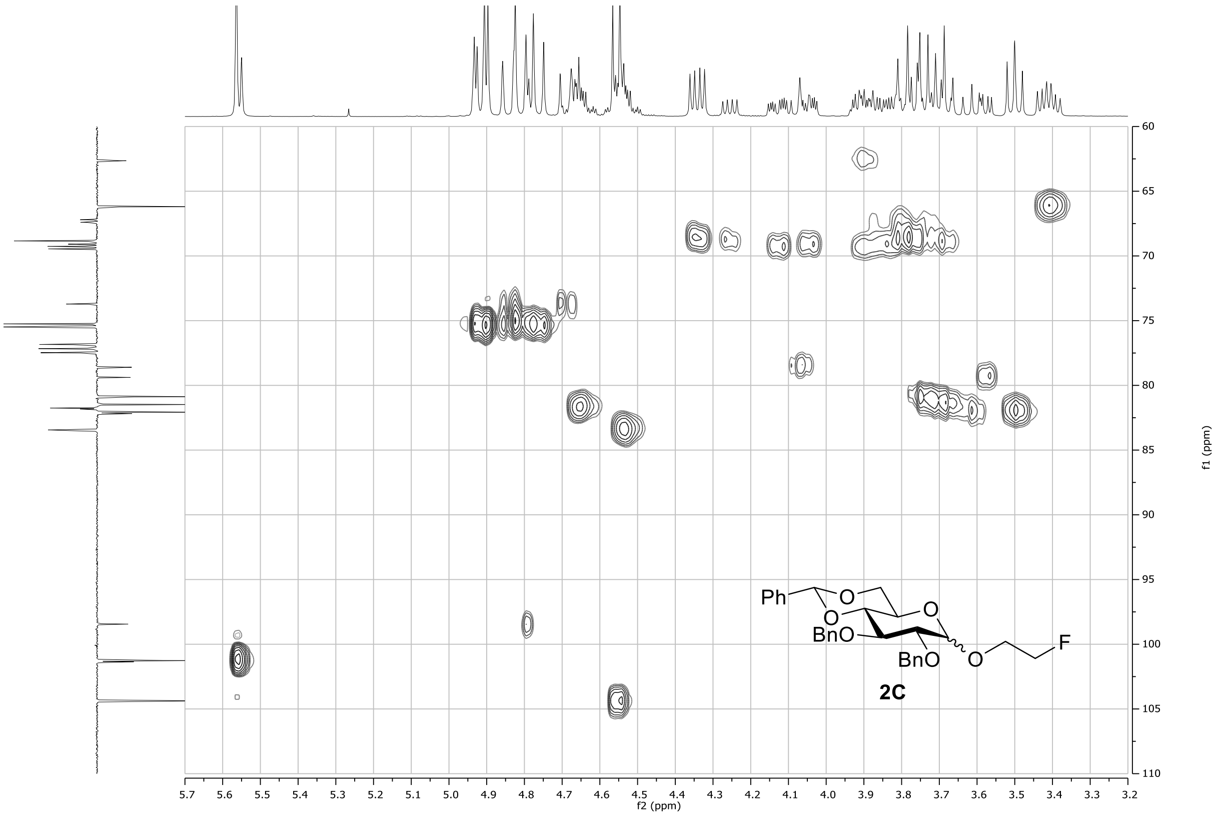

^{1}H NMR, 400 MHz, $CDCl_3$ of compound **2D**

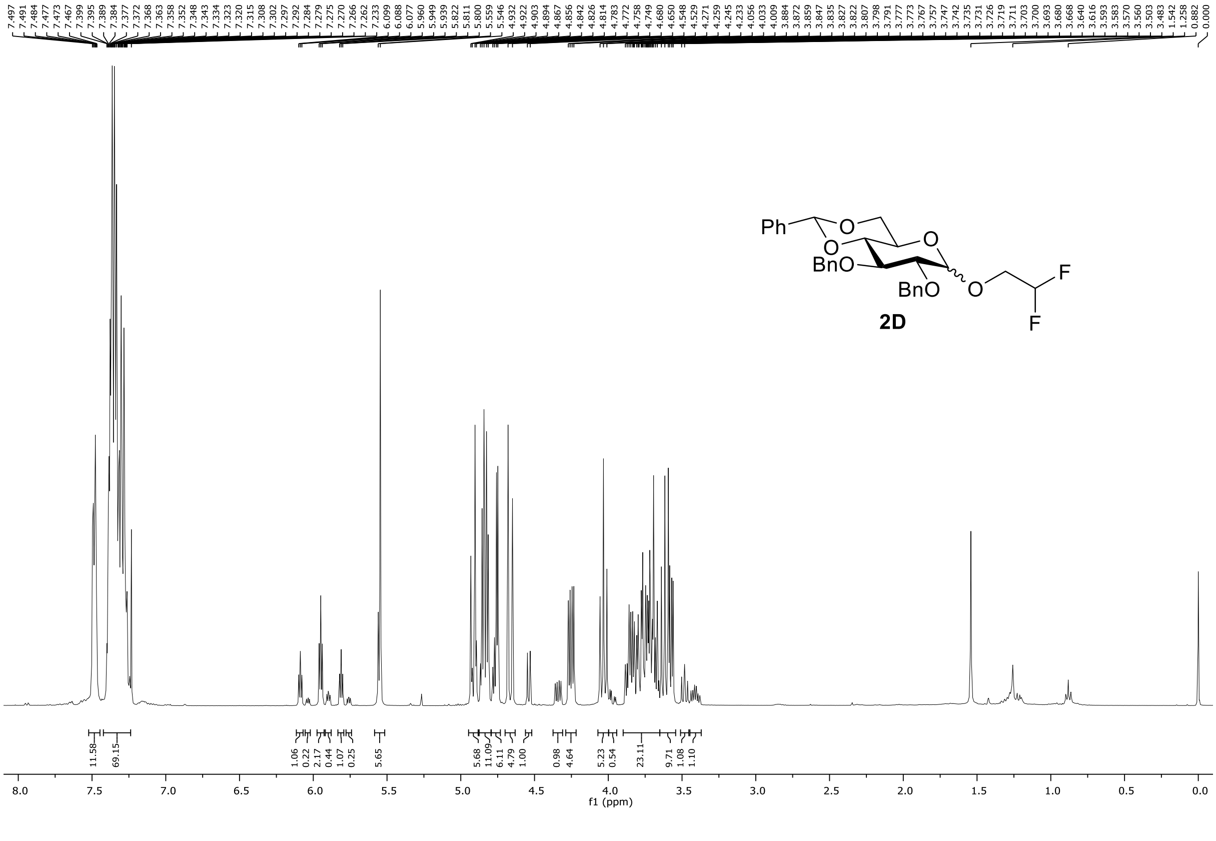

^{13}C-APT NMR, 101 MHz, $CDCl_3$ of compound **2D**

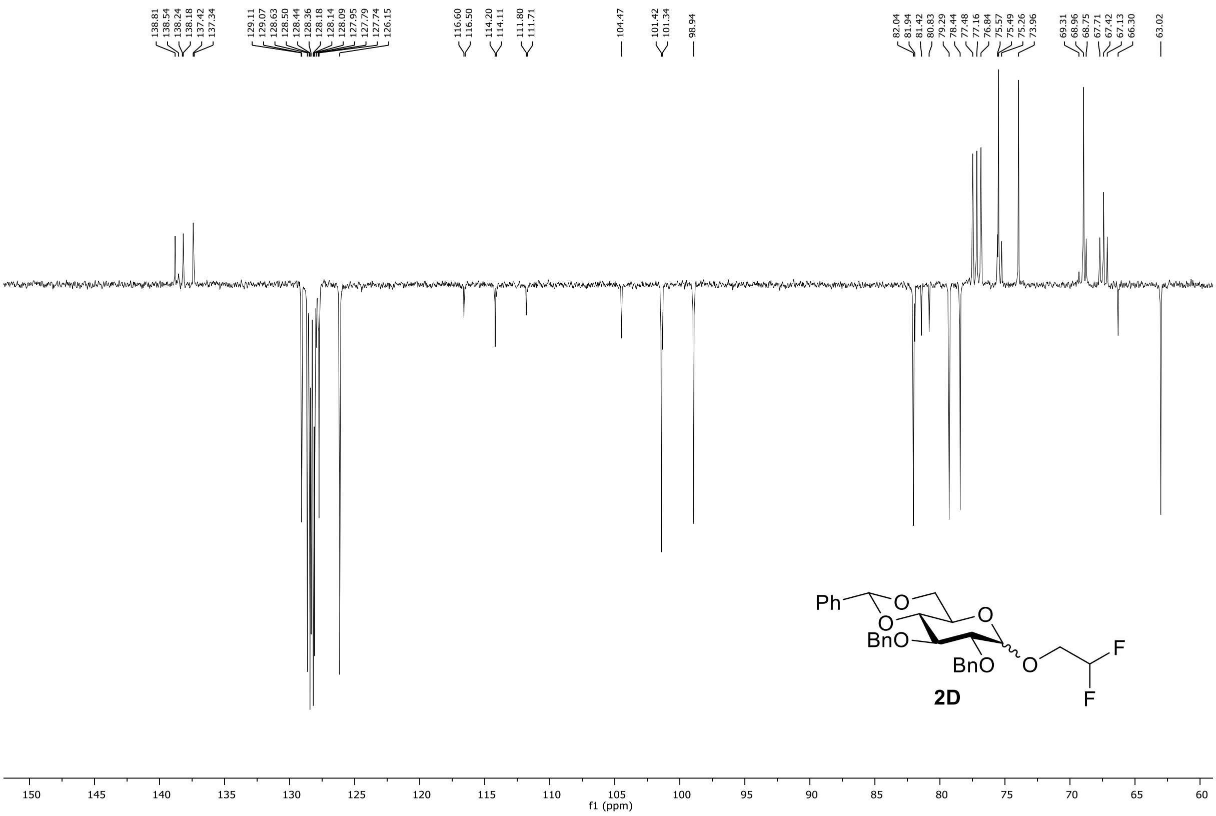

COSY NMR of compound **2D**

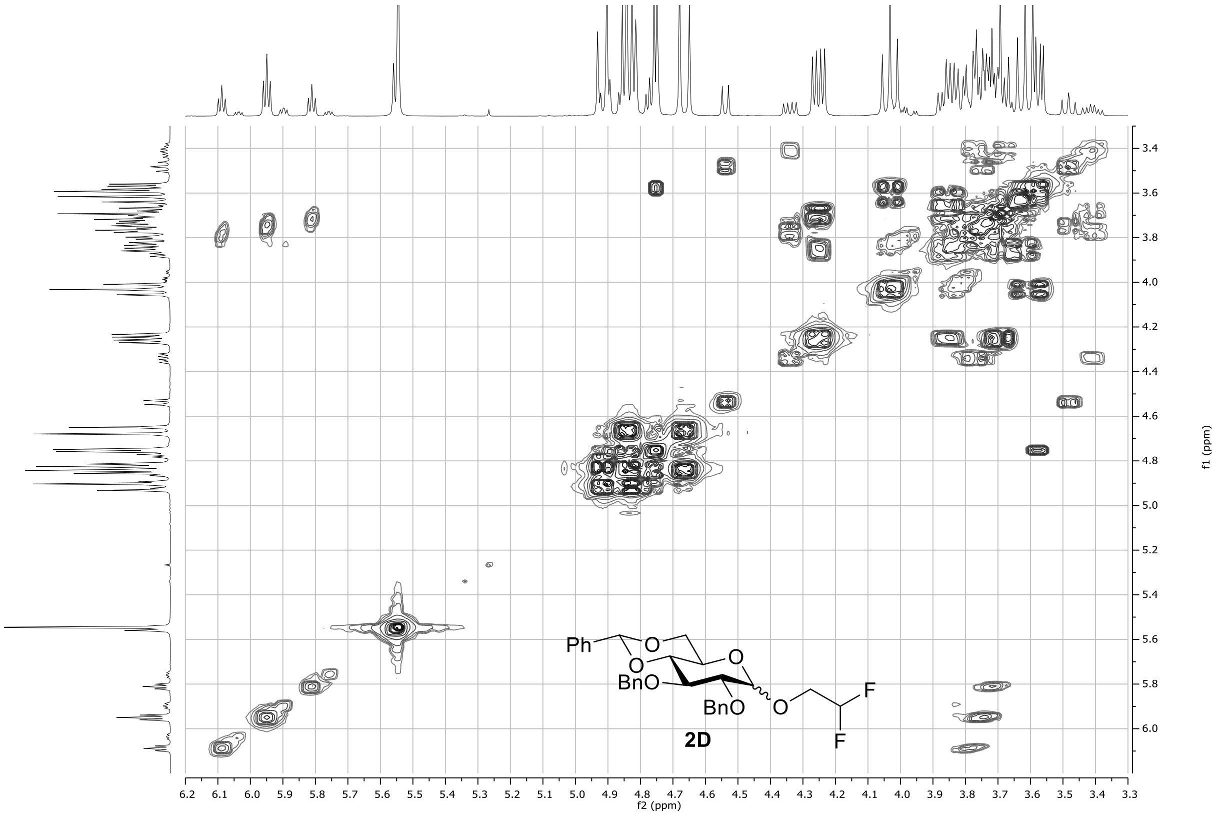

HSQC NMR of compound **2D**

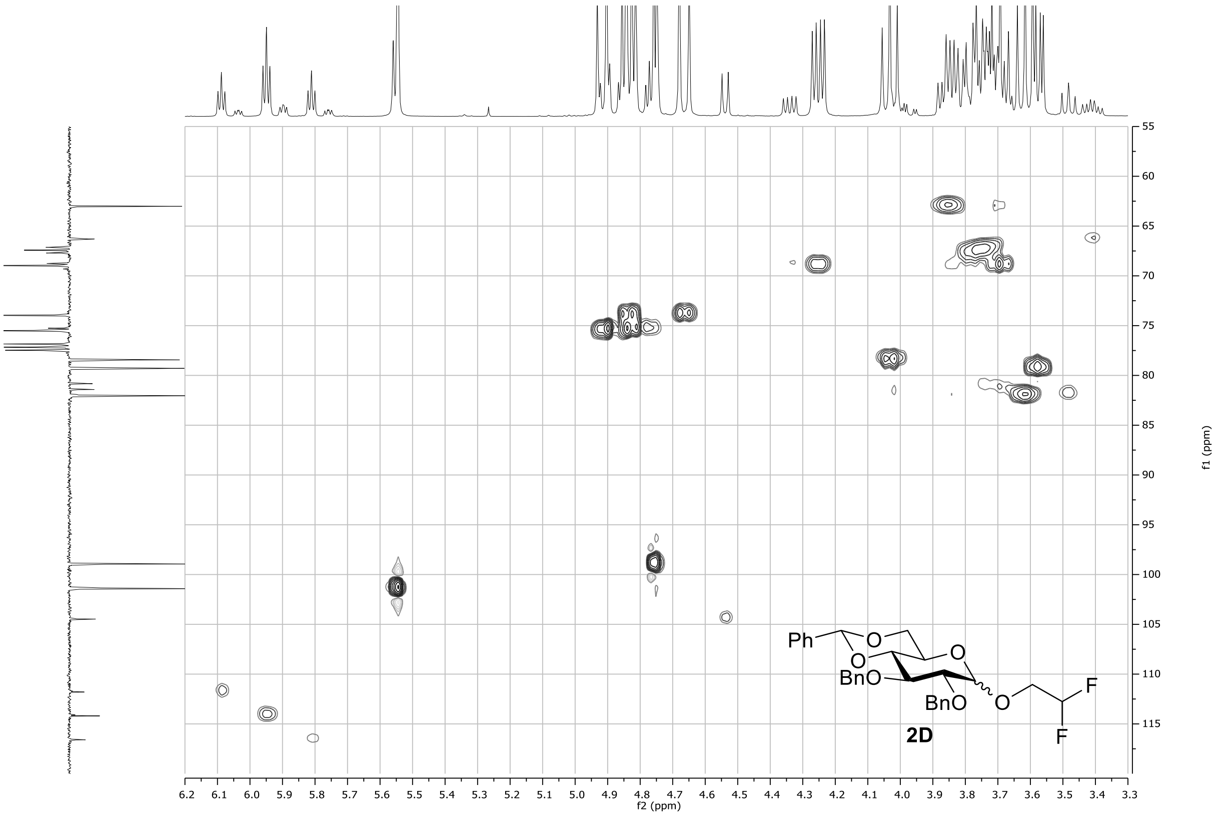

^{1}H NMR, 400 MHz, $CDCl_3$ of compound **2E**

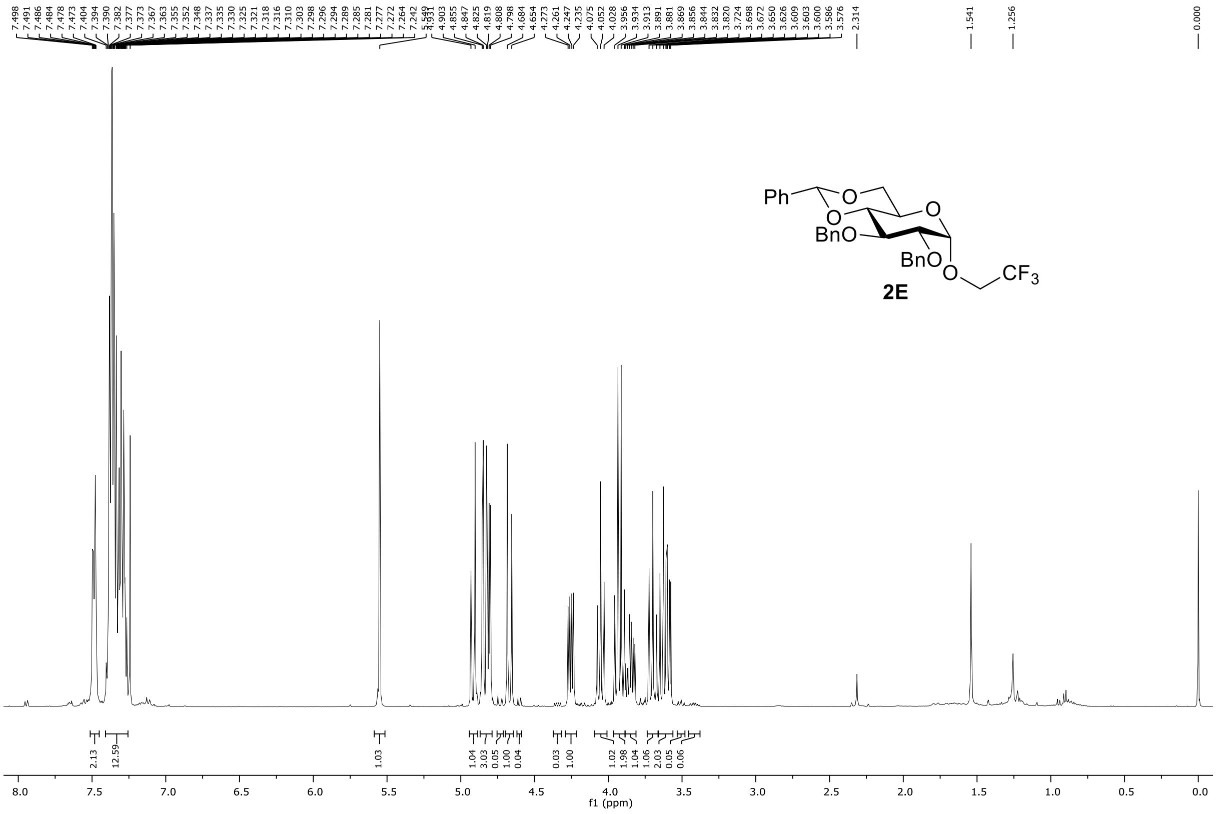

^{13}C-APT NMR, 101 MHz, $CDCl_3$ of compound **2E**

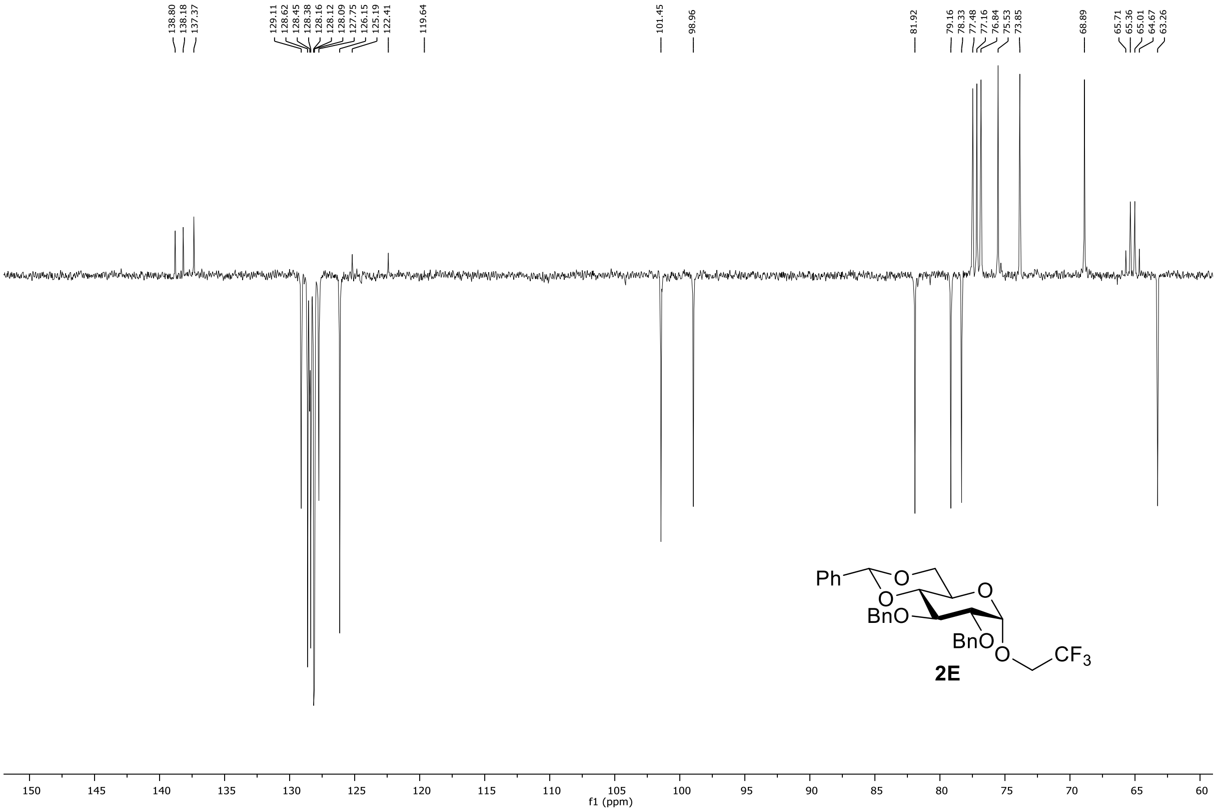

COSY NMR of compound **2E**

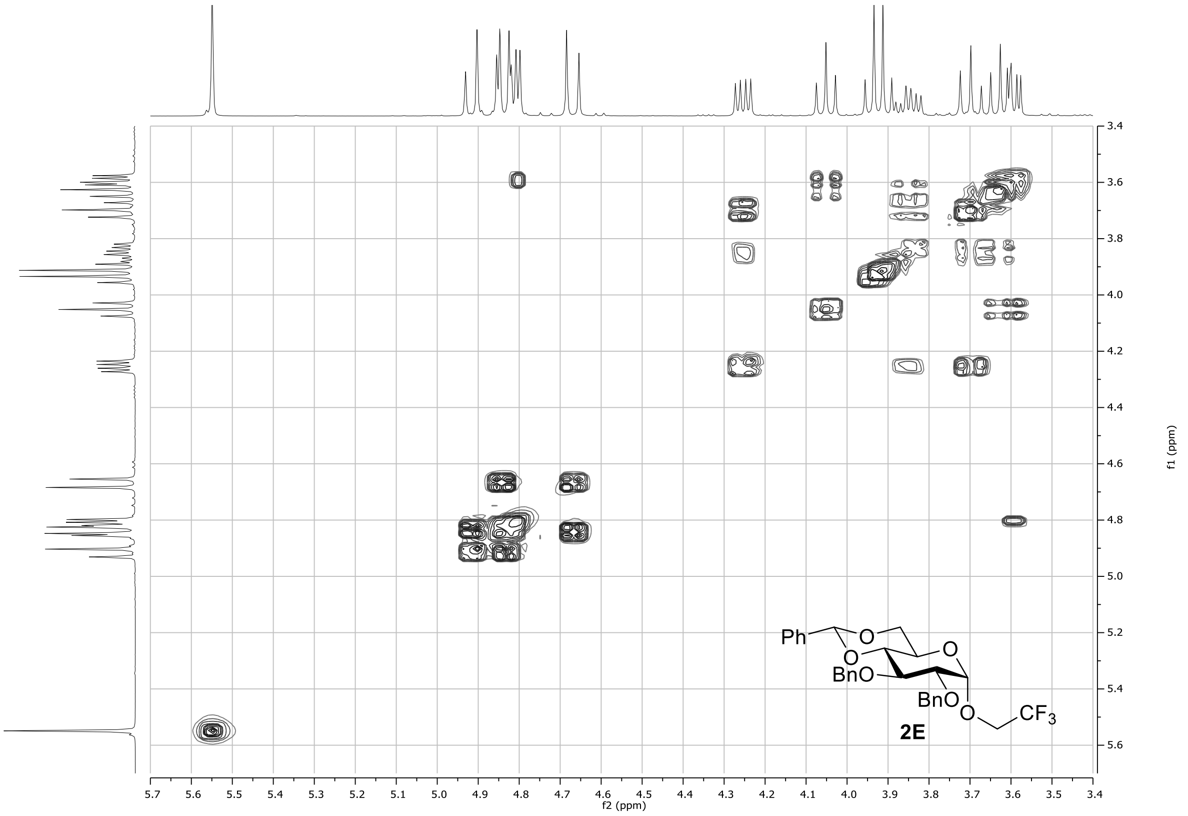

HSQC NMR of compound **2E**

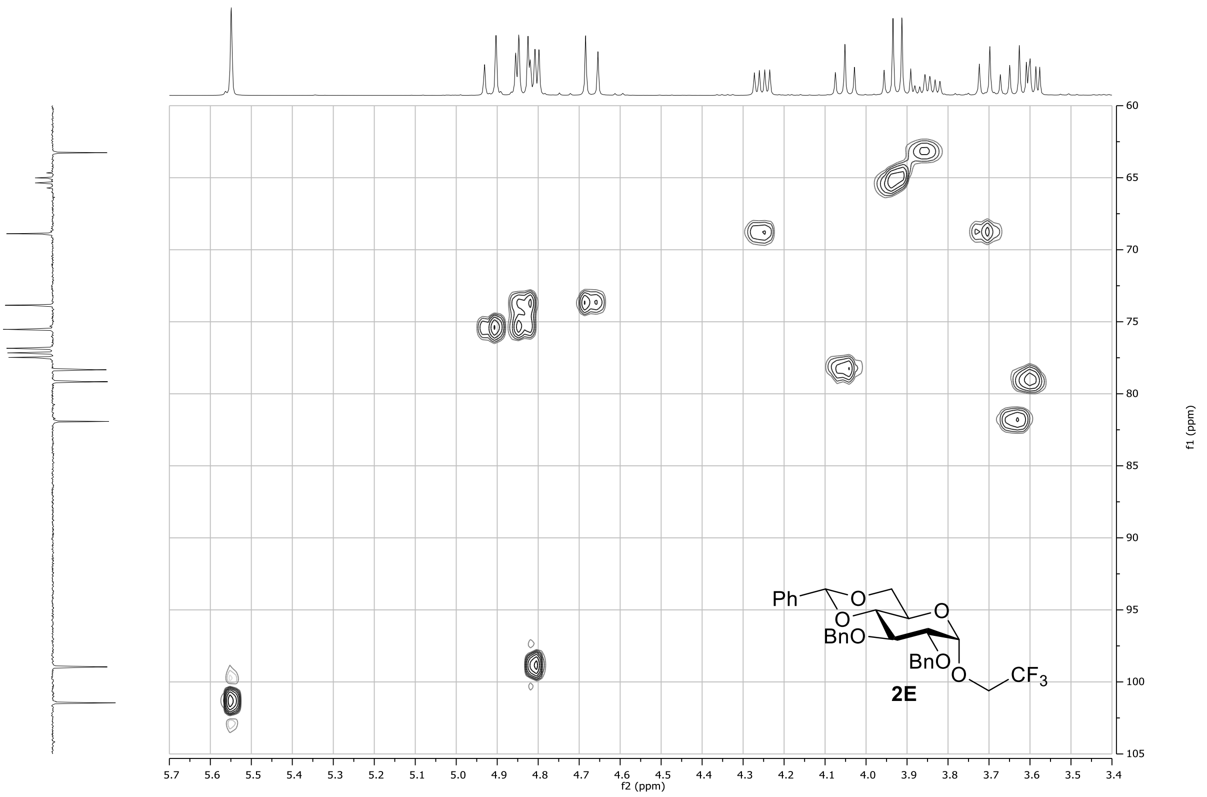

^{1}H NMR, 400 MHz, $CDCl_3$ of compound **2F**

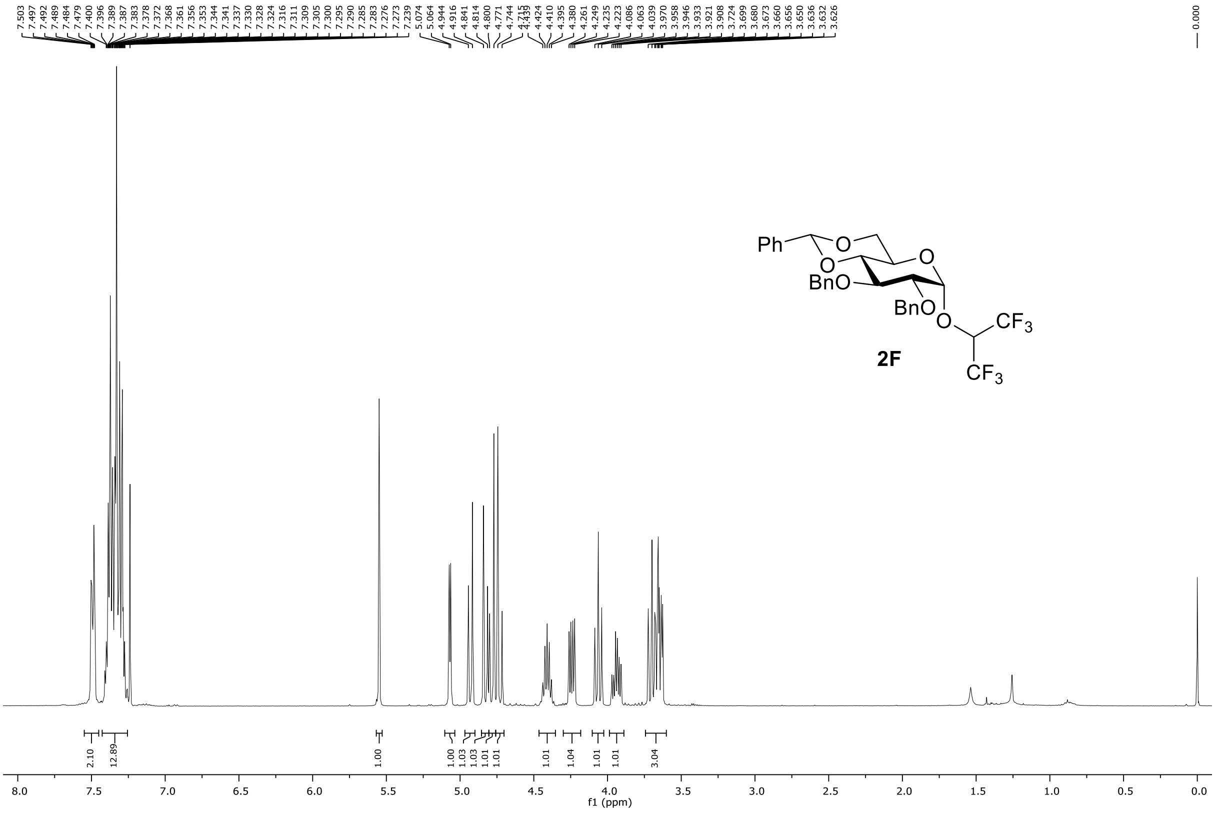

^{13}C-APT NMR, 101 MHz, $CDCl_3$ of compound **2F**

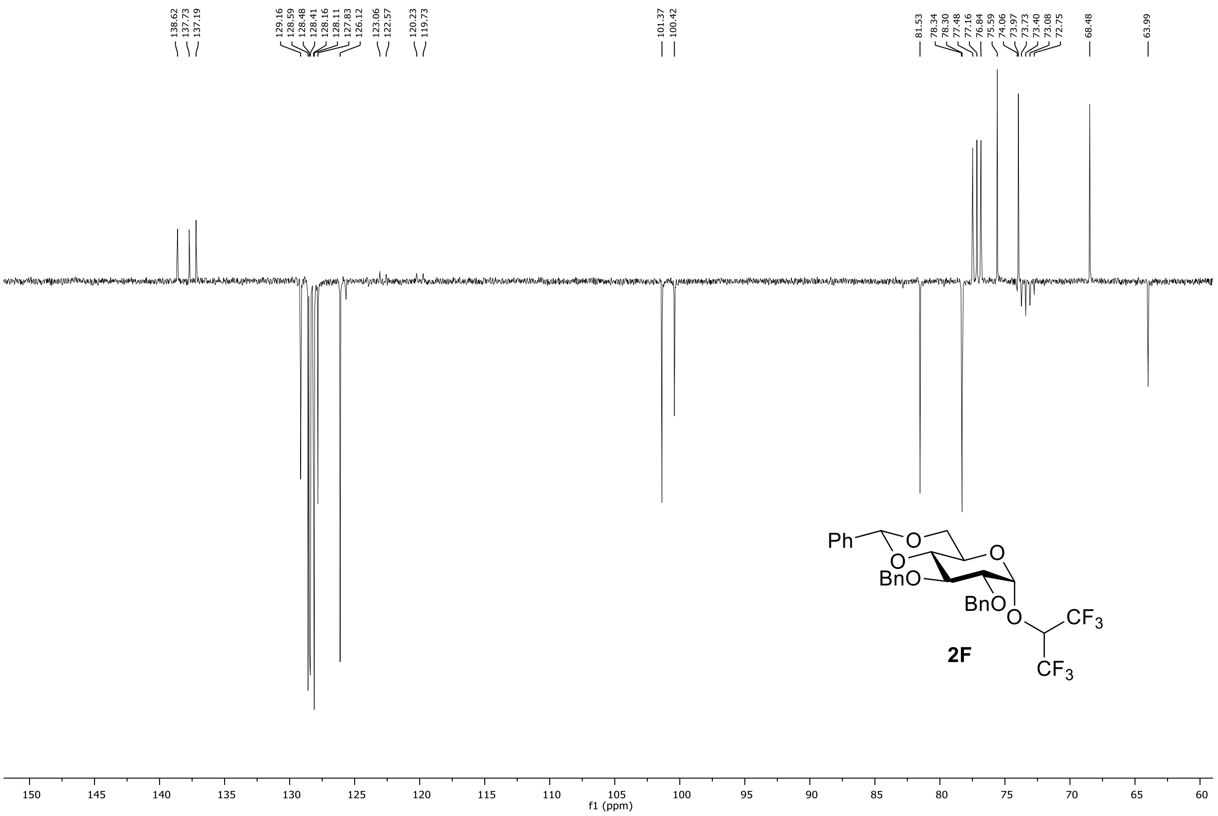

COSY NMR of compound **2F**

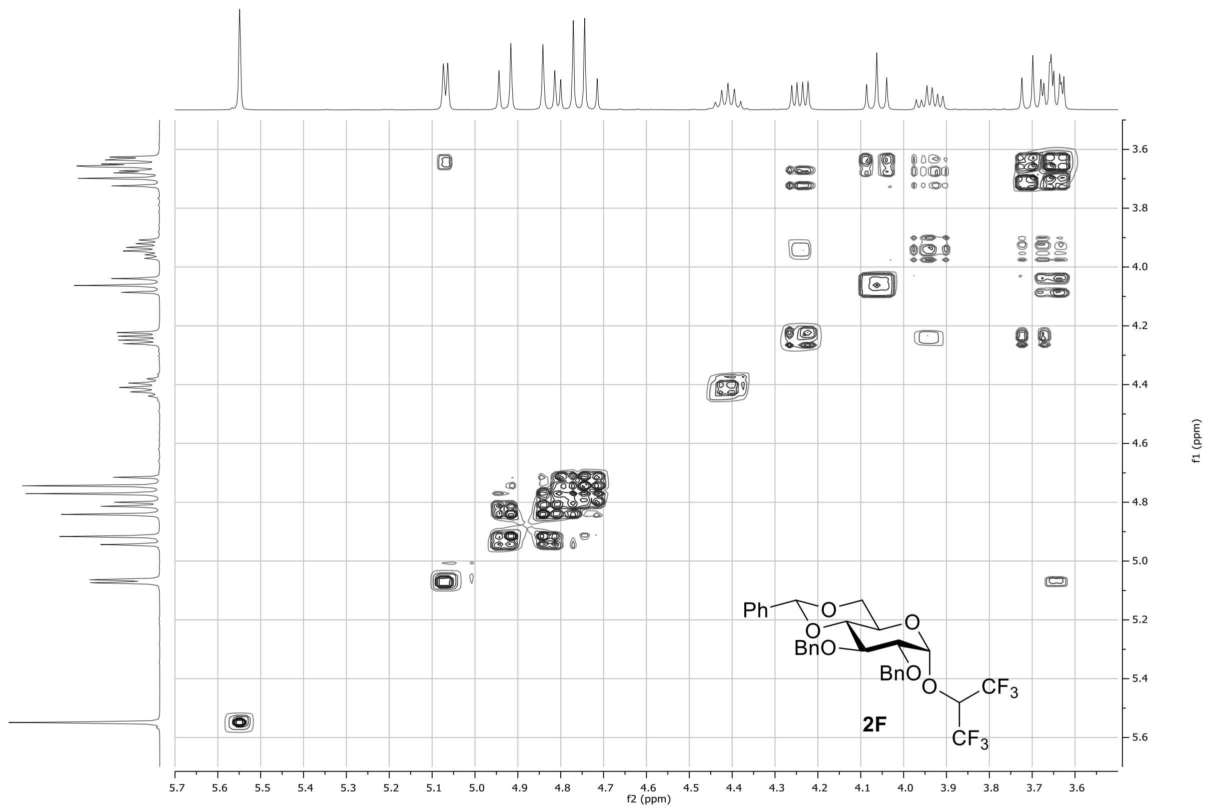

HSQC NMR of compound **2F**

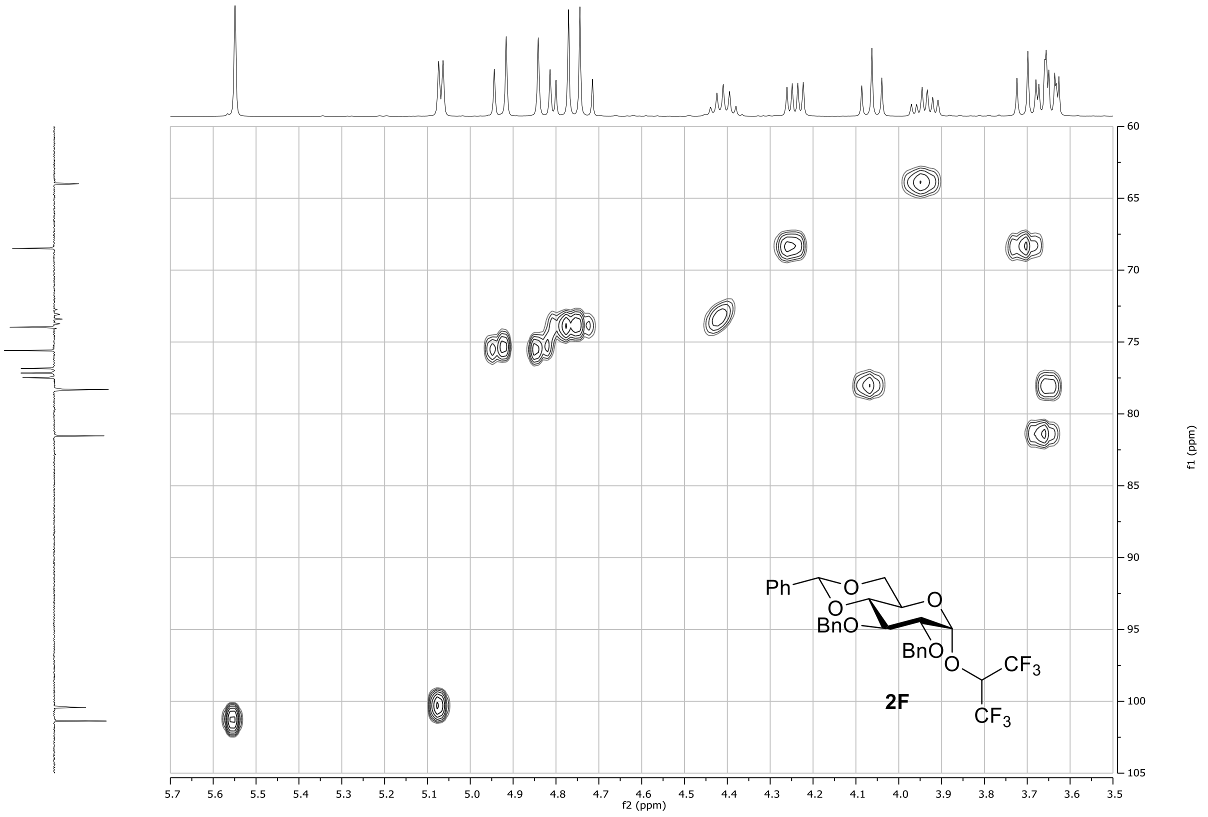

HMBC NMR of compound **2F**

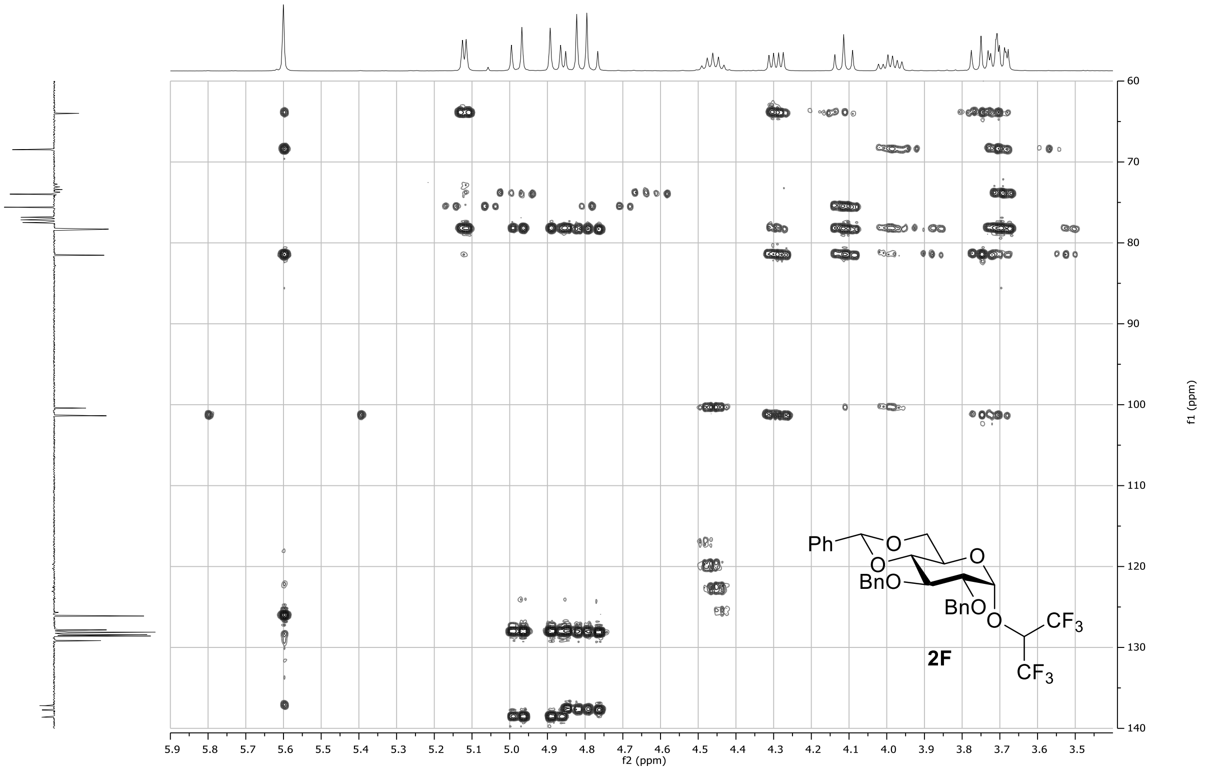

^{1}H NMR, 400 MHz, $CDCl_3$ of compound **2G**

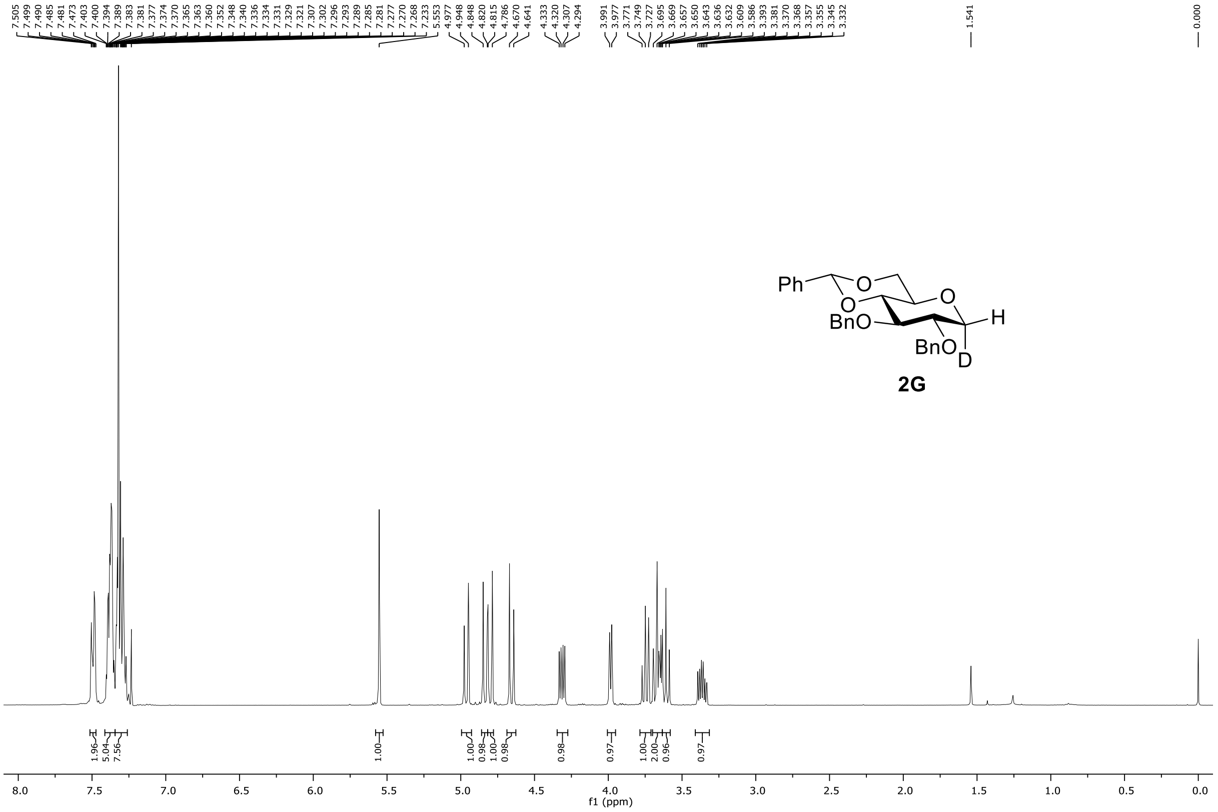

^{13}C-APT NMR, 101 MHz, $CDCl_3$ of compound **2G**

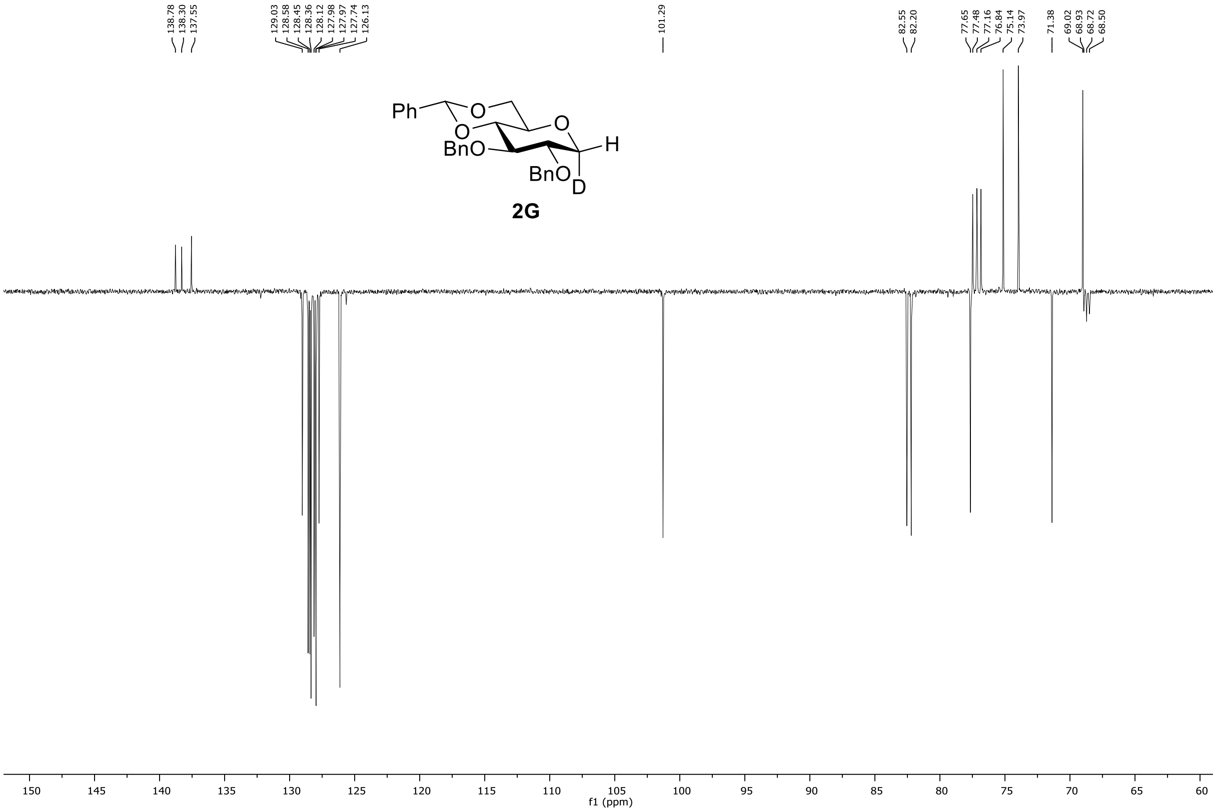

COSY NMR, $CDCl_3$ of compound **2G**

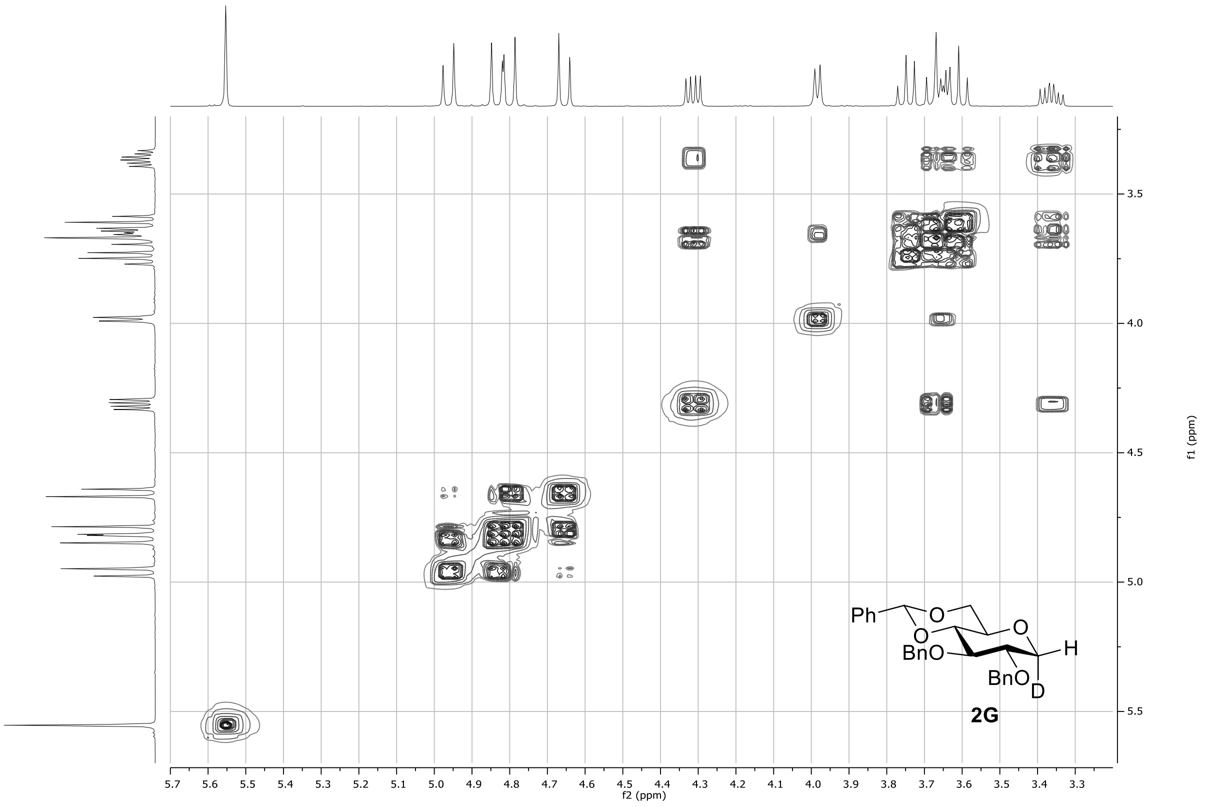

HSQC NMR, $CDCl_3$ of compound **2G**

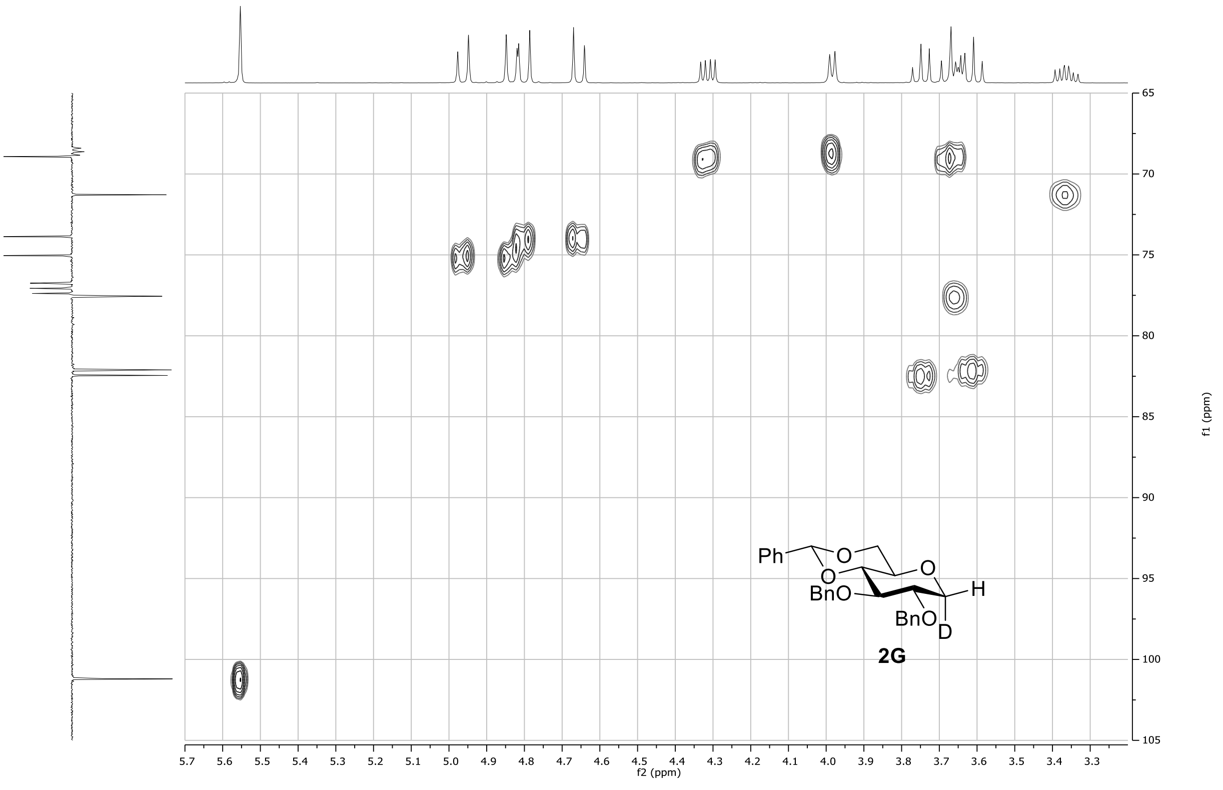

^{2}H NMR, 61 MHz, $CHCl_3$ of compound **2G**

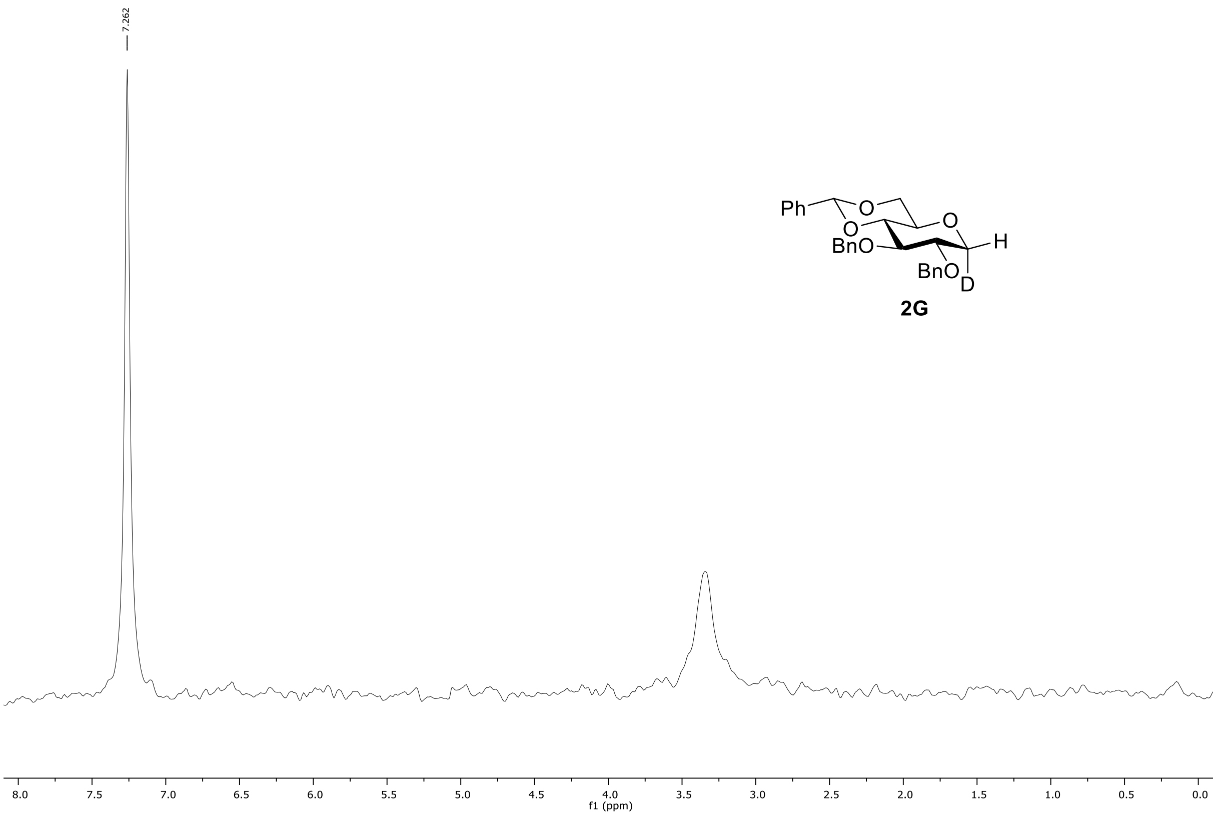

^{1}H NMR, 400 MHz, $CDCl_3$ of compound **27**

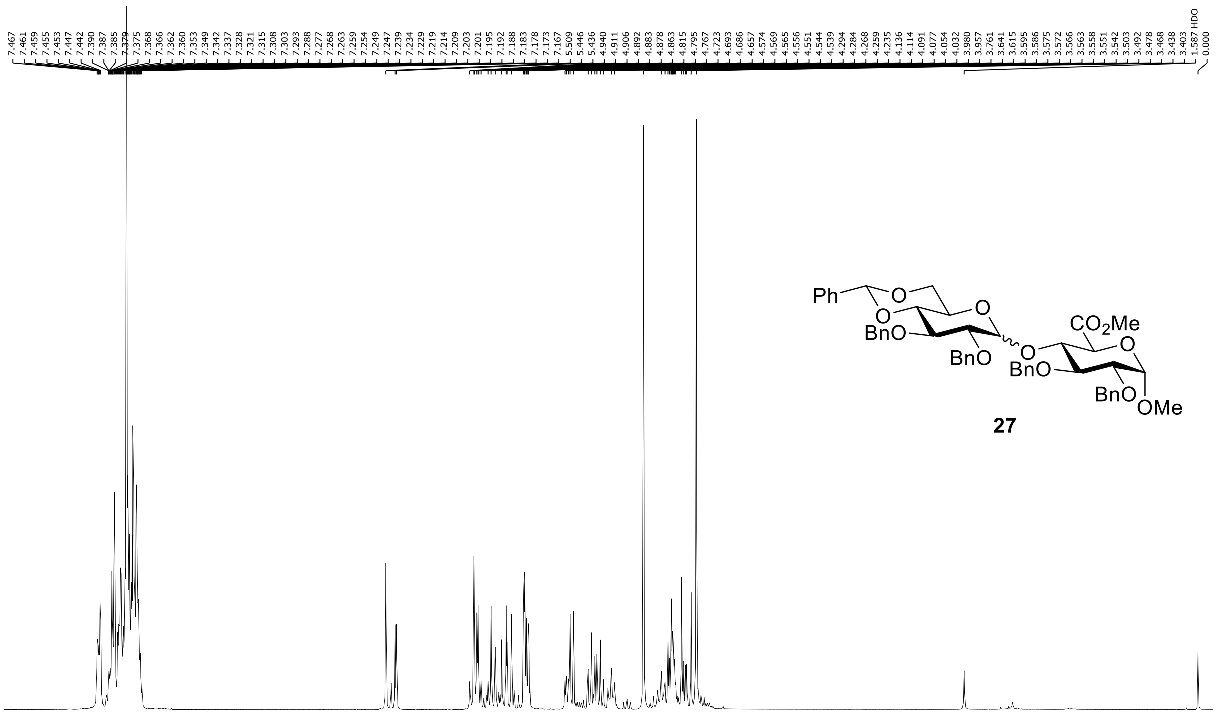

^{13}C-APT NMR, 101 MHz, $CDCl_3$ of compound **27**

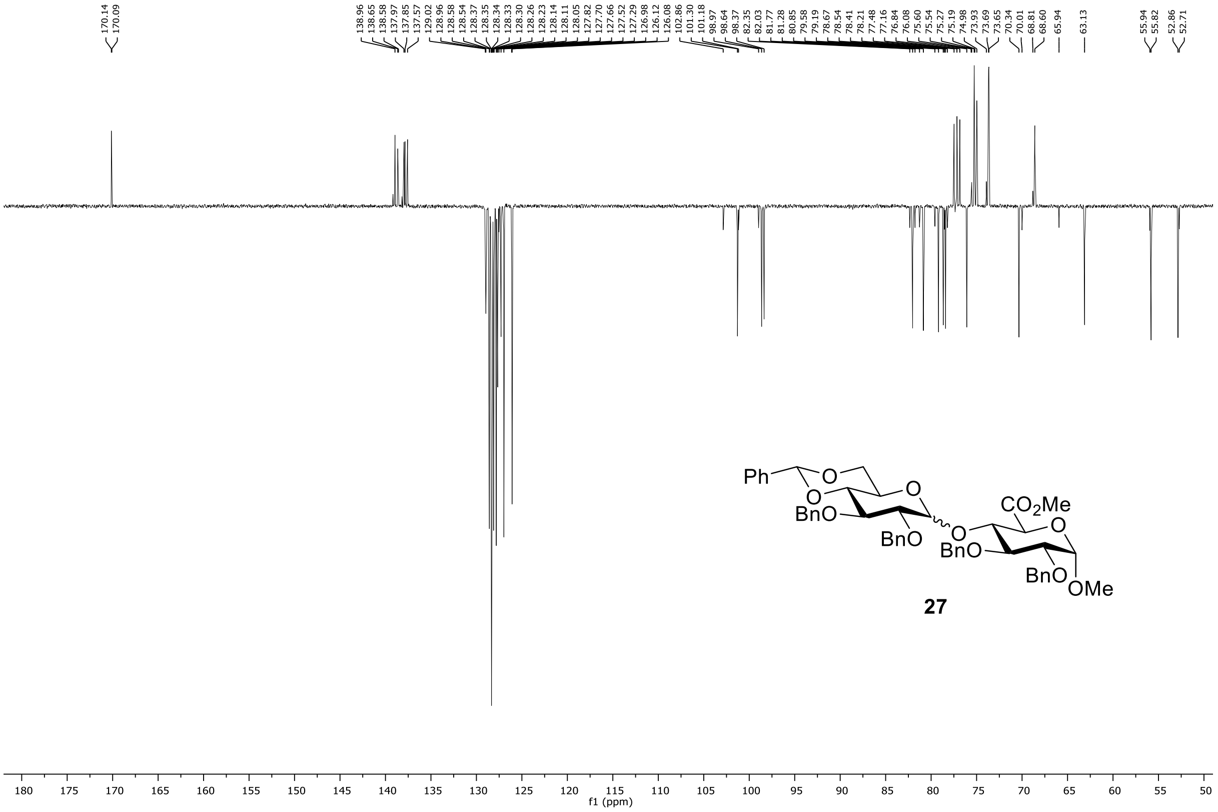

COSY NMR of compound **27**

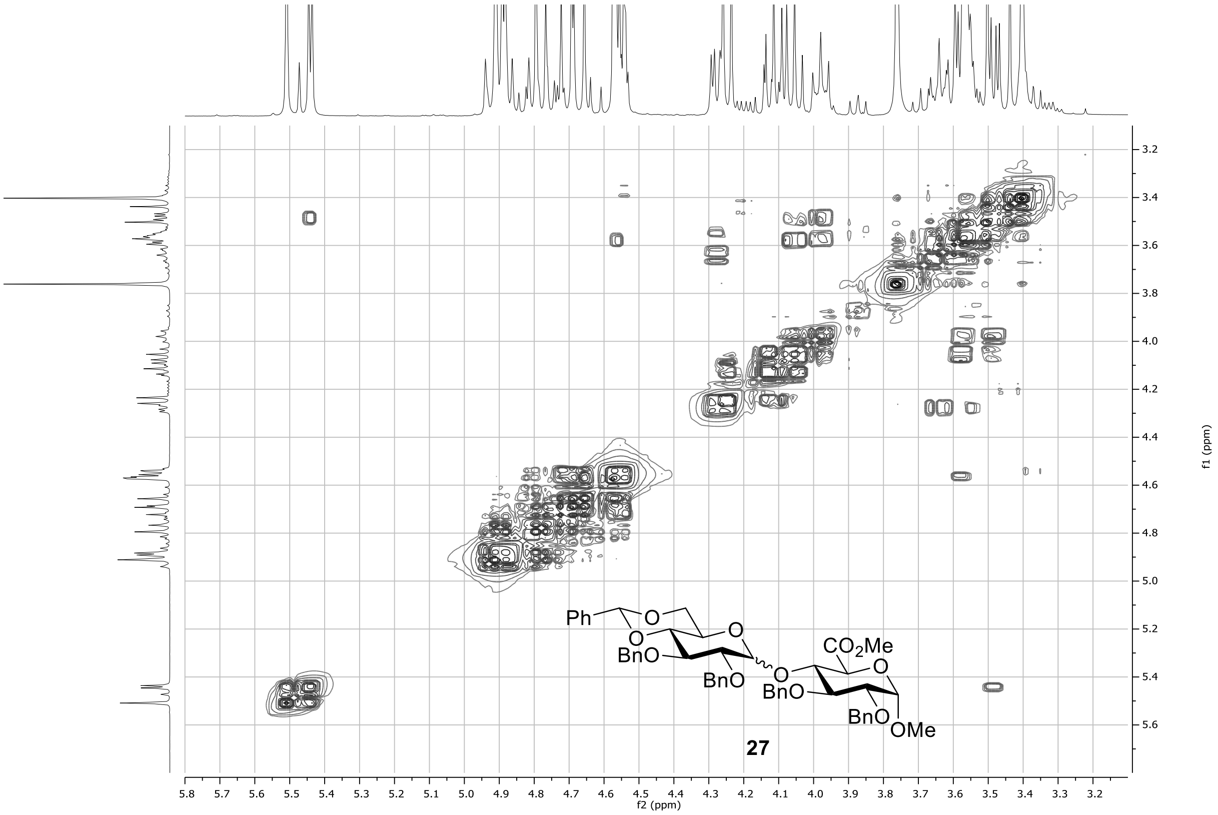

HSQC NMR of compound **27**

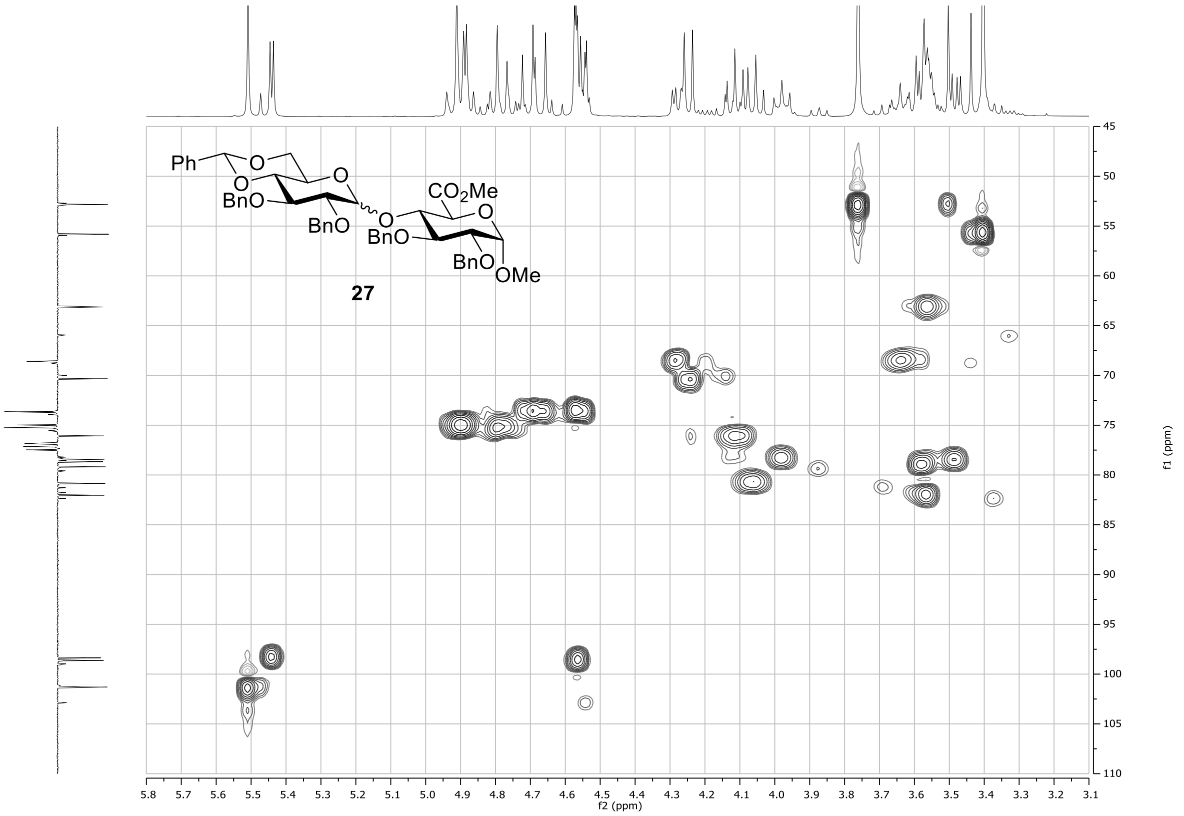

HMBC NMR of compound **27**

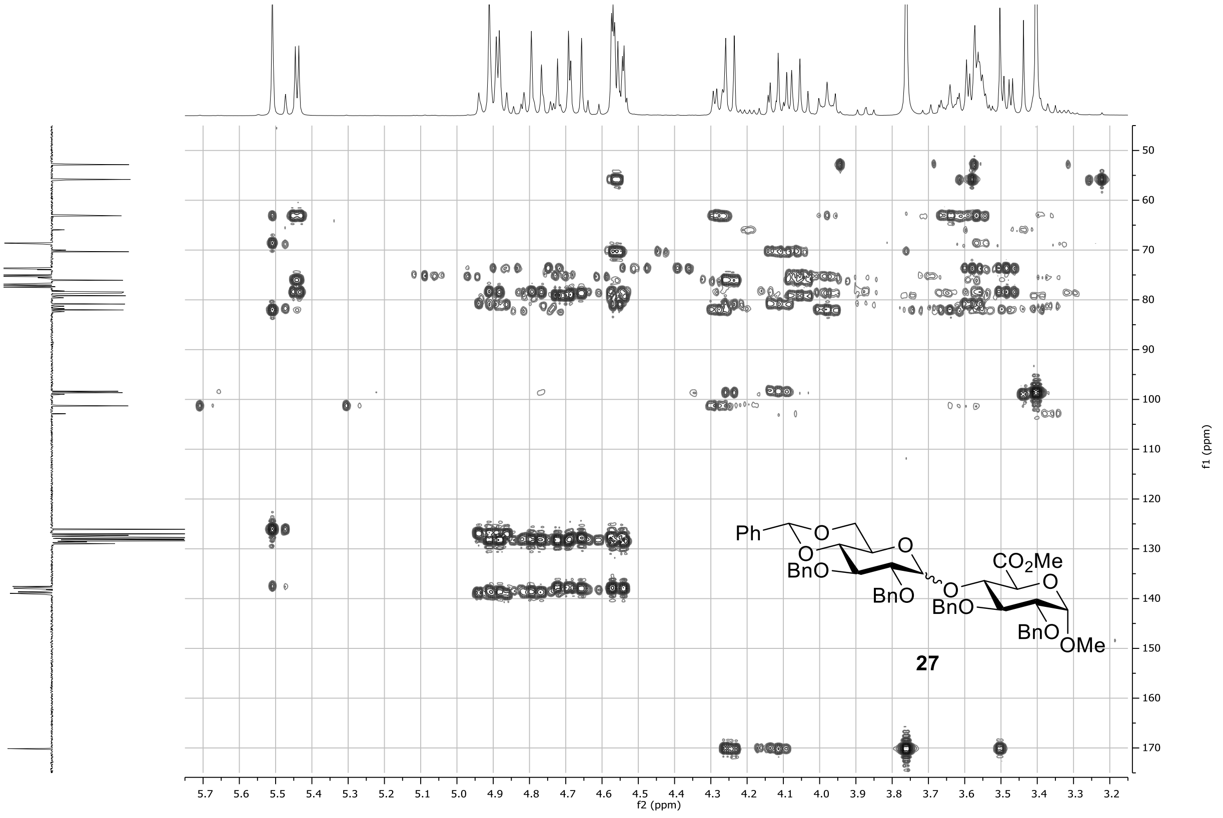

^{1}H NMR, 400 MHz, $CDCl_3$ of compound **28**

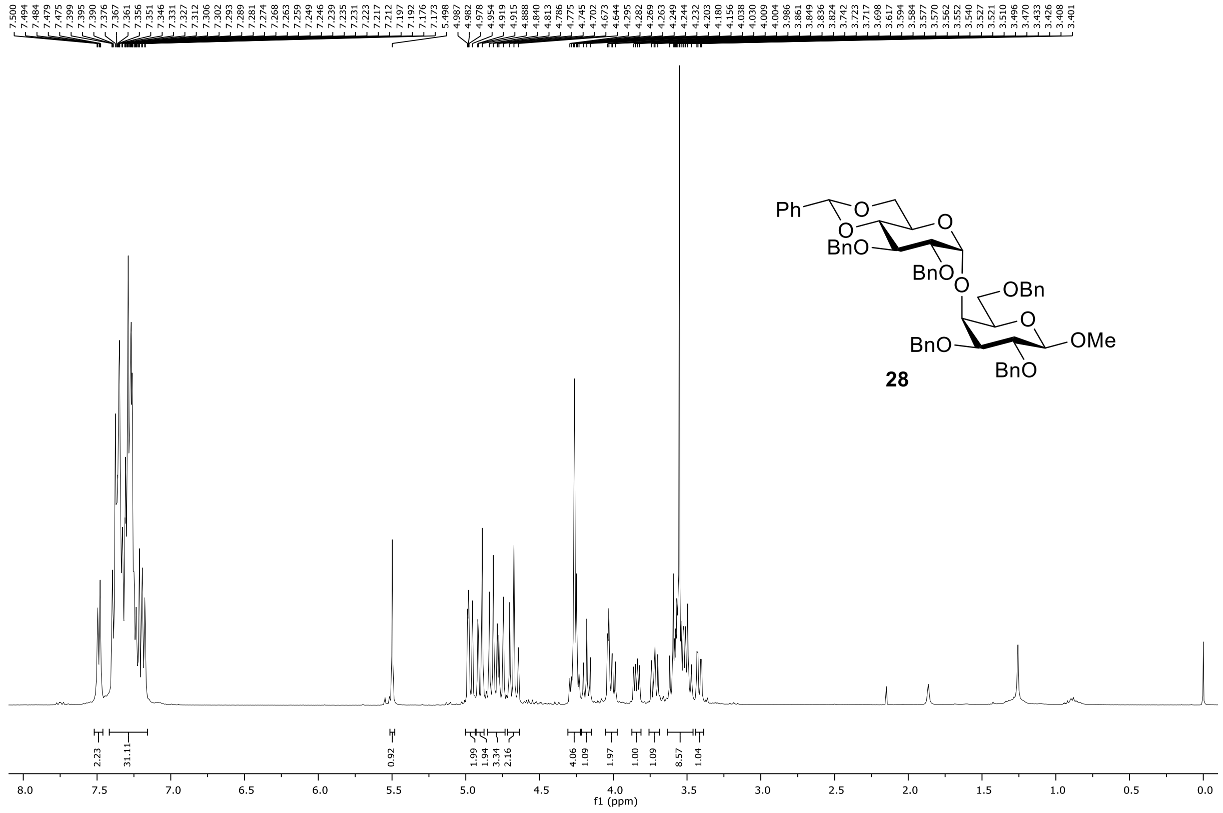

^{13}C-APT NMR, 101 MHz, $CDCl_3$ of compound **28**

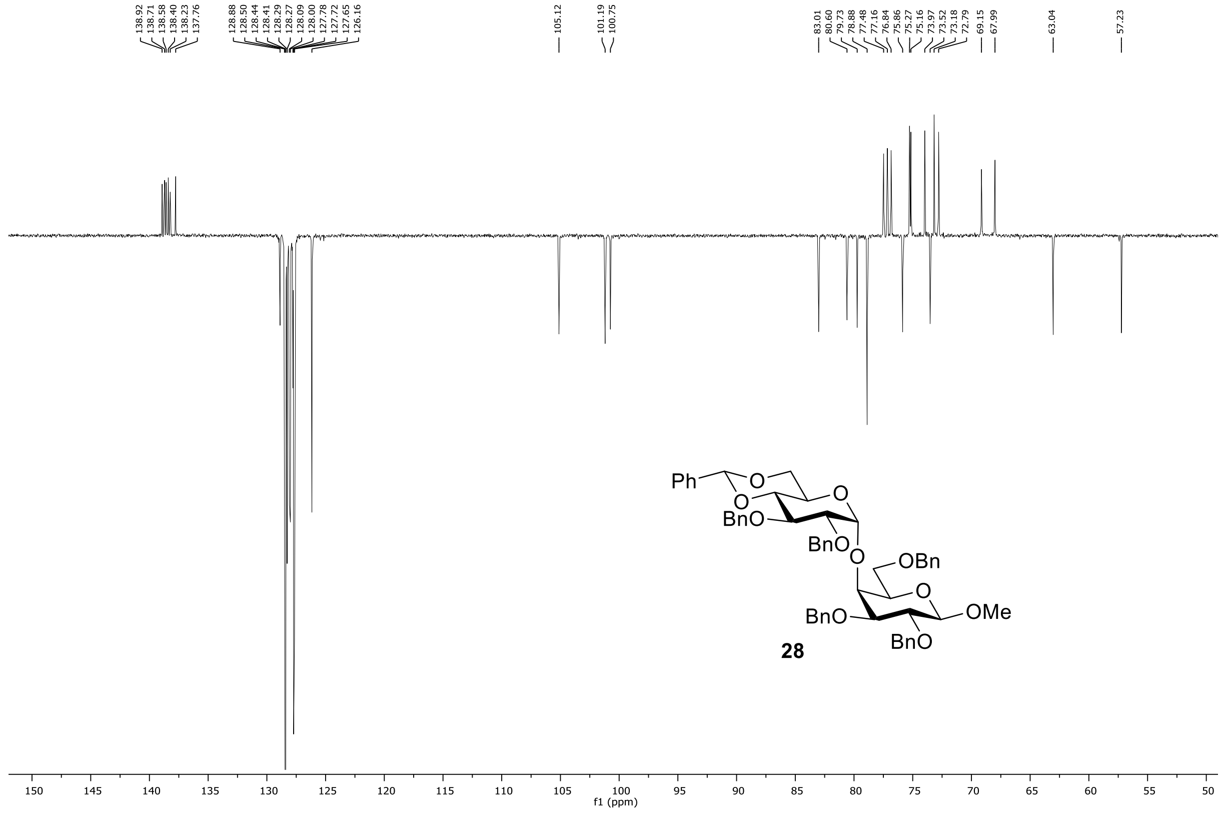

COSY NMR of compound **28**

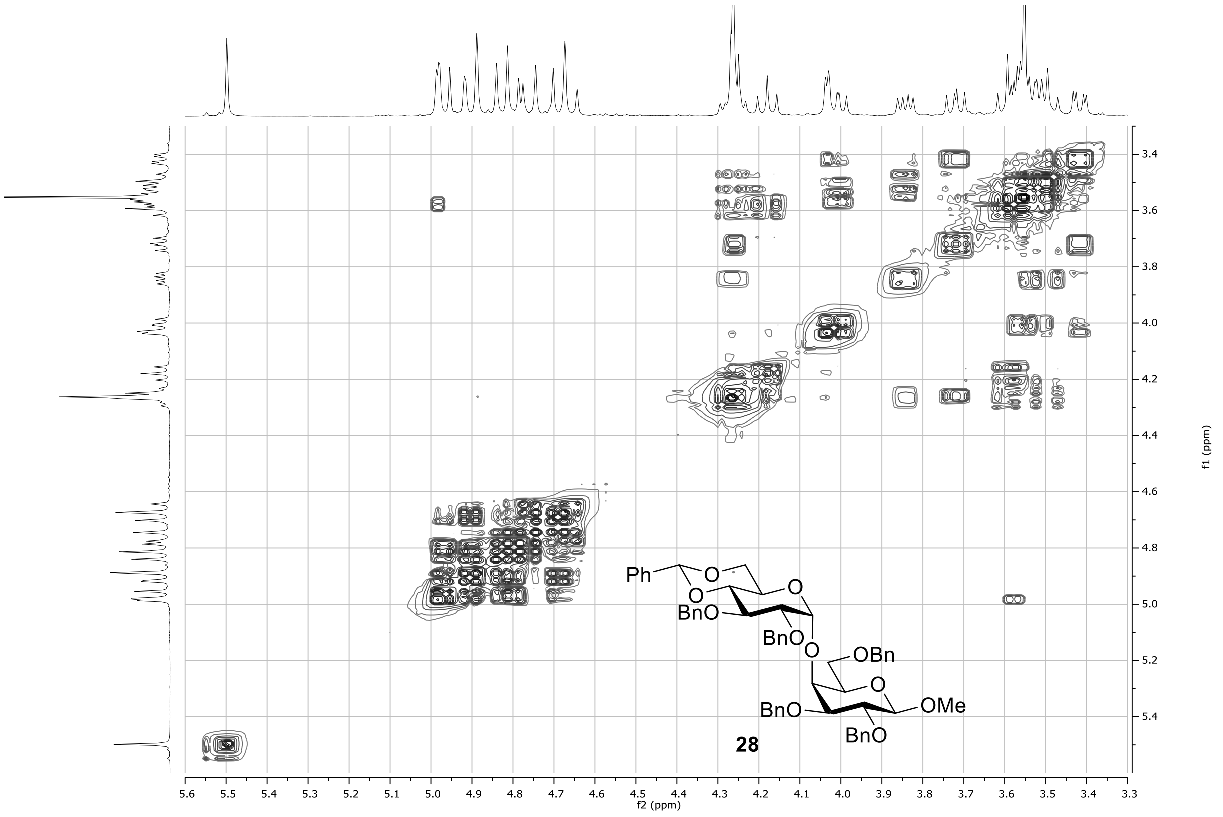

HSQC NMR of compound **28**

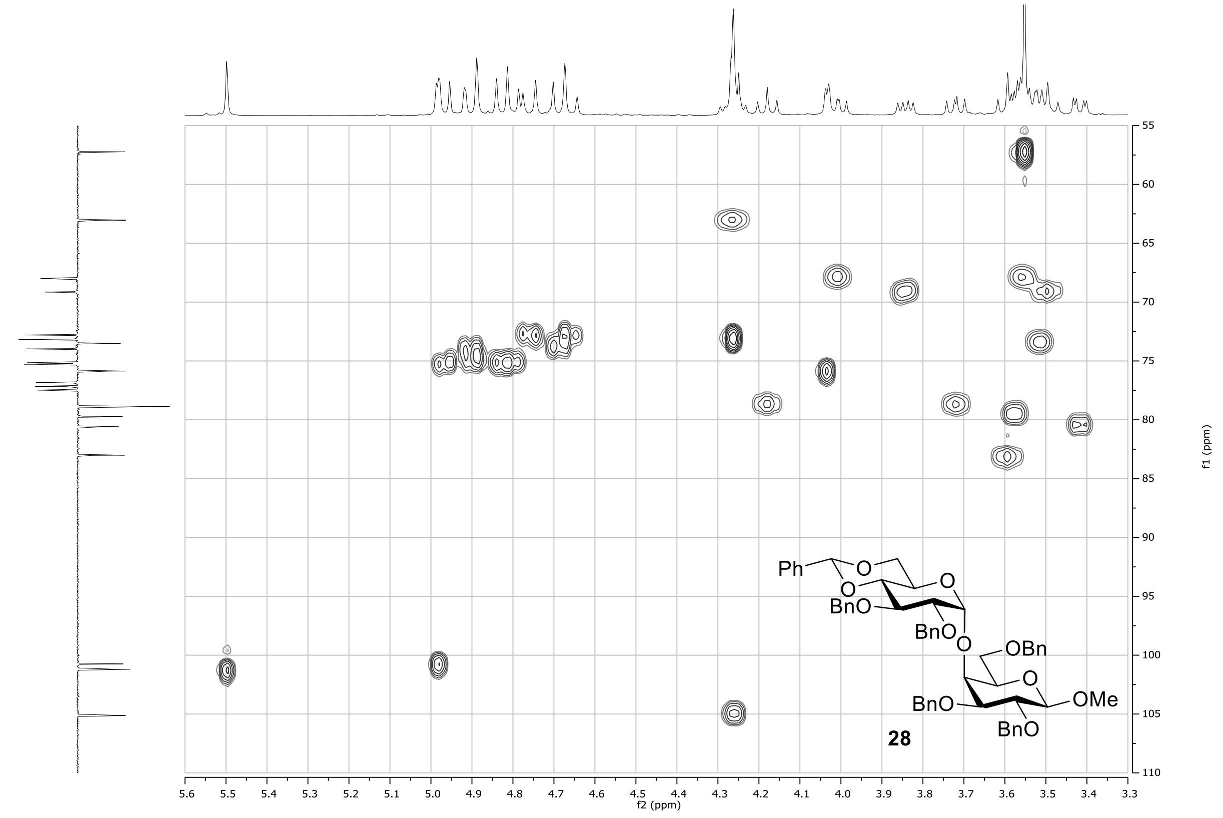

HMBC NMR of compound **28**

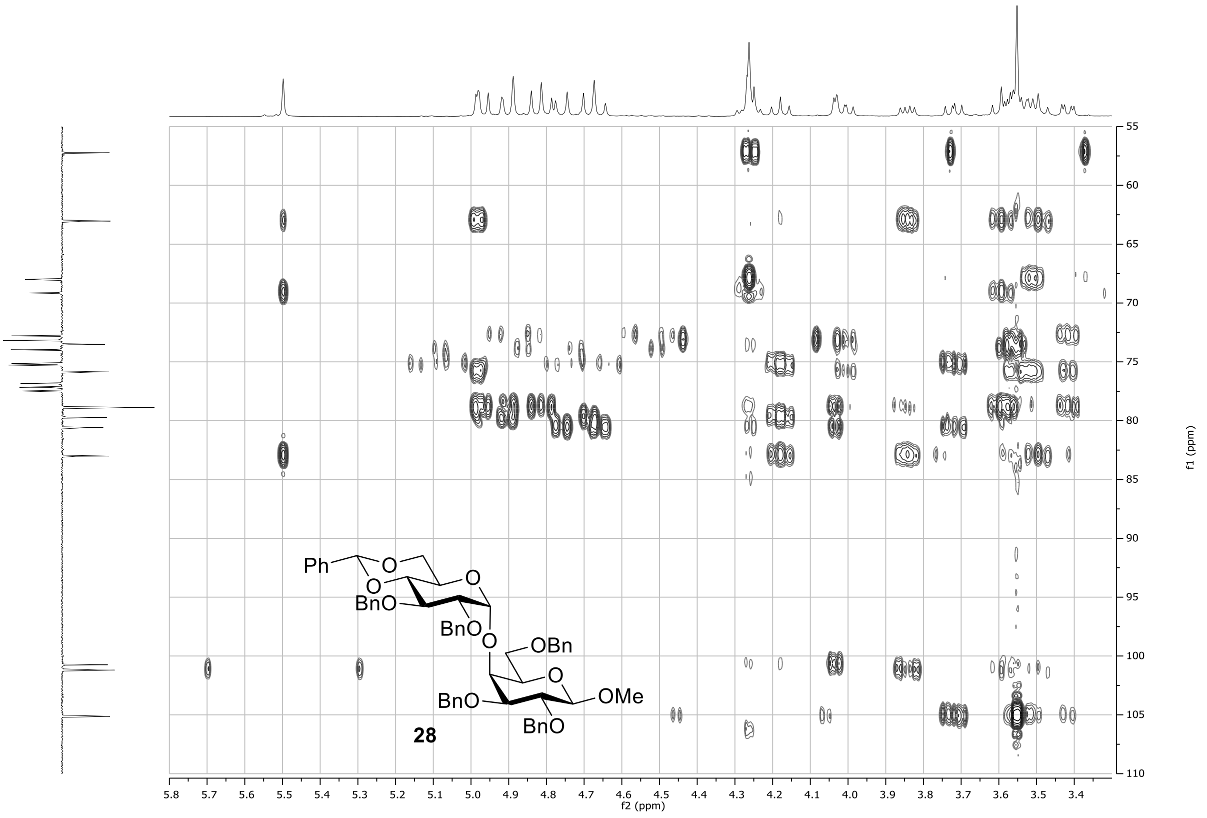

^{1}H NMR, 400 MHz, $CDCl_3$ of compound **3**

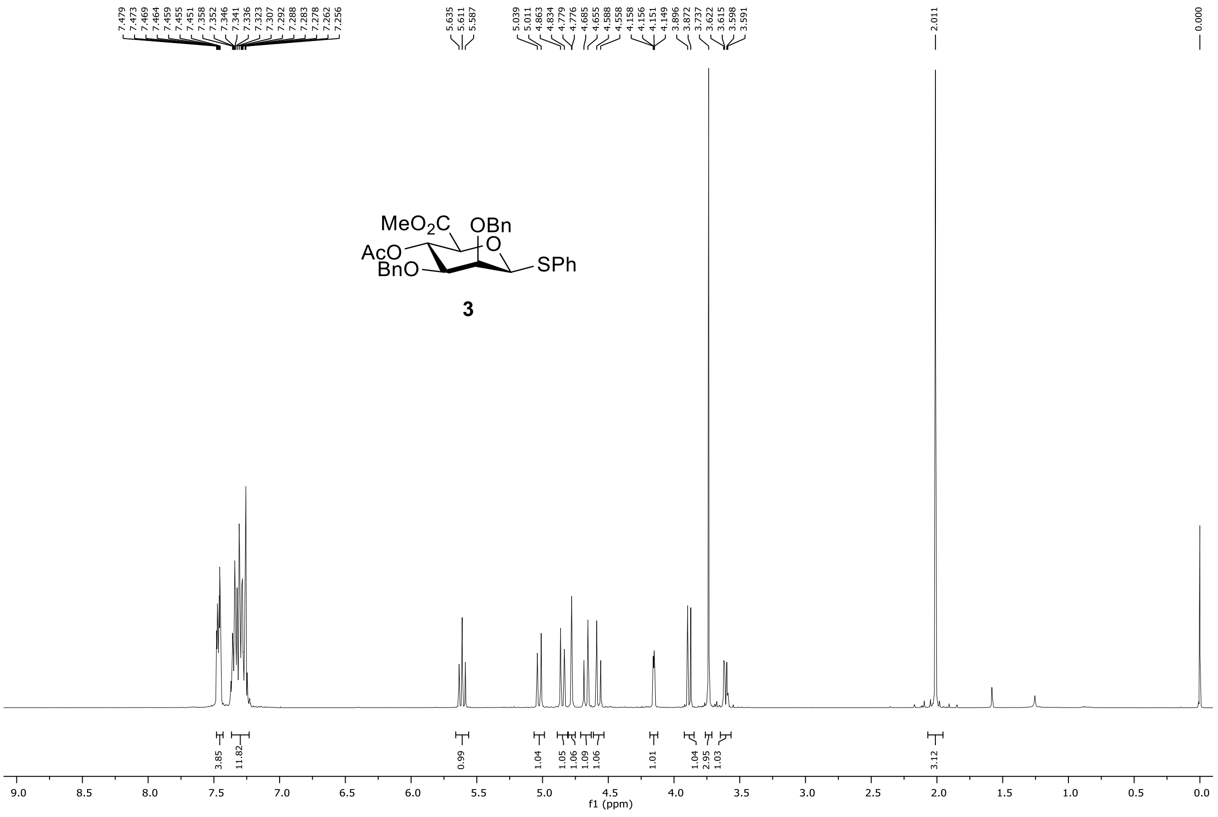

^{13}C-APT NMR, 101 MHz, $CDCl_3$ of compound **3**

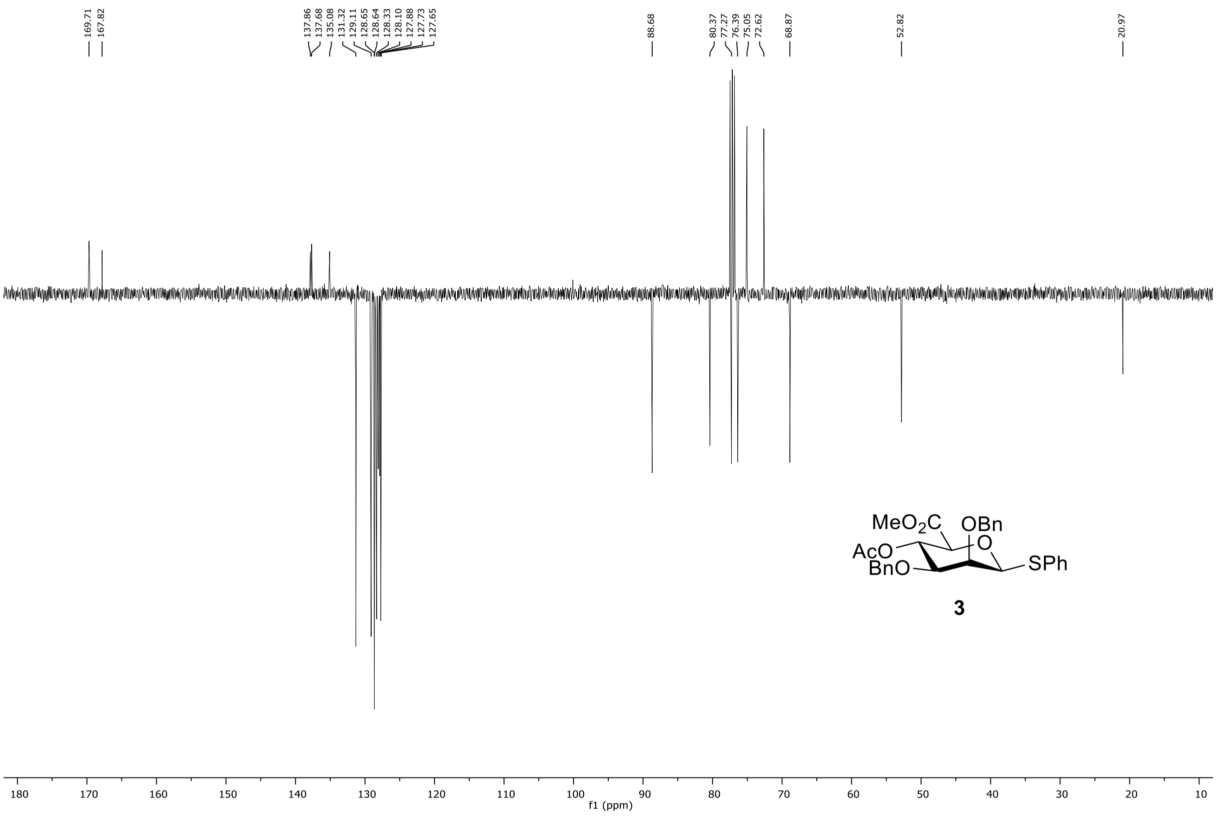

COSY NMR of compound **3**

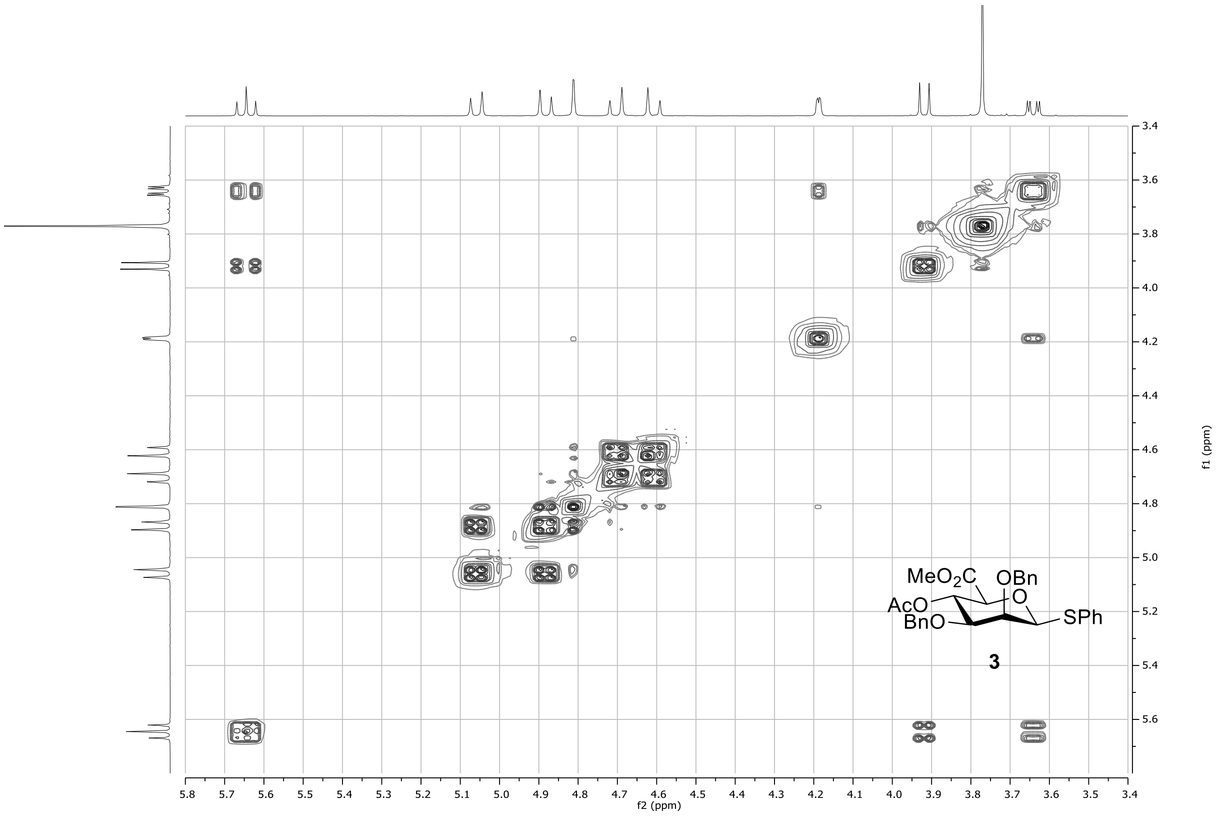

HSQC NMR of compound **3**

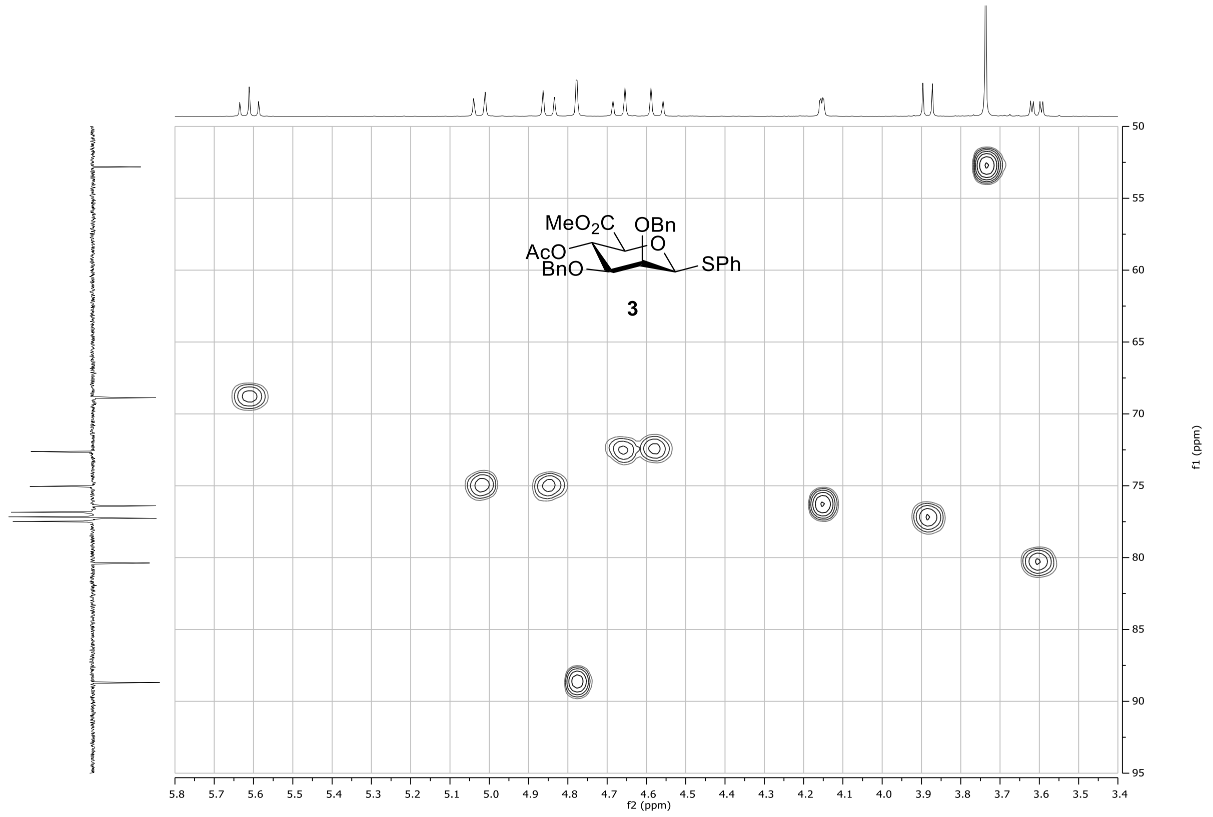

^{1}H NMR, 400 MHz, $CDCl_3$ of compound **3A**

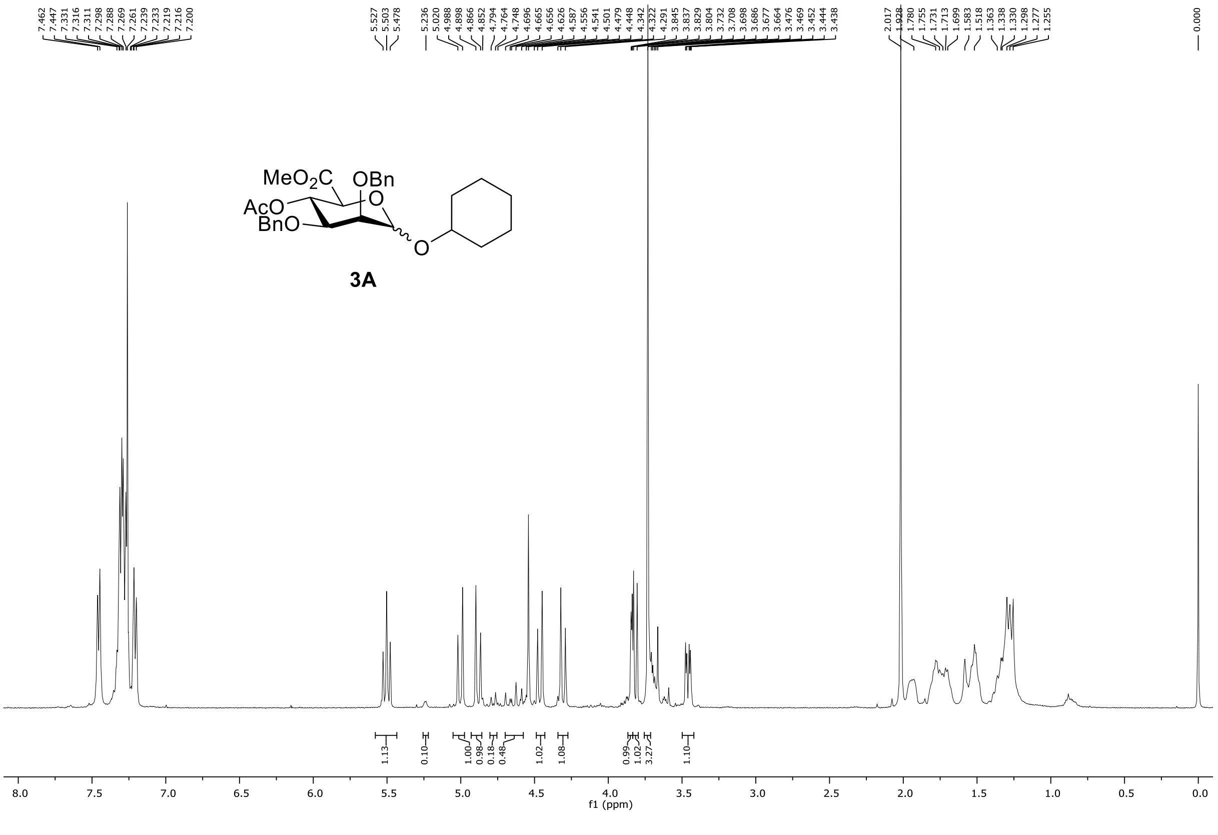

^{13}C-APT NMR, 101 MHz, CDCl$_3$ of compound **3A**

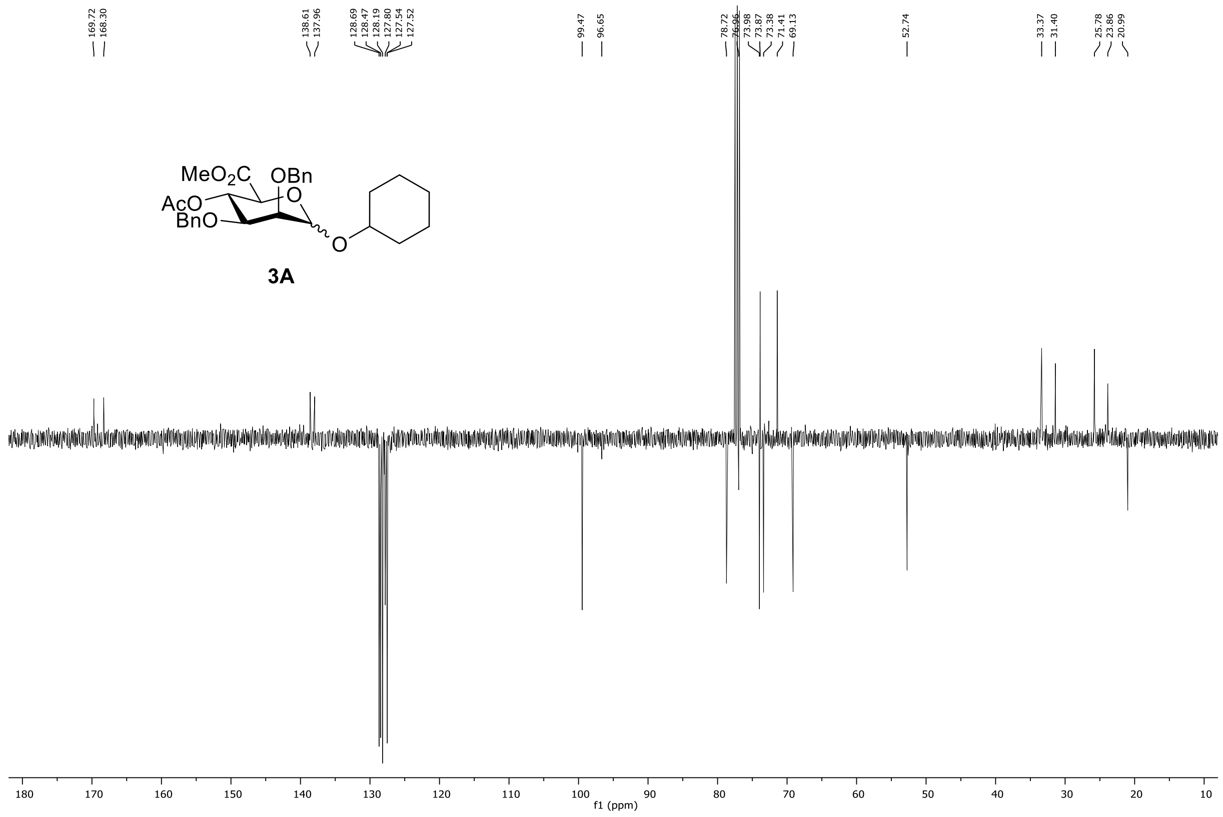

COSY NMR of compound **3A**

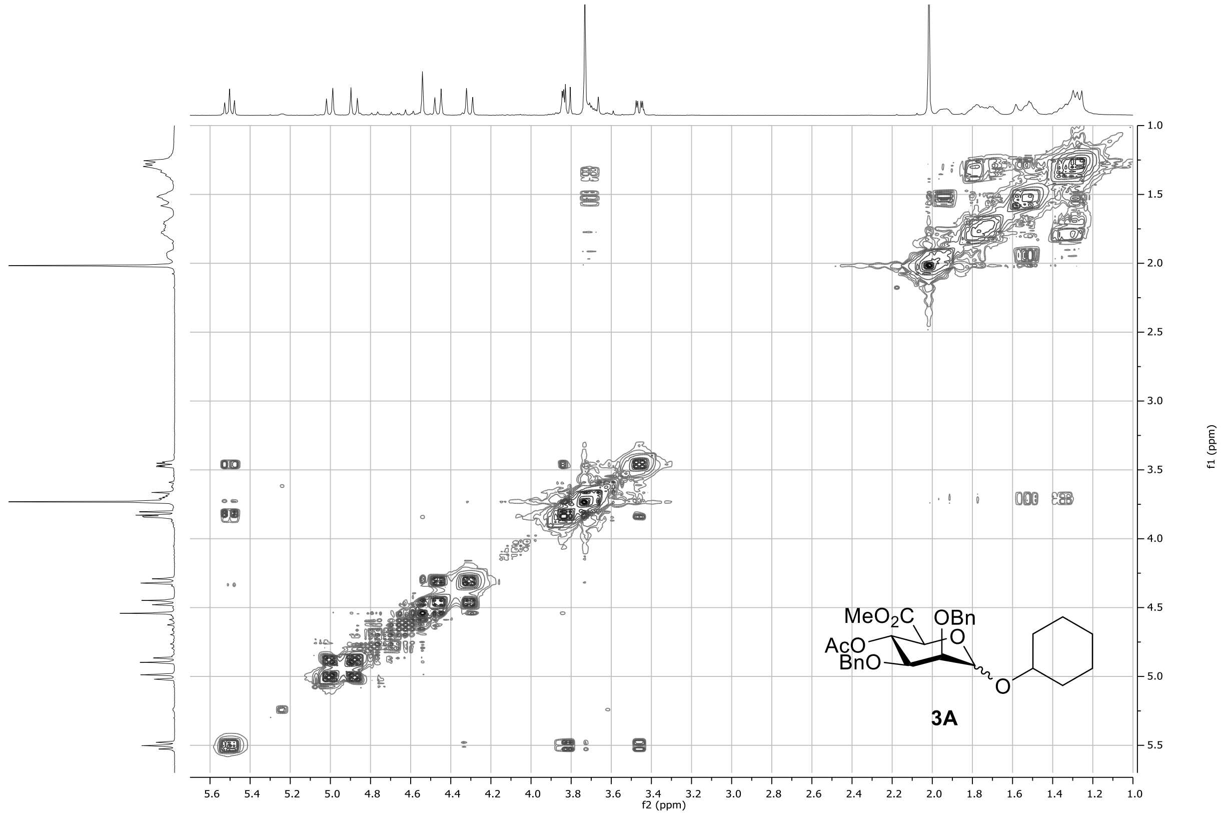

HSQC NMR of compound **3A**

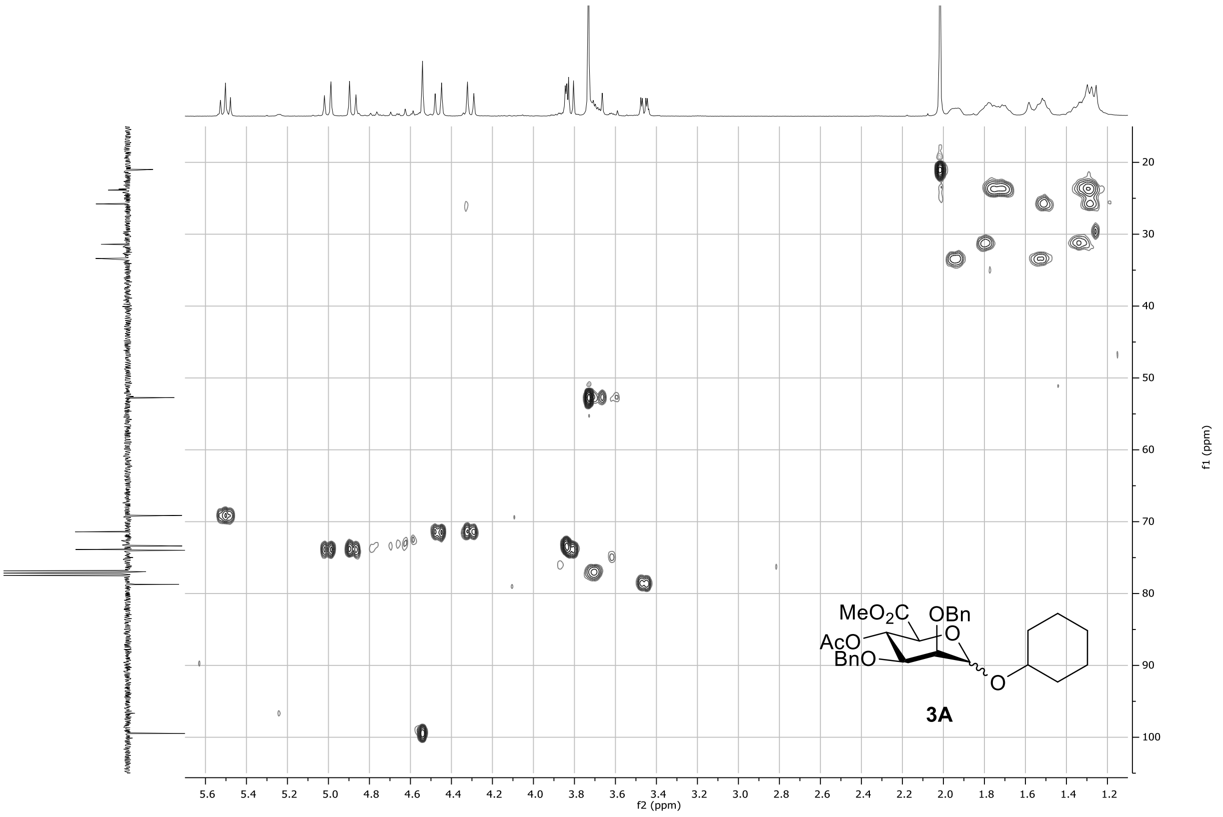

^{1}H NMR, 400 MHz, $CDCl_3$ of compound **3B**

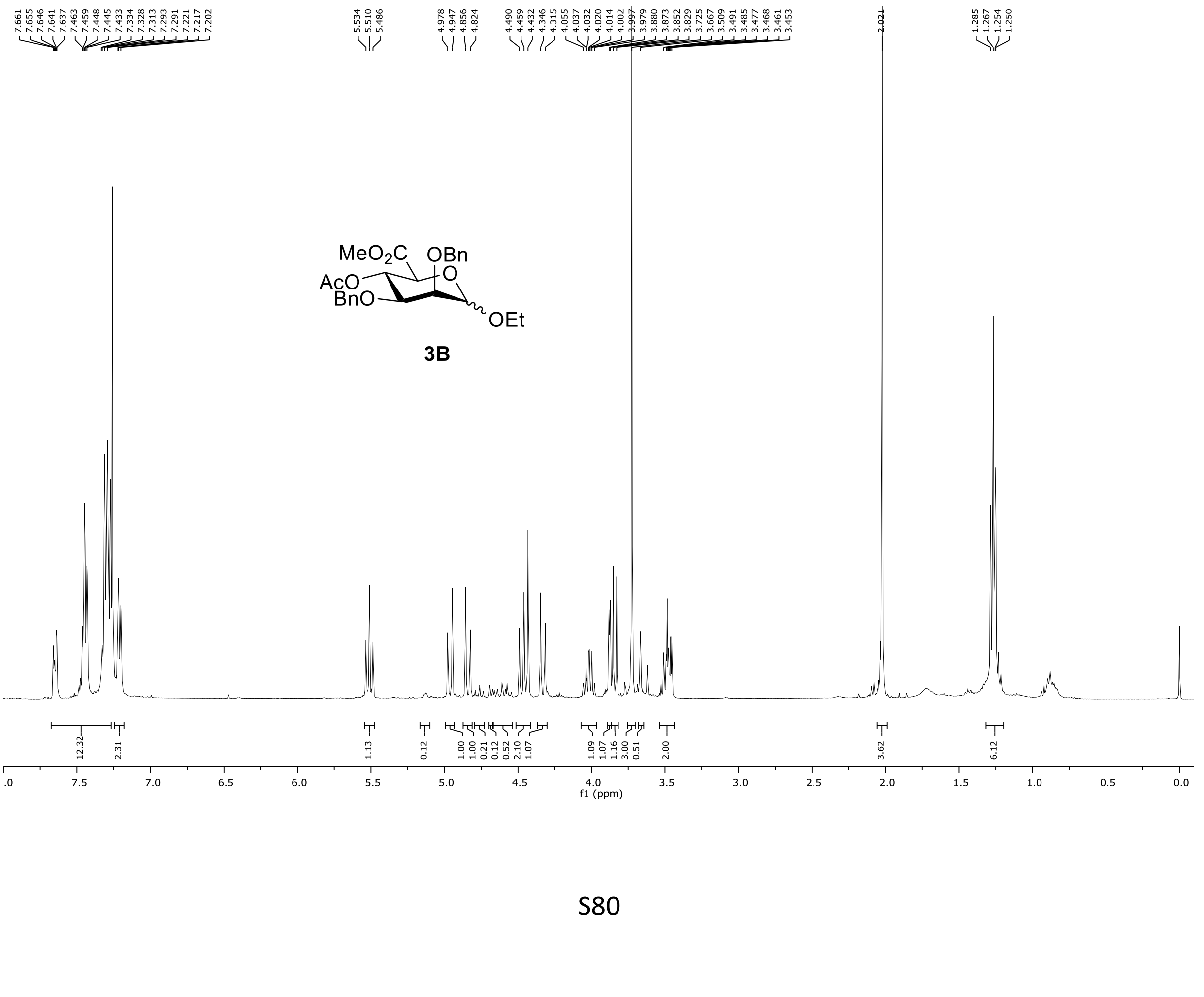

^{13}C-APT NMR, 101 MHz, $CDCl_3$ of compound **3B**

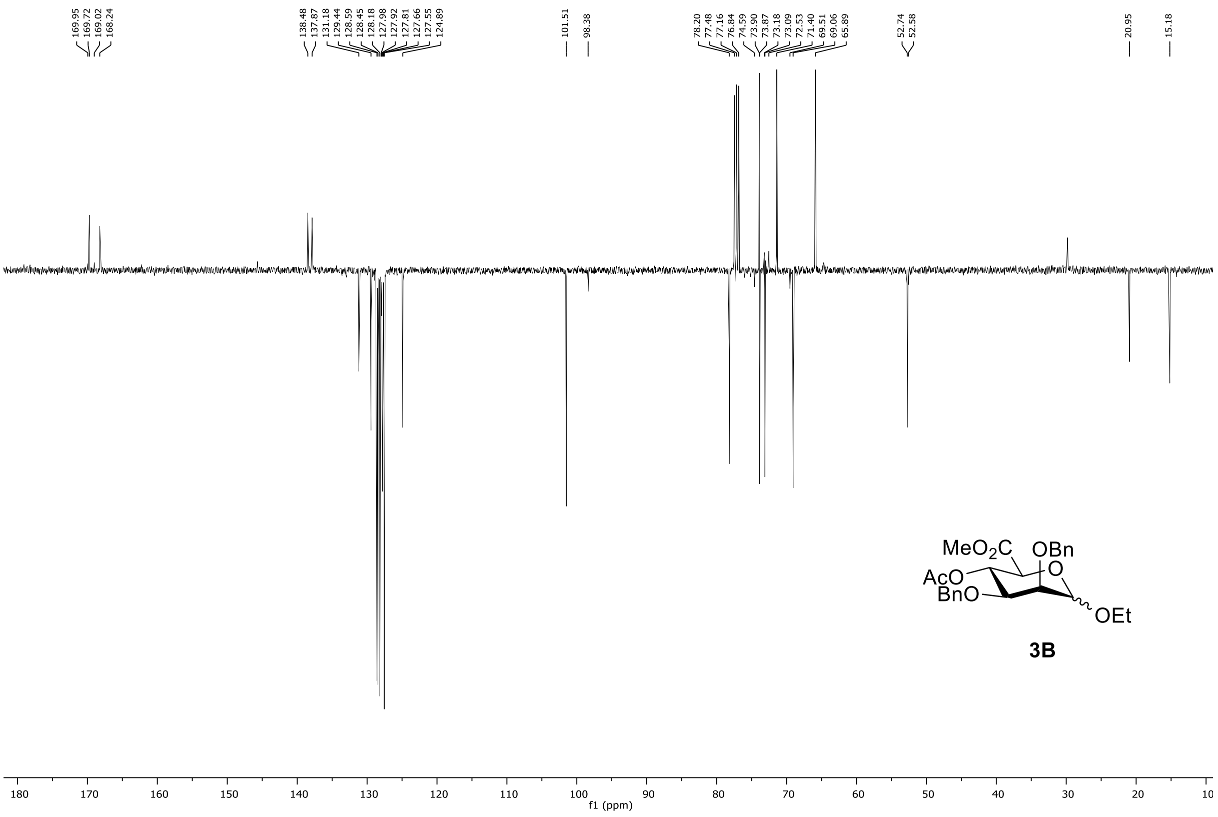

COSY NMR of compound **3B**

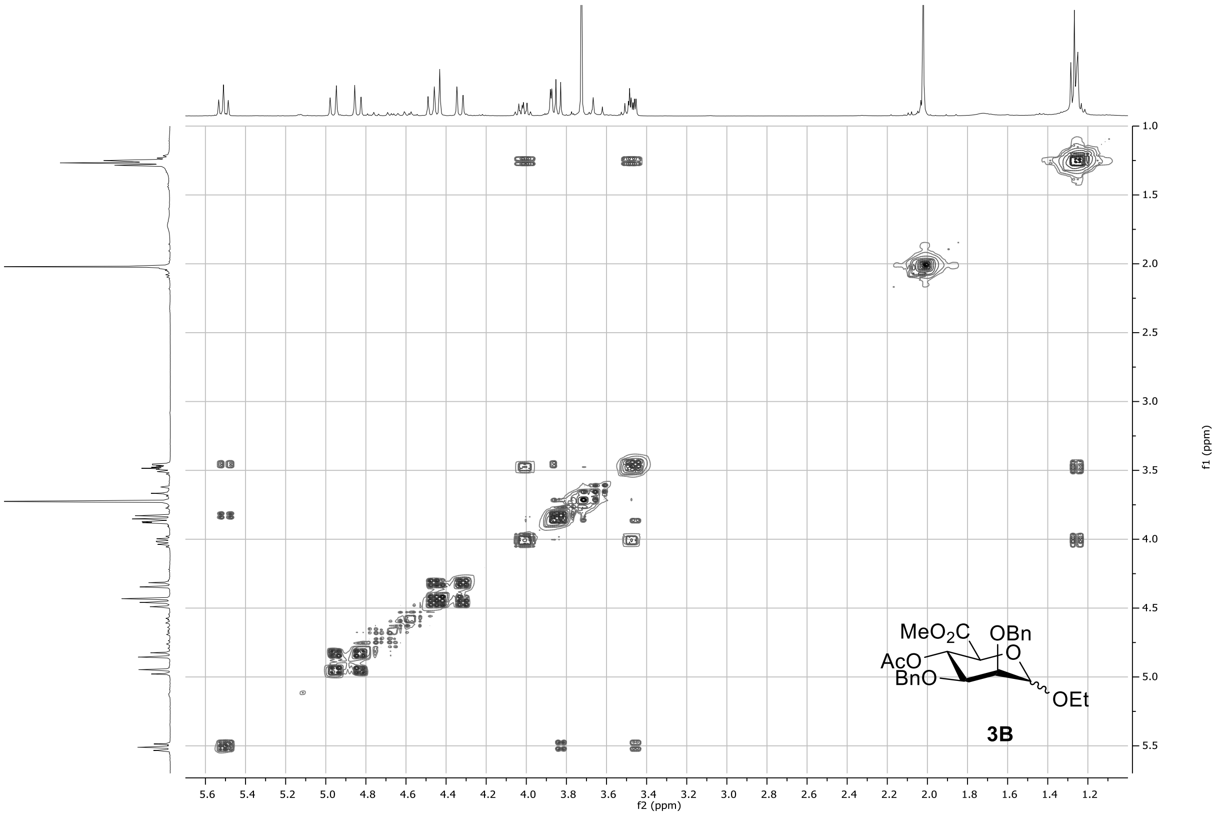

HSQC NMR of compound **3B**

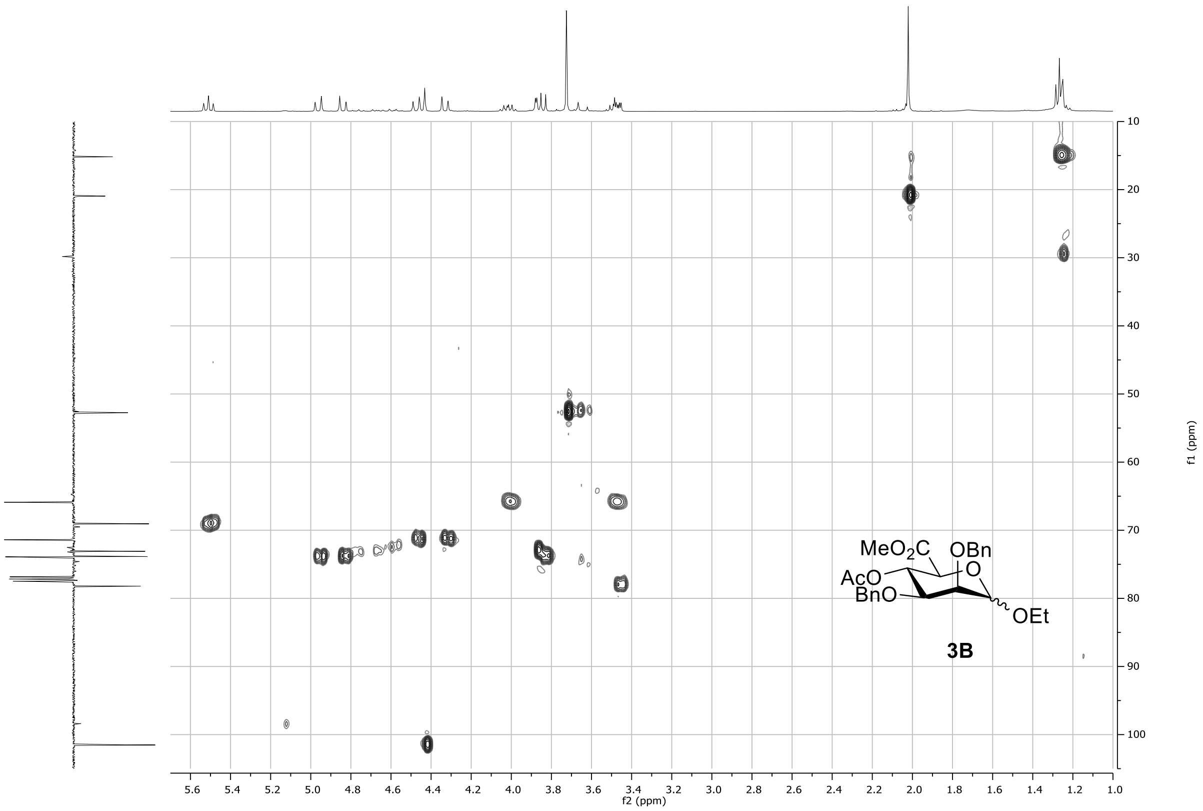

^{13}C GATED NMR, 101 MHz, $CDCl_3$ of compound **3B**

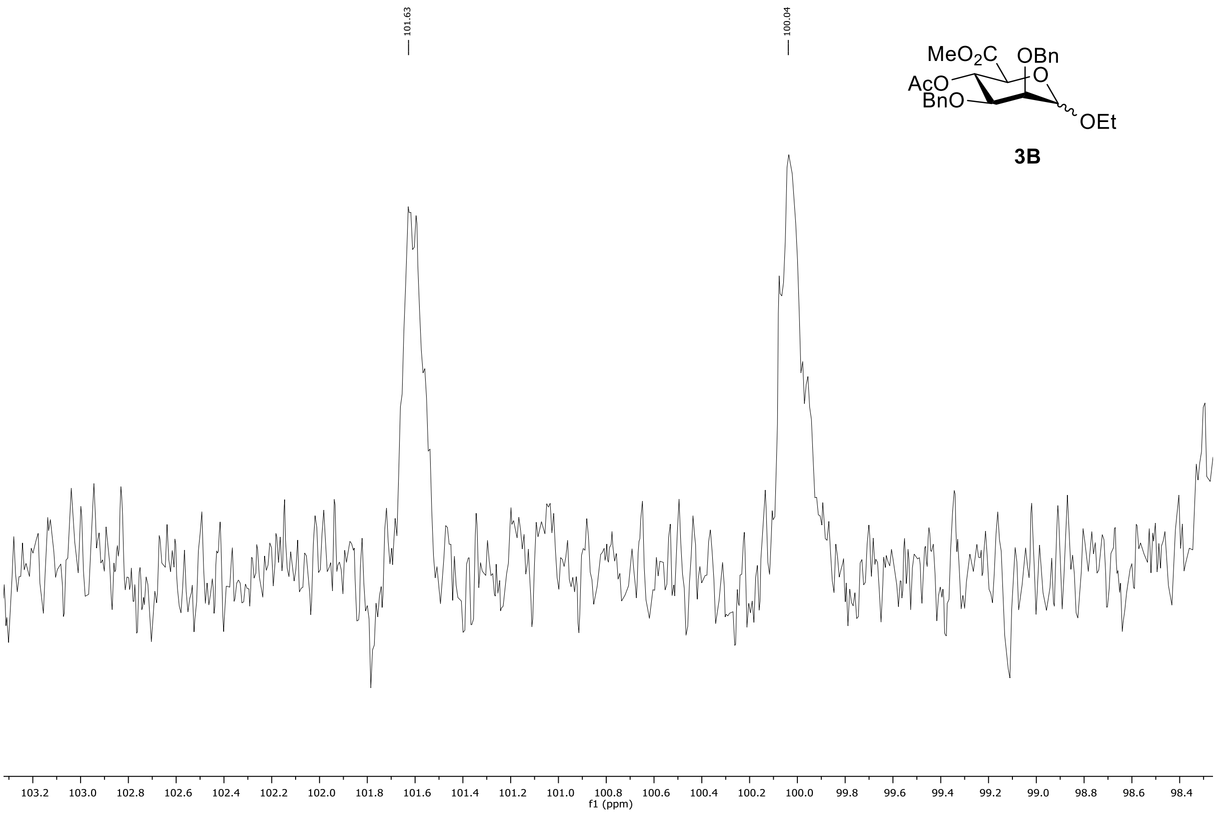

^{1}H NMR, 400 MHz, $CDCl_3$ of compound **3C**

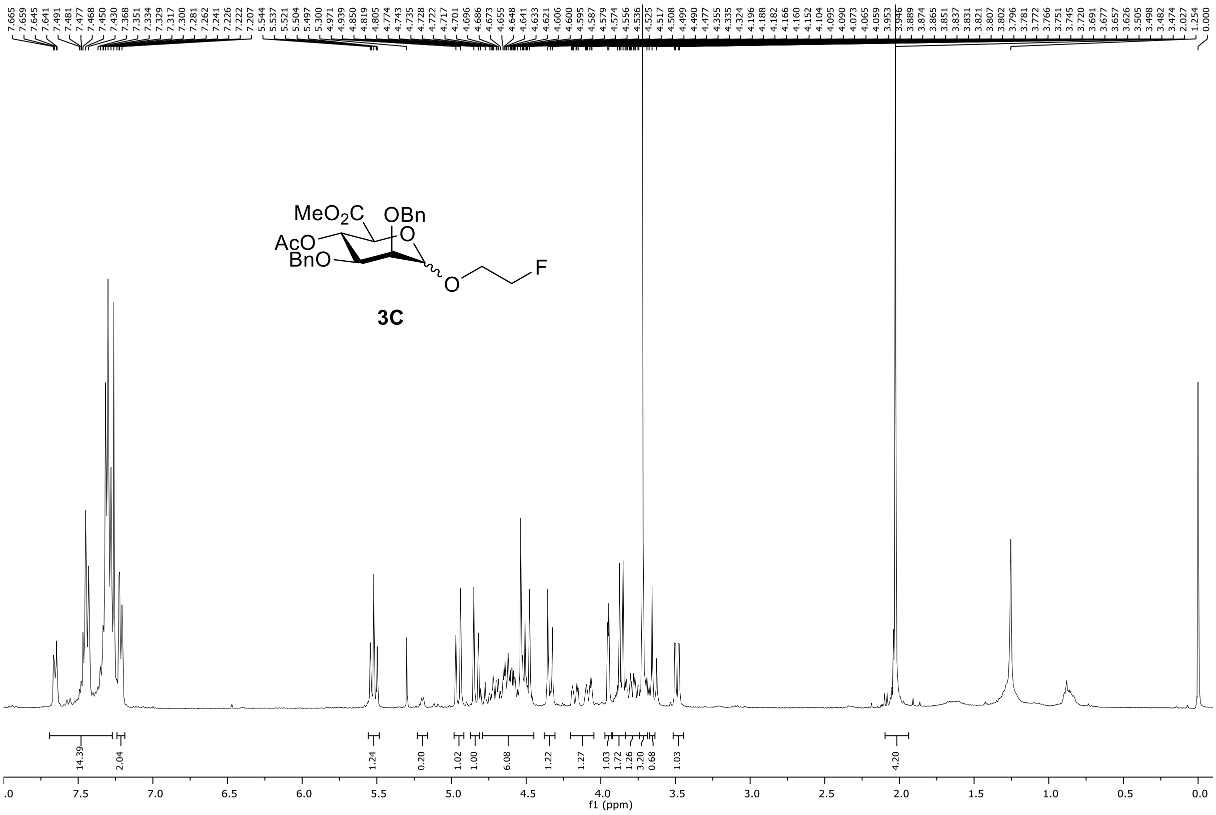

^{13}C-APT NMR, 101 MHz, $CDCl_3$ of compound **3C**

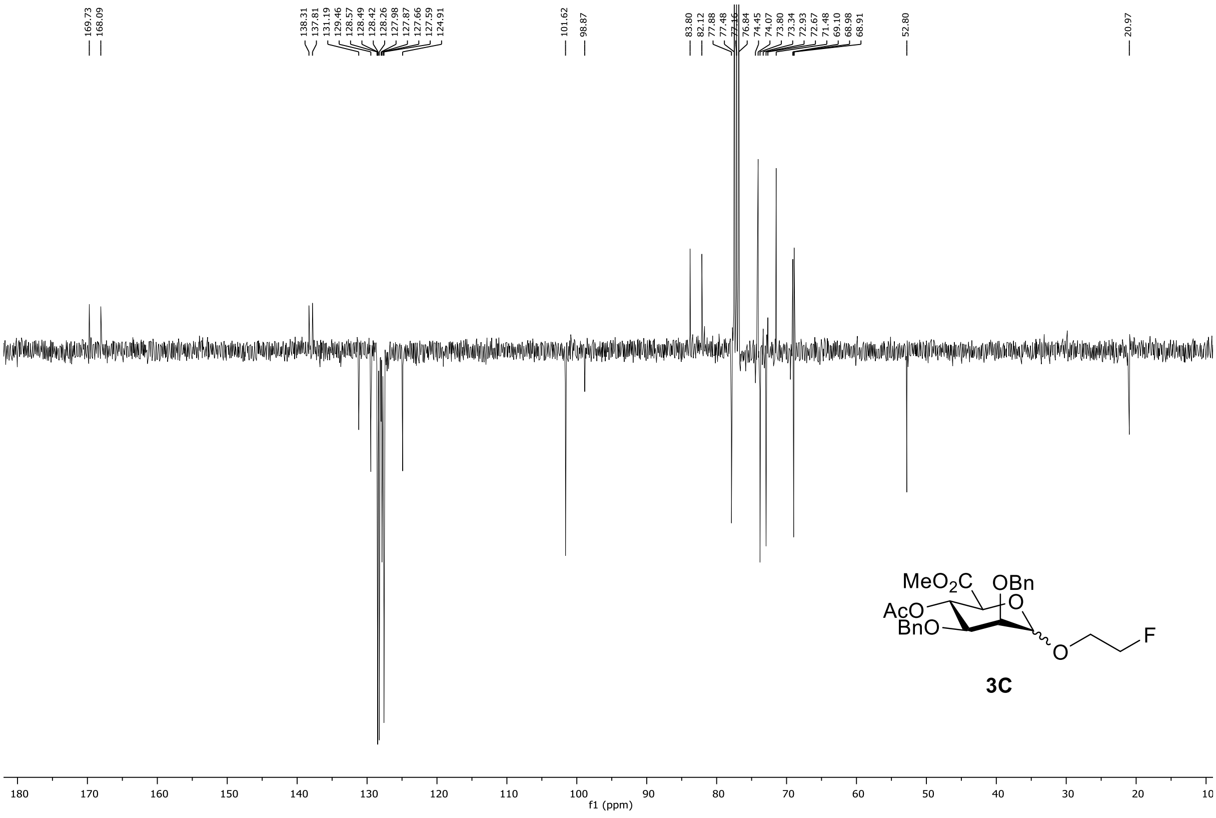

COSY NMR of compound **3C**

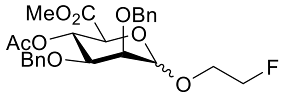

3C

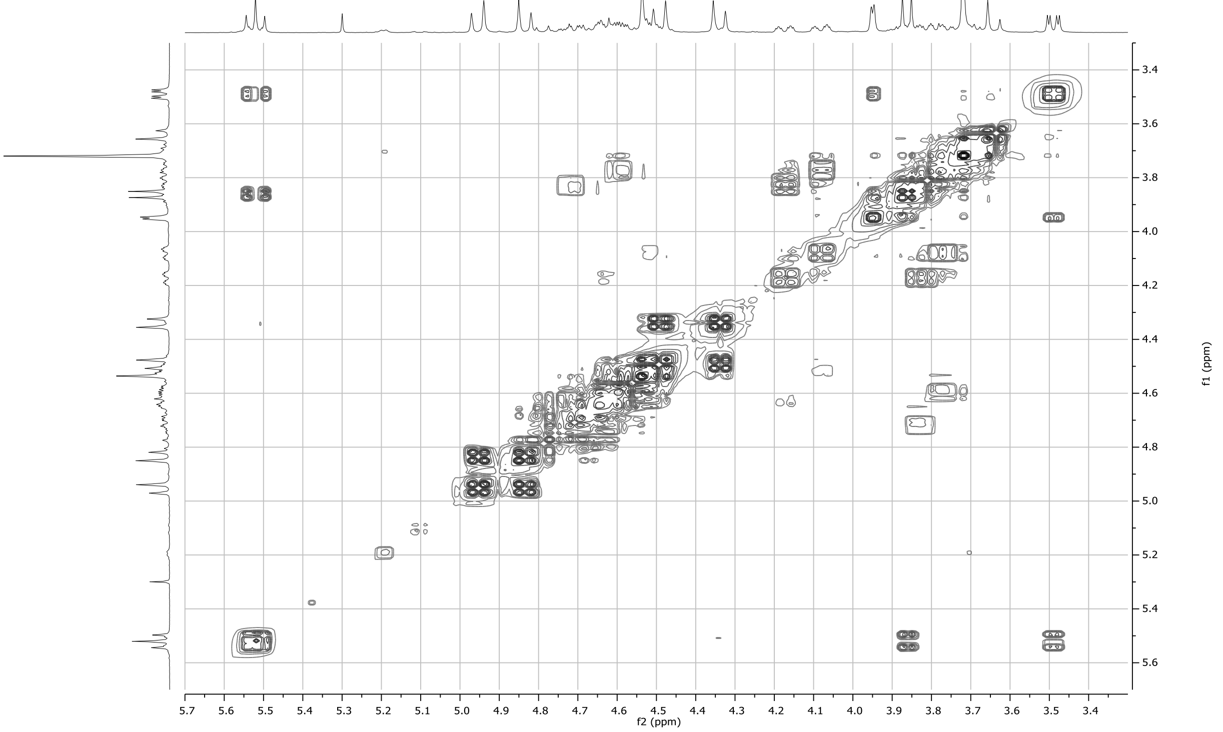

HSQC NMR of compound **3C**

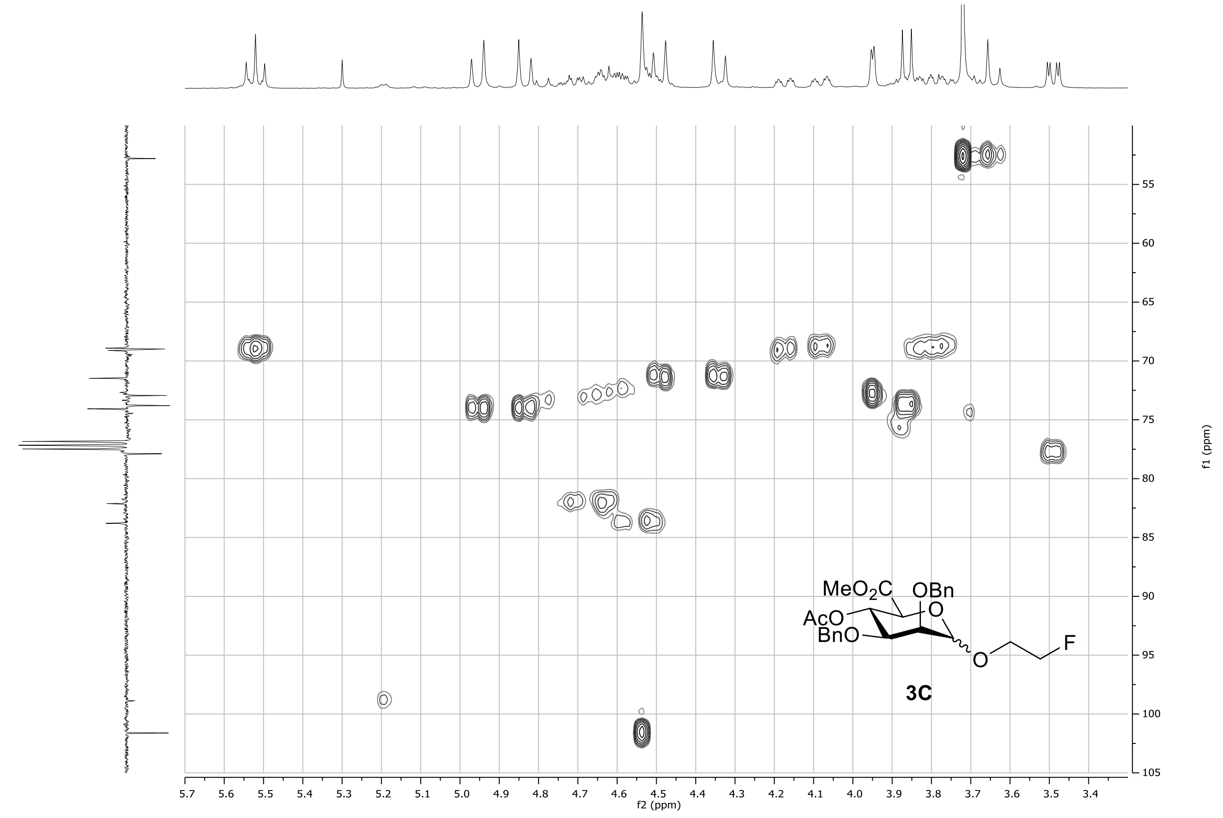

^{13}C GATED NMR, 101 MHz, $CDCl_3$ of compound **3C**

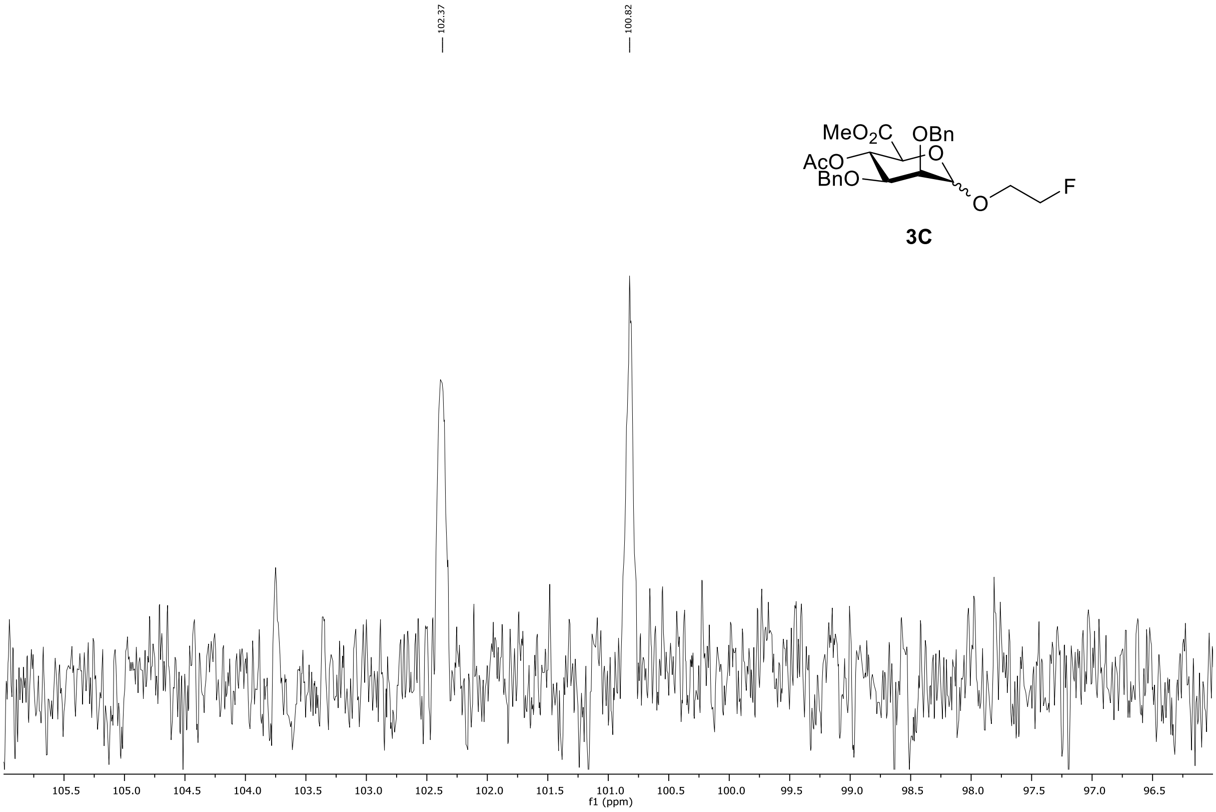

^{1}H NMR, 400 MHz, $CDCl_3$ of compound **3D**

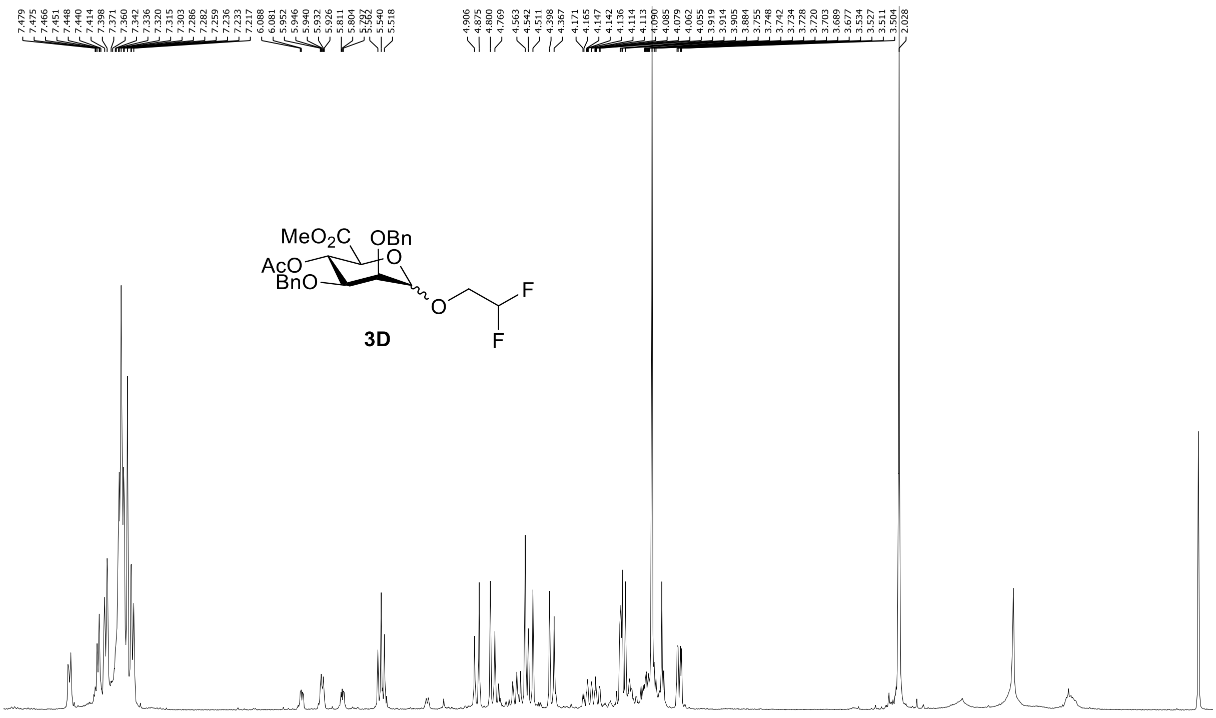

^{13}C-APT NMR, 101 MHz, $CDCl_3$ of compound **3D**

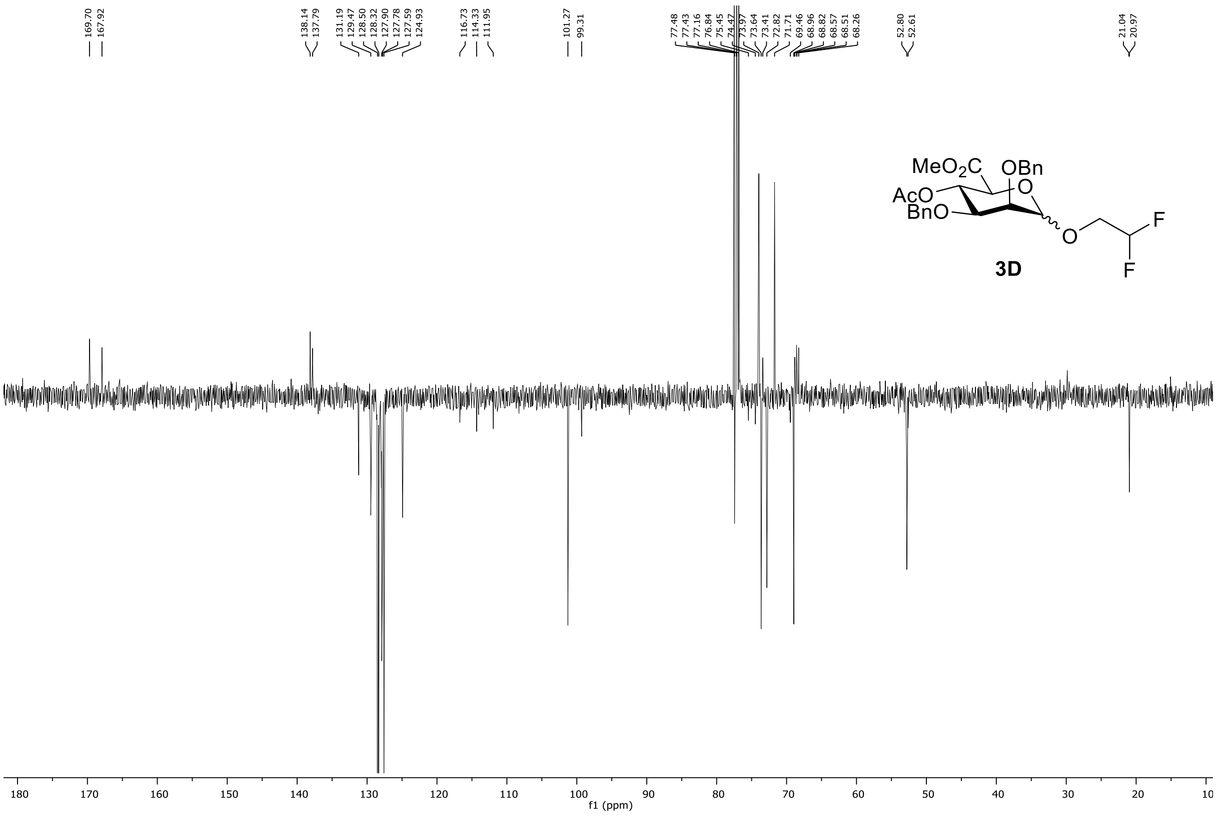

COSY NMR of compound **3D**

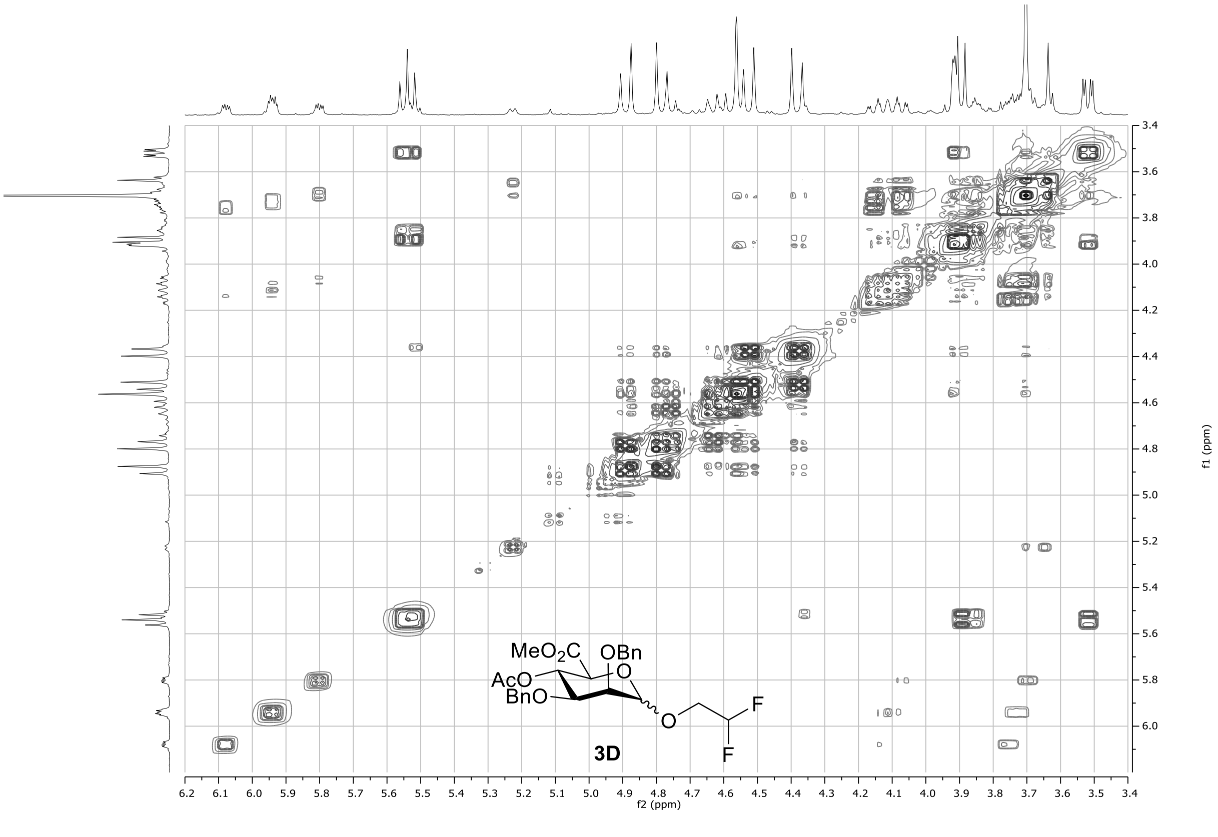

HSQC NMR of compound **3D**

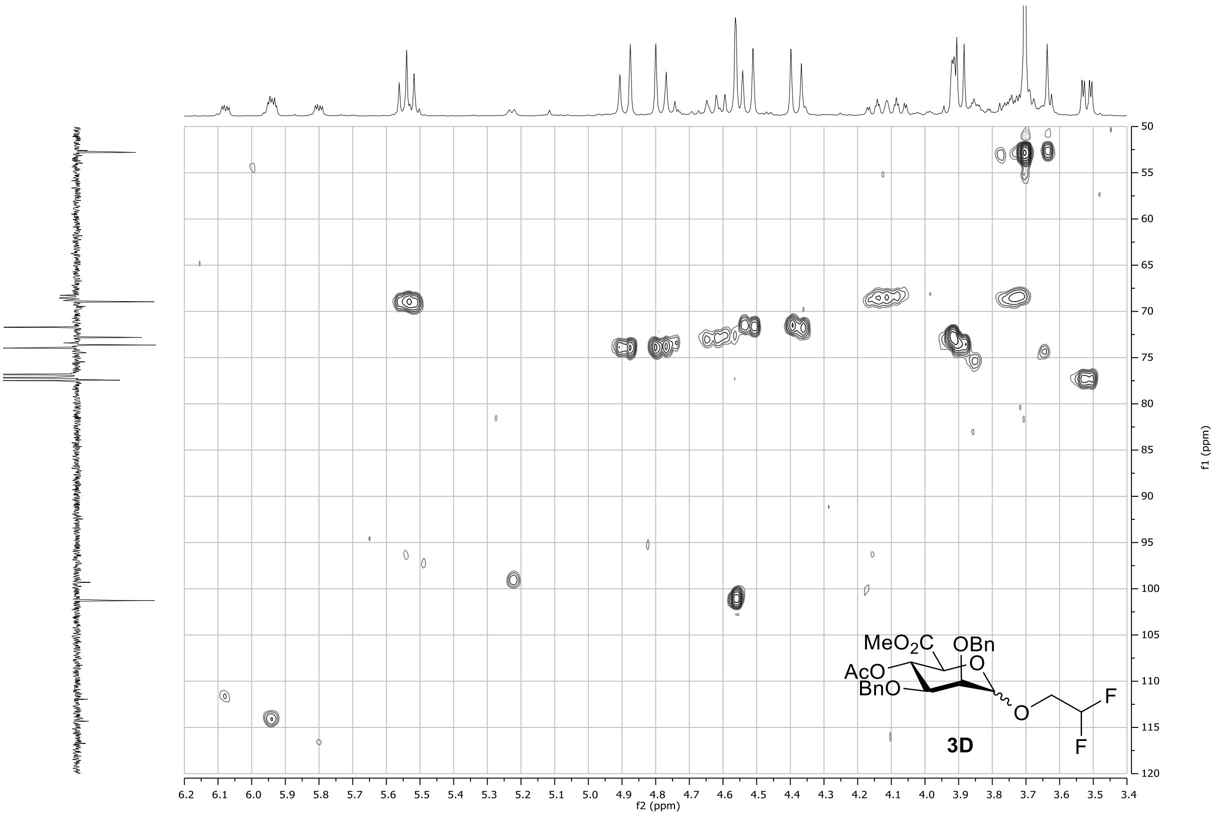

^{13}C GATED NMR, 101 MHz, $CDCl_3$ of compound **3D**

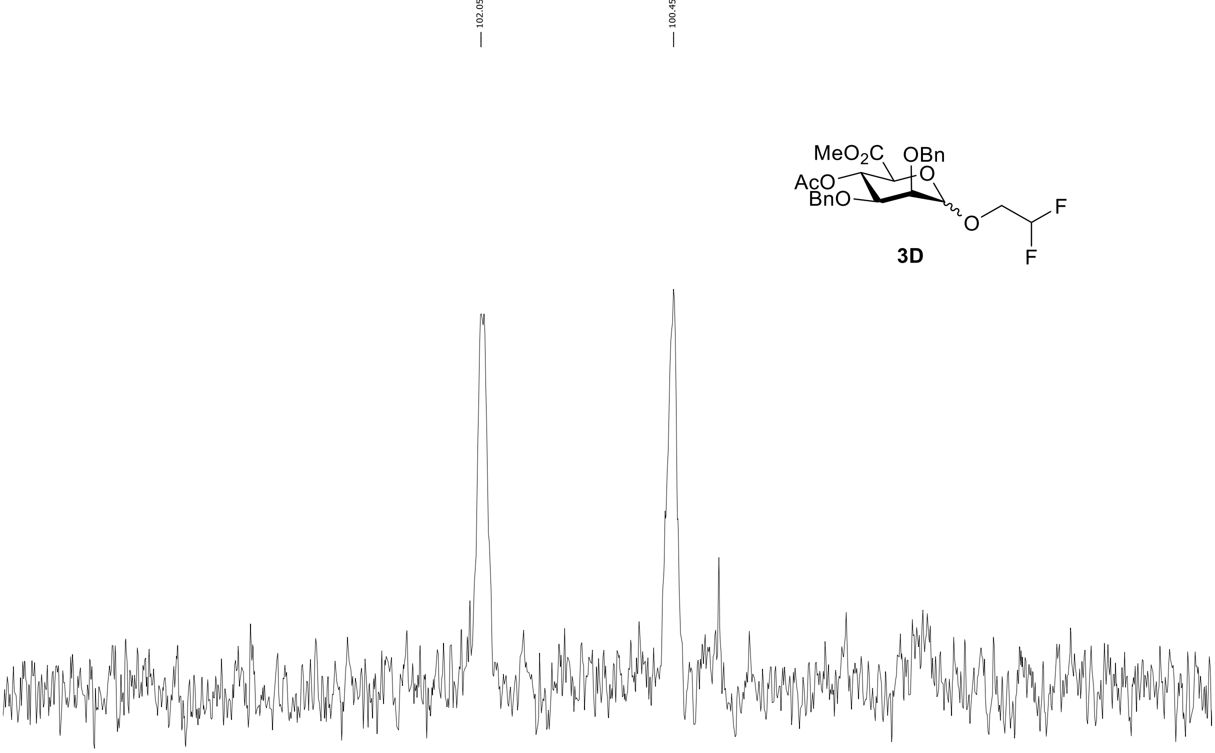

^{1}H NMR, 400 MHz, $CDCl_3$ of compound **3E**

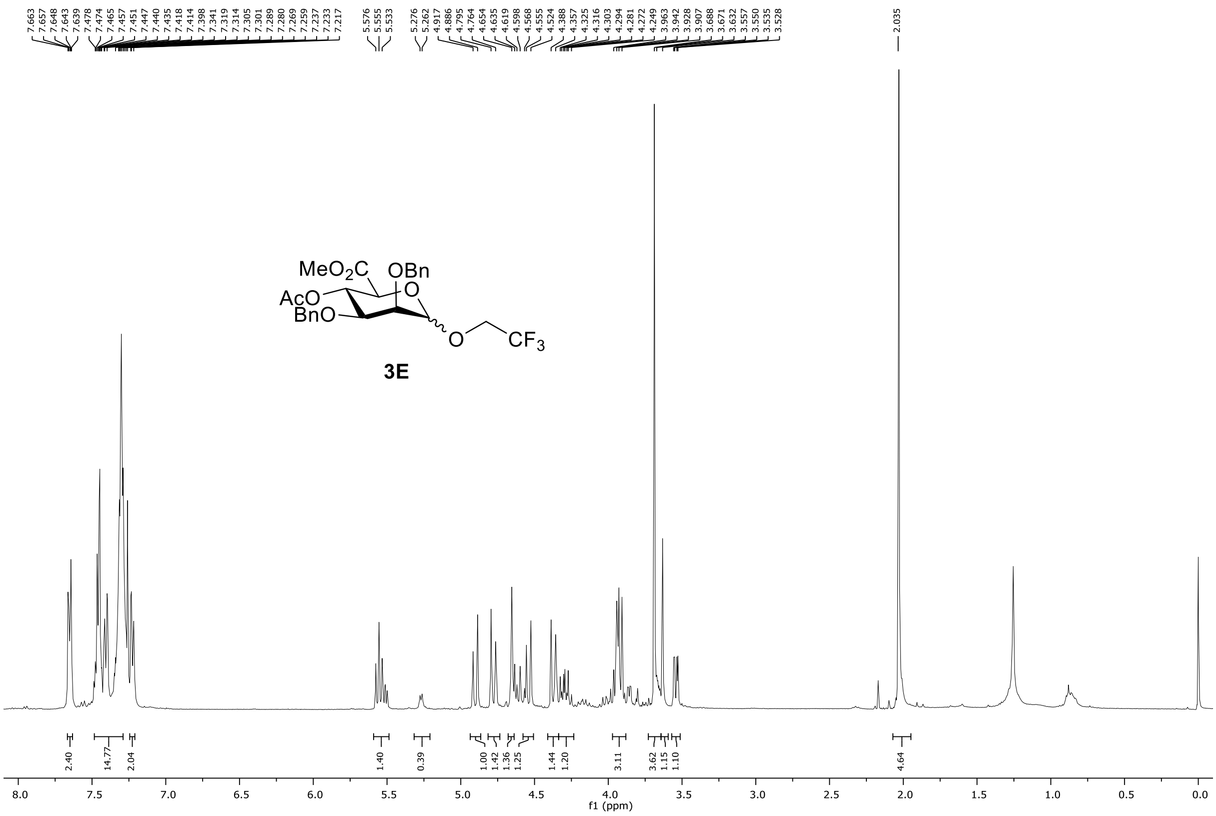

^{13}C-APT NMR, 101 MHz, $CDCl_3$ of compound **3E**

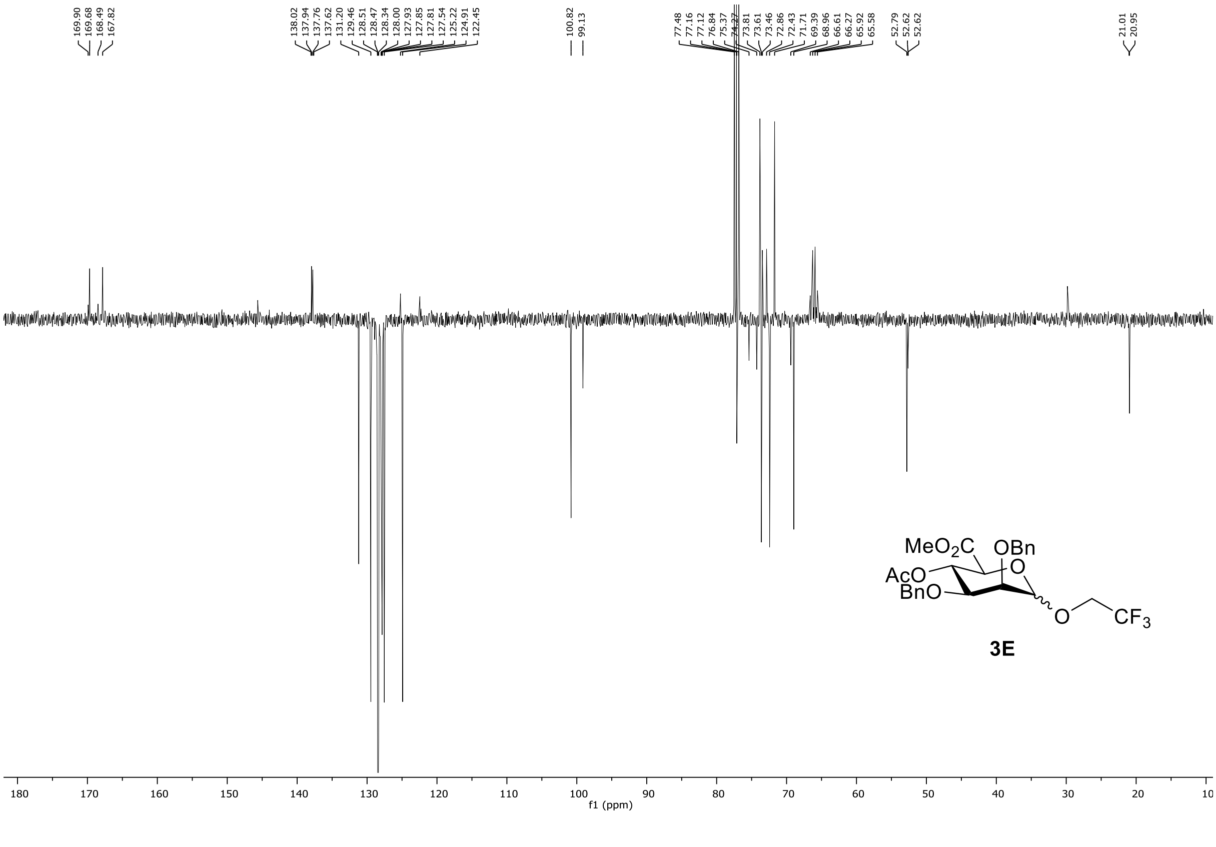

COSY NMR of compound **3E**

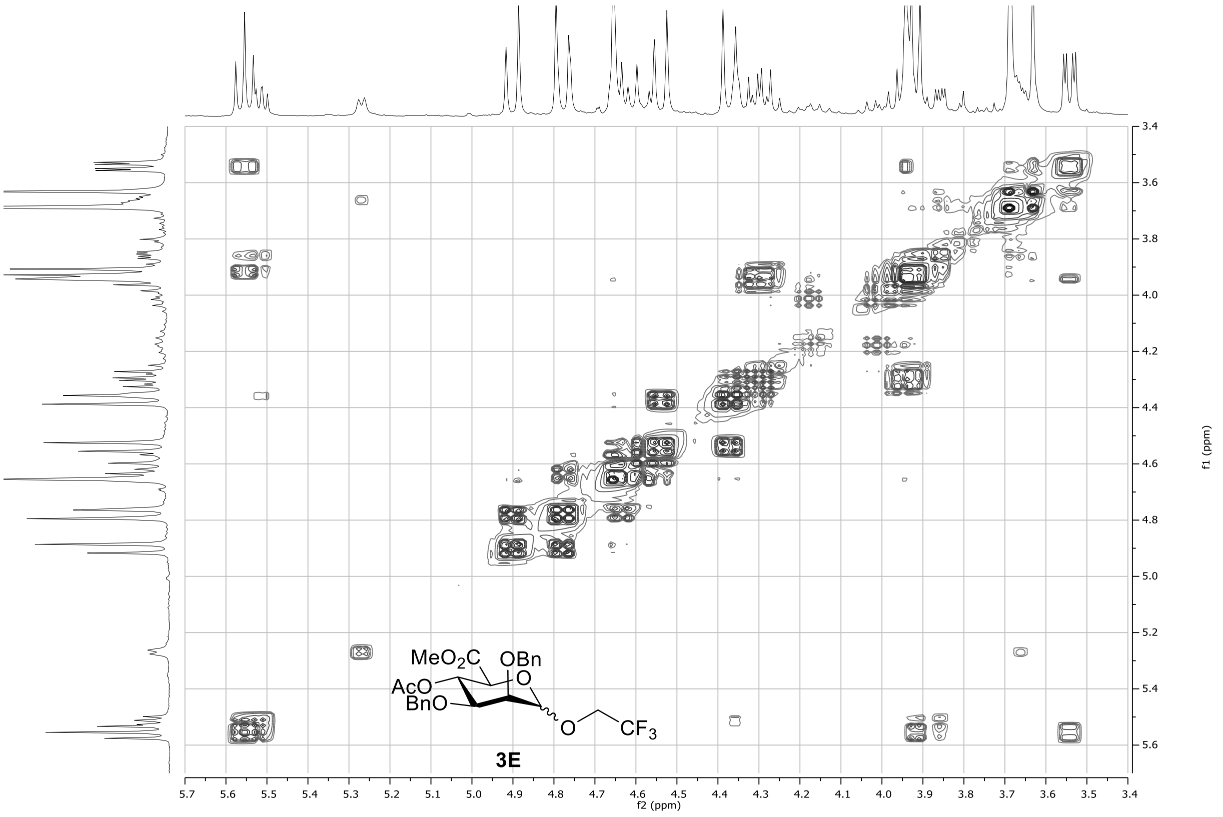

HSQC NMR of compound **3E**

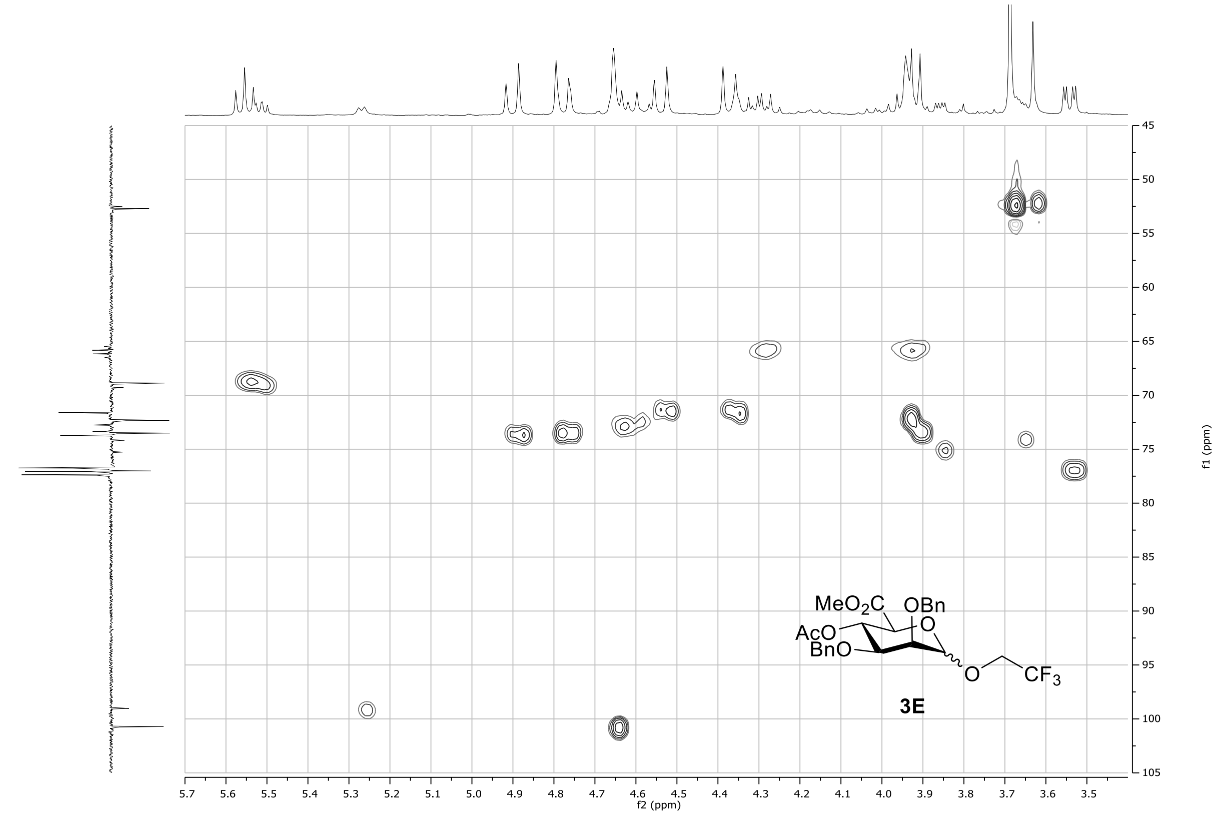

^{13}C GATED NMR, 101 MHz, $CDCl_3$ of compound **3E**

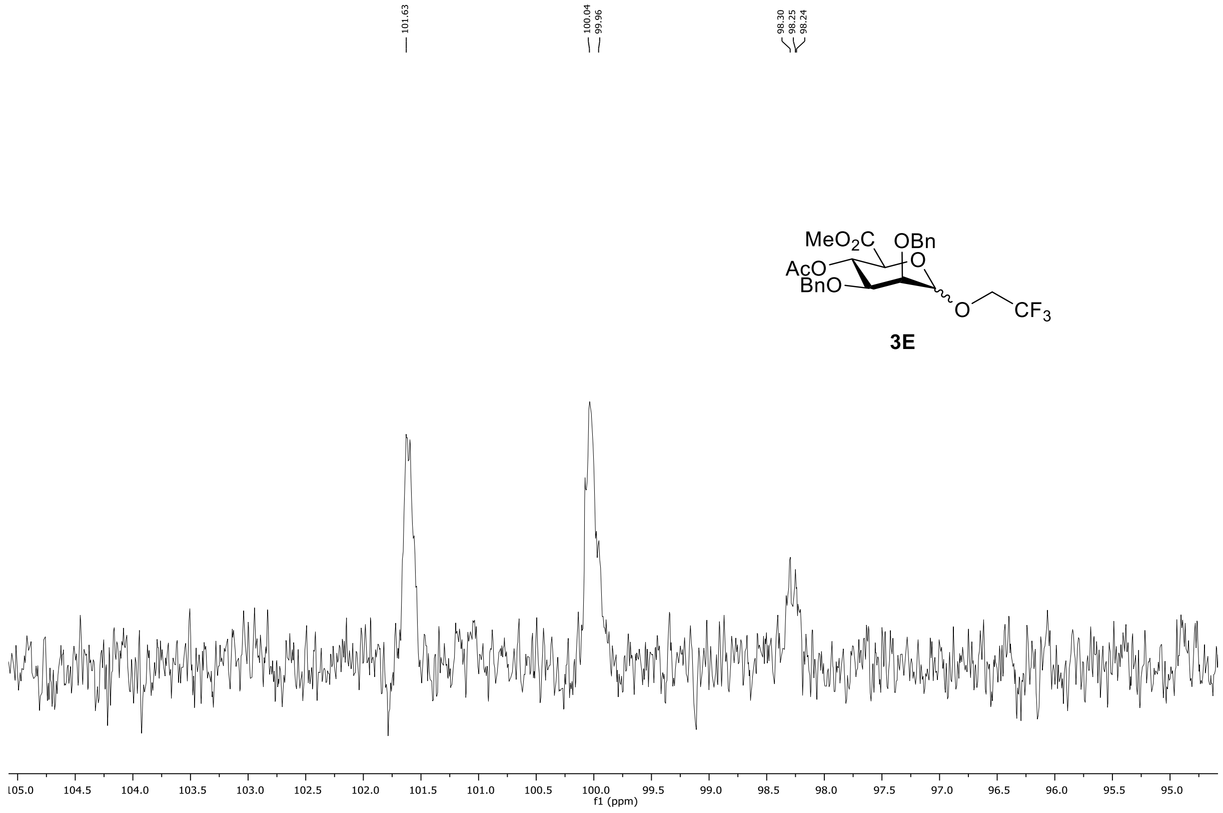

^{1}H NMR, 400 MHz, $CDCl_3$ of compound **3F**

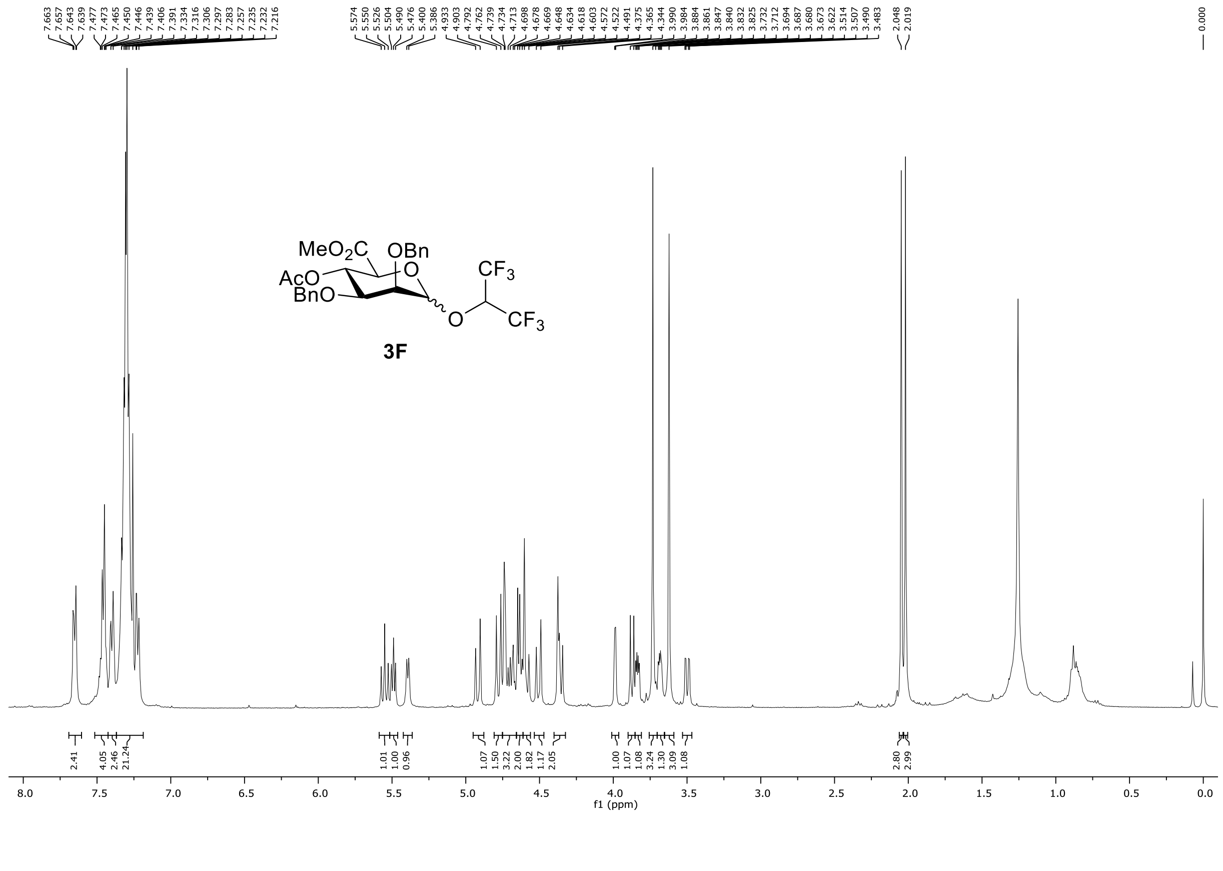

^{13}C-APT NMR, 101 MHz, $CDCl_3$ of compound **3F**

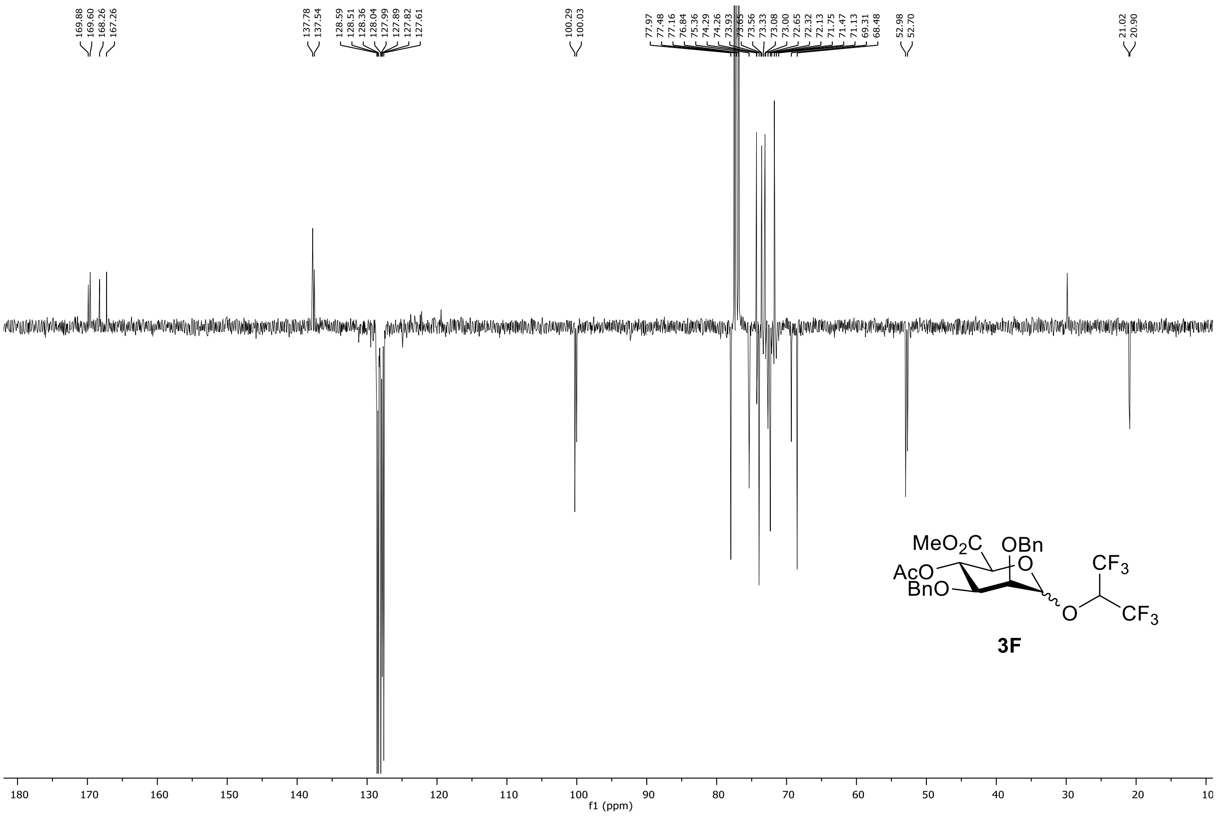

COSY NMR of compound **3F**

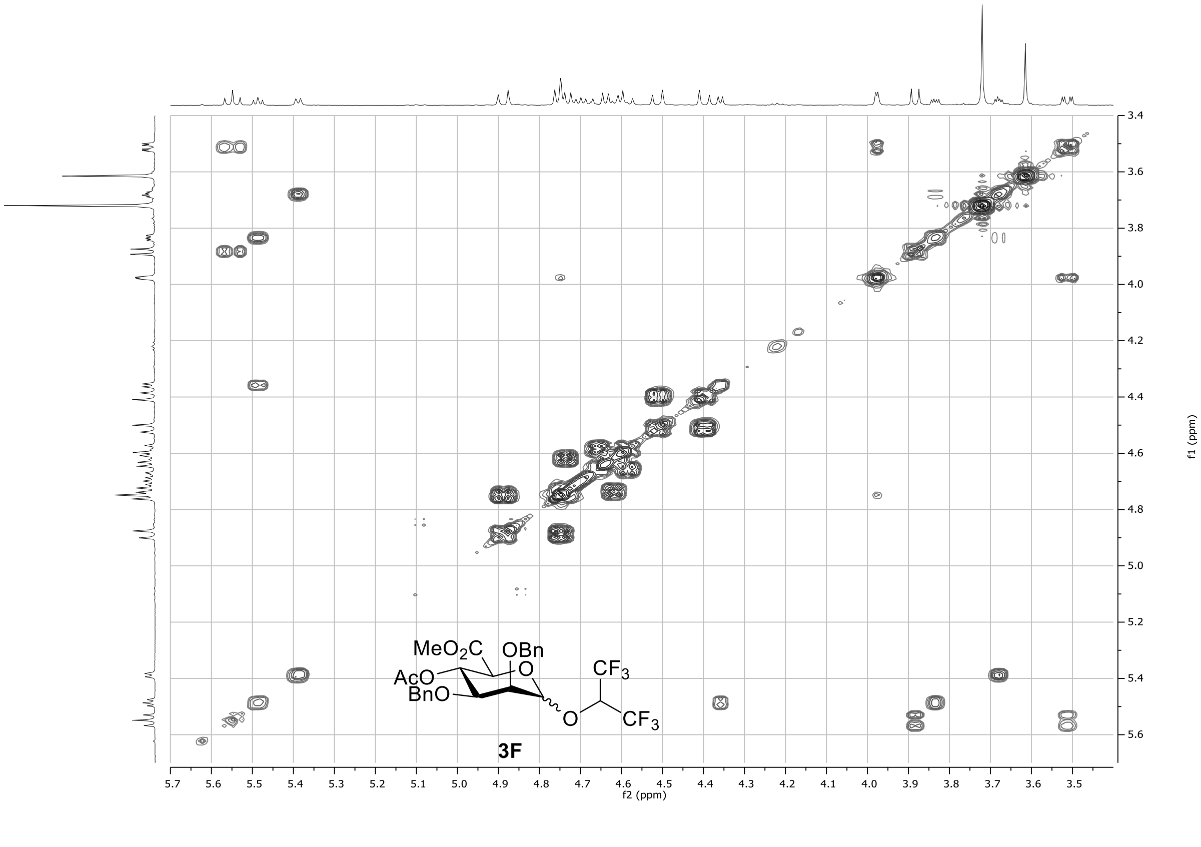

HSQC NMR of compound **3F**

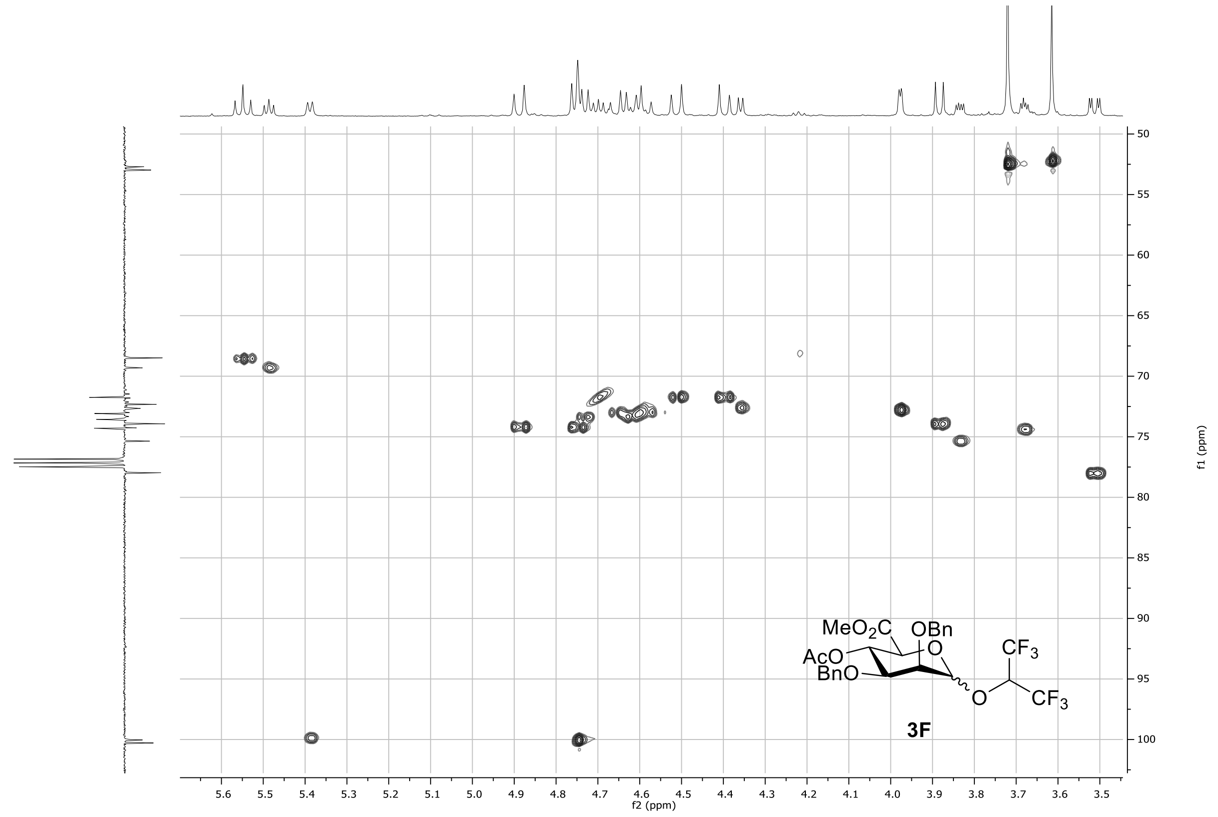

HMBC-GATED of compound **3F**

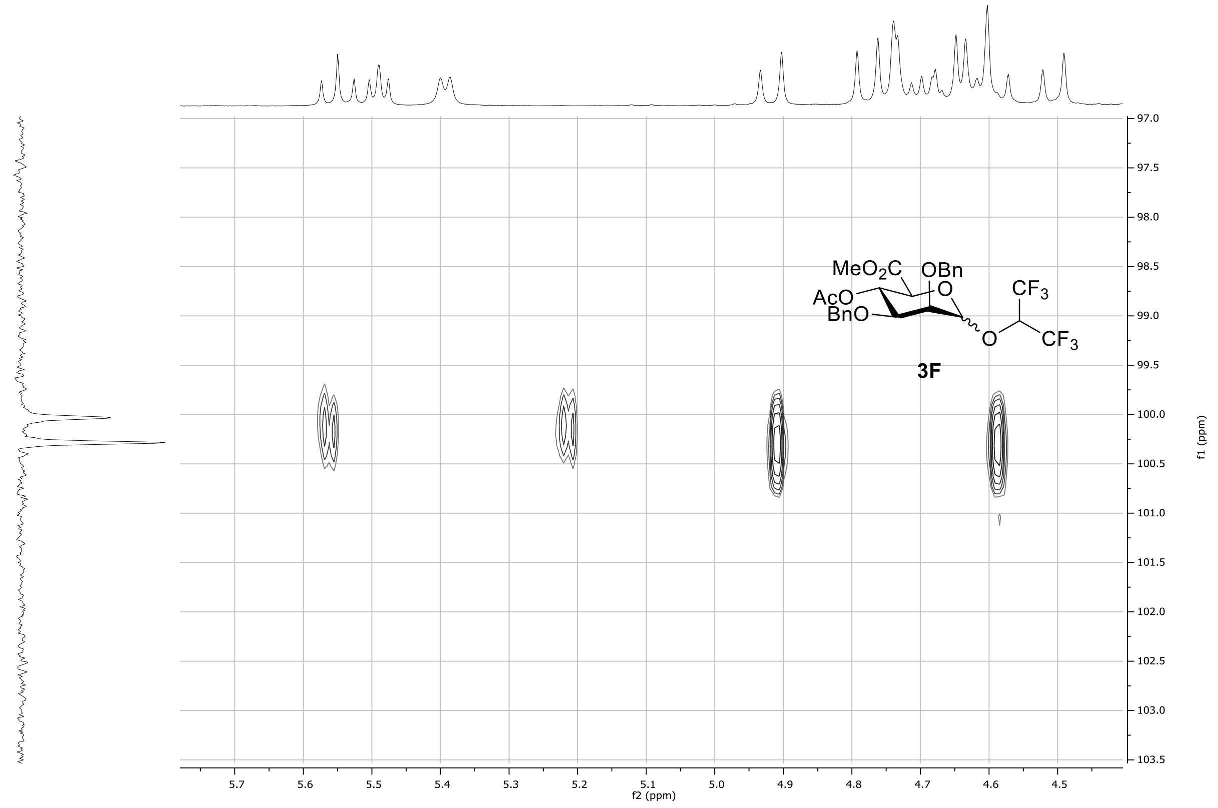

NOESY of compound **3F**

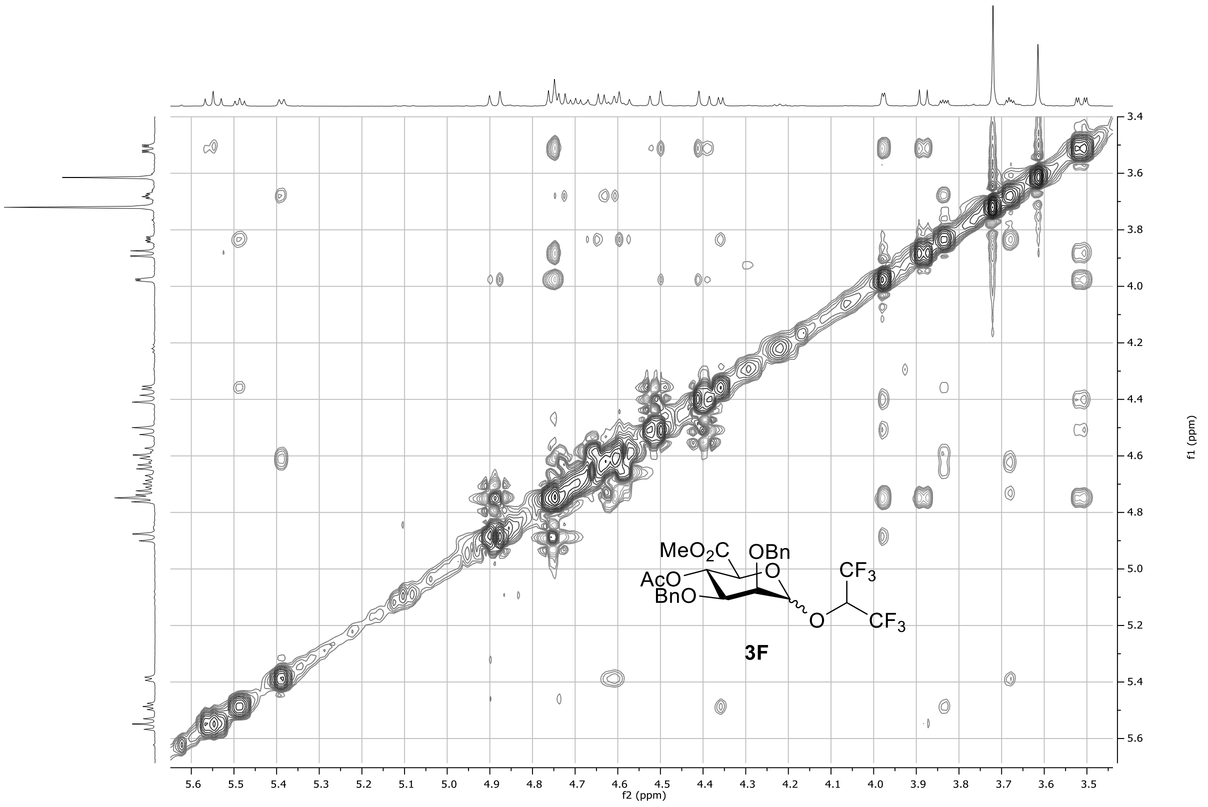

^{1}H NMR, 400 MHz, $CDCl_3$ of compound **3G**

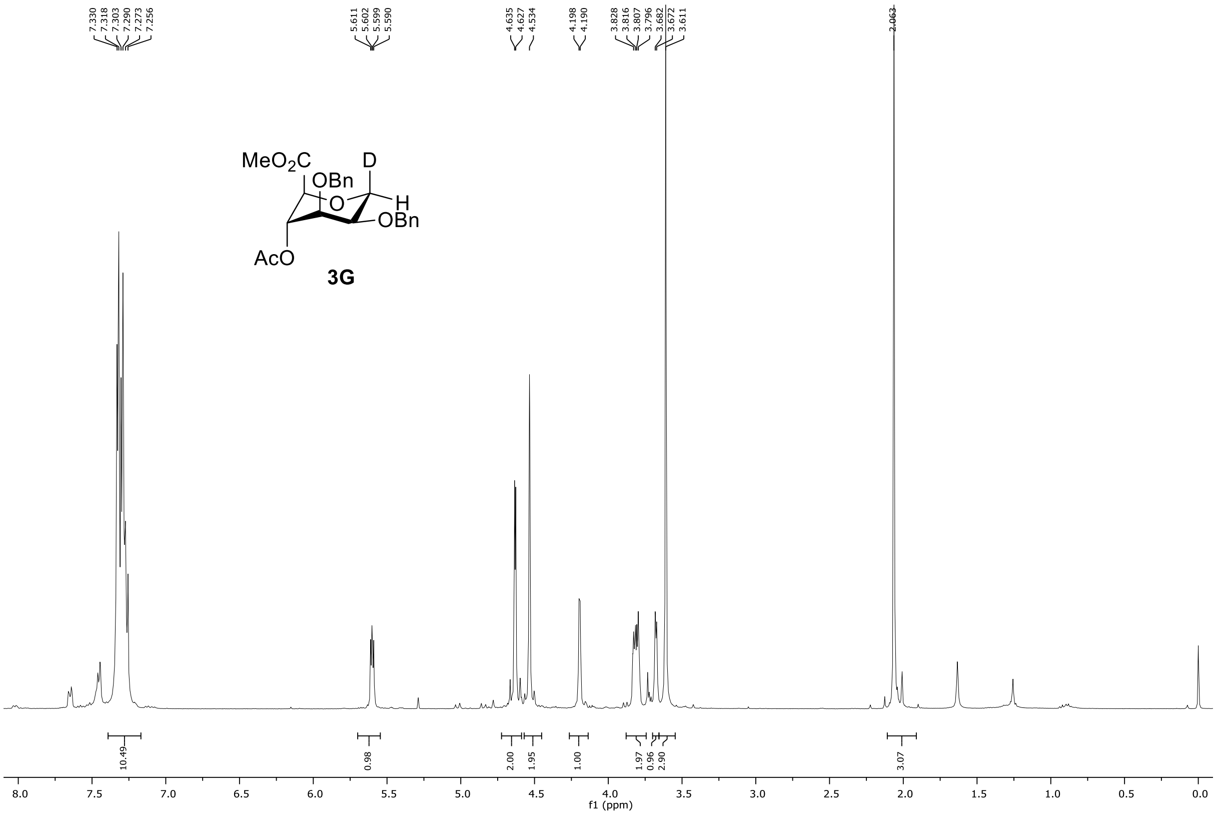

^{13}C-APT NMR, 101 MHz, $CDCl_3$ of compound **3G**

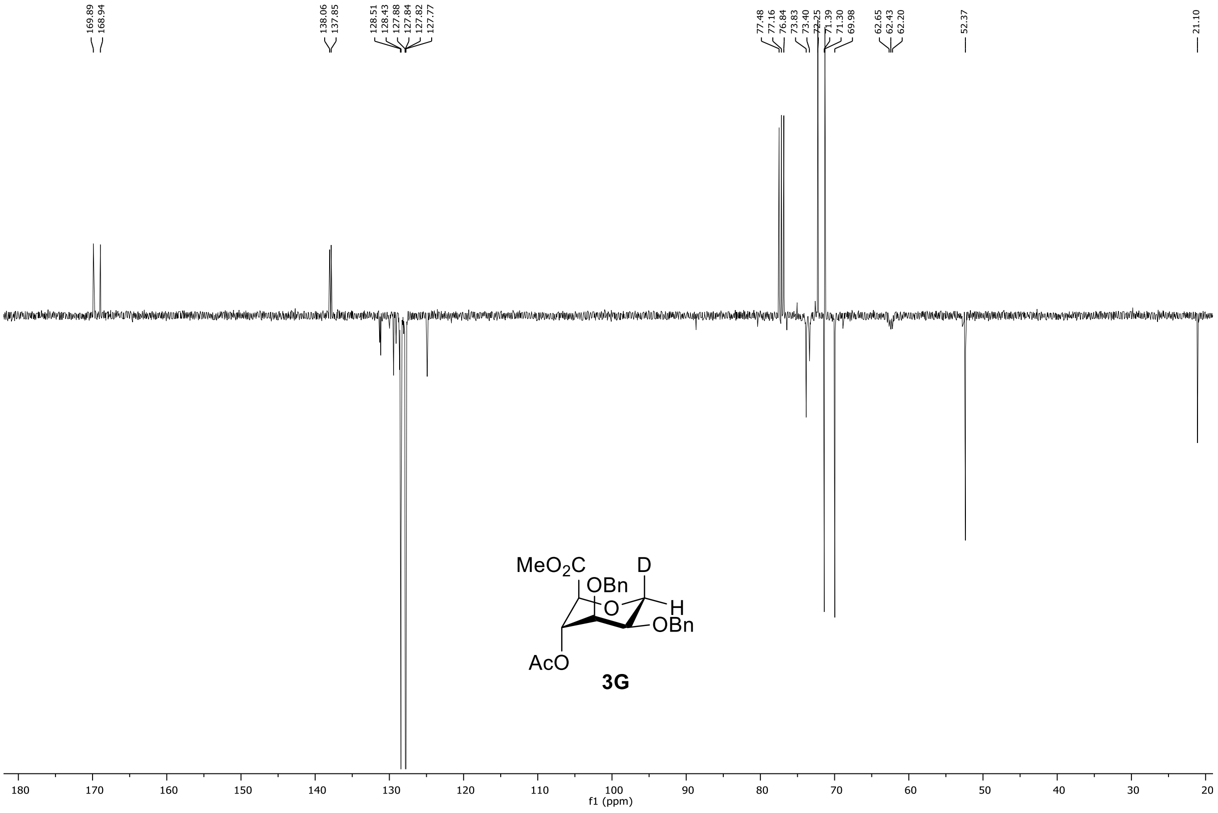

COSY NMR of compound **3G**

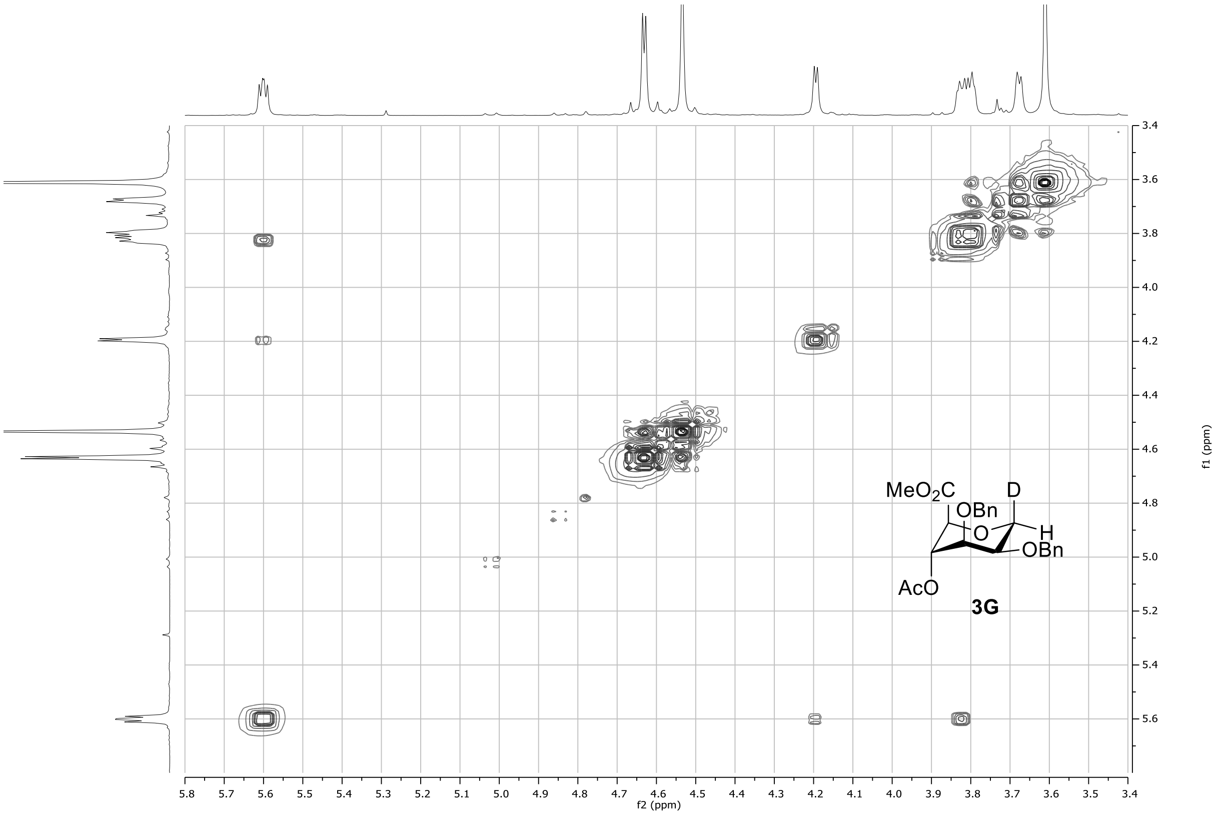

HSQC NMR of compound **3G**

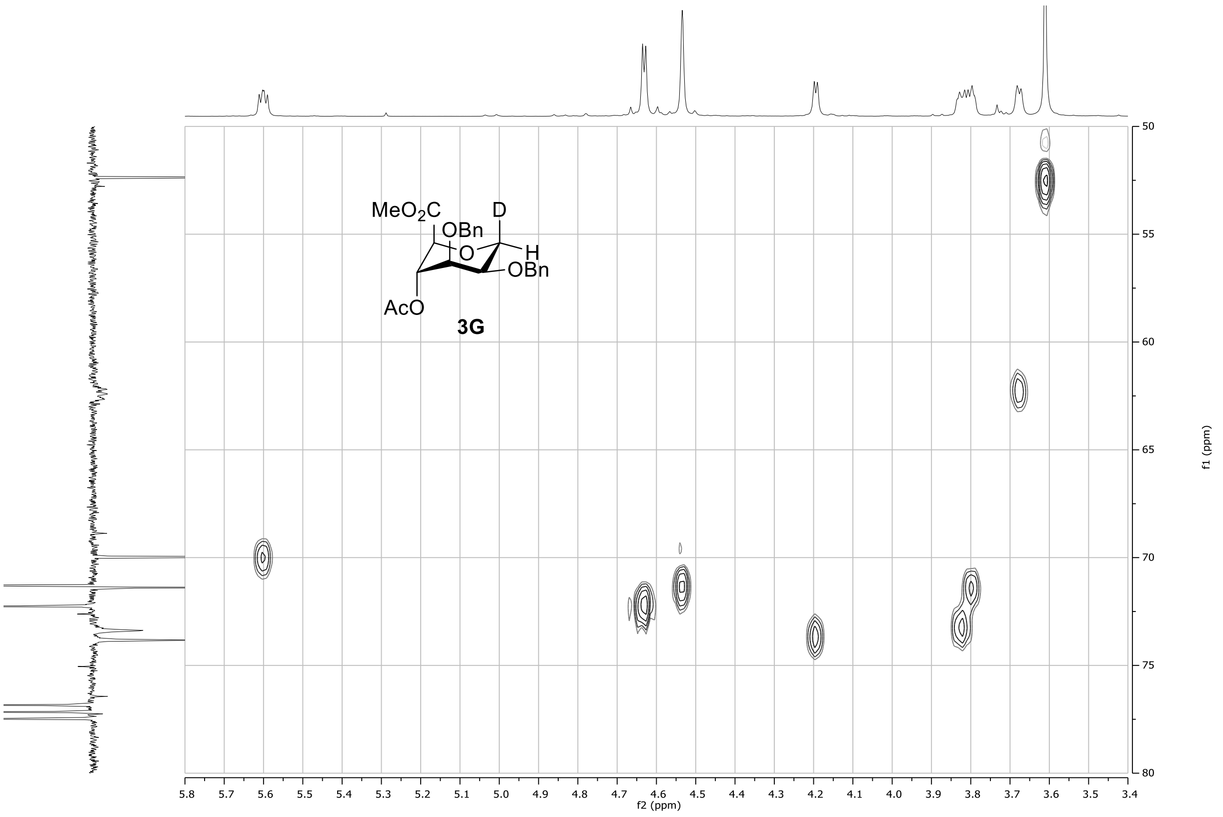

HMBC NMR of compound **3G**

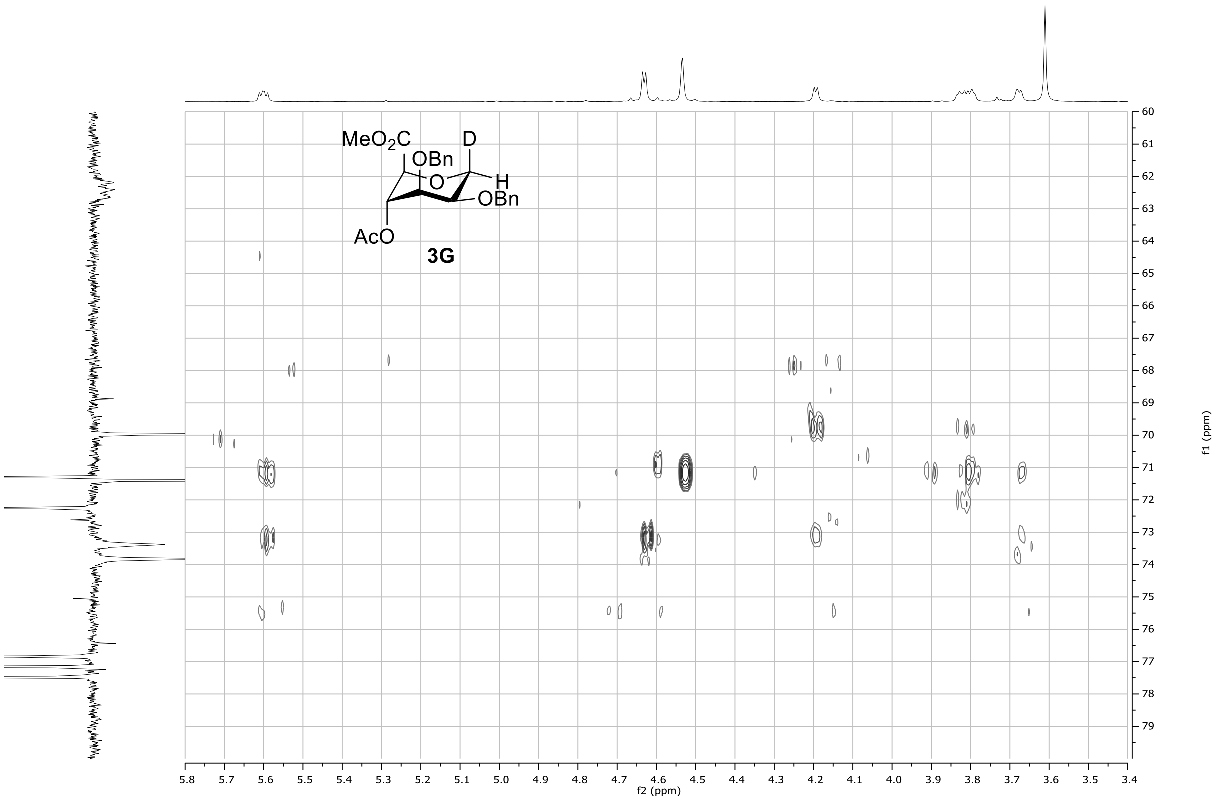

NOESY NMR of compound **3G**

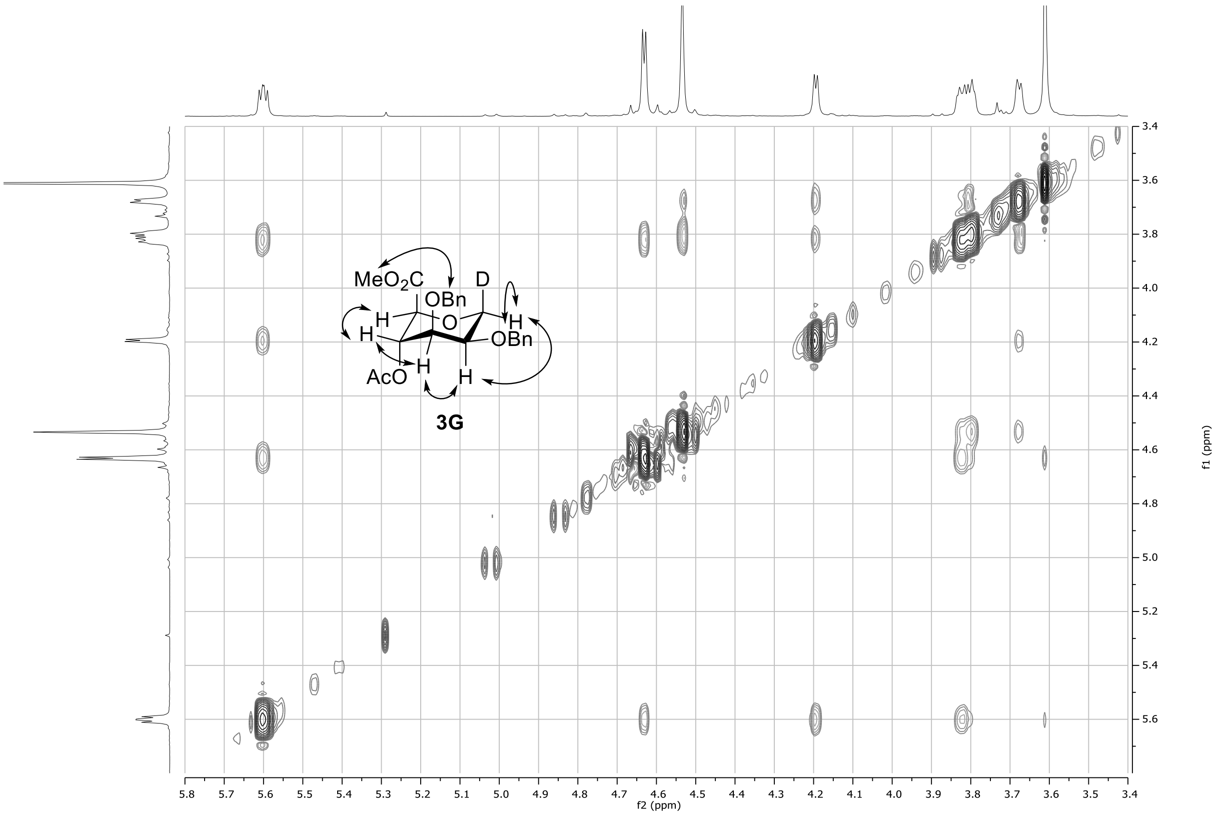

^{2}D NMR, 77 MHz, $CHCl_3$ of compound **3G**

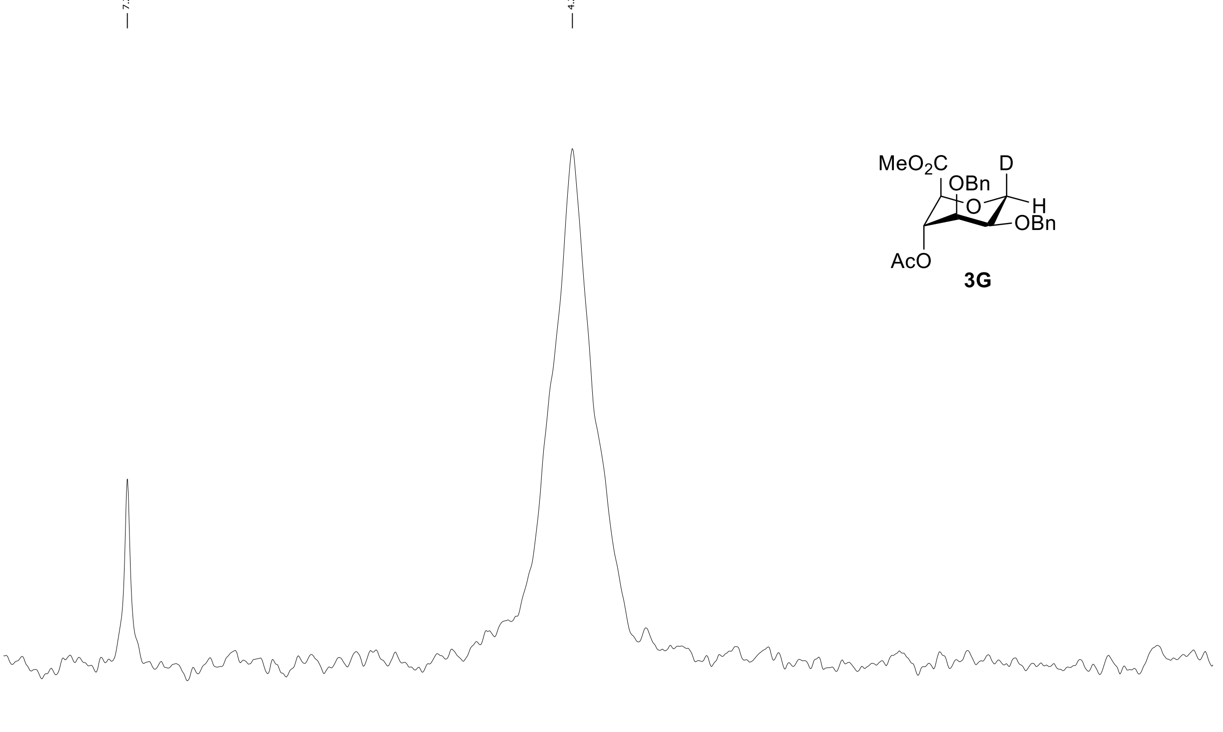

^{1}H NMR, 400 MHz, $CDCl_3$ of compound **3H**

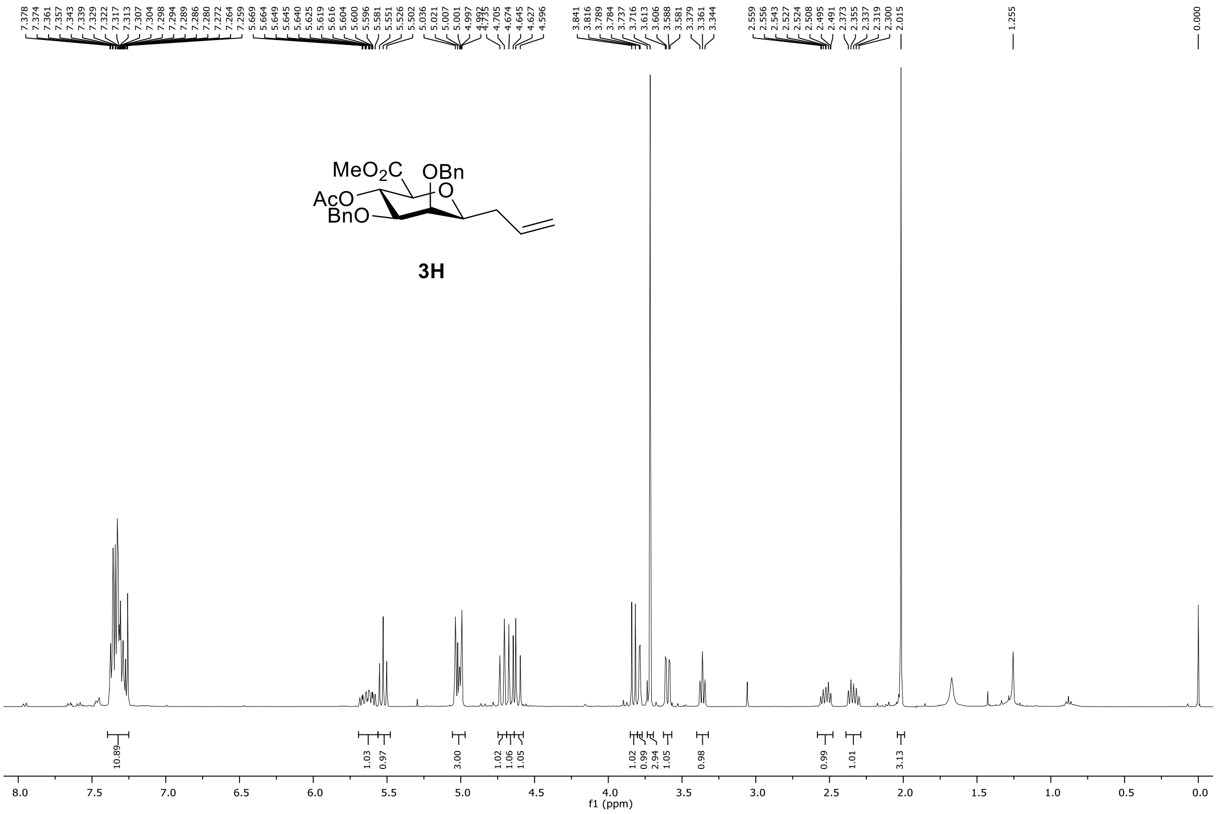

^{13}C NMR, 101 MHz, $CDCl_3$ of compound **3H**

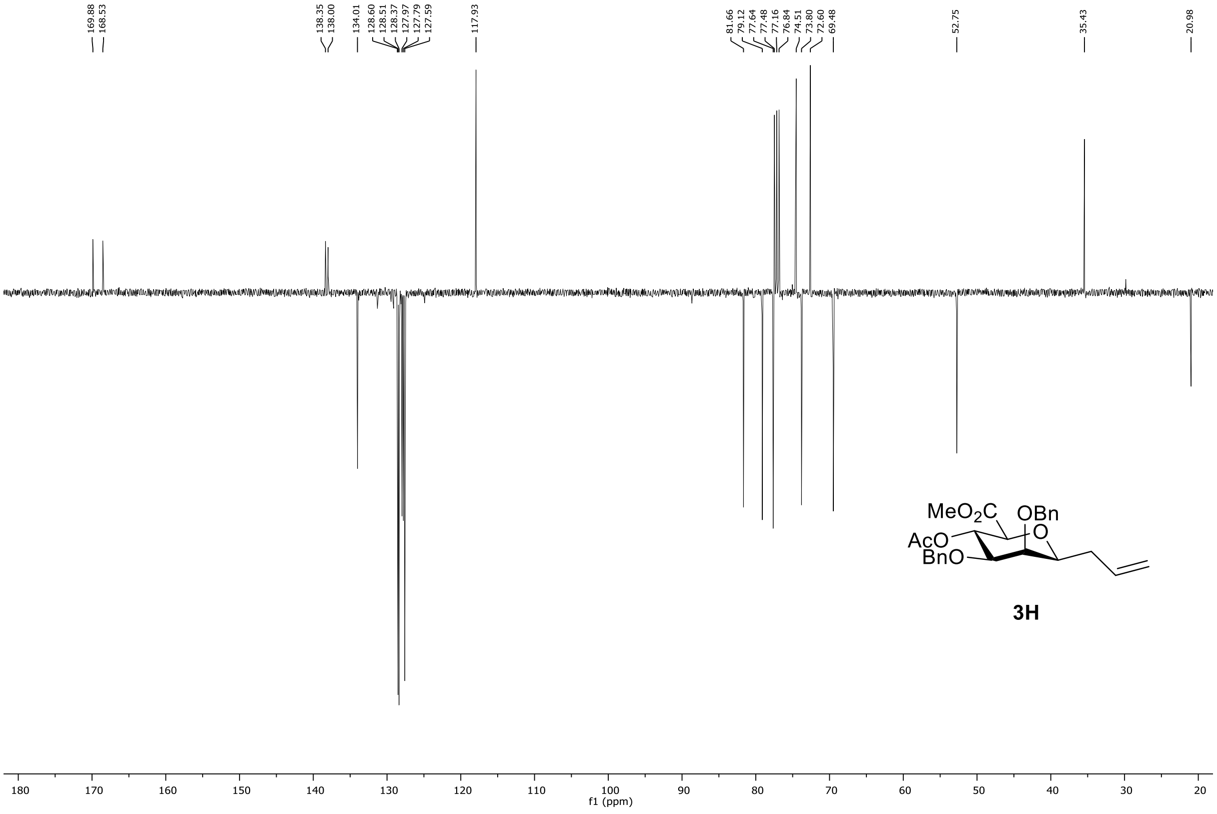

COSY NMR of compound **3H**

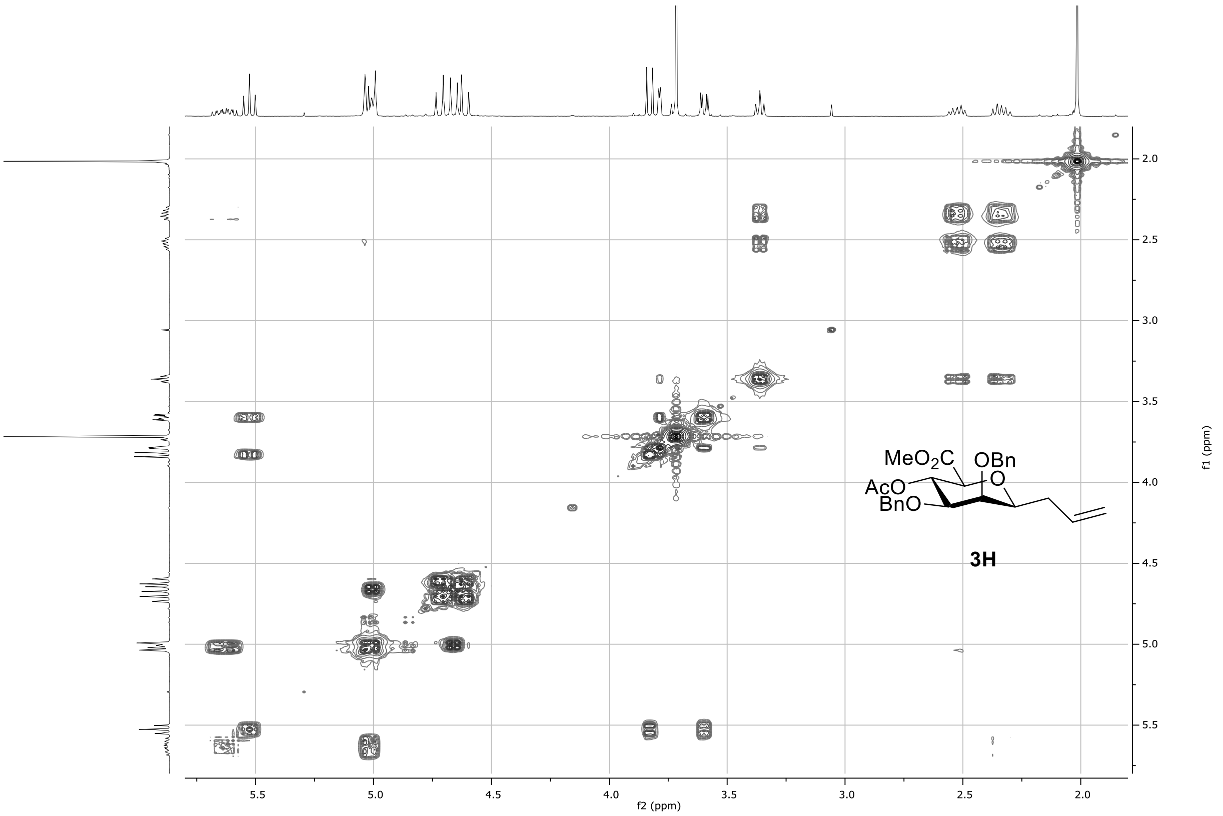

HSQC NMR of compound **3H**

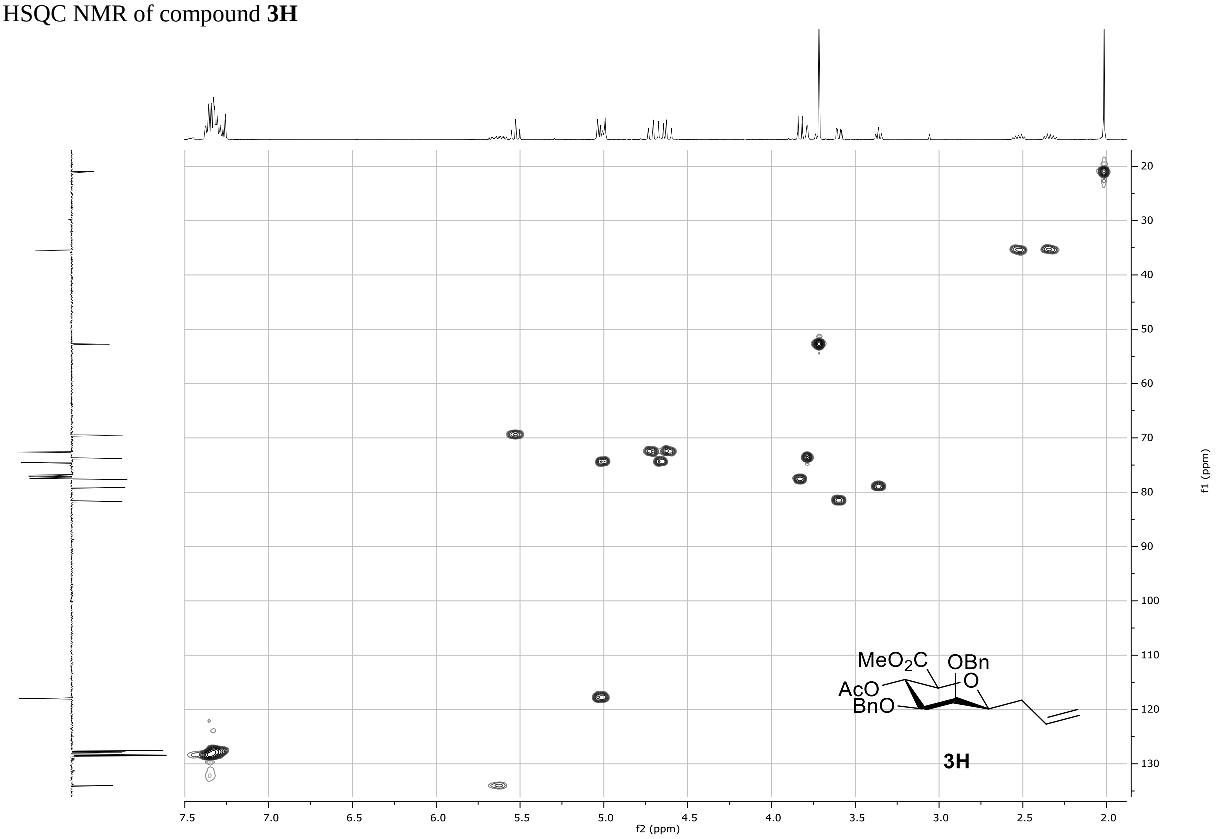

NOESY NMR of compound **3H**

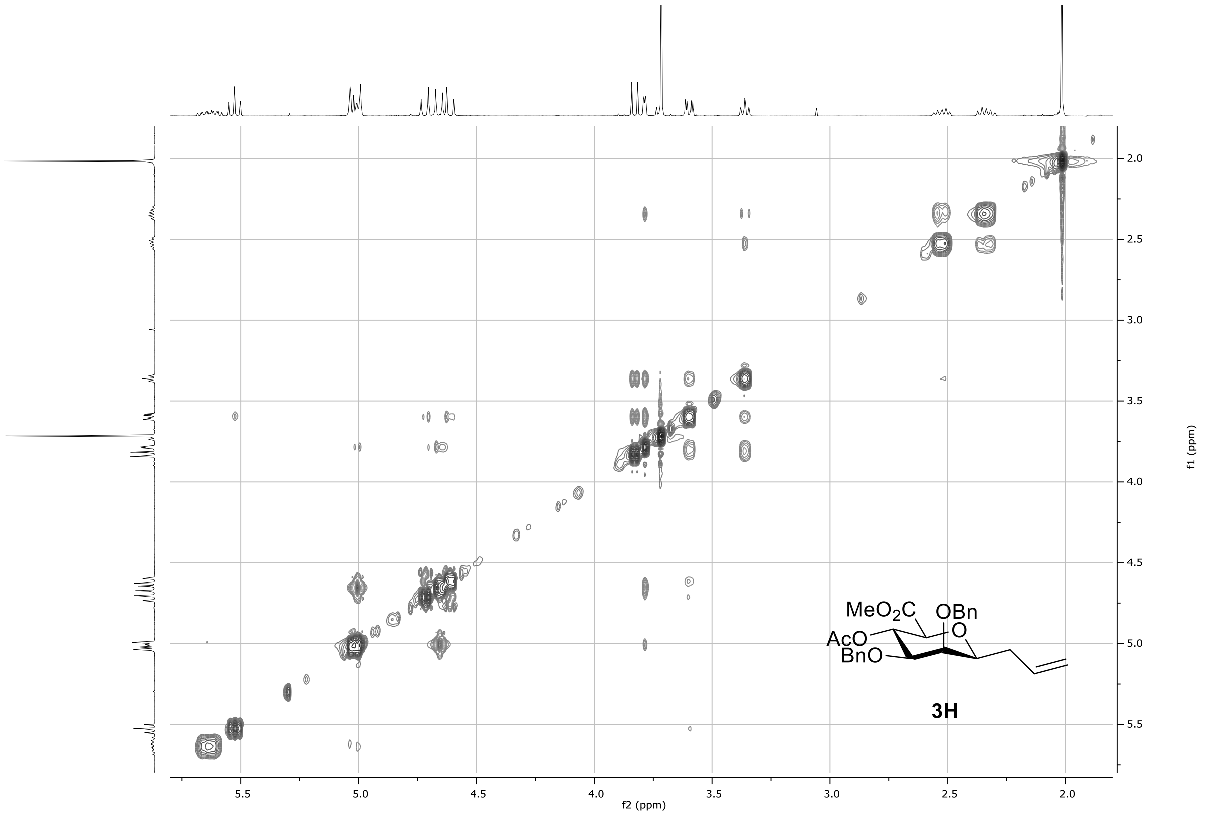

^{1}H NMR, 400 MHz, $CDCl_3$ of compound **31**

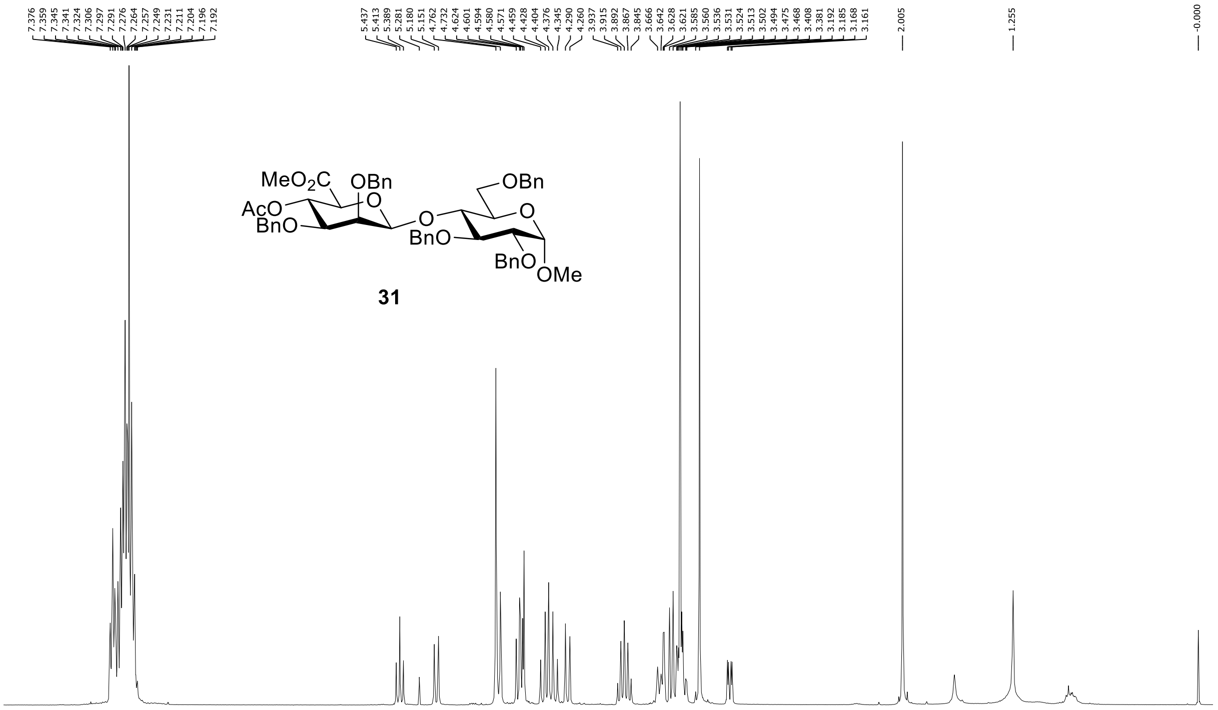

^{13}C-APT NMR, 101 MHz, $CDCl_3$ of compound **31**

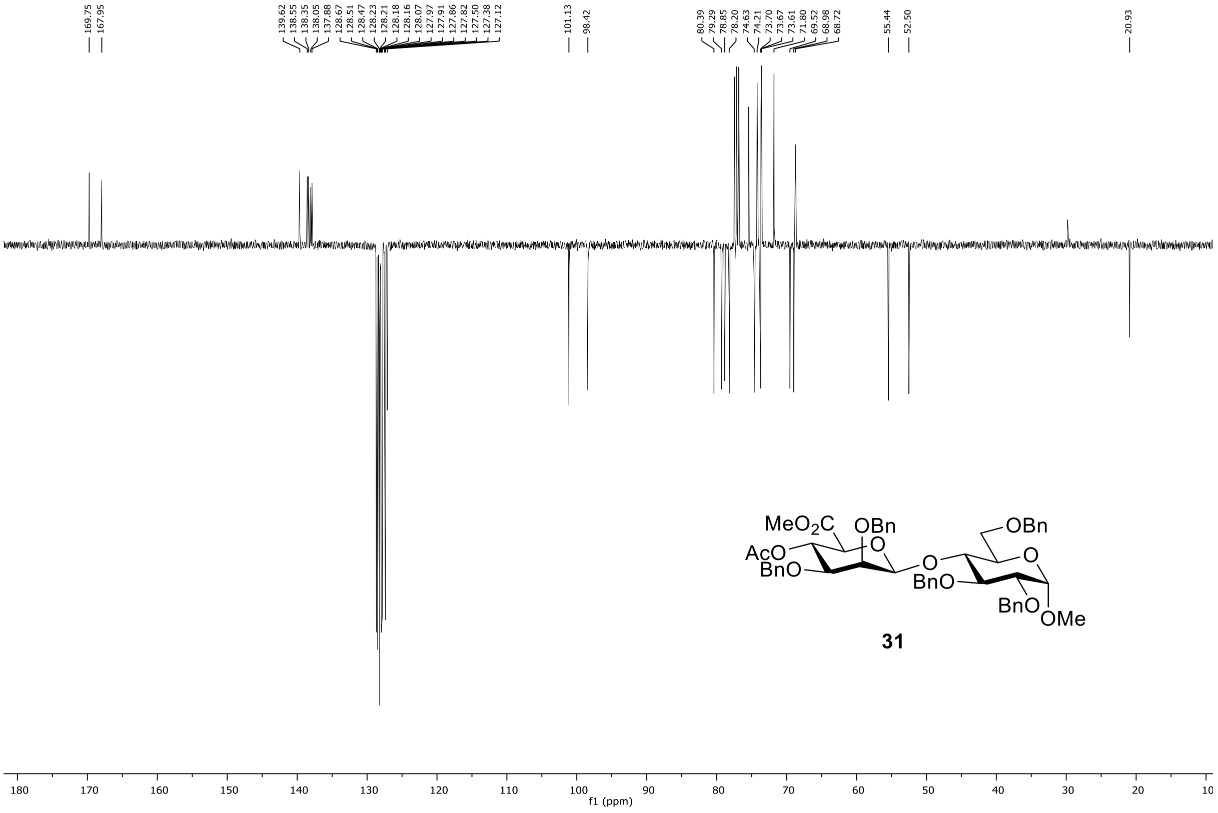

COSY NMR of compound **31**

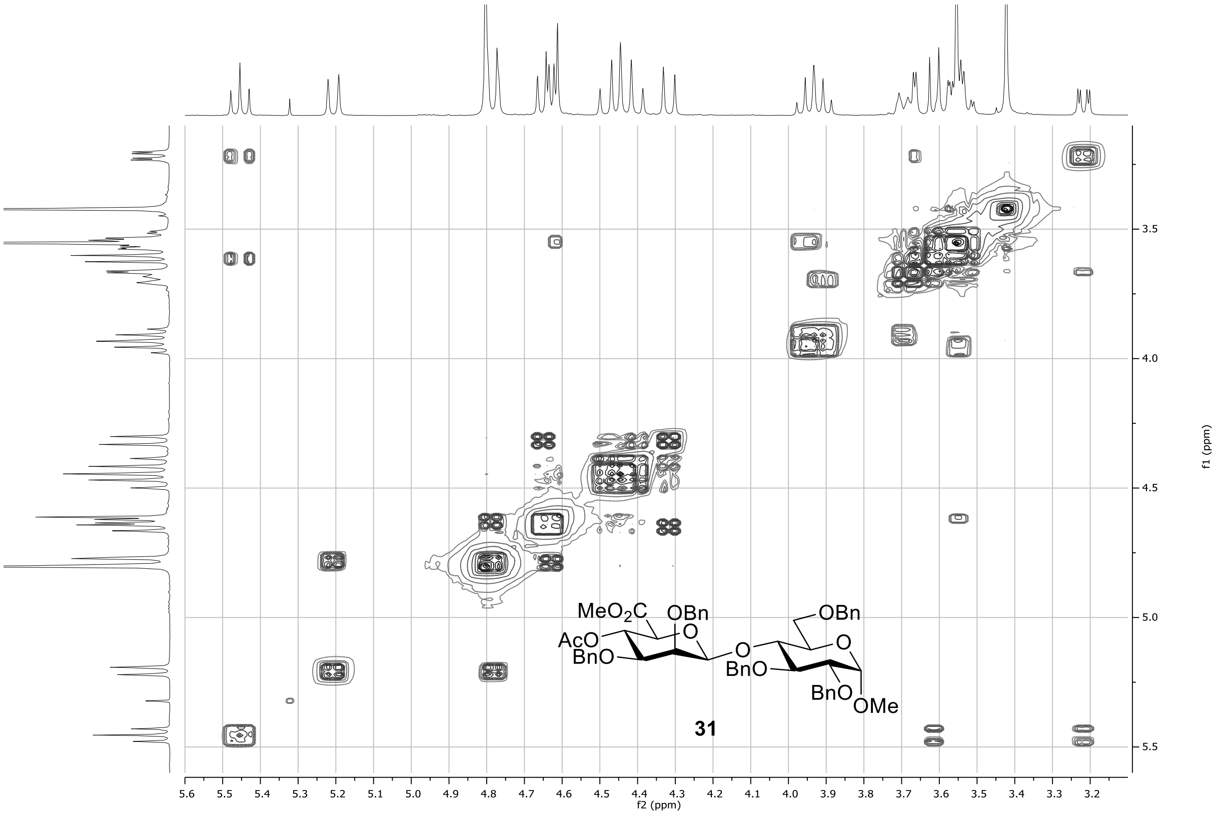

HSQC NMR of compound **31**

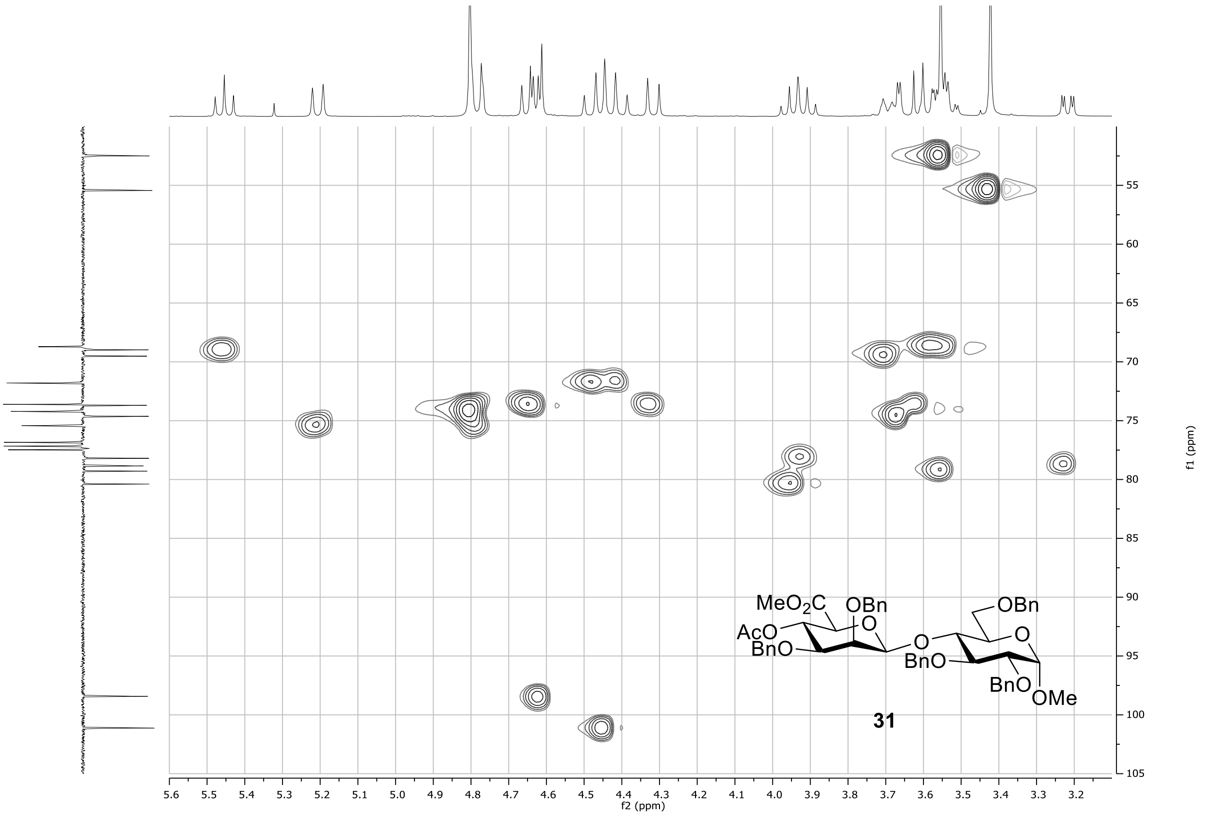

^{13}C-GATED NMR, 101 MHz, $CDCl_3$ of compound **31**

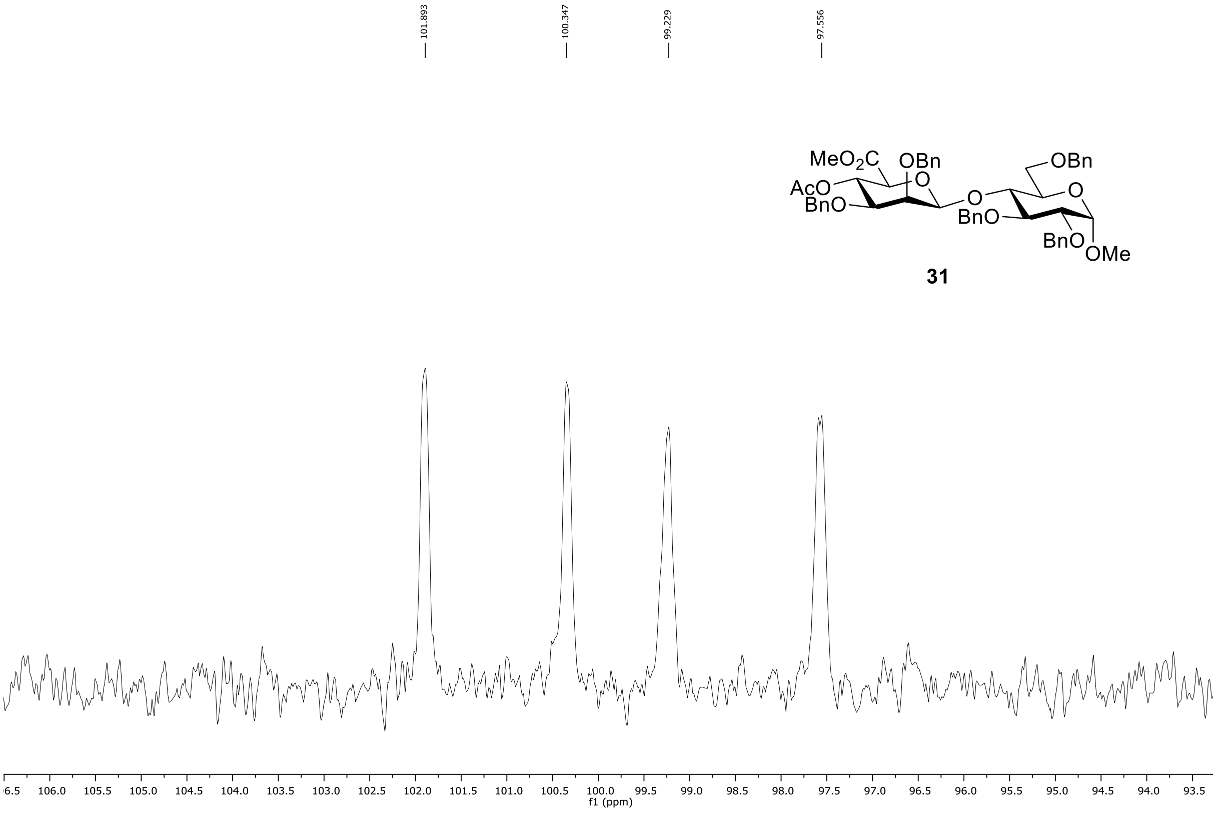

^{1}H NMR, 400 MHz, $CDCl_3$ of compound **32**

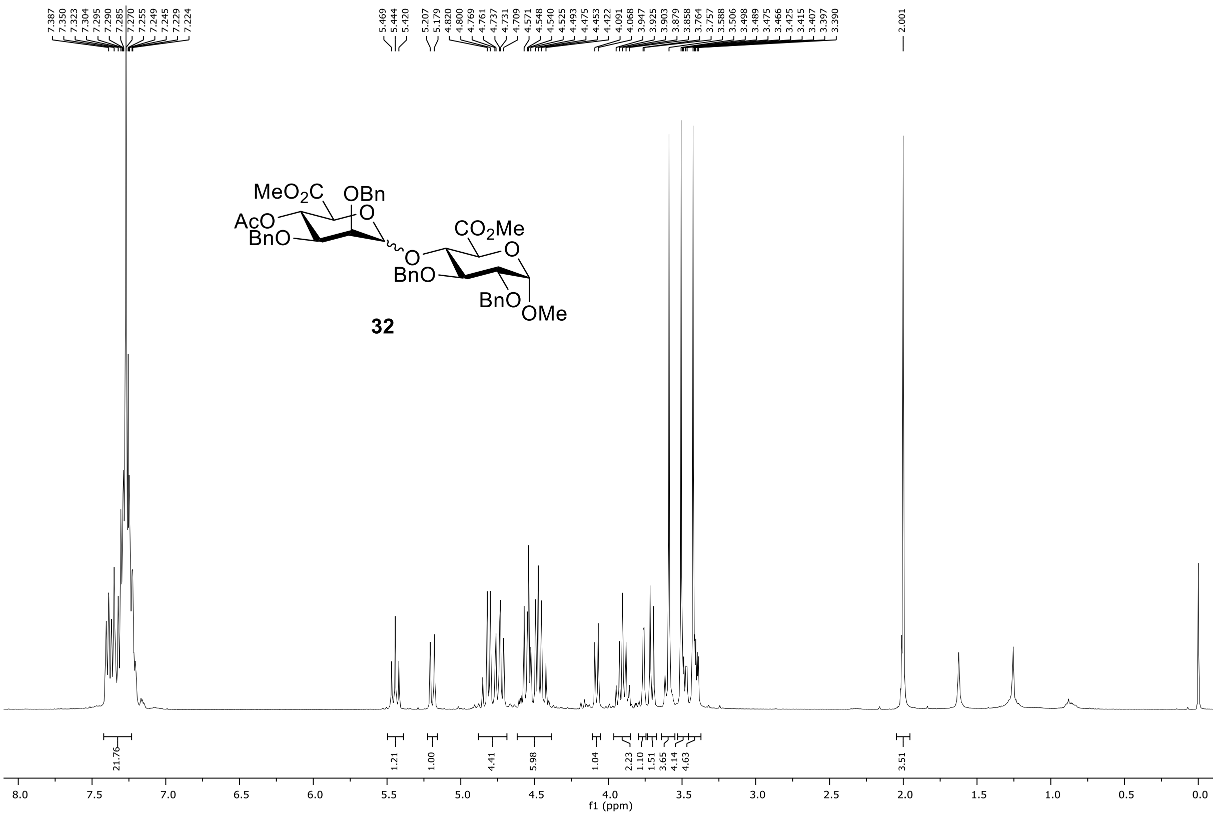

^{13}C-APT NMR, 101 MHz, $CDCl_3$ of compound **32**

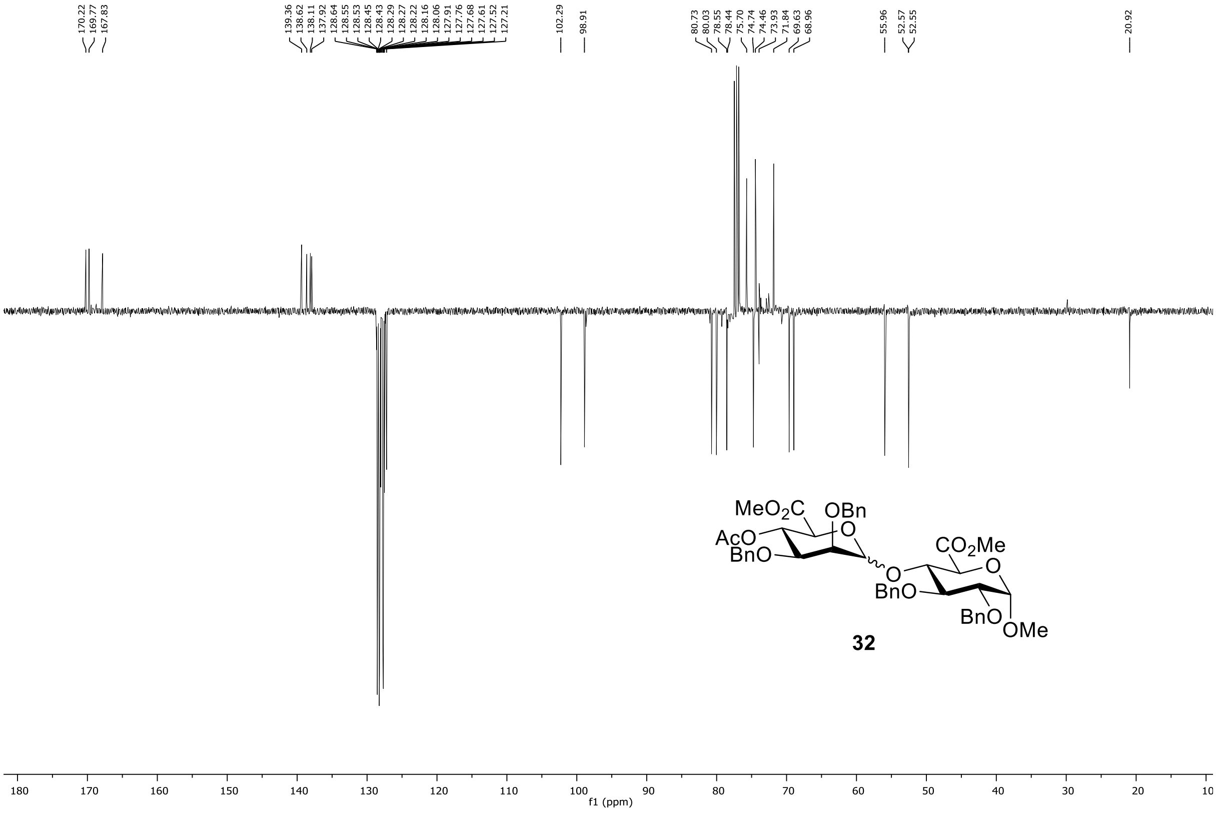

COSY NMR of compound **32**

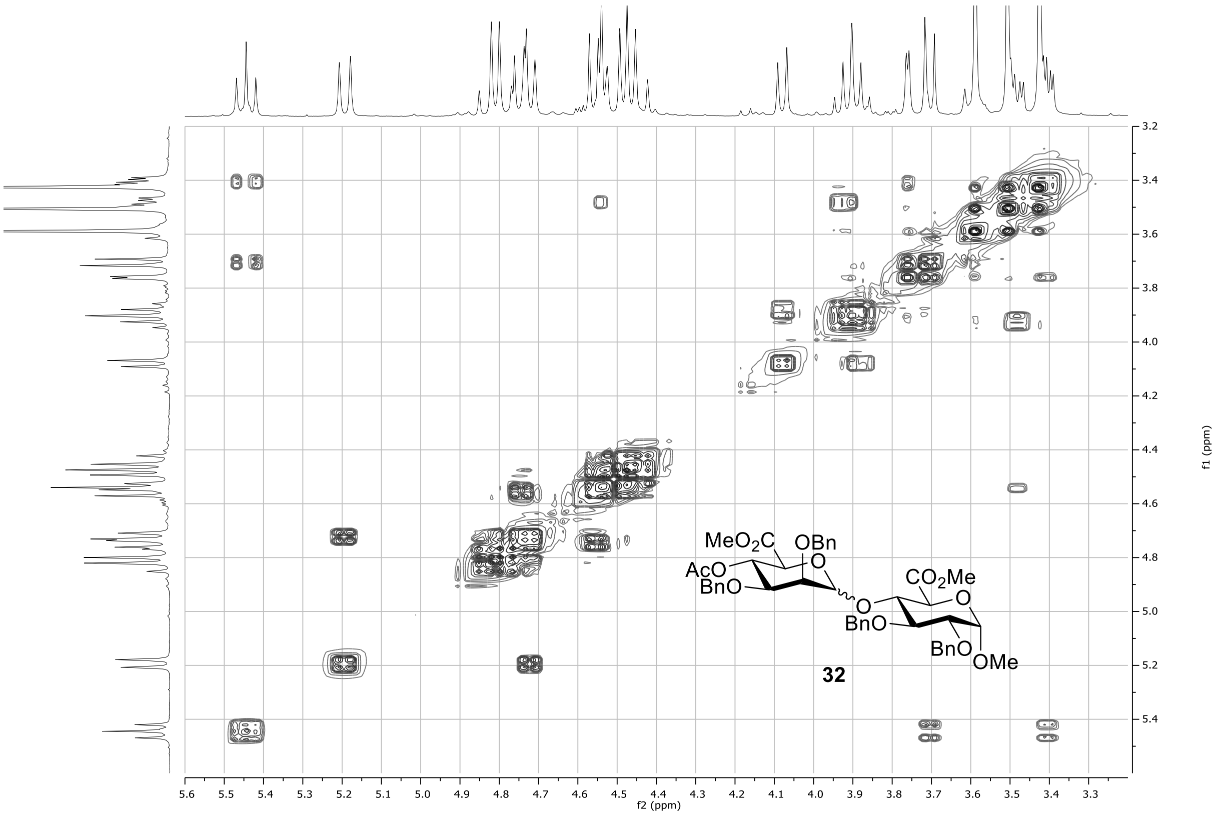

HSQC NMR of compound **32**

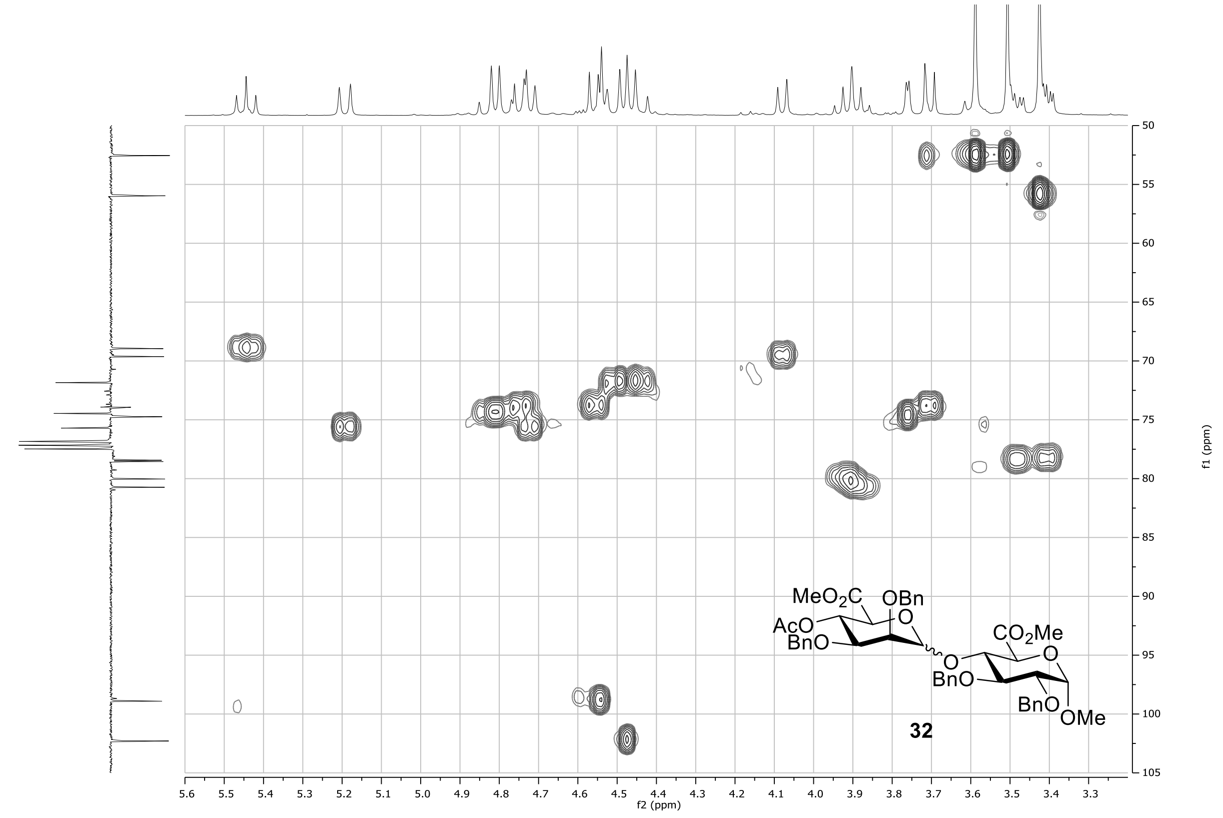

HMBC NMR of compound **32**

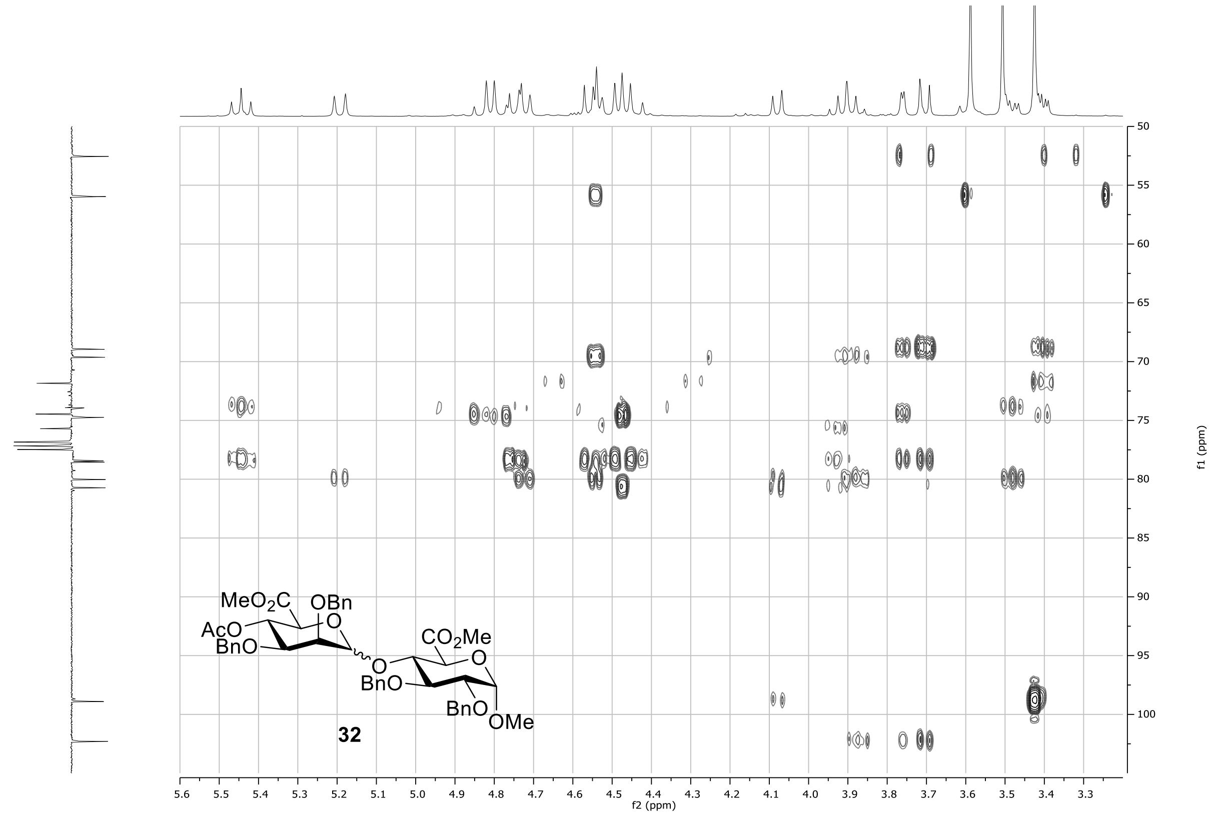

^{1}H NMR, 400 MHz, $CDCl_3$ of compound **33**

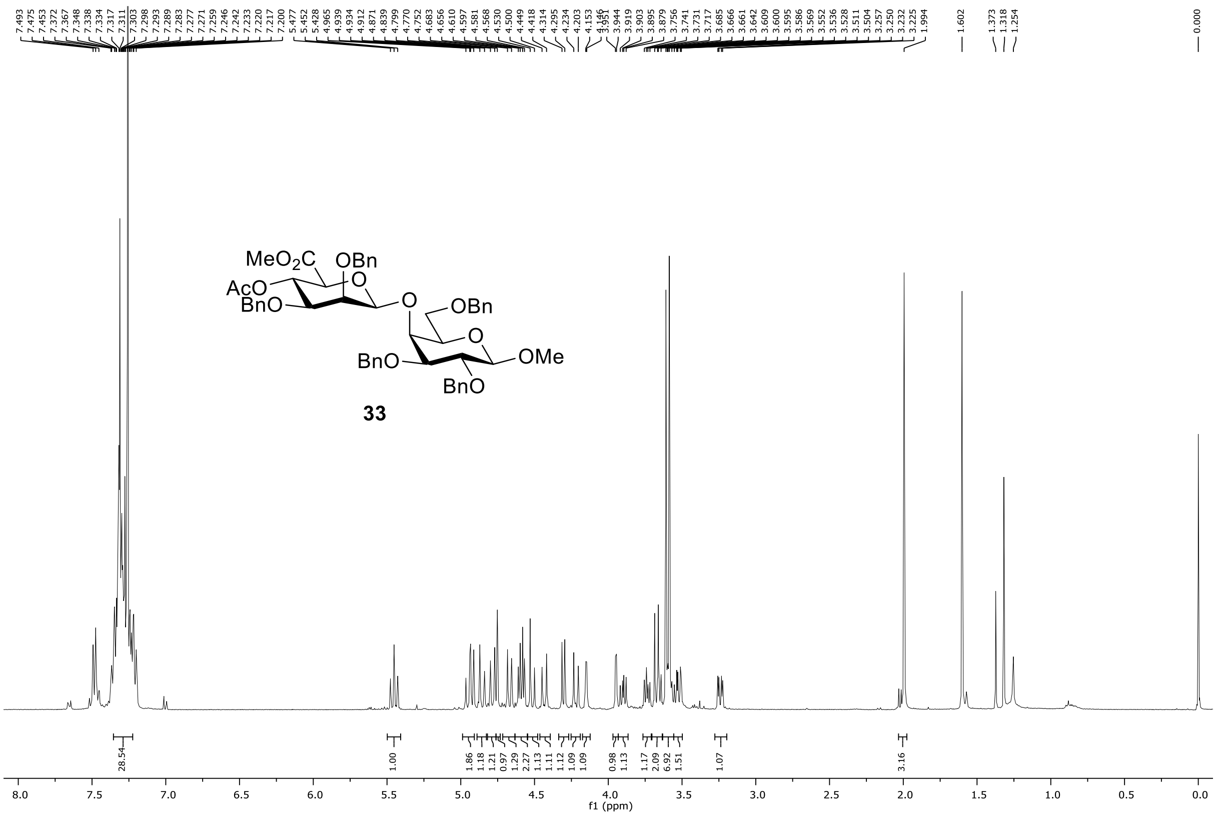

^{13}C-APT NMR, 101 MHz, $CDCl_3$ of compound **33**

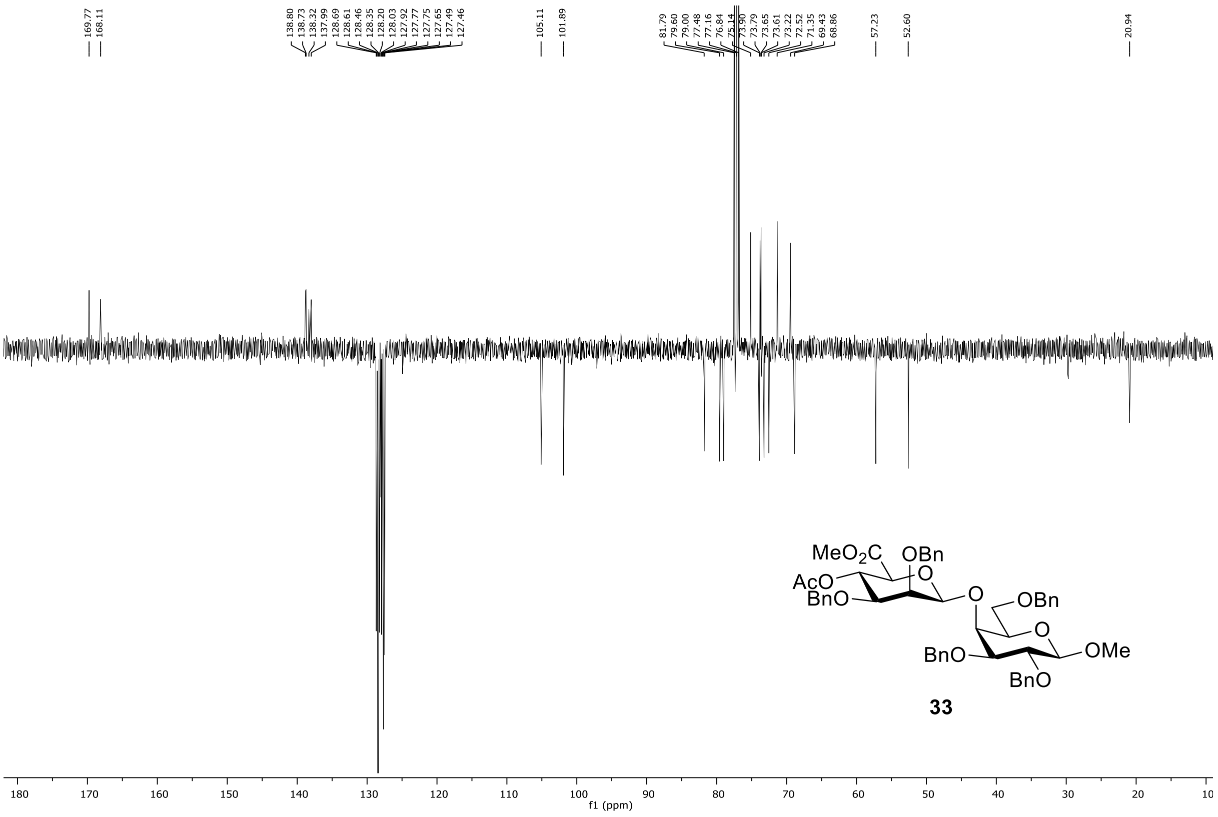

COSY NMR of compound **33**

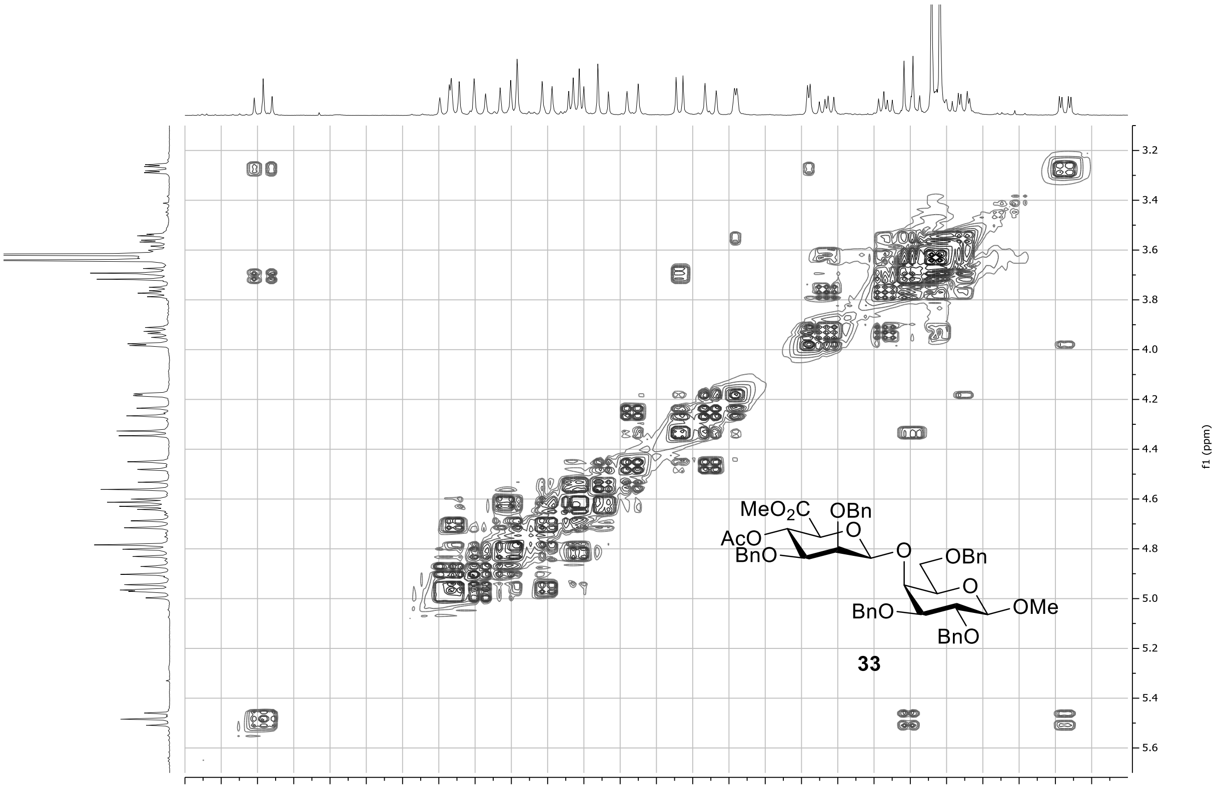

HSQC NMR of compound **33**

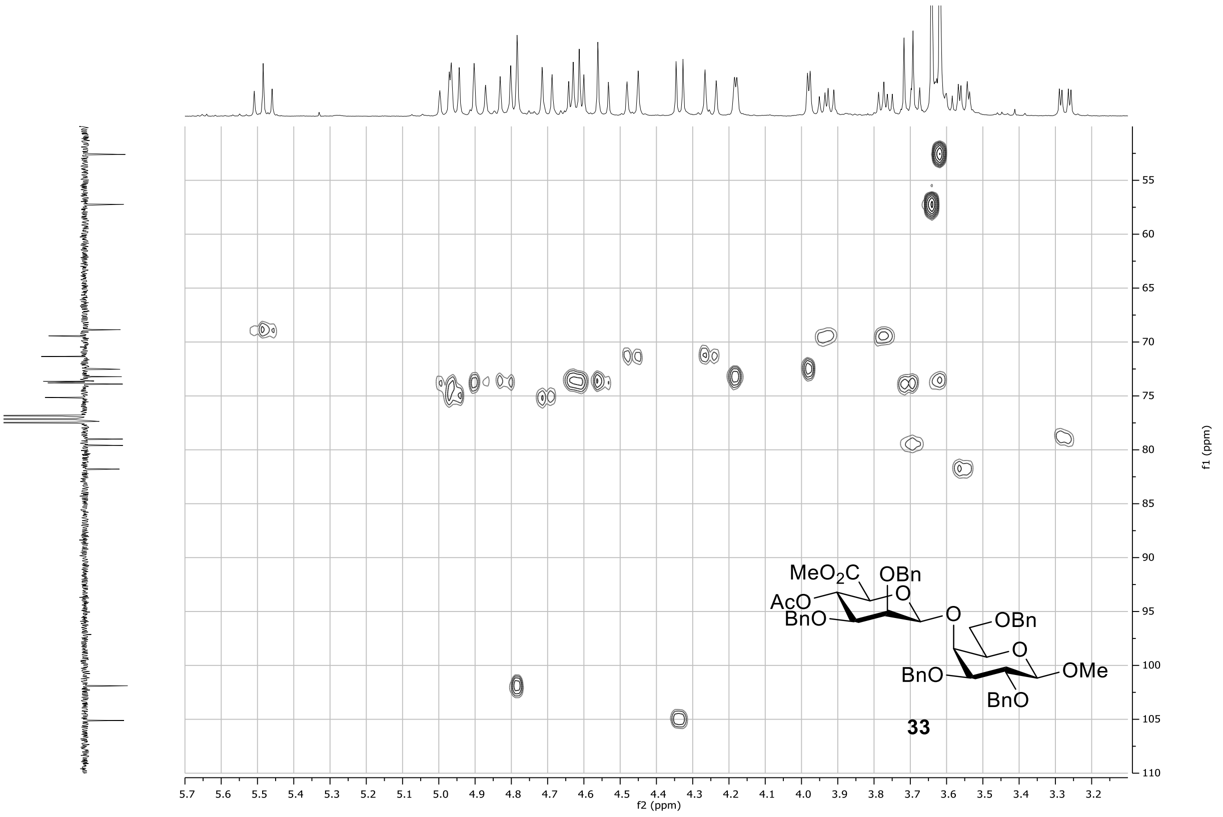

HMBC NMR of compound **33**

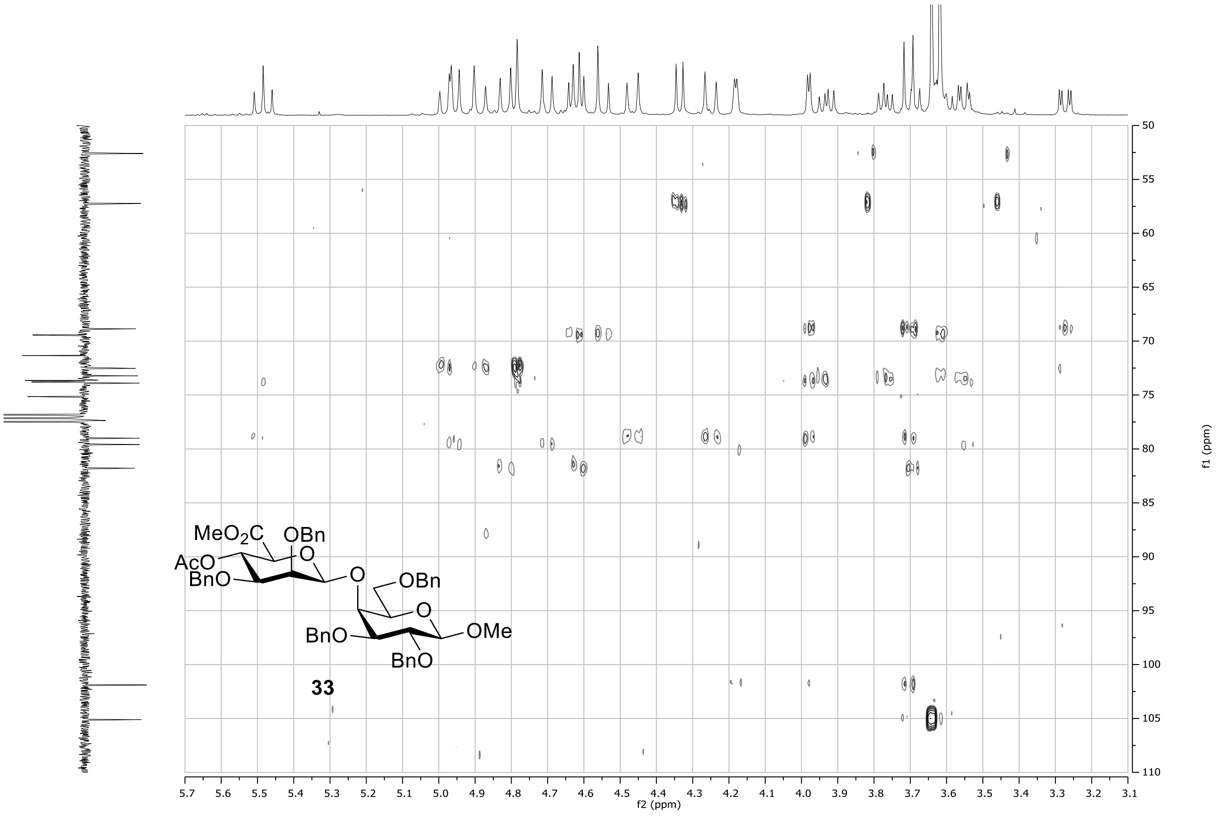

^{1}H NMR, 400 MHz, $CDCl_3$ of compound **34**

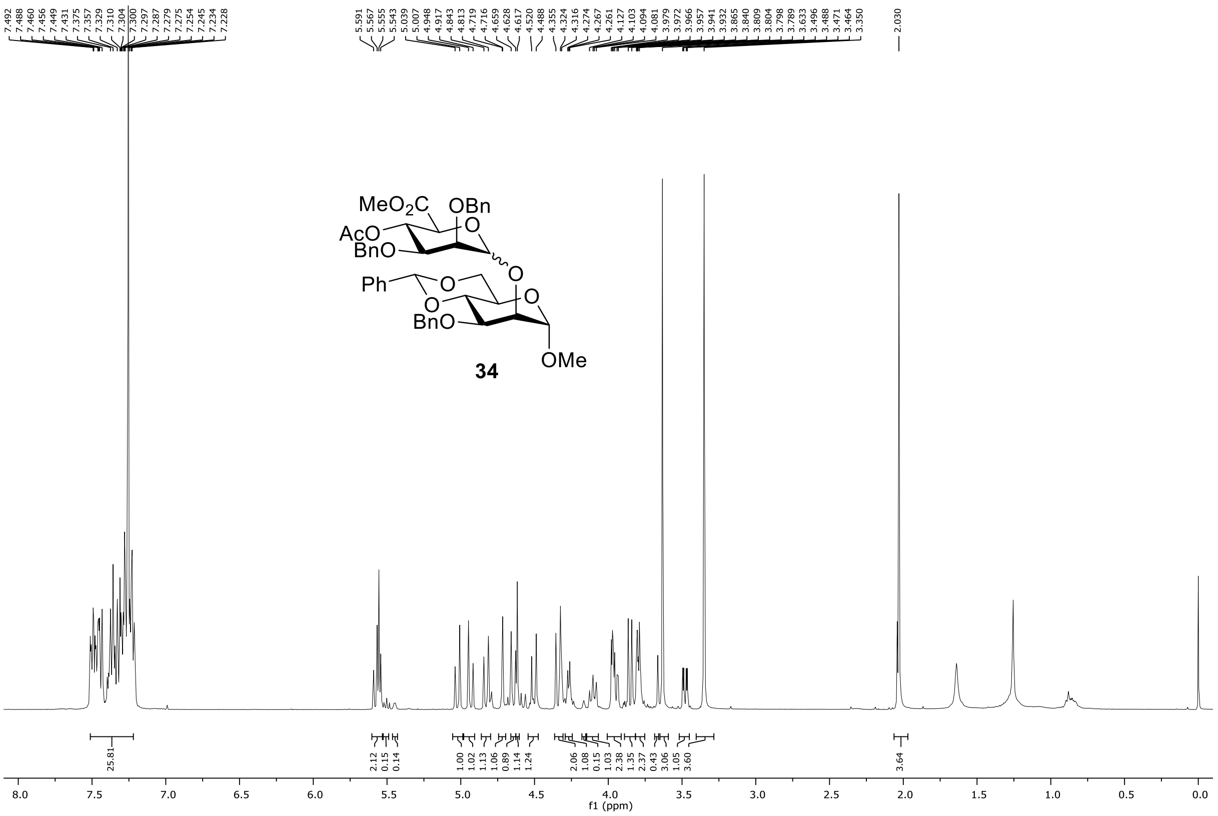

^{13}C-APT NMR, 101 MHz, $CDCl_3$ of compound **34**

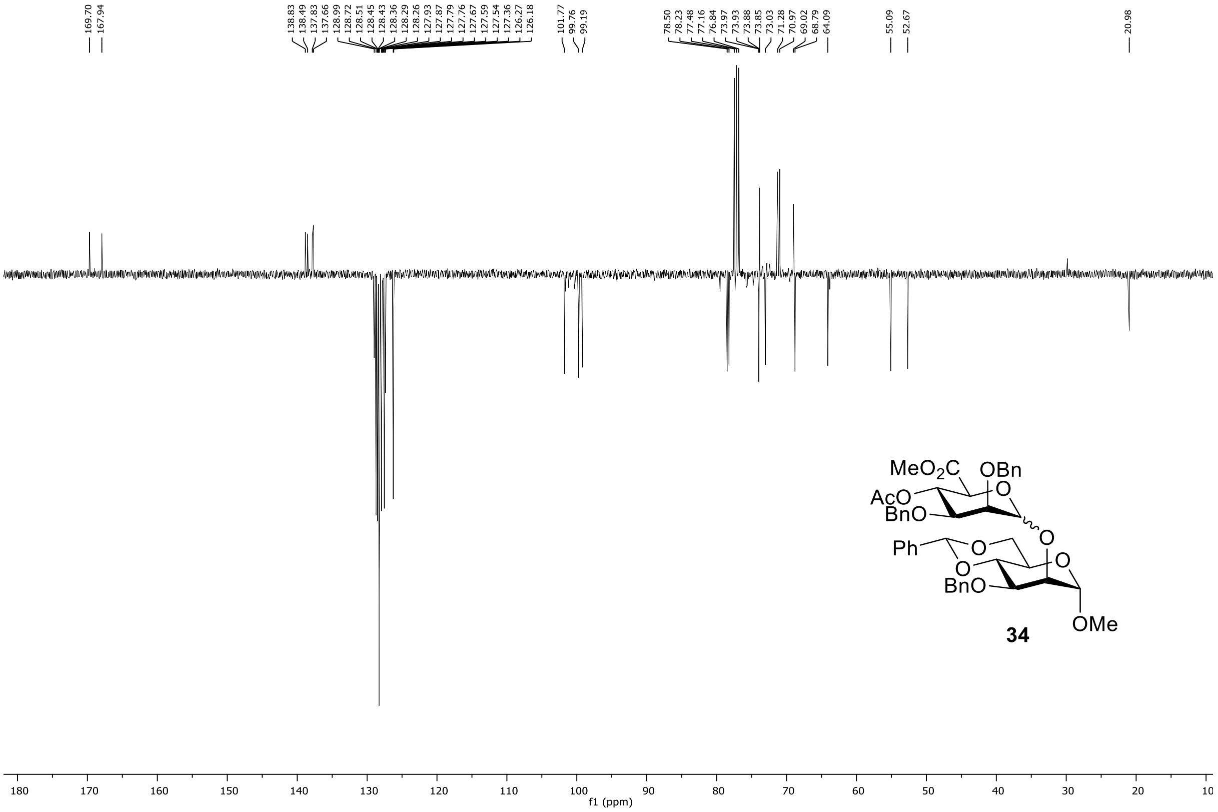

COSY NMR of compound **34**

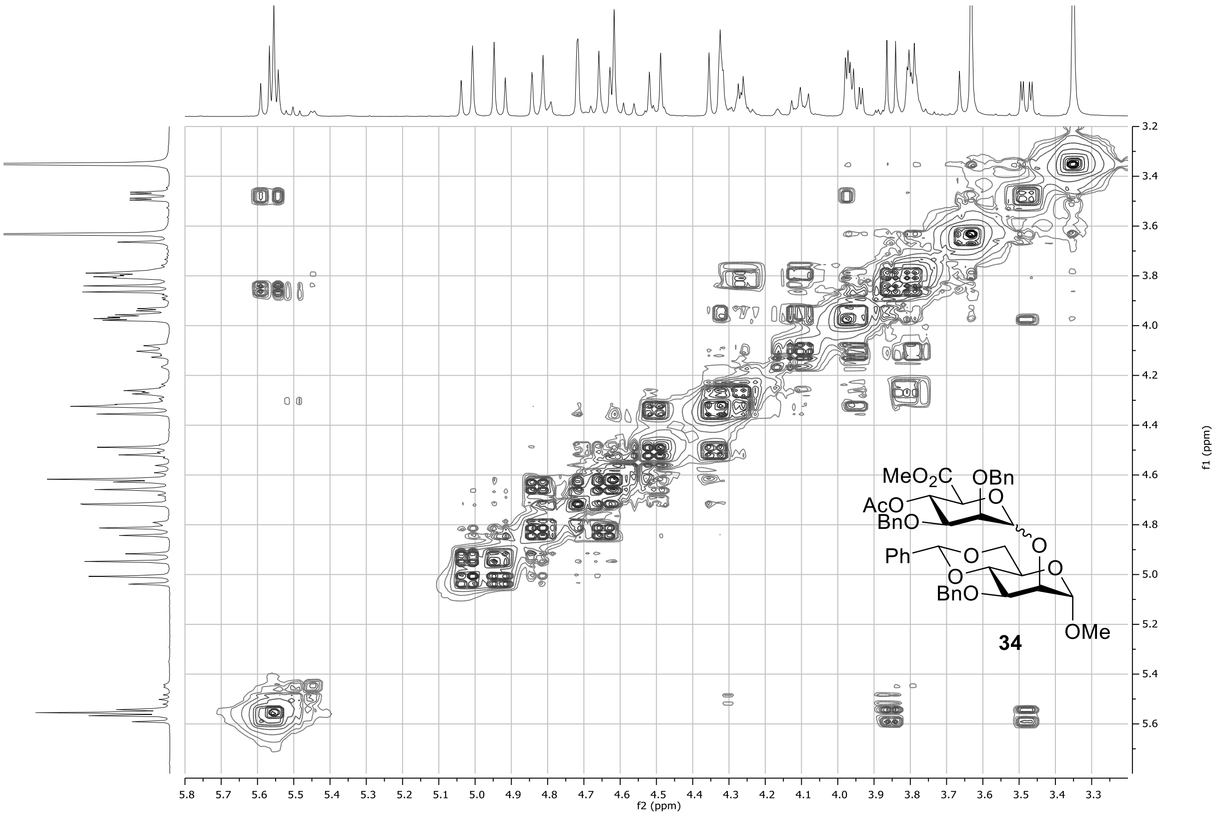

HSQC NMR of compound **34**

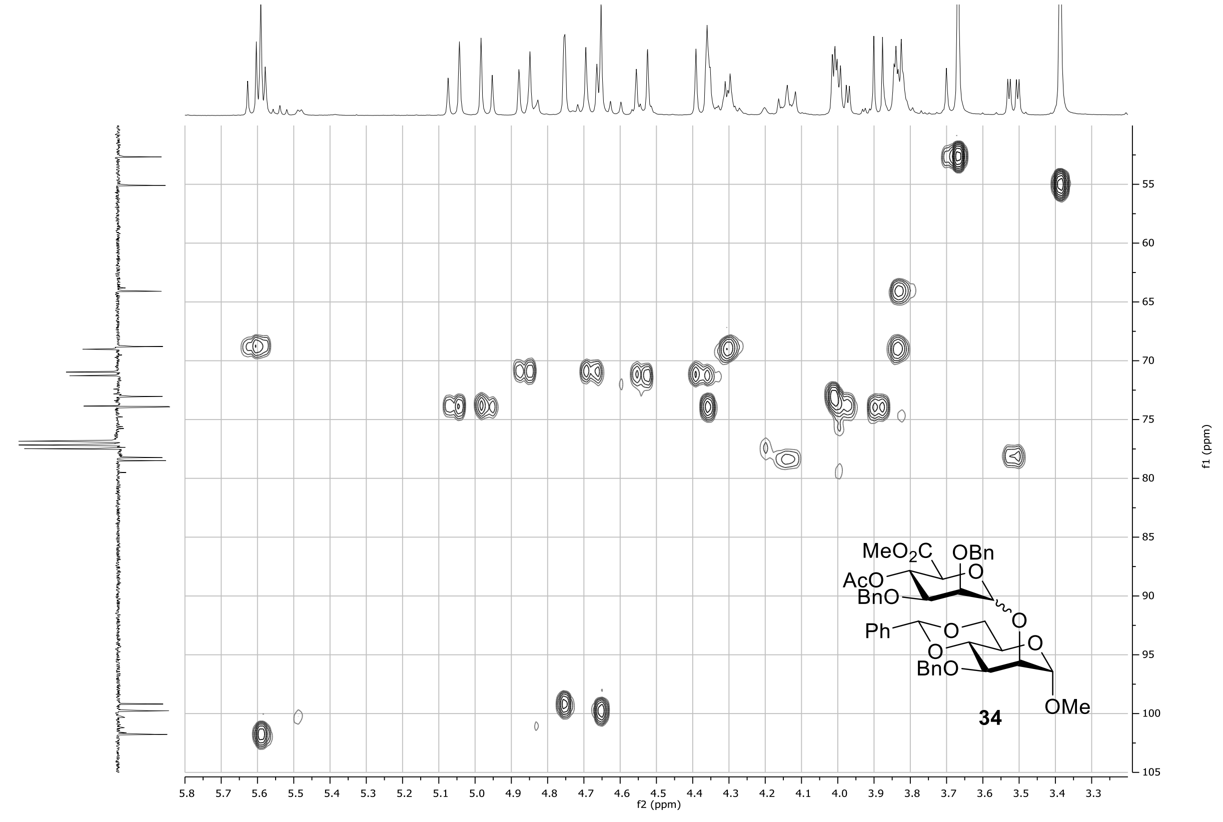

HMBC NMR of compound **34**

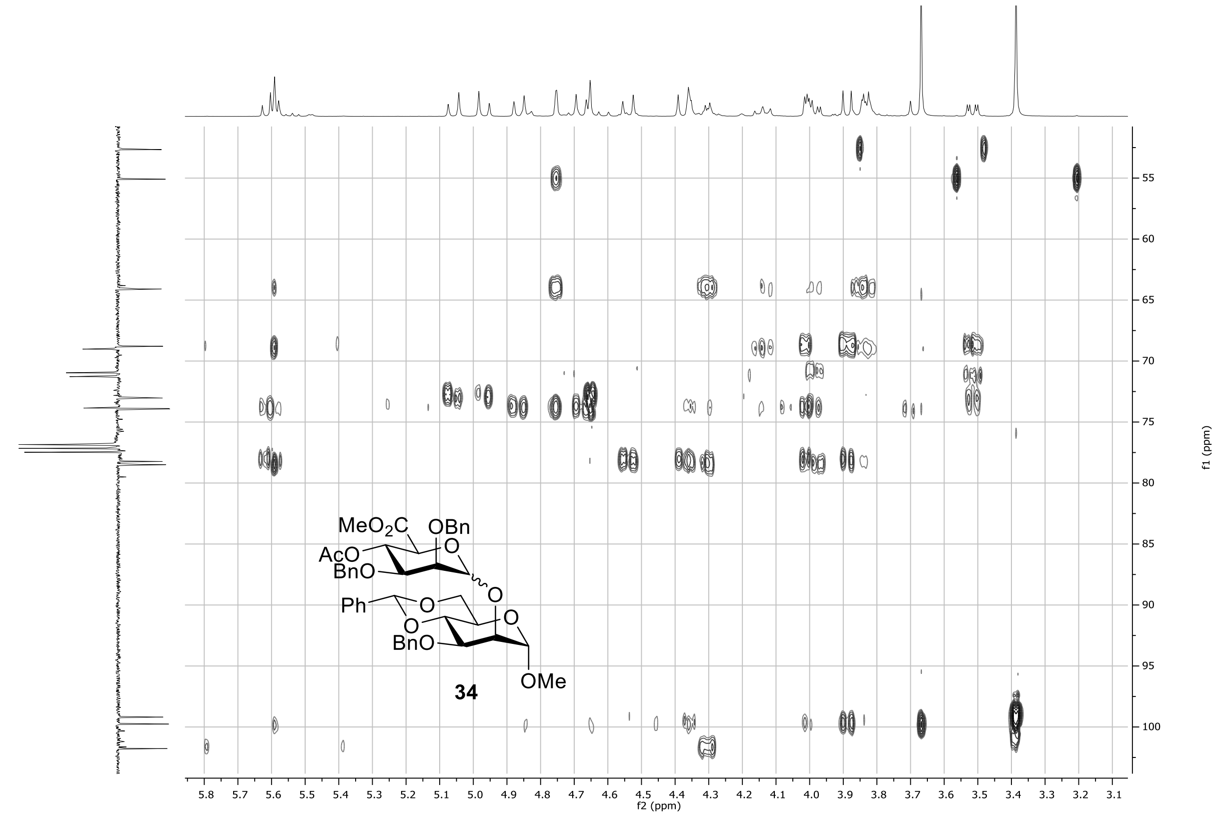

Methyl 2,3,4-tri-*O*-methyl mannopyranosyl uronate oxocarbenium ion atom coordinates and energies of the $^3H_4$, $^4H_3$, $B_{2,5}$ conformations

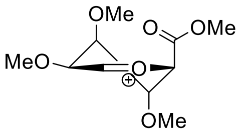

S18 ($^3H_4$)
0 kcal mol^{-1}

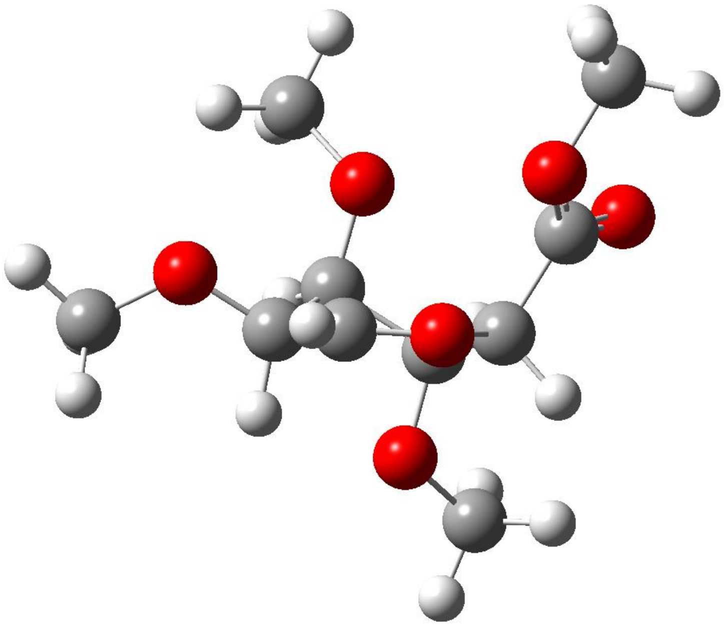

```
Gas phase energy =  -842.538825958 Hartree

 Zero-point correction=                           0.272524 (Hartree/Particle)
 Thermal correction to Energy=                    0.290829
 Thermal correction to Enthalpy=                  0.291773
 Thermal correction to Gibbs Free Energy=         0.224624
 Sum of electronic and zero-point Energies=           -842.266302
 Sum of electronic and thermal Energies=              -842.247997
 Sum of electronic and thermal Enthalpies=            -842.247053
 Sum of electronic and thermal Free Energies=         -842.314202

Solvated (methylene chloride) energy = -842.605384995 Hartree

Total energy: (-842.605384995 + 0.272524) Hartree
```

* 627.509469 kcal mol^{-1} Hartree^{-1} = -528571.845 kcal mol^{-1}

Center Number	Atomic Number	Atomic Type	Coordinates (Angstroms) X	Y	Z
1	6	0	0.998510	0.913869	-0.571364
2	6	0	-0.021284	1.268433	0.521163
3	6	0	-0.943782	0.066979	0.782220
4	6	0	-1.719208	-0.205693	-0.525454
5	6	0	-0.774646	-0.331514	-1.663688
6	8	0	0.366935	0.182969	-1.716623
7	6	0	2.200015	0.106263	-0.069813
8	8	0	-2.513413	-1.358974	-0.502794
9	8	0	-0.113465	-1.021864	1.105770
10	8	0	-0.838877	2.342997	0.115048
11	8	0	2.410221	-1.005123	-0.751611
12	8	0	2.865832	0.537257	0.835501
13	6	0	3.546837	-1.807672	-0.333305
14	6	0	-0.349455	3.640498	0.475033
15	6	0	-0.664580	-1.988750	2.010564
16	6	0	-3.919252	-1.127016	-0.318667
17	1	0	1.389432	1.811168	-1.060195
18	1	0	0.540859	1.493889	1.435258
19	1	0	-1.644265	0.317446	1.587998
20	1	0	-2.293334	0.710222	-0.776227
21	1	0	-1.058999	-0.904758	-2.555971
22	1	0	3.558044	-2.656429	-1.011290
23	1	0	4.463550	-1.225027	-0.421186
24	1	0	3.407502	-2.133257	0.697333
25	1	0	-0.242499	3.722357	1.561576
26	1	0	0.612395	3.855660	-0.004060
27	1	0	-1.090826	4.355942	0.123457
28	1	0	-1.500604	-2.520232	1.552232
29	1	0	0.139307	-2.688960	2.232685
30	1	0	-0.989456	-1.503551	2.937141
31	1	0	-4.386652	-2.109729	-0.301400
32	1	0	-4.113785	-0.613880	0.628670
33	1	0	-4.327461	-0.541342	-1.148481

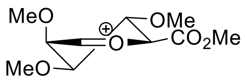

S18 ($^{4}H_{3}$)
+ 5.0 kcal mol^{-1}

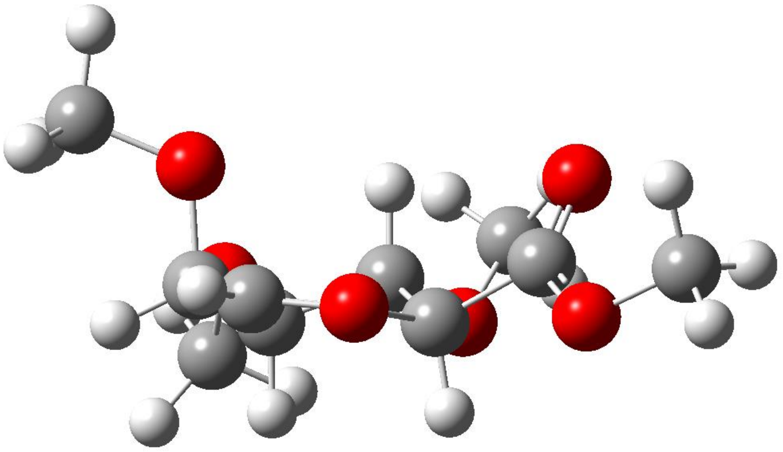

Gas phase energy = -842.528599137 Hartree

Zero-point correction=	0.272955 (Hartree/Particle)
Thermal correction to Energy=	0.291338
Thermal correction to Enthalpy=	0.292282
Thermal correction to Gibbs Free Energy=	0.225197
Sum of electronic and zero-point Energies=	-842.255644
Sum of electronic and thermal Energies=	-842.237261
Sum of electronic and thermal Enthalpies=	-842.236317
Sum of electronic and thermal Free Energies=	-842.303402

Solvated (methylene chloride) energy = -842.597802066 Hartree

Total energy: (-842.597802066 + 0.272955) Hartree

* 627.509469 kcal mol^{-1} Hartree^{-1} = -528566.818 kcal mol^{-1}

Relative energy to $^{3}H_{4}$: +5.03 kcal mol^{-1}

Center Number	Atomic Number	Atomic Type	Coordinates (Angstroms) X	Y	Z
1	6	0	1.005063	-0.073754	-0.638312
2	6	0	-0.150594	0.692640	0.006931
3	6	0	-1.477849	0.281978	-0.636583
4	6	0	-1.661319	-1.227993	-0.492138
5	6	0	-0.430310	-2.020045	-0.779304
6	8	0	0.735326	-1.548995	-0.762598
7	6	0	2.321347	0.010106	0.148169
8	8	0	-1.731255	-1.612307	0.883241
9	8	0	-2.561987	0.918447	-0.001455
10	8	0	0.130612	2.053157	-0.246676
11	8	0	3.318499	-0.470356	-0.580168
12	8	0	2.401671	0.453461	1.264244
13	6	0	4.629328	-0.490867	0.046913
14	6	0	0.154644	2.896603	0.915304
15	6	0	-3.204678	1.935251	-0.779107
16	6	0	-2.925329	-2.318394	1.276371
17	1	0	1.164778	0.251724	-1.672067
18	1	0	-0.185976	0.481224	1.079375
19	1	0	-1.438093	0.517906	-1.711253
20	1	0	-2.512417	-1.592127	-1.079055
21	1	0	-0.473437	-3.109578	-0.889628
22	1	0	5.292920	-0.921729	-0.697513
23	1	0	4.932334	0.525272	0.299086
24	1	0	4.599049	-1.106180	0.946177
25	1	0	0.371522	3.900645	0.552820
26	1	0	-0.816045	2.890061	1.419092
27	1	0	0.937660	2.577181	1.607738
28	1	0	-3.641541	1.515979	-1.692561
29	1	0	-4.000257	2.338710	-0.153962
30	1	0	-2.503890	2.732814	-1.040841
31	1	0	-3.022988	-3.262496	0.733322
32	1	0	-2.812493	-2.516244	2.340574
33	1	0	-3.795874	-1.683572	1.096524

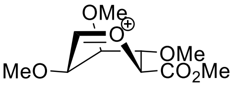

S18 ($B_{2,5}$)
+ 6.1 kcal mol^{-1}

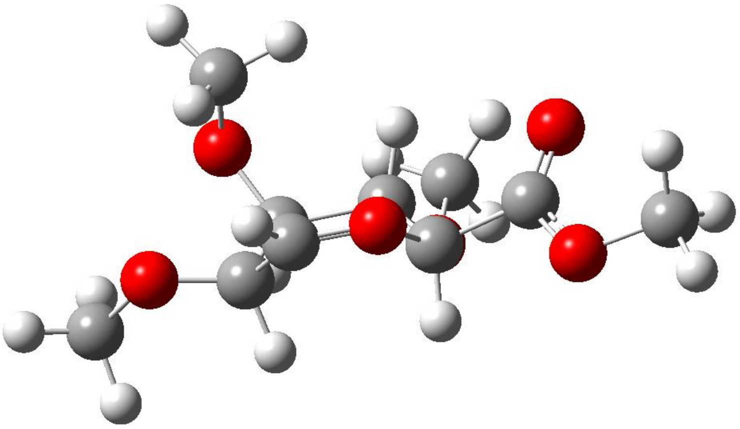

Gas Phase Energy = -842.526083446 Hartree

Zero-point correction=	0.272637 (Hartree/Particle)
Thermal correction to Energy=	0.290545
Thermal correction to Enthalpy=	0.291489
Thermal correction to Gibbs Free Energy=	0.225398
Sum of electronic and zero-point Energies=	-842.253447
Sum of electronic and thermal Energies=	-842.235539
Sum of electronic and thermal Enthalpies=	-842.234594
Sum of electronic and thermal Free Energies=	-842.300686

Solvated (methylene chloride) energy = -842.595751241 Hartree

Total energy: (-842.595751241 + 0.272637) Hartree
* 627.509469 kcal mol^{-1} Hartree^{-1} = -528565.730 kcal mol-1

Relative energy to $^3H_4$: +6.12 kcal mol^{-1}

Center Number	Atomic Number	Atomic Type	Coordinates (Angstroms) X	Y	Z
1	6	0	0.951799	-0.354386	-0.392622
2	6	0	0.104904	0.881365	-0.093108
3	6	0	-1.423336	0.631498	-0.199092
4	6	0	-1.730559	-0.912199	-0.500957
5	6	0	-0.826734	-1.765417	0.267468
6	8	0	0.415807	-1.542707	0.328512
7	6	0	2.399514	-0.216937	0.097813
8	8	0	-3.031730	-1.318598	-0.245004
9	8	0	-2.180098	1.083858	0.884791
10	8	0	0.550778	1.820439	-1.048358
11	8	0	3.159170	-1.141783	-0.468968
12	8	0	2.754624	0.614603	0.891892
13	6	0	4.557097	-1.166944	-0.071314
14	6	0	0.604267	3.170305	-0.565311
15	6	0	-1.956782	0.451837	2.138999
16	6	0	-4.032709	-0.756005	-1.106015
17	1	0	0.929132	-0.607587	-1.459804
18	1	0	0.350224	1.217566	0.919361
19	1	0	-1.780396	1.192649	-1.064659
20	1	0	-1.427375	-1.046353	-1.567504
21	1	0	-1.174106	-2.644581	0.825399
22	1	0	4.998395	-1.981390	-0.638788
23	1	0	5.026710	-0.215931	-0.322107
24	1	0	4.632446	-1.351163	1.000327
25	1	0	-0.387560	3.524236	-0.265372
26	1	0	1.297166	3.247730	0.278534
27	1	0	0.968064	3.774865	-1.394533
28	1	0	-0.943191	0.618409	2.519212
29	1	0	-2.672755	0.885721	2.834996
30	1	0	-2.157529	-0.628744	2.074918
31	1	0	-4.958654	-1.272238	-0.860805
32	1	0	-4.151573	0.314193	-0.919609
33	1	0	-3.782161	-0.932585	-2.157653
